# Investigating the Structure–Activity Relationship of 1,2,4-Triazine G-Protein-Coupled Receptor 84 (GPR84) Antagonists

**DOI:** 10.1021/acs.jmedchem.2c00804

**Published:** 2022-08-10

**Authors:** Amit Mahindra, Laura Jenkins, Sara Marsango, Mark Huggett, Margaret Huggett, Lindsay Robinson, Jonathan Gillespie, Muralikrishnan Rajamanickam, Angus Morrison, Stuart McElroy, Irina G. Tikhonova, Graeme Milligan, Andrew G. Jamieson

**Affiliations:** † School of Chemistry, University of Glasgow, Joseph Black Building, University Avenue, Glasgow G12 8QQ, U.K.; ‡ Centre for Translational Pharmacology, Institute of Molecular, Cell and Systems Biology, University of Glasgow, Davidson Building, Glasgow G12 8QQ, U.K.; § BioAscent Discovery Ltd., Newhouse, Lanarkshire ML1 5UH, U.K.; ∥ European Screening Centre, University of Dundee, Newhouse, Lanarkshire ML1 5UH, U.K.; ⊥ School of Pharmacy, Medical Biology Centre, Queen’s University Belfast, Belfast BT9 7BL, U.K.

## Abstract

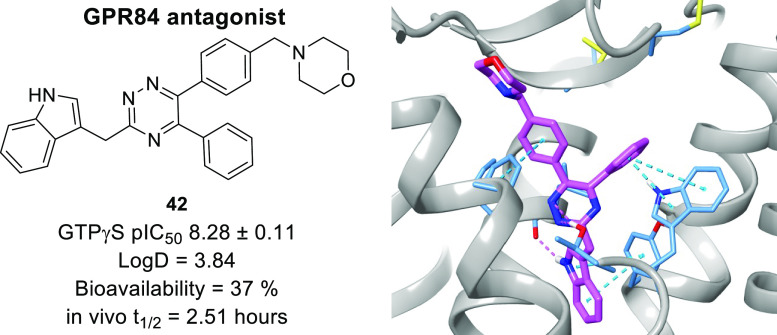

G-protein-coupled receptor 84 (GPR84) is a proinflammatory orphan G-protein-coupled receptor implicated in several inflammatory and fibrotic diseases. Several agonist and antagonist ligands have been developed that target GPR84; however, a noncompetitive receptor blocker that was progressed to phase II clinical trials failed to demonstrate efficacy. New high-quality antagonists are required to investigate the pathophysiological role of GPR84 and to validate GPR84 as a therapeutic target. We previously reported the discovery of a novel triazine GPR84 competitive antagonist **1**. Here, we describe an extensive structure–activity relationship (SAR) of antagonist **1** and also present in silico docking with supporting mutagenesis studies that reveals a potential binding pose for this type of orthosteric antagonist. Lead compound **42** is a potent GPR84 antagonist with a favorable pharmacokinetic (PK) profile suitable for further drug development.

## 1 Introduction

GPR84 is a poorly characterized rhodopsin-like G-protein-coupled receptor expressed predominantly by immune cell types.^1,2^ Upregulation of GPR84 occurs in proinflammatory conditions, and thus it is considered a promising therapeutic target in inflammatory conditions, including ulcerative colitis and fibrotic diseases.^3^

**Figure 1:**
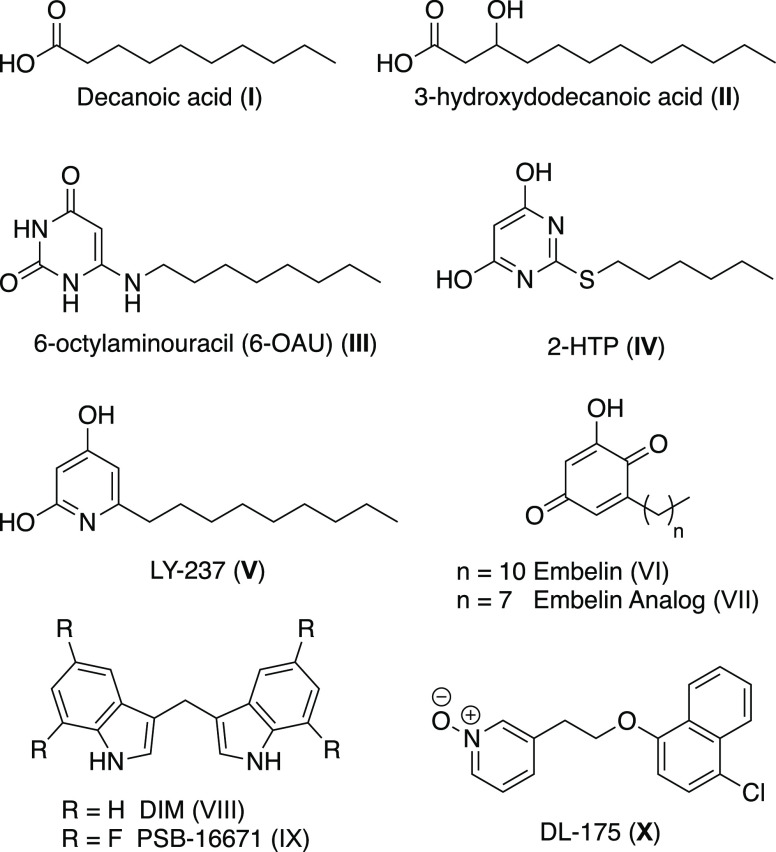
Chemical structures of known GPR84 activators.

GPR84 is activated by medium-chain free fatty acids (MCFAs) with chain lengths of C10–12. However, even decanoic acid **I** (Figure 1) has only moderate potency as an agonist and, as such, GPR84 is still classed as an orphan receptor.^4,5^ Studies have demonstrated that GPR84 is also activated by hydroxylated MCFAs such as 3-hydroxydodecanoic acid **II** (Figure 1).^6^ The first synthetic orthosteric GPR84 agonist to be reported was 6-octylaminouracil (6-OAU) **III** (Figure 1), discovered from a high-throughput screen (HTS) of a small-molecule library using a GTPγS binding assay.^6,7^ In a different HTS, the thiopyrimidine agonist 2-(hexylthio)pyrimidine-4,6 diol (2-HTP) **IV** (Figure 1) was discovered to have a markedly higher potency than the MCFAs.^8^ Subsequent optimization of 2-(hexylthio)pyrimidine-4,6 diol (2-HTP) led to the development of the 2,4-dihydroxypyridine agonist LY-237 **V** (Figure 1) with 3 orders of magnitude improved potency over 2-(hexylthio)pyrimidine-4,6 diol (2-HTP).^9^ Embelin **VI** (Figure 1), a 2-hydroxy-1,4-benzoquinone natural product isolated from the plant *Embelia ribes* (Myrsinaceae), also activates GPR84 with moderate potency.^10^ A structure–activity relationship (SAR) study of embelin led to an analogue **VII** (Figure 1) with 45-fold higher potency at human GPR84 over embelin.^11^ Diindolylmethane (DIM) **VIII** (Figure 1) is another naturally occurring compound that activates GPR84.^12^ However, DIM acts as an ago-allosteric agonist of GPR84, binding at a site distinct from MCFAs and the other orthosteric ligands described above.^13,14^ SAR studies of DIM resulted in the development of the tetrafluoro analogue PSB-16671 **IX** (Figure 1) as a more potent GPR84 activator.^15,16^ Biased agonist DL-175 **X** was discovered from a library screen against a predictive quantitative structure–activity relationship (QSAR) model and subsequent hit optimization.^17^

**Figure 2:**
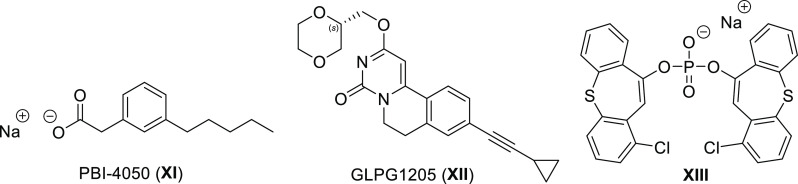
Chemical structures of selected GPR84 antagonists.

Proinflammatory stimuli increase the expression of GPR84 and, therefore there is significant interest in assessing whether antagonism of the receptor might provide therapeutic benefit in a range of inflammatory conditions, either by restricting the movement of immune cells into inflamed tissue or by enhancing disease resolution.^3,4,6^ Liminal Biosciences (previously Prometic Life Sciences, Inc.) has identified a low-affinity GPR84 antagonist, 2-(3-pentylphenyl)acetic acid sodium salt, PBI-4050 **XI** (Figure 2) that also binds to other targets, which reached phase III clinical trials for Alström Syndrome.^18,19^ Recently Galapagos NV used a small-molecule HTS to identify GPR84 antagonists.^20^ Subsequent optimization generated a series of GPR84 antagonists including 9-cyclopropylethynyl-2-((*S*)-1-[1,4]dioxan-2-ylmethoxy)-6,7-dihydropyrimido[6,1-*a*]isoquinolin-4-one, GLPG 1205 **XII** (Figure 2) a potent and selective GPR84 antagonist.^21^ This compound was shown to reduce disease activity index score and neutrophil infiltration in a mouse dextran sodium sulfate-induced chronic inflammatory bowel disease model and, although failing to achieve predefined efficacy endpoints in clinical studies of ulcerative colitis this ligand has also been assessed for the treatment of idiopathic pulmonary fibrosis. Recently, Chen et al. published a series of phosphodiester GPR84 antagonists and showed the ability of an exemplar molecule (**XIII**) to inhibit the chemotaxis of mouse neutrophils and macrophages upon GPR84 activation.^22^

Better understanding of the pathophysiological roles of GPR84, and its potential as a therapeutic target in a range of conditions would be enhanced by the availability of a wider range of receptor antagonists. Therefore, there is currently a need to identify new ligands for GPR84 that can be used as chemical probes or lead compounds for drug development.

**Figure 3:**
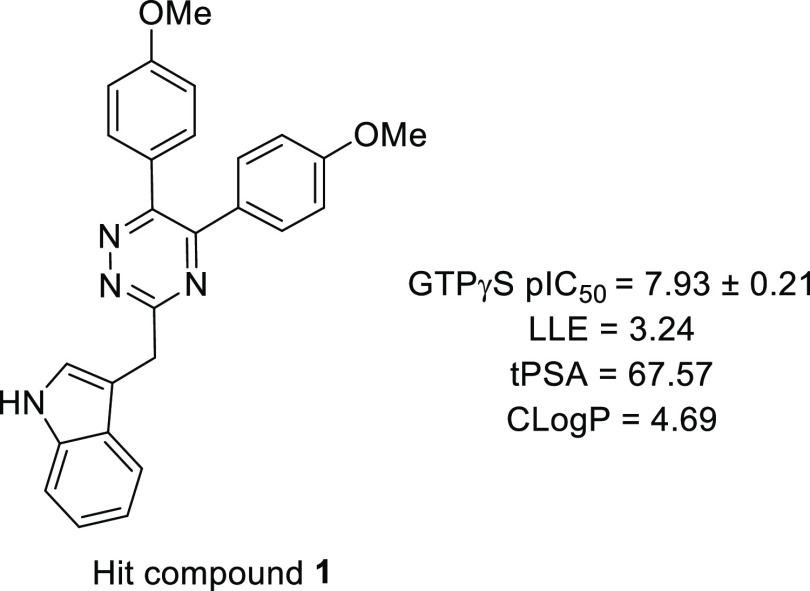
Chemical structure, calculated physicochemical properties, and bioactivity of hit 1,2,4-triazine compound **1**.

We recently reported the discovery of 3-((5,6-bis(4-methoxyphenyl)-1,2,4-triazin-3-yl)methyl)-1*H*-indole **1** (Figure 3) as the first, high-affinity and highly selective competitive antagonist of human GPR84.^23^ In this work, we report a substantial SAR of this compound and novel highly potent analogues with improved druglike properties. The SAR analysis together with a docking study provides a better understanding of the potential antagonist mode of binding to GPR84 and build a foundation for further compound optimization.

## 2 Results and Discussion

### 2.1 Chemistry

1,2,4-Triazines and their derivatives are a class of azaheterocycles with a broad spectrum of biological activities and are thus privileged scaffolds in medicinal chemistry.^24,25^

The predominant method used for the synthesis of substituted 1,2,4-triazines is the condensation reaction of a 1,2-dicarbonyl with the corresponding acid hydrazide.^26^ This chemistry is most often used for preparing 1,2,4-triazines for which the 5- and 6-substituents are identical. A limitation of this chemistry is that reaction of unsymmetrical 1,2-diketones produces regioisomeric mixtures.^27^

**Scheme 1:**
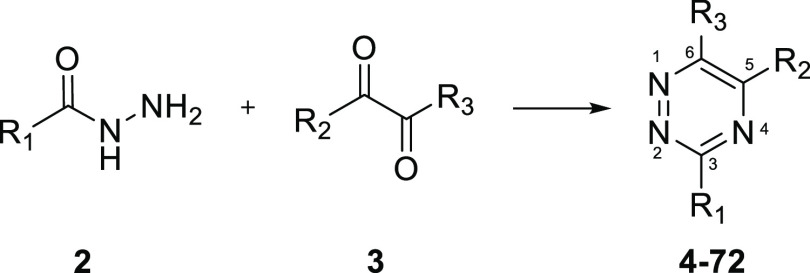
Synthesis of 3,5,6-Trisubstituted 1,2,4-Triazines **4-72** via Condensation of 1,2-Diketones

SAR optimization was initiated with the aim to improve the potency and druglike properties of compound **1** (Figure 3). The general synthetic strategy used for the preparation of this series of compounds is summarized in Scheme 1. The required hydrazides were either sourced commercially or prepared by reported procedures.^28−30^ Hydrazides were prepared from the corresponding esters by refluxing with hydrazine in ethanol. The hydrazides were then reacted with symmetrical and unsymmetrical 1,2-diketones (Scheme 1) in the presence of ammonium acetate and acetic acid to afford the 3,5,6-trisubstituted 1,2,4-triazines **4**–**72**. Regioisomers produced by reacting unsymmetrical 1,2-diketones were separated by supercritical fluid chromatography (SFC).

**Figure 4:**
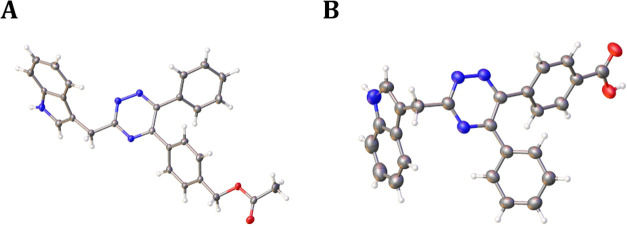
X-ray crystal structures of (A) **37** and (B) **52** (carbon = gray, hydrogen = white, oxygen = red, nitrogen = blue).

Unsymmetrical 1,2-diketones react with hydrazides to produce mixtures of 5,6-diaryl triazine regioisomers. The accurate structure of this series of molecules was identified by X-ray crystallography for compounds **37** and **52** (Figure 4) and extrapolation through relative reversed-phase high-performance liquid chromatography (RP-HPLC) retention times.

**Scheme 2:**
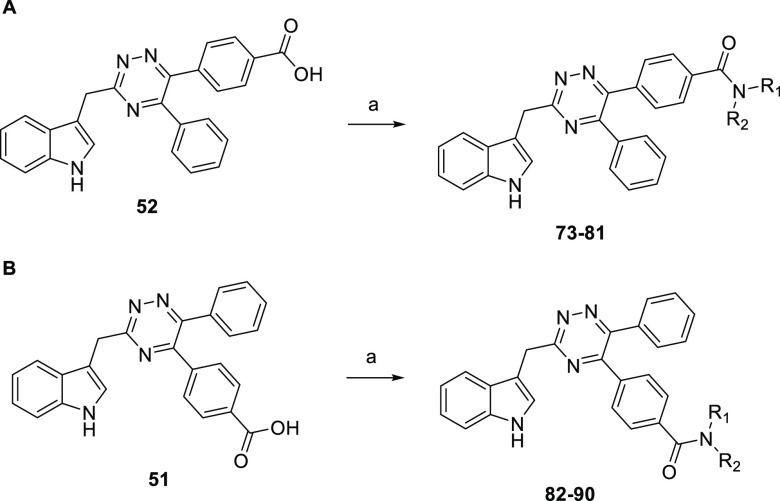
Late-Stage Structural Diversification of (A) **52** to Yield **73**–**81** (B) **51** to Yield Amides **82**–**90** (R_1_ and R_2_ Defined in Table 8).

After confirming the configuration of **52** with phenyl in the 5-position and benzoic acid in the 6-position of the 1,2,4-triazines, we exploited the carboxylic acid functionality to structurally diversify and produce amides **73**–**81** (Scheme 2). Similarly, the other regioisomer **51** was functionalized leading to compounds **82**–**90**.

### 2.2 In Silico Docking

**Figure 5:**
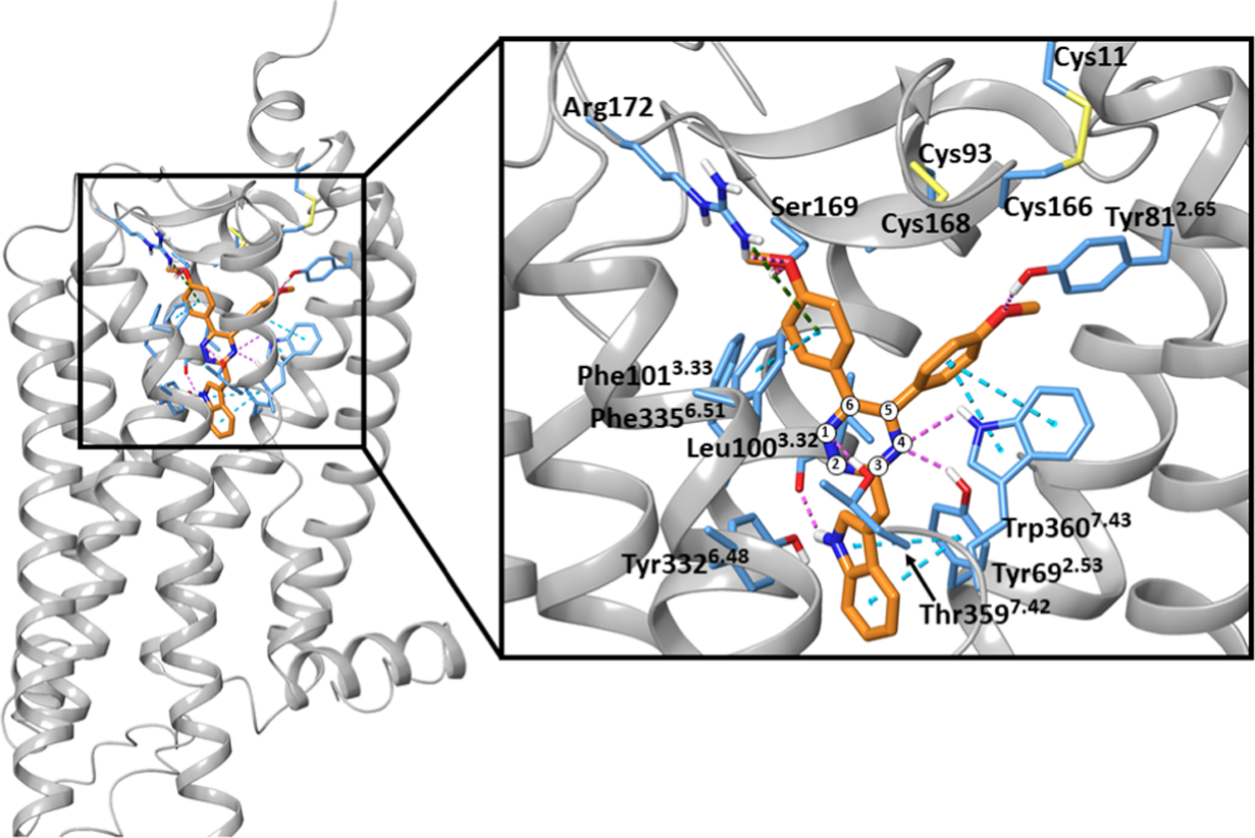
Proposed binding mode of compound **1** at the human GPR84 receptor Alphafold (AF-Q9NQS5-F1). The overall view of the ligand–receptor complex and the zoomed ligand binding site are on the left and right. The ligand and the amino acids are shown by orange and blue sticks, respectively. Hydrogen bonds, π–π, and cation−π interactions are in pink, cyan, and green dashed lines, respectively. A section of helix 7 was hidden for a clear view in the zoomed image. The 1,2,4-triazine ring is numbered.

A molecular docking study was carried out to provide a structural hypothesis of an antagonist binding mode. An experimentally determined structure of GPR84 is not available. The AlphaFold structure of human GPR84 generated by the AlphaFold deep learning algorithm^31^ was thus used for antagonist docking. In our previous work, this structure was used effectively in docking of the agonist compounds 2-HTP, 6-OAU, and DL-175 and to provide an explanation of their functional bias,^32^ so it was applicable to the current study. A key feature of both this model and our previously generated homology model^13^ is that Arg172, located within extracellular loop 2 (ECL2), points inward toward the binding cavity to coordinate the carboxylate of medium-chain fatty acids that are the native activators of the receptor. This is very different from other homology models^33,34^ where the fatty acid was predicted to have the opposite orientation, with the carboxylate located deep with the transmembrane helix bundle and interacting with Asn104. The preferred binding pose of compound **1** is shown in Figure 5. Similar to the agonists, compound **1** is predicted to sit within the helical bundle forming direct contacts with helices 2, 3, 6, and 7 and extracellular loop 2 (ECL2). This is consistent with functional and pharmacological studies that show compound **1** to act as a competitive antagonist of the agonists noted above.^23^ In particular, the indole functionality is predicted to project from the 3-position of the triazine ring into the bottom of the extracellular cavity. This indole is likely to form a H-bond with the backbone carbonyl of Leu100^3.32^ (The GPCR Ballesteros–Weinstein numbering is provided in the superscript.^35^) A π-stacking interaction is also predicted to occur between Tyr69^2.53^ and the indole ring.

In the selected docking pose, the anisole substituent in the 6-position of the 1,2,4-triazine forms π–π interactions with Phe101^3.33^ and Phe335^6.51^ and cation−π interaction with Arg172 of ECL2. The 4-methoxy group then projects up toward the extracellular opening and makes a H-bond interaction with Ser169 of ECL2. The anisole substituent in the 5-position is predicted to be buried in a pocket beneath the β-sheets of ECL2. In this pocket, Trp360^7.43^ may form a T-shaped π–π stacking interaction with the anisole ring. A H-bond between the side-chain hydroxyl of Tyr81^2.65^ and the methoxy group is also likely to occur. However, there appears to be limited space for aryl substituents in this pocket as two disulfide bridges appear to restrict the mobility of ECL2.

**Figure 6:**
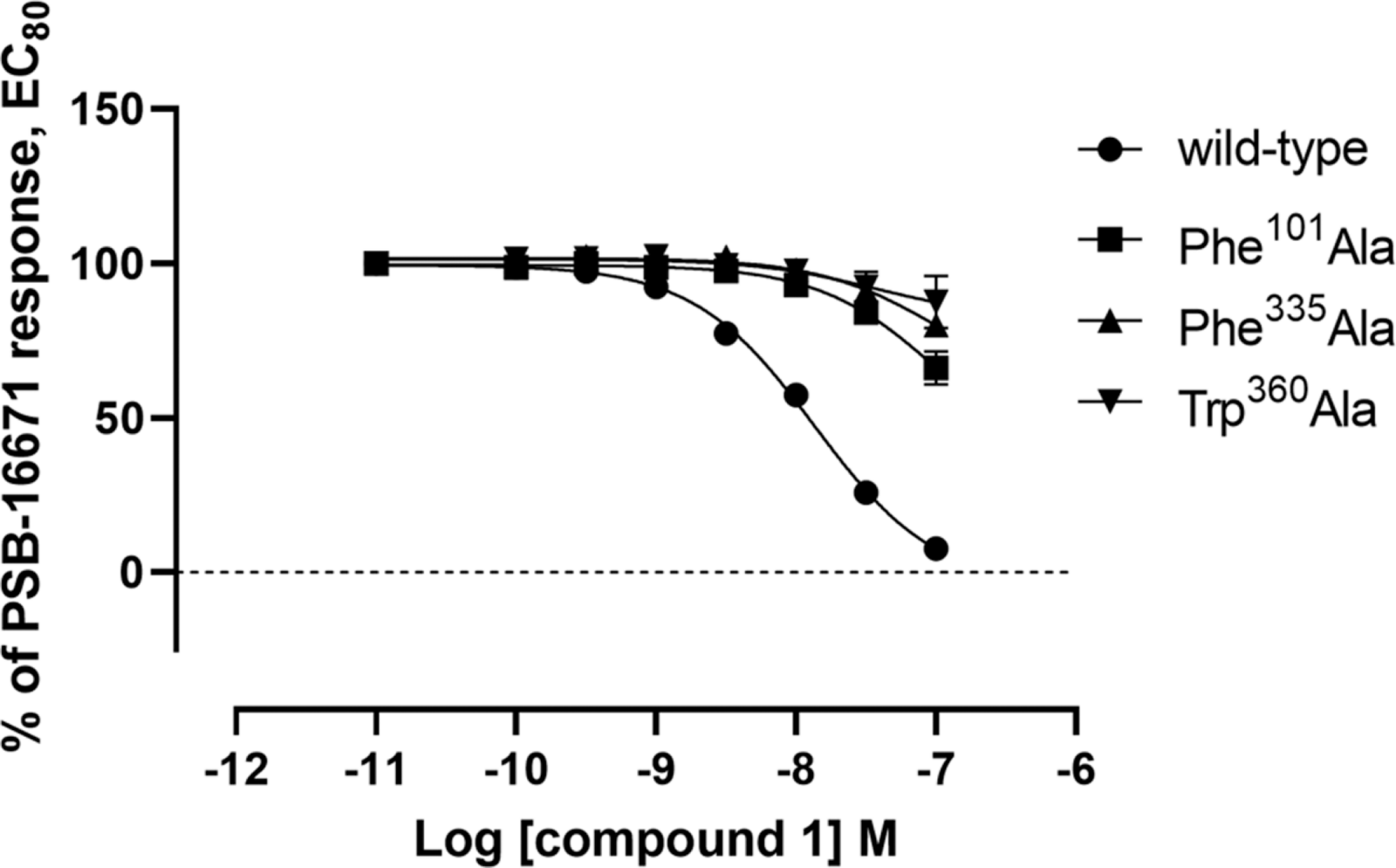
[^35^S]GTPγS binding was performed on membrane preparations from Flp-In T-REx 293 cells expressing the indicated GPR84-Gα_i2_ fusion proteins. Conversion of each of Phe101, Phe335, and Trp360 to Ala resulted in a substantial loss of affinity of compound **1** to block effects of the allosteric agonist PSB-16671. PSB-16671 showed pEC50 = 6.70 ± 0.05 at wild type, 6.97 ± 0.29 at Phe101Ala, 5.88 ± 0.02 at Phe335Ala, and 7.17 ± 0.08 at Trp360Ala.

Initial mutagenesis studies provided support for this docking pose where an alteration to Ala of each of Phe101^3.33^, Phe335^6.51^, and Trp360^7.43^ resulted in substantial loss of affinity of compound **1** to antagonize PSB-16671-promoted binding of [^35^S]GTPγS (Figure 6). The obtained binding pose of compound **1** was thus used to rationalize the SAR of analogues.

### 2.3 Structure–Activity Relationship

The structural requirements of symmetrical 3-methylindole-5,6-trisubstituted 1,2,4-triazines were investigated first (Table 1). Hit compound **1** incorporates anisole groups in the 5- and 6-positions of the triazine ring that face ECL2 in the docking model. Substitution of these rings with halides (compounds **5**–**7**) decreased activity in an atomic-size-dependent manner. The larger and more electronegative pyridine analogue **8** also lost significant activity. A decrease in activity is observed for compound **9**, which incorporates isopropyl groups with central sp^3^-hybridized carbons, suggesting hindrance with the buried ECL2 in the binding site. Indeed, compound **9** could be docked to the site only when the van der Waals radii of receptor atoms were reduced. The two predicted Cys11–Cys166 and Cys93–Cys168 disulfide bridges between ECL2 and the N-terminus of helix 3 would restrain the position of the loop and reduce its flexibility. Likewise, analogue **10** incorporating a large substituent on the phenyl ring loses significant activity. Increasing the size of these aryl substituents is clearly disfavored with a steady decrease in potency as the size increases. Removing the aryl substituents altogether gave the best results with diphenyl analogue **4**, the most potent ligand identified (pIC_50_ 8.14 ± 0.14), although with a lower than desired LLE value. Thiophene analogue **11**, which is smaller, although more polar than the phenyl analogue **4** resulted in a modest decrease in activity.

**Table 1:**
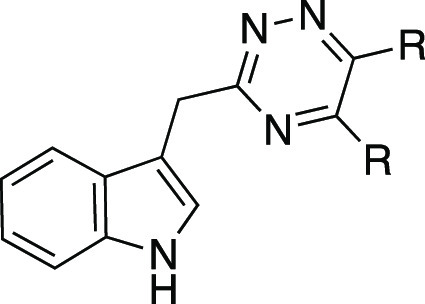

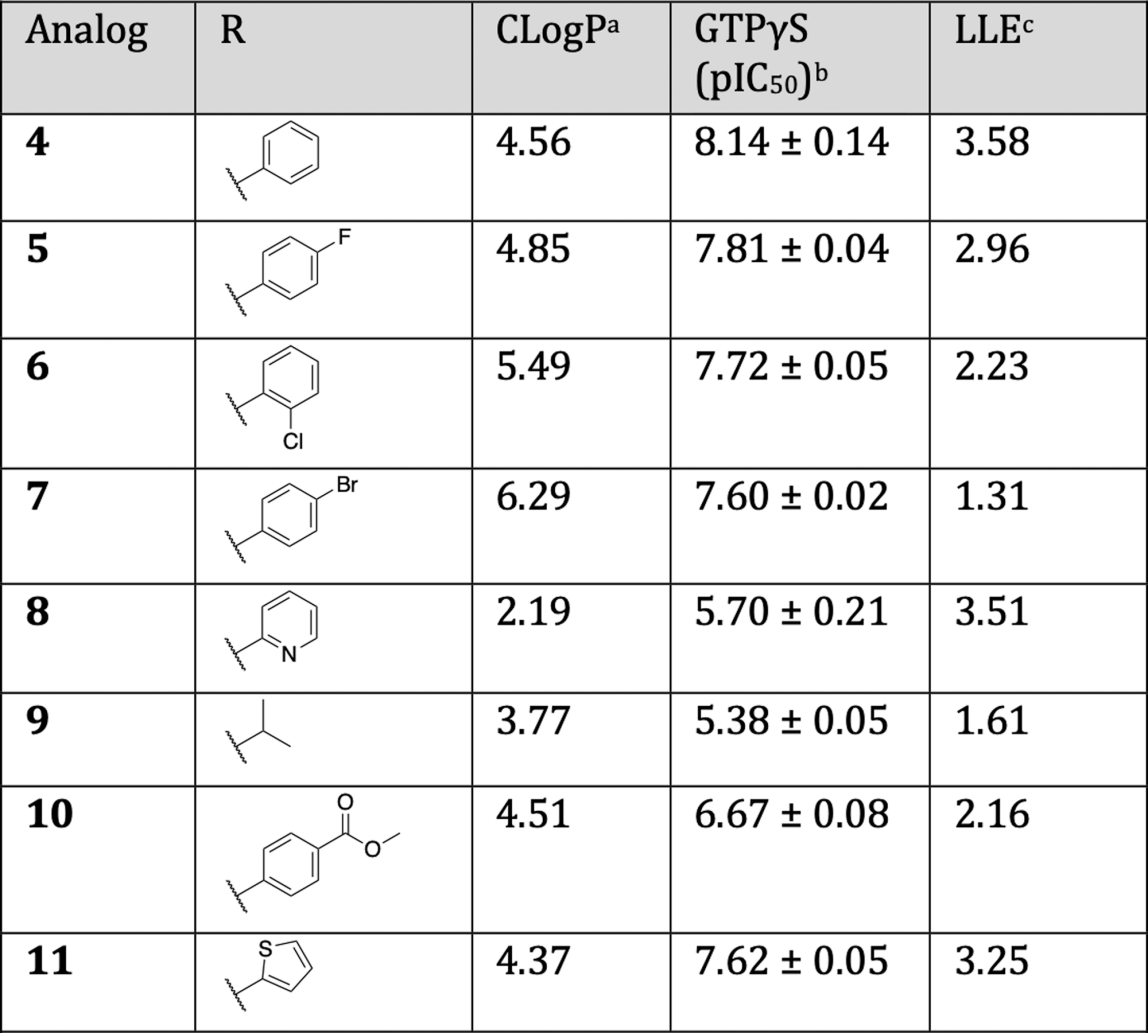
Physicochemical Properties and Activity Data of 3,5,6-Trisubstituted 1,2,4-Triazines **4**–**11**

a Logarithm of the partition coefficient for *n*-octanol/water (CLog *P*) was calculated using ChemDraw2019.

b Negative log of the IC_50_ in molar (pIC_50_); guanosine 5′-*O*-[gammathio]triphosphate ([^35^S]GTPγS) assay. Data are expressed as mean ± standard error of the mean (SEM).

c Ligand lipophilic efficiency (LLE) = pIC_50_ – CLog *P*; GLPG 1205(XII), pIC_50_ = 7.27 ± 0.04.

Attention then turned to the role of the indole ring for activity. Three analogues of **1** were produced in this series (Table 2). Analogue **12** incorporates an electronegative fluorine atom at the 5-position of the indole, which results in a decrease in activity. The indole NH is predicted to form a H-bond with the backbone carbonyl of Leu100^3.32^. This could explain why *N*-methyl analogue **13** is not active. These data further support our proposed binding hypothesis in which the indole is predicted to be bound in a deep narrow pocket.

**Table 2:**
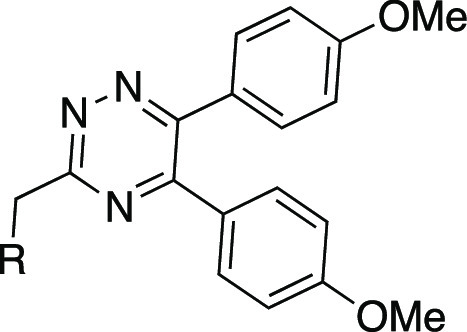

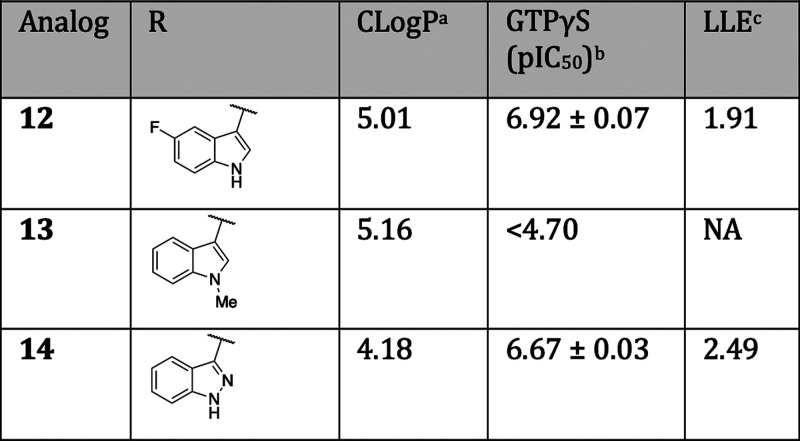
Physicochemical Properties and Activity Data of 3,5,6-Trisubstituted 1,2,4-Triazines **12**–**14**

a Logarithm of the partition coefficient for *n*-octanol/water (CLog *P*) was calculated using ChemDraw2019.

b Negative log of the IC_50_ in molar (pIC_50_); guanosine 5′-*O*-[gammathio]triphosphate ([^35^S]GTPγS) assay. Data are expressed as mean ± standard error of the mean (SEM).

c Ligand lipophilic efficiency (LLE) = pIC_50_ – CLog *P*; GLPG 1205(XII), pIC_50_ = 7.27 ± 0.04; NA, not applicable.

To further probe the steric and electronic requirements of the indole ring for binding, and taking into account the apparent limitations of anisole groups in both the 5- and 6-positions of the triazine ring, we switched to preparing analogues with phenyl groups in these positions (Table 3). Again, substitution in either the 5- or 6-positions of the indole ring was not tolerated (compounds **15** and **16**). It is of note that in these docked structures the diphenyl analogues move up the binding pocket to try and fit these substituents into the indole pocket.

**Table 3:**
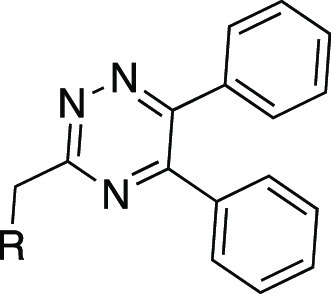

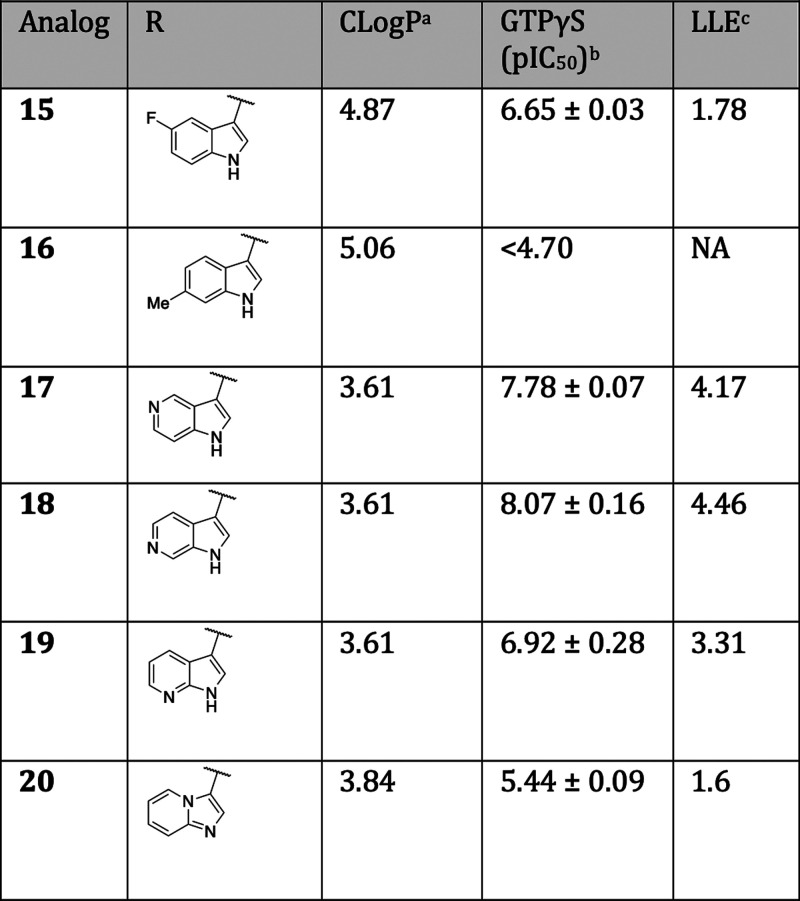
Physicochemical Properties and Activity Data of 3,5,6-Trisubstituted 1,2,4-Triazines **15**–**20**

a Logarithm of the partition coefficient for *n*-octanol/water (CLog *P*) was calculated using ChemDraw2019.

b Negative log of the IC_50_ in molar (pIC_50_); guanosine 5′-*O*-[gammathio]triphosphate ([^35^S]GTPγS) assay. Data are expressed as mean ± standard error of the mean (SEM).

c Ligand lipophilic efficiency (LLE) = pIC_50_ – CLog *P*; GLPG 1205(XII), pIC_50_ = 7.27 ± 0.04; NA, not applicable.

A series of azaindole analogues was prepared to investigate the effect of incorporating H-bond acceptor nitrogen heteroatoms into the indole ring and to reduce the lipophilicity of the molecules (Table 3). The three azaindole analogues **17**–**19** retain potency yet, again with a log unit less in calculated lipophilicity. Both the 5- and 7-azaindoles have less activity than indole analogue **4**. 6-Azaindole retains activity and has the most favorable LLE in the series. Imidazopyridine **20** incorporates a nitrogen atom connecting the two aromatic rings and as a result converts the indole NH into a H-bond acceptor. This analogue was the only molecule in this series to lose significant activity, supporting the proposed role of the indole H-bond donor for binding.

A series of molecules with a diverse set of substituents in the 3-position of the triazine ring was explored next (Table 4). However, none of these compounds were more active than indole analogue **4** or 6-azaindole analogue **18**.

**Table 4:**
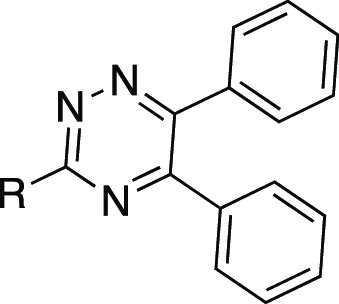

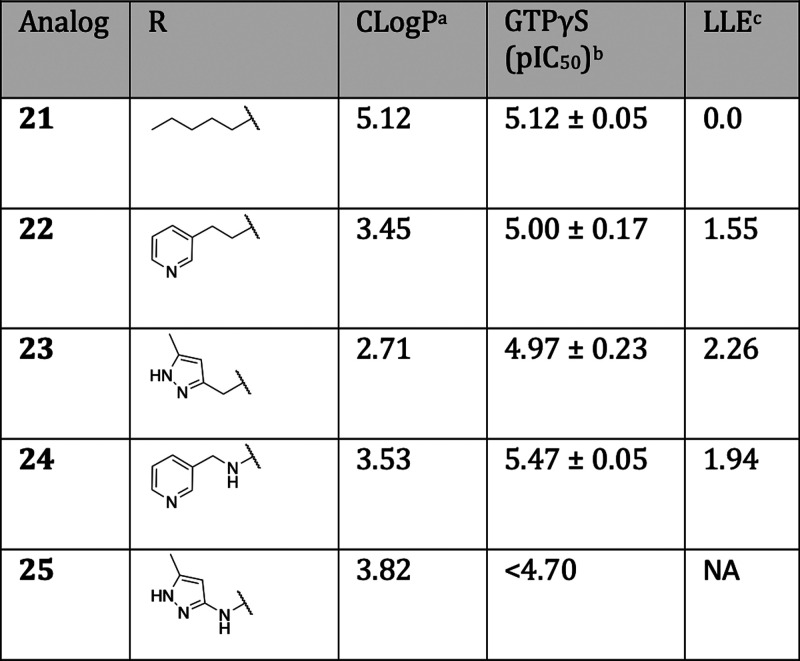
Physicochemical Properties and Activity Data of 3,5,6-Trisubstituted 1,2,4-Triazines **21**–**25**

a Logarithm of the partition coefficient for *n*-octanol/water (CLog *P*) was calculated using ChemDraw2019.

b Negative log of the IC_50_ in molar (pIC_50_); guanosine 5′-*O*-[gammathio]triphosphate ([^35^S]GTPγS) assay. Data are expressed as mean ± standard error of the mean (SEM).

c Ligand lipophilic efficiency (LLE) = pIC_50_ – CLog *P*; GLPG 1205(XII), pIC_50_ = 7.27 ± 0.04; NA, not applicable.

In our binding model, the 5- and 6-aryl substituents of the 1,2,4-triazine ring appear to bind in distinct pockets. A series of unsymmetrical 3-methylindole-5,6-trisubstituted 1,2,4-triazine analogues **26**–**58** (Table 5) and 3-(methyl-6-azaindole)-5,6-trisubstituted 1,2,4-triazine **59**–**70** (Table 6) were produced to interrogate the SAR of the 5- and 6-aryl substituents. The regioselective synthesis of these molecules is currently not possible, and so in each case, the two regioisomers were synthesized and separated using chiral SFC where necessary. The isomers were assigned based on retention time and in comparison with structurally assigned analogues **37** and **52** (vide supra) using X-ray crystallography. The antagonist activity of the analogues presented in Table 5 is entirely consistent with our proposed binding mode for this class of molecule. From compound docking, we see that 6-aryl substituents are predicted to have access to the extracellular opening providing a space to accommodate a larger substituent, whereas the pocket accommodating 5-aryl substitutions is sterically restricted. Of particular note are 4-benzyl acetate analogues **37** and **38**. Compound **37** (pIC_50_ 8.30 ± 0.35) is the most potent compound discovered during this SAR investigation.

**Table 5:**
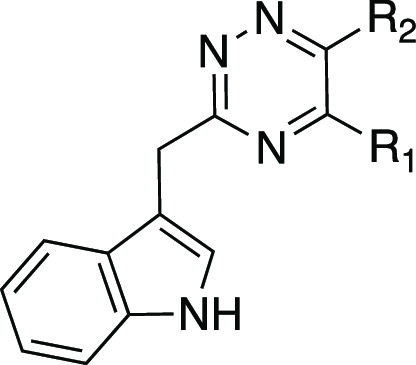

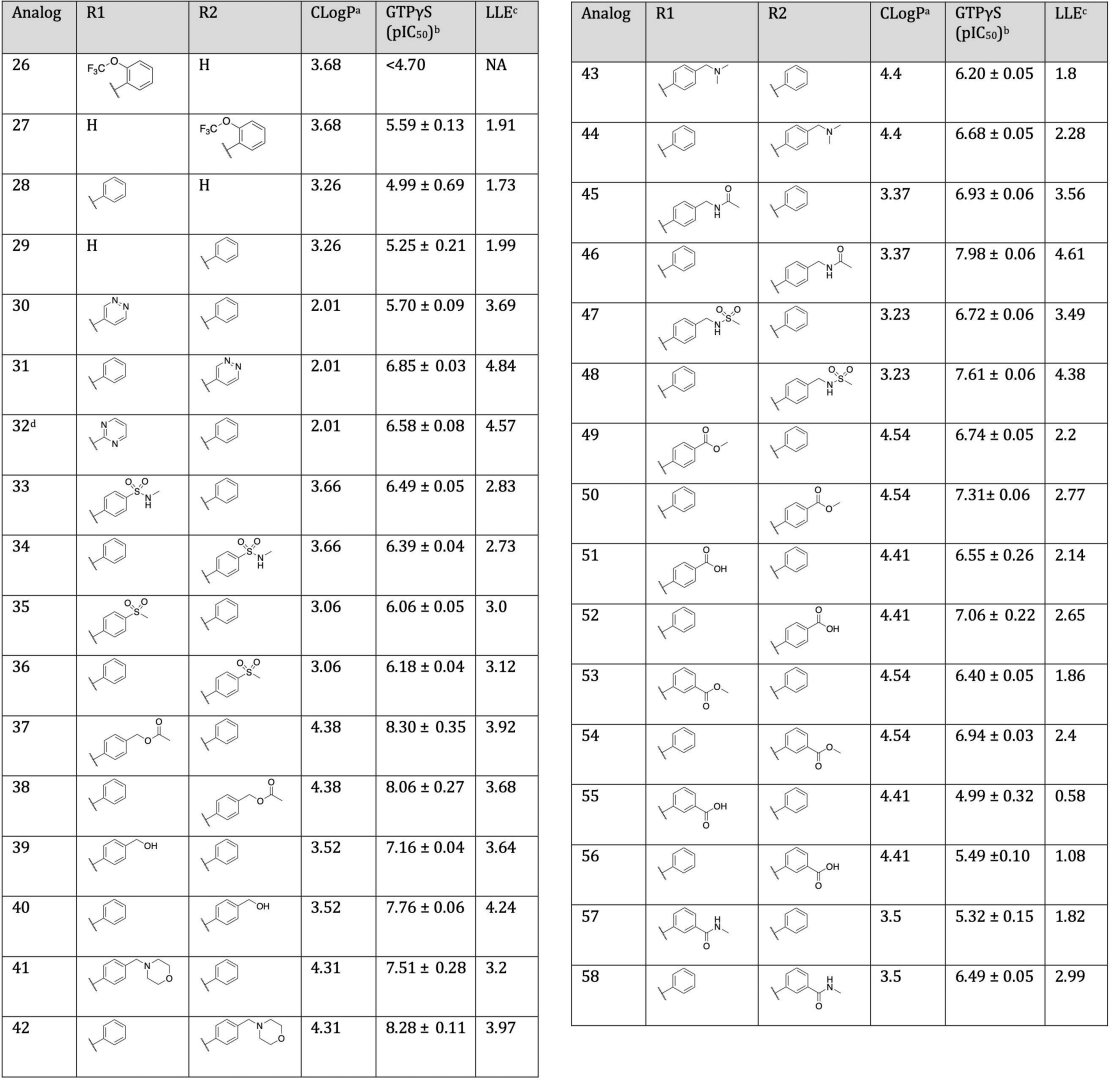
Physicochemical Properties and Activity Data of 3,5,6-Trisubstituted 1,2,4-Triazines **26**–**58**

a Logarithm of the partition coefficient for *n*-octanol/water (CLog *P*) was calculated using ChemDraw2019.

b Negative log of the IC_50_ in molar (pIC_50_); guanosine 5′-*O*-[gammathio]triphosphate ([^35^S]GTPγS) assay. Data are expressed as mean ± standard error of the mean (SEM).

c Ligand lipophilic efficiency (LLE) = pIC_50_ – CLog *P*.

d Only one isomer was isolated and regiochemistry could not be assigned; GLPG 1205(XII), pIC_50_ = 7.27 ± 0.04; NA, not applicable.

**Table 6:**
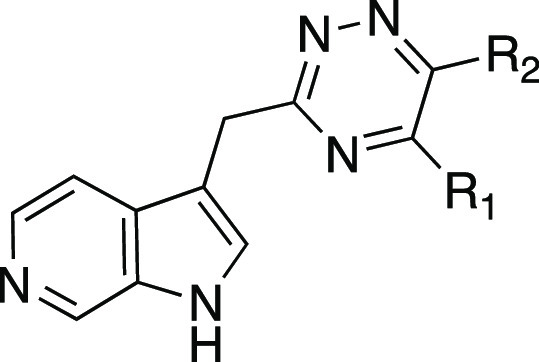

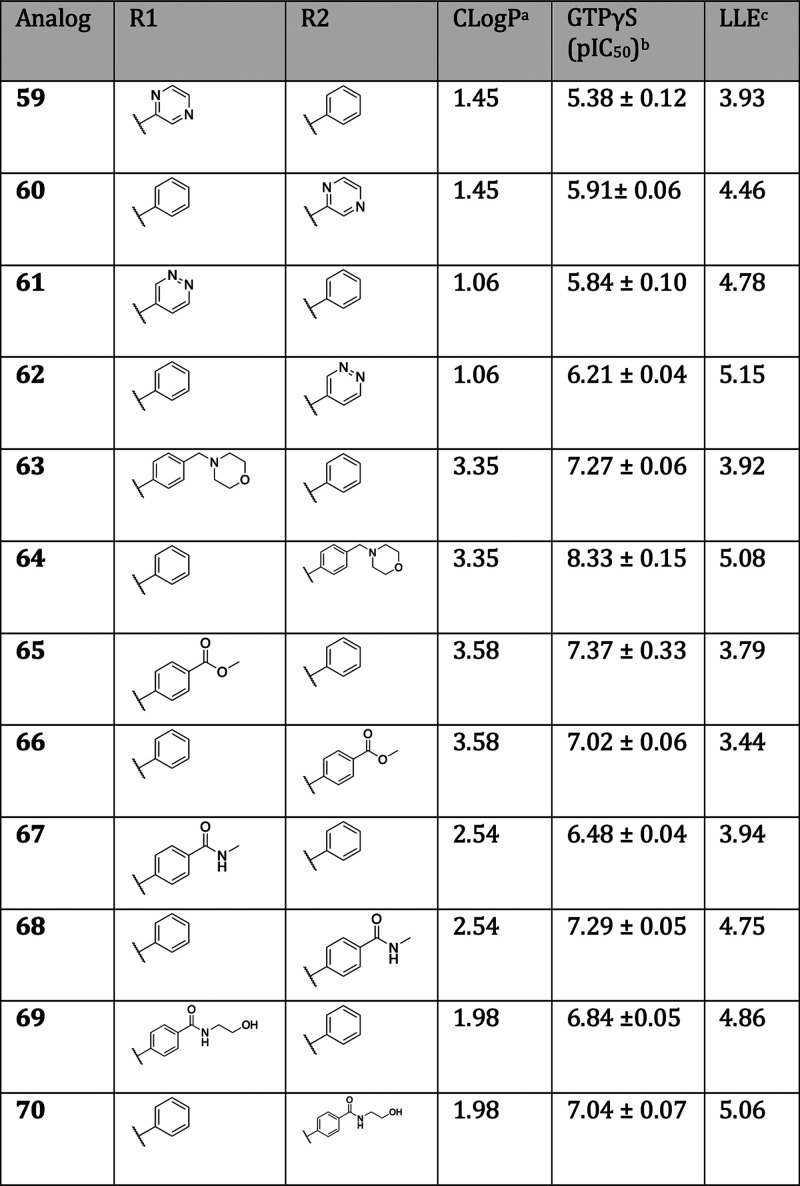
Physicochemical Properties and Activity Data of 3,5,6-Trisubstituted 1,2,4-Triazines **59**–**70**

a Logarithm of the partition coefficient for *n*-octanol/water (CLog *P*) was calculated using ChemDraw2019.

b Negative log of the IC_50_ in molar (pIC_50_); guanosine 5′-*O*-[gammathio]triphosphate ([^35^S]GTPγS) assay. Data are expressed as mean ± standard error of the mean (SEM).

c Ligand lipophilic efficiency (LLE) = pIC_50_ – CLog *P*; GLPG 1205(XII), pIC_50_ = 7.27 ± 0.04.

In considering pharmacokinetic (PK) parameters suitable to take compounds forward, we evaluated the CLog *P* and LLE of these molecules. The 6-azaindole analogues are consistently predicted to have better solubility, with CLog *P* range 1.06–3.58, compared to CLog *P* range 2.01–4.83 for the indole analogues.

5-Azaindole analogues **71** and **72** were prepared for direct comparison with the corresponding 6-azaindole analogues **67** and **68**. As expected the physicochemical properties are predicted to be the same; however, the biological activity is one log unit higher for the 6-azaindole analogue vs the 5-azaindole analogue (Table 7).

**Table 7:**
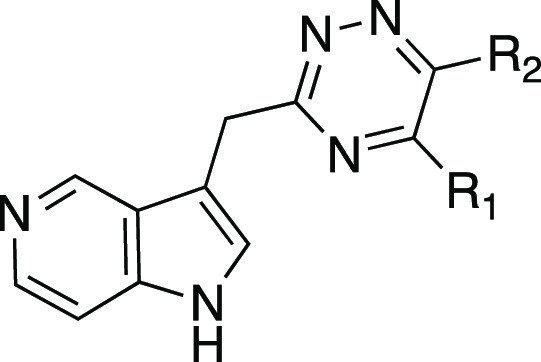

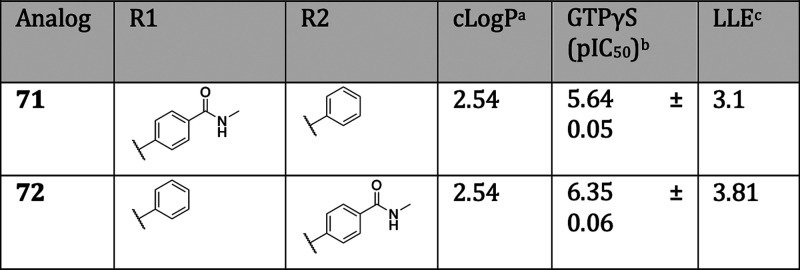
Physicochemical Properties and Activity Data of 3,5,6-Trisubstituted 1,2,4-Triazines **71** and **72**

a Logarithm of the partition coefficient for *n*-octanol/water (CLog *P*) was calculated using ChemDraw2019.

b Negative log of the IC_50_ in molar (pIC_50_); guanosine 5′-*O*-[gammathio]triphosphate ([^35^S]GTPγS) binding assay. Data are expressed as mean ± standard error of the mean (SEM).

c Ligand lipophilic efficiency (LLE) = pIC_50_ – CLog *P*; GLPG 1205(XII), pIC_50_ = 7.27 ± 0.04.

Next, we exploited the carboxylic acid of **51** and **52** to structurally diversify and produce a series of amides **73**–**90** (Table 8), each series with defined regiochemistry (Scheme 2). Again, this series of molecules was active in the [^35^S]GTPγS assay, with methyl amide **82** (pIC_50_ 8.01 ± 0.06) being most active.

**Table 8:**
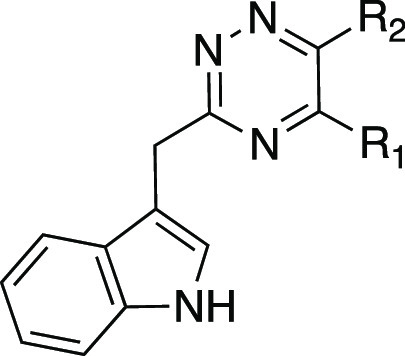

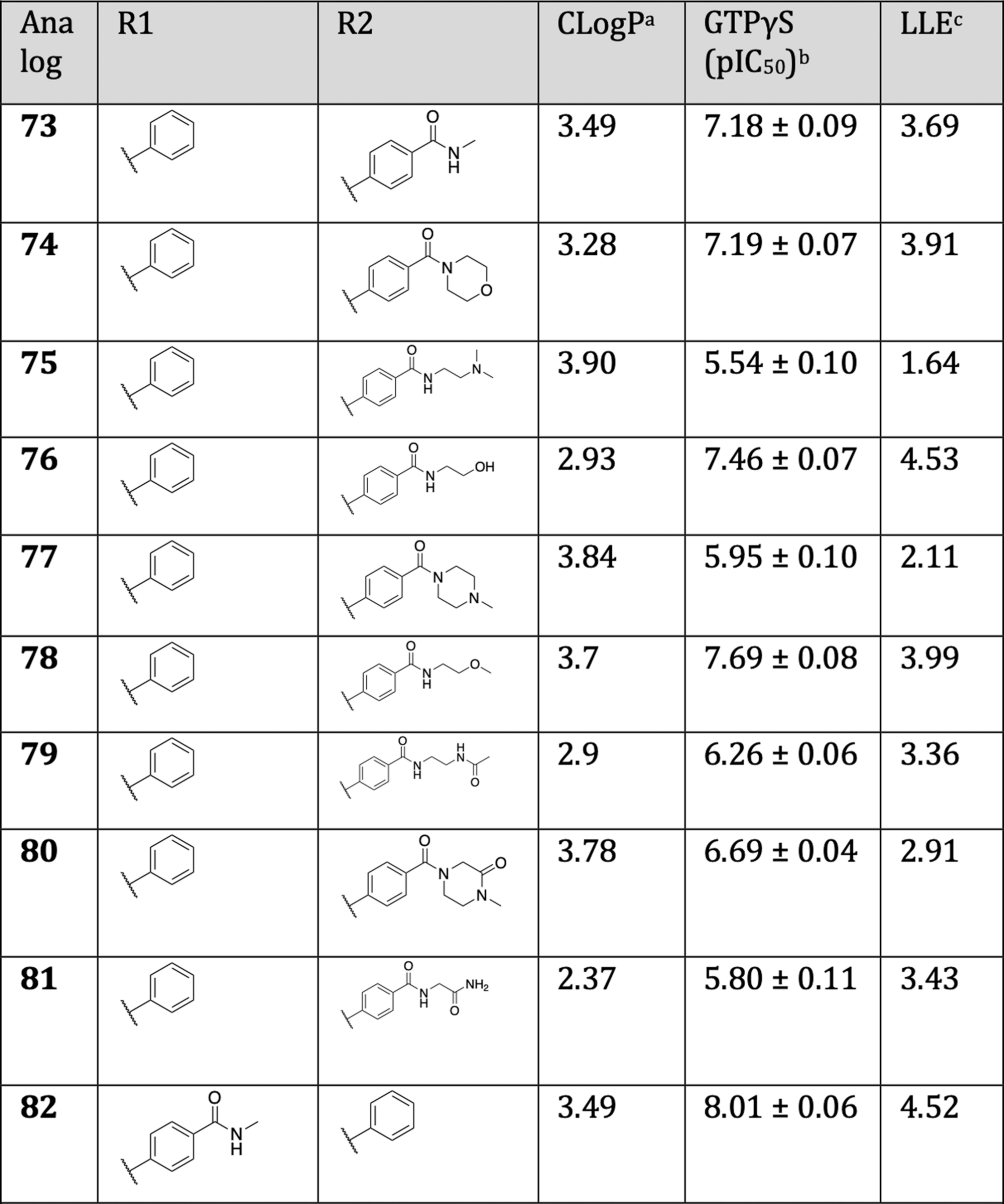
Physicochemical Properties and Activity Data of 3,5,6-Trisubstituted 1,2,4-Triazines **73**–**90**^c^

a Logarithm of the partition coefficient for *n*-octanol/water (CLog *P*) was calculated using ChemDraw2019.

b Negative log of the IC_50_ in molar (pIC_50_); guanosine 5′-*O*-[gammathio]triphosphate ([^35^S]GTPγS) assay. Data are expressed as mean ± standard error of the mean (SEM).

c Ligand lipophilic efficiency (LLE) = pIC_50_ – CLog *P*; GLPG 1205(XII), pIC_50_ = 7.27 ± 0.04.

### 2.4 Selectivity

**Figure 7:**
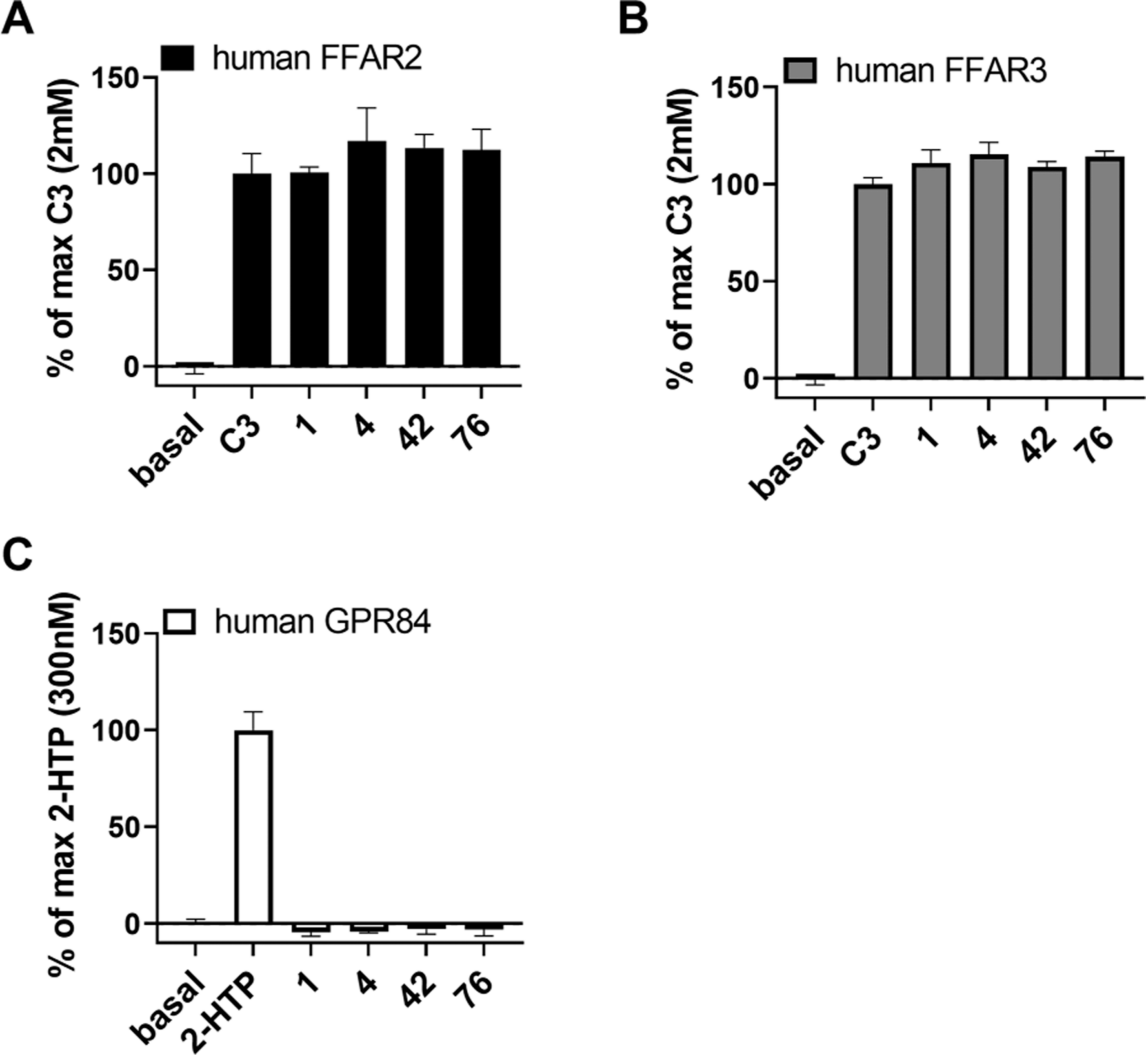
[^35^S]GTPγS binding was performed on membrane preparations from Flp-In T-REx 293 cells induced to express human FFAR2 (A), human FFAR3 (B), or a human GPR84-Gα_i2_ fusion protein (C). Compounds **1**, **4**, **42**, and **76** (at 10 μM) did not block effects of C3 at either FFAR2 or FFAR3.

We have previously shown that compound **4** shows high selectivity for GPR84 against a panel of 167 other human GPCRs.^23^ However, although the assessed GPCR set did include each of the long-chain fatty acid receptors FFAR1 (aka GPR40) and FFAR4 (aka GPR120), it did not include either FFAR2 or FFAR3 that respond instead to short-chain fatty acids. Compounds **1**, **4**, **42**, and **76** displayed no ability to block the actions of the C3 fatty acid propionate at either FFAR2 or FFAR3 when tested at 10 μM (Figure 7). We have also shown that compound **1** and compound **4** are unable to antagonize mouse GPR84 and provided a molecular basis for this lack of activity via comparison of homology models of human and mouse GPR84 linked to mutagenesis studies.^23^ To extend this, we tested the antagonist activity of all of the compounds reported herein at mouse GPR84. At 10 μM, very few of the compounds displayed any inhibitory activity (SI Figure 1). Compounds that did produce greater than 50% inhibition at 10 μM were assessed further in concentration–response curves. None produced pIC_50_ within 1000-fold of its activity at human GPR84.

### 2.5 Drug Metabolism and Pharmacokinetics (DMPK)

A series of analogues were selected based on activity and predicted physicochemical properties to be profiled in some preliminary in vitro DMPK assays (Table 9). Highly potent analogue **4** is rapidly metabolized in both human liver microsomes and mouse hepatocytes, is highly protein bound, and has poor kinetic solubility. Its azaindole analogue (compound **18**) has improved LLE, resulting from lower Log *D*_7.4_, improved kinetic solubility, and a modest improvement in metabolic stability. Similar improvements in LLE and stability were achieved through substitution of the 6-phenyl ring with the more polar 4-benzylmorpholine group (**42**) or 2-(hydroxyethyl)benzamide (**76**). Compound **68** incorporates both of these substitutions, namely, replacing the indole with a 6-azaindole and also the 6-phenyl ring with more polar functionality. As expected the combination of these two groups resulted in lower Log *D*_7.4_ and plasma protein binding (PPB) with a significant increase in kinetic solubility and a modest improvement in metabolic stability in comparison to compound **4**.

**Table 9:** Experimental Physicochemical and In Vitro ADME Properties of Selected Compounds^a^^c^

analog	GTPγS (pIC_50_)	Log *D*_7.4_	LLE	kinetic solubility (μM)	mouse heps *t*_1/2_ (min)	human mics *t*_1/2_ (min)	mouse PPB (%free)
4	8.14 ± 0.14	4.24	3.9	1.4	4.4	21.8	<1
18	8.07 ± 0.16	3.49	4.58	71	9.7	66.2	1.9
42	8.28 ± 0.11	3.84	4.44	14	16	12.8	<1
68	7.29 ± 0.05	2.71	4.58	146	12.5	39.9	3.8
76	7.46 ± 0.07	3.09	4.37	61	47.3	57.2	1.1

a Guanosine 5′-*O*-[gammathio]triphosphate ([^35^S]GTPγS) assay, data are expressed as mean ± standard error of the mean (SEM); Log *D*_7.4_ (distribution coefficient).

c Ligand lipophilic efficiency (LLE) = pIC_50_ – Log *D*_7.4_; mouse hepatocyte (mouse heps); human microsomes (human mics); mouse plasma protein binding (mouse PPB).

Based on these data, compounds **42** and **76** were selected for in vivo PK profiling (Table 10). Compound **42** has a good elimination half-life (2.51 h), moderate rate of clearance, and bioavailability. Analogue **76** has a slower rate of clearance, moderate bioavailability, and shorter elimination half-life (1.22 h).

**Table 10:** Experimental in Vivo Mouse PK Properties of Selected Compounds^a^

	IV (1 mg/kg)					PO (10 mg/kg)			
analog	half-life (h)	CL (mL/min/kg)	*V*_ss_ (L/kg)	*C*_0_ (ng/mL)	AUCall (ng/mL·h)	*C*_max_ (ng/mL)	*T*_max_ (h)	AUCall (ng/mL·h)	bioavailability (%)
42	2.51 (6.55)	38.9 (13.9)	7.24 (9.2)	394 (19.1)	420 (12.5)	402 (31.8)	1	1590 (18.7)	36.8 (18.7)
76	1.22 (13.5)	22.0 (11.3)	1.81 (15.2)	1170 (29.6)	757 (10.8)	1080 (16.1)	1	3160 (8.82)	41.5 (8.73)

a Intravenous (IV); per os (PO), percentage of relative standard deviation (CV %) provided in parenthesis, calculated using formula CV % = SD/*M* × 100 (CL = clearance level); volume of distribution at steady state (*V*_ss_); area under curve (AUCall); peak drug concentration (*C*_max_); peak time (*T*_max_).

## 3 Conclusions

The development of small-molecule antagonists that target GPR84 represents a promising potential therapeutic strategy for the treatment of inflammatory diseases. High-quality small-molecule antagonists and methods to interrogate GPR84 structure and function are invaluable to realize the potential of GPR84 as a therapeutic target. Here, we present a potential binding mode and substantial SAR study of GPR84 antagonist **1** that was previously discovered by us in an HTS. Molecular docking revealed that antagonist **1** sits within the helical bundle forming several direct contacts with the GPR84 receptor helices and ECL2. This binding mode was used to rationalize the SAR of analogues. The structural requirements of the indole ring, and 5- and 6-substituents of the triazine core scaffold of antagonist **1** were investigated. A series of four potent analogues were progressed to in vitro DMPK assays. Compounds **42** and **76** were subsequently progressed to in vivo PK profiling. Of these compounds, compound **42** displays potent antagonist activity and a favorable PK profile. These data provide an understanding of antagonist mode of binding and lead compound **42** is a promising novel GPR84 antagonist for further drug development.

Antagonist **4** was shown to be highly selective for GPR84 when assessed against a panel of 167 other GPCRs^23^ and although we demonstrate that compound **42** lacks activity at other members of the free fatty acid group of GPCRs whether this compound will be equally selective across the broader family of GPCRs remains to be assessed.

## 4 Experimental Section

### 4.1 General Information

Chemicals and solvents were purchased from commonly used suppliers and used without further purification. Celite was purchased from Acros Organics or Fisher. Hydrophobic frits and SCX columns were purchased from Telos or Biotage. Telos columns were purchased from Telos. Daicel OJ-H columns and Daicel AD-H columns were purchased from Daicel. Reacti-Vials were purchased from Thermo Fisher. Chromatographic purifications were performed using prepacked Telos silica columns using a Biotage Isolera Purification system (Uppsala, Sweden). Deuterated solvent was obtained from Cambridge Isotopes. All NMR spectra were recorded using a Bruker Avance 400 MHz spectrometer. Chemical shifts (δ) are given in ppm relative to the solvent peak, and coupling constants (*J*) are reported in hertz. Reactions under microwave radiation were performed on Biotage Initiator Eight or Sixty under standard operating conditions. The purity of all final compounds is >95%, as determined by liquid chromatography–mass spectrometry (LC–MS).

#### 4.1.1 Method A: General Method for the Synthesis of 1,2-Diketones

Synthesis of α-hydroxy ketones required for the synthesis of 1,2-diketones was performed according to published literature methods.^36,37^ The desired ethanone (1 equiv) dissolved in dimethyl sulfoxide (DMSO) (3.00 mL) was stirred at room temperature, and *N*-bromosuccinimide (NBS) (1 equiv) was added in one portion. The resultant solution was stirred at room temperature overnight. Worked up by partitioning mixture between water (30 mL) and ethyl acetate (3 × 10 mL). Combined extracts washed with water, brine, dried over Na_2_SO_4_, filtered, and concentrated to dryness under reduced pressure to afford symmetrical 1,2-diketones. Used without further purification.

#### 4.1.2 Method B: General Method for the Synthesis of Methyl Esters

To a solution of the desired acid (1 equiv) in methanol (5 mL) was added H_2_SO_4_ (95.0%, 3 equiv), and the mixture was microwaved at 100 °C for 10 min, worked up by a basifying mixture by the addition of sat. NaHCO_3_, and concentrated under reduced pressure to remove methanol. The residue was partitioned between water and ethyl acetate (2 × 10 mL), and the combined organics were washed with brine, dried over Na_2_SO_4_, filtered, and concentrated to dryness under reduced pressure to afford the corresponding methyl esters.

#### 4.1.3 Method C: General Method for the Synthesis of Hydrazides

A solution of methyl ester (1 equiv) was transferred to a 5 mL microwave vial and dissolved in ethanol (2 mL). Hydrazine hydrate (2 equiv) was added in one portion, the vial was capped, and the mixture was heated at 90 °C for 5.5 h. The mixture was cooled to room temperature, further hydrazine hydrate (2 equiv) was added, the mixture was heated at 90 °C overnight, and worked up by concentrating reaction mixture to dryness under reduced pressure to afford the subsequent hydrazides.

#### 4.1.4 Method D: General Method for the Synthesis of 3,5,6-Trisubstituted 1,2,4-Triazines with Symmetrical 1,2-Diketones

A solution of desired acetohydrazide (1.0 equiv), symmetrical 1,2-diketones (1.0 equiv), and ammonium acetate (10.0 equiv) was transferred to a 5 mL microwave vial. Acetic acid (2.5 mL) was added, and the resultant suspension was subjected to microwave radiation at 180 °C for 5 min. The reaction mixture was concentrated to dryness under reduced pressure. Residue was partitioned between water (20 mL) and dichloromethane (DCM) (2 × 10 mL). Combined extracts were washed with brine, dried over Na_2_SO_4_, filtered, and concentrated to dryness under reduced pressure. The residue was purified by flash, preparative HPLC or SFC as indicated.

#### 4.1.5 Method E: General Method for the Synthesis of 3,5,6-Trisubstituted 1,2,4-Triazines with Unsymmetrical 1,2-Diketones

To a microwave vial were added acetohydrazide (1.0 equiv), unsymmetrical 1,2-diketones (1.0 equiv), and acetic acid (2 mL). Then, the vial was sealed and subjected to microwave radiation for 5 min at 180 °C. The reaction mixture was cooled, ammonium acetate (10 equiv) was added, and the vial was resealed before subjecting it again to microwave radiation for 5 min at 180 °C. The mixture was concentrated, and the resulting residues were purified on preparative HPLC (basic conditions). The desired fractions were concentrated to afford a solid or oil. The mixture of regioisomers was separated by SFC using a Daicel AD-H column (10 × 250 mm^2^, 20% methanol, 15 mL/min).

#### 4.1.6 Method F: General Method for the Synthesis of 3,5,6-Trisubstituted 1,2,4-Triazines with Unsymmetrical 1,2-Diketones

To a microwave vial were added acetohydrazide (1.0 equiv), unsymmetrical 1,2-diketones (1.0 equiv), and acetic acid (2 mL). Then, the vial was sealed and subjected to microwave radiation for 5 min at 180 °C. The reaction mixture was cooled, ammonium acetate (10 equiv) was added, and the vial was resealed before subjecting it again to microwave radiation for 5 min at 180 °C. The reaction mixture was concentrated and passed through an SCX cartridge eluting the product with 2 M NH_3_/methanol. The resulting residue was purified by preparative HPLC (basic conditions) to give a mixture of regioisomers. The regioisomers were separated by SFC using a Daicel OJ-H (10 × 250 mm^2^, 30% methanol, 15 mL/min).

#### 4.1.7 Method G: General Method for the Synthesis of Amide Bond Formation

To a solution of desired acid (1 equiv) in DCM (1 mL) was added propylphosphonic anhydride (50% in ethyl acetate,1.5 equiv), and the resultant solution was stirred at room temperature for 10 min. A solution of desired amine (1.2 equiv) and *N*,*N*-diisopropylethylamine (3 equiv) in DCM (0.5 mL) was added, and the resultant orange solution was stirred at room for 3 h. The crude mix was partitioned between water/brine (20 mL) and DCM (3 × 10 mL), and the organic layers were separated using a hydrophobic frit, combined, and concentrated to dryness under reduced pressure. The resulting residues were purified by flash chromatography using 0–5% methanol in DCM. The relevant fractions were combined and concentrated to dryness.

#### 4.1.8 Method H: General Method for Boc Protection of Amine

To a solution of desired amine (1 equiv) in DCM (5.00 mL) were added di-*tert*-butyl dicarbonate (1.5 equiv) and triethylamine (3 equiv), and the mixture was gently heated to 40 °C for 4 h. The mixture was further diluted in DCM and washed with brine. The organic layers were passed through a phase separator and concentrated to dryness.

#### 4.1.9 Preparative HPLC Method

Preparative HPLC was carried out on a Waters HPLC comprising a Waters 2767 Sample Manager, a Waters 2545 Binary Gradient Module, a Waters Systems Fluidics Organiser, a Waters 515 ACD pump, a Waters 2998 Photodiode Array Detector, and a Waters XBridge Prep OBD C18, 5 μm, 19 mm × 50 mm i.d. column and a flow rate of 20 mL/min. The general method that may be used to purify compounds are: acidic reversed-phase HPLC (water/acetonitrile/0.1% trifluoroacetic acid (TFA)) using a standard gradient of 5% acetonitrile/95% water to 100% acetonitrile or basic reversed-phase HPLC (water/acetonitrile/0.01 M ammonia solution) using a standard gradient of 10% acetonitrile/90% water to 100% acetonitrile. UV detection, e.g., 254 nM, is used for the collection of fractions from HPLC.

#### 4.1.10 Supercritical Fluid Chromatography (SFC)

SFC was carried out on a Waters Investigator SFC comprising a Waters 05962 fluid delivery module, a Waters 07419 autosampler, a Waters 2489 UV/vis detector, a Waters 08005 column oven, a Waters 279002192 heat exchanger, a Waters ABPR-20A back-pressure regulator, and a Waters 08127 fraction collection module. The general method used liquid CO_2_ and the appropriate modifier as stated in the experimental as an isocratic mix unless otherwise stated. The preparative column used was as stated in the experimental. UV detection, e.g., 254 nM, was used for the collection of fractions from SFC.

#### 4.1.11 Analytical Methods for the Pairs of Separate Isomers

##### 4.1.11.1 Method A

Phenomenex Luna C18 (2)-HST column (2.5 μm, 50 × 2.0 mm^2^). Mobile phase A contained 0.1% formic acid in 18 MΩ water, and mobile phase B contained 0.1% formic acid in HPLC-grade acetonitrile. A flow rate of 1.00 mL/min was used over a 5.5 min gradient starting with 99% mobile phase A gradually increasing to 100% mobile phase B. The samples were monitored at 254 nm.

##### 4.1.11.2 Method B

Phenomenex Luna C18 (2)-HST column (2.5 μm, 50 × 2.0 mm^2^). Mobile phase A contained 0.1% formic acid in 18 MΩ water, and mobile phase B contained 0.1% formic acid in HPLC-grade acetonitrile. A flow rate of 1.00 mL/min was used over a 5.5 min gradient starting with 90% mobile phase A gradually increasing to 100% mobile phase B. The samples were monitored at 254 nm.

##### 4.1.11.3 Method C

Phenomenex Kinetix EVO C18 (2.6 μm, 50 × 2.1 mm^2^) column. Mobile phase A contained 0.1% formic acid in 18 MΩ water, and mobile phase B contained 0.1% formic acid in HPLC-grade acetonitrile. A flow rate of 1.00 mL/min was used over a 5.5 min gradient starting with 90% mobile phase A gradually increasing to 100% mobile phase B. The samples were monitored at 254 nm.

#### 4.1.12 LCMS

Low-resolution mass spectrometry data (*m*/*z*) were obtained from an Agilent 6140 series quadrupole mass spectrometer with a multimode source attached to an Agilent 1200 series HPLC.

### 4.2 Experimental Procedures and Characterization Data

#### 4.2.1 3-((5,6-Bis(4-methoxyphenyl)-1,2,4-triazin-3-yl)methyl)-1*H*-indole (**1**)

Prepared according to method D. The residue was purified by flash chromatography eluting with 0–2.5% methanol in DCM and then re-purified by flash chromatography using 0–100% ethyl acetate in heptane to give a pale orange solid. The solid was sonicated with ether (10 mL), filtered off, washed with ether, and dried under suction to give the title compound **1** (47 mg, 19%). ^1^H NMR (400 MHz, CDCl_3_) δ 8.07–8.21 (br s, 1 H), 7.90–8.01 (m, 1 H), 7.55–7.63 (m, 2 H), 7.47–7.54 (m, 2 H), 7.33–7.42 (m, 2 H), 7.12–7.26 (m, 2 H), 6.79–6.95 (m, 4 H), 4.70 (s, 2 H), 3.85 (s, 3 H), 3.84 (s, 3 H); *m*/*z*: calcd for C_26_H_23_N_4_O_2_ [M + H]^+^, 423.0; found, 423.0.

#### 4.2.2 3-((5,6-Diphenyl-1,2,4-triazin-3-yl)methyl)-1*H*-indole (**4**)

Prepared according to method D. The residue was purified by flash chromatography eluting with 0–30% ethyl acetate in heptane to give a pale orange solid. The solid was sonicated with ether (2 mL), solid filtered off, washed with ether, and dried under suction to give the title compound **4** (80 mg, 38%). ^1^H NMR (400 MHz, CDCl_3_) δ 8.16 (br s, 1H), 7.96 (m, 1H), 7.54 (m, 4H), 7.30–7.47 (m, 8H), 7.20 (m, 2H), 4.73 (s, 2H); *m*/*z*: calcd for C_24_H_19_N_4_ [M + H]^+^, 363.4; found, 363.4.

#### 4.2.3 3-((5,6-Bis(4-fluorophenyl)-1,2,4-triazin-3-yl)methyl)-1*H*-indole (**5**)

Prepared according to method D. The residue was purified by flash chromatography eluting with 0–30% ethyl acetate in heptane to give a pale orange solid. The solid was sonicated with ether (2 mL), filtered off, washed with ether, and dried under suction to give the title compound **5** (65 mg, 28%). ^1^H NMR (400 MHz, CDCl_3_) δ 8.08–8.21 (m, 1H), 7.89–7.98 (m, 1H), 7.47–7.61 (m, 4H), 7.32–7.41 (m, 2H), 7.14–7.26 (m, 2H), 7.00–7.12 (m, 4H), 4.72 (s, 2H); *m*/*z*: calcd for C_24_H_17_F_2_N_4_ [M + H]^+^, 399.1; found, 399.2.

#### 4.2.4 3-((5,6-Bis(2-chlorophenyl)-1,2,4-triazin-3-yl)methyl)-1*H*-indole (**6**)

Prepared according to method D. The residue was purified by flash chromatography eluting with 0–50% ethyl acetate in heptane. The resulting solid was further purified by preparative HPLC (basic conditions) to give the title compound **6** (8 mg, 3%). ^1^H NMR (400 MHz, CDCl_3_) δ 8.05–8.27 (br s, 1H), 7.80–7.93 (m, 1H), 7.08–7.48 (m, 12H), 4.78 (s, 2H); *m*/*z*: calcd for C_24_H_17_Cl_2_N_4_ [M + H]^+^, 431.1; found, 431.0.

#### 4.2.5 3-((5,6-Bis(4-bromophenyl)-1,2,4-triazin-3-yl)methyl)-1*H*-indole (**7**)

Prepared according to method D. The residue was purified by flash chromatography eluting with 0–40% ethyl acetate in heptane. The resulting solid was further purified by sonicating with ether (2 mL), and the resultant yellow solid was filtered off, washed with further ether (5 mL), and dried under suction to give the title compound **7** (192 mg, 32%). ^1^H NMR (400 MHz, CDCl_3_) δ 8.06–8.15 (br s, 1H), 7.89–7.96 (m, 1H), 7.51 (m, 4H), 7.35–7.45 (m, 5H), 7.31–7.35 (m, 1H), 7.20–7.26 (m, 1H), 7.13–7.19 (m, 1H), 4.71 (s, 2H); *m*/*z*: calcd for C_24_H_17_Br_2_N_4_ [M + H]^+^, 519.0; found, 520.8.

#### 4.2.6 3-((5,6-Di(pyridin-2-yl)-1,2,4-triazin-3-yl)methyl)-1*H*-indole (**8**)

Prepared according to method D. The resulting residue was purified by flash chromatography eluting with 50–80% ethyl acetate in heptane to give the title compound **8** (18 mg, 8%). ^1^H NMR (400 MHz, CDCl_3_) δ 8.20–8.39 (m, 3H), 8.09–8.19 (m, 1H), 7.97–8.08 (m, 1H), 7.73–7.95 (m, 3H), 7.32–7.38 (m, 1H), 7.28 (m, 5H), 4.76 (s, 2H); *m*/*z*: calcd for C_22_H_17_N_6_ [M + H]^+^, 365.1; found, 365.0.

#### 4.2.7 3-((5,6-Diisopropyl-1,2,4-triazin-3-yl)methyl)-1*H*-indole (**9**)

Prepared according to method D. Residue purified by flash chromatography eluting with 20–80% ethyl acetate in heptane. The resulting residues were further purified by preparative HPLC (basic conditions) to afford the title compound **9** as a pale yellow gum. (76 mg, 16%). ^1^H NMR (400 MHz, CDCl_3_) δ 8.00–8.18 (br s, 1H), 7.83–7.97 (m, 1H), 7.30–7.43 (m, 1 H), 7.25–7.28 (m, 1H), 7.10–7.24 (m, 2H), 4.53 (s, 2H), 3.33 (m, 1H), 3.24 (m, 1H), 1.38 (m, 6H), 1.29 (m, 6H); *m*/*z*: calcd for C_18_H_23_N_4_ [M + H]^+^, 295.2; found, 295.2.

#### 4.2.8 Methyl 4-[3-(1*H*-Indol-3-ylmethyl)-6-(4-methoxycarbonylphenyl)-1,2,4-triazin-5-yl]benzoate (**10**)

Prepared according to method D. The resulting residue was purified by flash chromatography eluting with a gradient 0–50% ethyl acetate in heptane. The resulting residues were further purified by preparative HPLC (basic conditions) to afford the title compound **10** (73 mg, 5%). ^1^H NMR (400 MHz, CDCl_3_) δ 8.08–8.16 (br s, 1H), 7.89–8.08 (m, 5H), 7.58 (dd, *J* = 8.5, 2.8 Hz, 4H), 7.32–7.43 (m, 2H), 7.13–7.27 (m, 2H), 4.75 (s, 2H), 3.95 (s, 6H); *m*/*z*: calcd for C_28_H_23_N_4_O_4_ [M + H]^+^, 479.2; found 479.0.

#### 4.2.9 3-((5,6-Di(thiophen-2-yl)-1,2,4-triazin-3-yl)methyl)-1*H-*indole (**11**)

Prepared according to method D. The resulting residue was purified by flash chromatography eluting with a gradient 0–5% methanol in DCM. The appropriate fractions were combined, and the was solvent removed at reduced pressure to afford the title compound **11** (30 mg, 15%). ^1^H NMR (400 MHz, DMSO-*d*_6_) δ 10.88–11.04 (m, 1H), 7.82–7.96 (m, 2H), 7.68–7.76 (m, 1H), 7.41–7.47 (m, 1H), 7.28–7.38 (m, 3H), 7.21 (dd, *J* = 3.76, 5.02 Hz, 1H), 6.93–7.16 (m, 3H), 4.49 (s, 2H); *m*/*z*: calcd for C_20_H_15_N_4_S_2_ [M + H]^+^, 375.1; found, 375.0.

#### 4.2.10 2-(5-Fluoro-1*H*-indol-3-yl)acetohydrazide (**12a**)

Prepared according to method C. Worked up by concentrating reaction mixture to dryness under reduced pressure to give the title compound **12a** (108 mg, 60%). ^1^H NMR (400 MHz, CDCl_3_) δ 7.21–7.34 (m, 3H), 6.88 (td, *J* = 9.1, 2.6 Hz, 1H), 3.58 (d, *J* = 2.5 Hz, 2H).

#### 4.2.11 3-((5,6-Bis(4-methoxyphenyl)-1,2,4-triazin-3-yl)methyl)-5- fluoro-1*H*-indole (**12**)

Prepared according to method D. The resulting residue was purified by flash chromatography with 0–50% ethyl acetate in heptane. The resulting beige solid was sonicated with ether (5 mL), filtered off, washed with ether, and dried to afford the title compound **12** (37 mg, 15%). ^1^H NMR (400 MHz, CDCl_3_) δ 8.16 (br s, 1H), 7.47–7.66 (m, 5H), 7.35–7.42 (m, 1H), 7.28 (m, 1H), 6.82–7.01 (m, 5H), 4.62 (s, 2H), 3.86 (s, 3H), 3.85 (s, 3H); *m*/*z*: calcd for C_26_H_22_FN_4_O_2_ [M + H]^+^, 441.2; found, 441.0.

#### 4.2.12 Methyl 2-(1-Methyl-1*H*-indol-3-yl)acetate (**13a**)

Prepared according to method B. ^1^H NMR (400 MHz, CDCl_3_) δ 7.65 (dt, *J* = 7.9, 1.0 Hz, 1H), 7.34 (dt, *J* = 8.2, 1.0 Hz, 1H), 7.28 (ddd, *J* = 8.3, 6.9, 1.2 Hz, 1H), 7.18 (ddd, *J* = 8.0, 6.9, 1.2 Hz, 1H), 7.08 (d, *J* = 0.9 Hz, 1H), 3.82 (d, *J* = 0.9 Hz, 2H), 3.79 (s, 3H), 3.75 (s, 3H).

#### 4.2.13 2-(1-Methyl-1*H*-indol-3-yl)acetohydrazide (**13b**)

Prepared according to method C. Worked up by concentrating reaction mixture to dryness under reduced pressure to afford the title compound **13b**, (98.4 mg, 65%). ^1^H NMR (400 MHz, CD_3_OD) δ 7.51–7.57 (m, 1H), 7.27–7.33 (m, 1H), 7.12–7.19, (m, 1H), 6.99–7.09 (m, 2H), 4.85 (s, 3H), 3.74 (s, 3H), 3.59 (s, 2H).

#### 4.2.14 3-((5,6-Bis(4-methoxyphenyl)-1,2,4-triazin-3-yl)methyl)-1-methyl-1*H*-indole (**13**)

Prepared according to method D. Residue was purified flash chromatography on silica eluting with 0–50% ethyl acetate in heptane to afford the title compound **13** (15 mg, 13%). ^1^H NMR (400 MHz, CDCl_3_) δ 7.94 (m, 1H), 7.47–7.61 (m, 4H), 7.21–7.36 (m, 2H), 7.12–7.21 (m, 2H), 6.82–6.96 (m, 4H), 4.66 (s, 2H), 3.85 (s, 3H), 3.78 (s, 3H); *m*/*z*: calcd for C_27_H_25_N_4_O_2_ [M + H]^+^, 437.2; found, 437.0.

#### 4.2.15 2-(1*H*-Indazol-3-yl)acetohydrazide (**14a**)

Prepared according to method C. ^1^H NMR (400 MHz, CD_3_OD) δ 7.71–7.80 (m, 1H), 7.43–7.51 (m, 1H), 7.31–7.41 (m, 1H), 7.03–7.18 (m, 1H), 3.86 (s, 2H).

#### 4.2.16 3-((5,6-Bis(4-methoxyphenyl)-1,2,4-triazin-3-yl)methyl)-1*H*-indazole (**14**)

Prepared according to method D. Residue was purified by flash chromatography on silica eluting with 0–2% methanol in DCM to give the title compound **14** (37 mg, 40%). ^1^H NMR (400 MHz, DMSO-*d*_6_) δ 12.81–12.87 (br s, 1H), 7.80–7.87 (m, 1H), 7.45 (m, 5H), 7.30–7.37 (m, 1H), 7.06–7.13 (m, 1H), 6.99 (m, 2H), 6.92 (m, 2H), 4.77 (m, 2H), 3.79 (s, 3H), 3.77 (s, 3H); *m*/*z*: calcd for C_25_H_22_N_5_O_2_ [M + H]^+^, 424.2; found, 424.0.

#### 4.2.17 Methyl 2-(6-Fluoro-1*H*-indol-3-yl)acetate (**15a**)

Prepared according to method B. ^1^H NMR (400 MHz, CDCl_3_) δ 7.97–8.17 (m, 1H), 7.47–7.59 (m, 1H), 7.14–7.22 (m, 1H), 7.01–7.11 (m, 1H), 6.93 (d, *J* = 1.76 Hz, 1H), 3.78 (s, 2H), 3.73 (s, 3H).

#### 4.2.18 2-(6-Fluoro-1*H*-indol-3-yl)acetohydrazide (**15b**)

Prepared according to method C. ^1^H NMR (400 MHz, DMSO-*d*_6_) δ 10.77–11.02 (m, 1H), 9.03–9.18 (m, 1H), 7.48–7.62 (m, 1H), 7.17 (d, *J* = 2.26 Hz, 2H), 6.68–6.92 (m, 1H), 4.17 (br s, 2H), 3.39–3.53 (m, 2H).

#### 4.2.19 3-((5,6-Diphenyl-1,2,4-triazin-3-yl)methyl)-6-fluoro-1*H-*indole (**15**)

Prepared according to method D. The resulting residue was purified by flash chromatography with a gradient 0–50% ethyl acetate in heptane to give the title compound **15** (109 mg, 30%). ^1^H NMR (400 MHz, DMSO-*d*_6_) δ 11.03 (br s, 1H), 7.71 (m, 1H), 7.31–7.51 (m, 11H), 7.08–7.16 (m, 1H), 6.87 (m, 1H), 4.56 (s, 2H); *m*/*z*: calcd for C_24_H_18_FN_4_ [M + H]^+^, 381.1; found, 381.2.

#### 4.2.20 Methyl 2-(6-Methyl-1*H*-indol-3-yl)acetate (**16a**)

Prepared according to method B. ^1^H NMR (400 MHz, CDCl_3_) δ 7.94 (s, 1H), 7.52 (d, *J* = 8.1 Hz, 1H), 7.18 (dp, *J* = 1.5, 0.8 Hz, 1H), 7.10–7.15 (m, 1H), 7.00 (ddd, *J* = 8.1, 1.4, 0.6 Hz, 1H), 3.79 (d, *J* = 1.0 Hz, 2H), 3.72 (s, 3H), 2.48 (s, 3H).

#### 4.2.21 2-(6-Methyl-1*H*-indol-3-yl)acetohydrazide (**16b**)

Prepared according to method C. ^1^H NMR (400 MHz, DMSO-*d*_6_) δ 10.54–10.74 (m, 1H), 9.07 (br s, 1H), 7.36–7.48 (m, 1H), 6.97–7.21 (m, 2H), 6.80 (d, *J* = 7.28 Hz, 1H), 4.16 (br s, 2H), 3.40 (s, 2H), 2.26–2.43 (m, 3H).

#### 4.2.22 3-((5,6-Diphenyl-1,2,4-triazin-3-yl)methyl)-6-methyl-1*H*-indole (**16**)

Prepared according to method D. The resulting residue was purified by flash chromatography eluting with a gradient 0–50% of ethyl acetate in heptane. The appropriate fractions were combined, and the solvent was removed at reduced pressure to afford the title compound **16** (59 mg, 32%). ^1^H NMR (400 MHz, CDCl_3_) δ 8.10 (br s, 1H), 7.77–7.88 (m, 1H), 7.48–7.62 (m, 4H), 7.22–7.47 (m, 8H), 7.16 (s, 1H), 6.93–7.05 (m, 1H), 4.70 (s, 2H); *m*/*z*: calcd For C_25_H_20_N_4_ [M + H]^+^, 377.2; found, 377.0.

#### 4.2.23 3-((5,6-Diphenyl-1,2,4-triazin-3-yl)methyl)-1*H*-pyrrolo[3,2-*c*]pyridine (**17**)

Prepared according to method D. The solution was filtered and concentrated under reduced pressure. The resulting residue was purified by flash chromatography eluting with a gradient 0–50% of 9:1:0.1 DCM/methanol/NH_4_OH in DCM. The resulting residue was loaded onto an SCX cartridge washed with methanol and then eluted 2 M NH_3_ in methanol. The basic fractions were combined to give the title compound **17** (13 mg, 23%). ^1^H NMR (400 MHz, CDCl_3_) δ 9.24 (br s, 1H), 8.61 (s, 1H), 8.11 (m, 1H), 7.65 (m, 1H), 6.99–7.41 (m, 12H), 4.53 (s, 2H); *m*/*z*: calcd for C_23_H_17_N_5_ [M + H]^+^, 364.1; found, 364.2.

#### 4.2.24 3-((5,6-Diphenyl-1,2,4-triazin-3-yl)methyl)-1*H*-pyrrolo[3,2-*c*]pyridine (**18**)

Prepared according to method D. The resulting residue was purified by flash chromatography eluting with a gradient 0–50% of 9:1:0.1 DCM/methanol/NH_4_OH in DCM. The resulting residue was loaded onto an SCX cartridge, washed with methanol, and then eluted 2 M NH_3_ in methanol. The basic fractions were combined to give the title compound **18** (37 mg, 33%). ^1^H NMR (400 MHz, DMSO-*d*_6_) δ 4.61 (s, 2H), 7.27–7.53 (m, 10H), 7.60–7.75 (m, 2H), 8.02–8.17 (m, 1H), 8.63–8.82 (m, 1H), 11.43–11.63 (m, 1H); *m*/*z*: calcd for C_23_H_18_N_5_ [M + H]^+^, 364.1; found, 364.0.

#### 4.2.25 Methyl 2-(1*H*-pyrrolo[2,3-*b*]pyridin-3-yl)acetate (**19a**)

Prepared according to method B. ^1^H NMR (400 MHz, CDCl_3_) δ 3.63 (s, 3H), 3.70 (s, 2H), 6.99–7.07 (m, 1H), 7.24–7.30 (m, 1H), 7.80–7.99 (m, 1H), 8.13–8.36 (m, 1H), 11.01–11.42 (m, 1H).

#### 4.2.26 2-(1*H*-Pyrrolo[2,3-*b*]pyridin-3-yl)acetohydrazide (**19b**)

Prepared according to method C. ^1^H NMR (400 MHz), CD_3_OD δ 3.62 (s, 2H), 7.05–7.13 (m, 1H), 7.28–7.35 (m, 1H), 8.02–8.07 (m, 1H), 8.14–8.20 (m, 1H).

#### 4.2.27 3-((5,6-Diphenyl-1,2,4-triazin-3-yl)methyl)-1*H*-pyrrolo[2,3-*b*]pyridine (**19**)

Prepared according to method D. Residue was purified by flash chromatography on silica eluting with 0–3% methanol in DCM. The resulting residues were washed with ether and dried to give the title compound **19** (37 mg, 23%). ^1^H NMR (400 MHz, DMSO-*d*_6_) δ 4.59 (s, 2H), 6.97–7.13 (m, 1H), 7.27–7.62 (m, 11H), 8.04–8.29 (m, 2H) 11.51 (br s, 1H); *m*/*z*: calcd for C_23_H_18_N_5_ [M + H]^+^, 364.1; found, 364.0.

#### 4.2.28 2-Imidazo[1,2-*a*]pyridin-3-ylacetohydrazide (**20a**)

Prepared according to method C. ^1^H NMR (400 MHz, CDCl_3_) δ 3.47 (br s, 2H), 3.87 (d, *J* = 0.7 Hz, 2H), 6.85 (td, *J* = 6.8, 1.2 Hz, 1H), 7.19 (ddd, *J* = 9.2, 6.7, 1.3 Hz, 1H), 7.48 (d, *J* = 0.7 Hz, 1H), 7.56 (dt, *J* = 9.1, 1.1 Hz, 2H), 8.07 (dt, *J* = 7.0, 1.2 Hz, 1H).

#### 4.2.29 3-((5,6-Diphenyl-1,2,4-triazin-3-yl)methyl)imidazo[1,2-*a*]pyridine (**20**)

Prepared according to method D. The material was purified by flash chromatography using 3–5% MeOH in DCM. The relevant fraction was concentrated to give a yellow gum, which was further purified by preparative HPLC (basic conditions), concentrated, further purified by preparative HPLC (acidic conditions), and concentrated. The resulting residues were taken up in methanol and passed through an SCX cartridge eluting with methanol and then 2 M NH_3_ in methanol. The NH_3_/methanol fractions were combined and concentrated to give the title compound **20** (56 mg, 29%). ^1^H NMR (400 MHz, CDCl_3_) δ 4.86 (s, 2H), 6.85–6.90 (m, 1H), 7.19–7.24 (m, 1H), 7.31–7.47 (m, 6H), 7.49–7.55 (m, 4H), 7.66 (d, *J* = 9.3 Hz, 1H), 7.75–7.76 (m, 1H), 8.56 (d, *J* = 7.0 Hz, 1H); *m*/*z*: calcd for C_23_H_19_N_5_ [M + H]^+^, 364.1; found, 365.2.

#### 4.2.30 Hexanehydrazide (**21a**)

Prepared according to method C. ^1^H NMR (400 MHz, CDCl_3_) δ 6.76–7.04 (m, 1H), 3.93 (br s, 2H), 2.16 (t, *J* = 7.5 Hz, 2H), 1.60–1.75 (m, 2H), 1.21–1.44 (m, 4H), 0.82–0.99 (m, 3H).

#### 4.2.31 3-Pentyl-5,6-diphenyl-1,2,4-triazine (**21**)

Prepared according to method D. The reaction mixture was evaporated to dryness and then partitioned between water and DCM and passed through a hydrophobic frit. The organics were concentrated and then purified by preparative HPLC (acidic conditions) to give the title compound **21** (37 mg, 15%). ^1^H NMR (400 MHz, CDCl_3_) δ 7.44–7.51 (m, 4H), 7.23–7.39 (m, 6H), 3.07–3.21 (m, 2H), 1.82–1.99 (m, 2H), 1.28–1.46 (m, 4H), 0.83–0.93 (m, 3H); *m*/*z*: calcd for C_20_H_21_N_3_ [M + H]^+^, 304.2; found, 304.2.

#### 4.2.32 Preparation of 3-(3-Pyridyl)propanehydrazide (**22a**)

Prepared according to method C. ^1^H NMR (400 MHz, CDCl_3_) δ 8.47 (d, *J* = 1.0 Hz, 2H), 7.49–7.60 (m, 1H), 7.24 (dd, *J* = 7.8, 4.8 Hz, 1H), 7.04 (br s, 1H), 3.55–4.22 (m, 1H), 3.01 (t, *J* = 7.5 Hz, 2H), 2.48 (t, *J* = 7.5 Hz, 2H), 1.75–2.33 (m, 1H).

#### 4.2.33 5,6-Diphenyl-3-(2-(pyridin-3-yl)ethyl)-1,2,4-triazine (**22**)

Prepared according to method D. The reaction mixture was evaporated to dryness and then partitioned between water and DCM and passed through a hydrophobic frit. The resulting residues were purified by preparative HPLC (acidic conditions). The relevant fractions were passed through an SCX cartridge eluting the desired product with 2 M NH_3_/methanol and concentrated to give the title compound **22** (37 mg, 17%). ^1^H NMR (400 MHz, CDCl_3_) δ 3.33–3.41 (m, 2H), 3.54–3.63 (m, 2H), 7.22–7.28 (m, 2H), 7.32–7.51 (m, 6H), 7.52–7.60 (m, 3H), 7.64–7.72 (m, 1H), 8.50 (m, 1H), 8.57–8.62 (m, 1H); *m*/*z*: calcd for C_22_H_19_N_4_ [M + H]^+^, 339.2; found, 339.0.

#### 4.2.34 2-(5-Methyl-1*H*-pyrazol-3-yl)acetohydrazide (**23a**)

Prepared according to method C. ^1^H NMR (400 MHz, DMSO-*d*_6_) δ 2.14 (s, 3H), 3.27 (s, 2H), 3.80–4.44 (m, 2H), 5.83 (s, 1H), 9.06 (s, 1H).

#### 4.2.35 3-((5-Methyl-1*H*-pyrazol-3-yl)methyl)-5,6-diphenyl-1,2,4-triazine (**23**)

Prepared according to method D. The mixture was evaporated to dryness, then partitioned between water and DCM, passed through a hydrophobic frit, and evaporated to dryness. The crude product was purified by prep HPLC (acidic conditions). Fractions containing the desired product were concentrated. The resulting residues were further purified by preparative HPLC (basic conditions). The fractions containing the product were extracted with DCM and passed through a hydrophobic frit, and the solvent was removed under reduced pressure and dried to give the title compound **23** (32 mg, 15%). ^1^H NMR (400 MHz, CDCl_3_) δ 7.51–7.59 (m, 4H), 7.30–7.48 (m, 6H), 6.13 (s, 1H), 4.59 (s, 2H), 2.30 (s, 3H); *m*/*z*: calcd for C_20_H_18_N_5_ [M + H]^+^, 328.1; found, 328.0.

#### 4.2.36 5,6-Diphenyl-*N*-(pyridin-3-ylmethyl)-1,2,4-triazin-3-amine (**24**)

3-Bromo-5,6-diphenyl-1,2,4-triazine (75.0 mg, 0.240 mmol) and K_2_CO_3_ (0.0996 g, 0.721 mmol) were combined in acetonitrile (0.500 mL). 3-Pyridylmethanamine (0.0312 g, 0.288 mmol) was added, and the reaction was heated to 150 °C for 30 min under microwave radiation. The reaction was diluted with DCM and water. The layers were separated. The organics were washed with brine, dried over Na_2_SO_4_, filtered, and the solvent was removed at reduced pressure. The resulting residue was purified by flash chromatography eluting with a gradient 0–50% methanol in DCM. The appropriate fractions were combined, and the solvent was removed at reduced pressure to afford the title compound **24** (46 mg, 56%). ^1^H NMR (400 MHz, CDCl_3_) δ 8.73 (dd, *J* = 2.3, 0.8, 1H), 8.58 (dd, *J* = 4.8, 1.7, 1H), 7.81 (d, *J* = 8.0, 1H), 7.52–7.28 (m, 11H), 5.88 (br s, 1H), 4.88 (d, *J* = 6.2, 2H); *m*/*z*: calcd for C_21_H_18_N_5_ [M + H]^+^, 340.1; found, 340.2.

#### 4.2.37 *N*-(5-Methyl-1*H*-pyrazol-3-yl)-5,6-diphenyl-1,2,4-triazin-3-amine (**25**)

3-Bromo-5,6-diphenyl-1,2,4-triazine (75 mg, 0.240 mmol), 5-methyl-1*H*-pyrazol-3-amine (33 mg, 0.336 mmol), Xantphos (17 mg, 0.0288 mmol), K_2_CO_3_ (166 mg, 1.20 mmol), and tris(dibenzylideneacetone)dipalladium(0) (14 mg, 0.0240 mmol) were combined in 1,4-dioxane (1.00 mL) in a sealed vial. The vial was evacuated and argon-bubbled through the suspension for 10 min. The reaction was heated to 150 °C for 1 h. The reaction was diluted with DCM and washed with water. The layers were separated and the organics were dried over Na_2_SO_4_, filtered, and the solvent was removed at reduced pressure. The resulting residue was purified by flash chromatography eluting with a gradient 0–50% of 90/10/1DCM/methanol/NH_4_OH in DCM. The appropriate fractions were combined, and the solvent was removed at reduced pressure. The resulting residues were further purified by preparative HPLC (basic conditions). The appropriate fractions were combined, and the solvent was removed at reduced pressure to afford the title compound **25** (7 mg, 9%). ^1^H NMR (400 MHz, DMSO-*d*_6_) δ 11.92–12.13 (m, 1H), 10.24–10.44 (m, 1H), 7.28–7.56 (m, 10H), 6.39–6.53 (m, 1H), 2.24 (s, 3H); *m*/*z*: calcd for C_19_H_17_N_7_ [M + H]^+^, 329.1; found, 329.2.

#### 4.2.38 3-((5-(2-(Trifluoromethoxy)phenyl)-1,2,4-triazin-3-yl)methyl)-1*H*-indole (**26**)

Prepared according to method E. Daicel AD-H column 4.6 × 250 mm^2^, 15% methanol, 5 mL/min, retention time 3.5 min, the title compound **26** (28 mg, 19%). ^1^H NMR (400 MHz, DMSO-*d*_6_) δ 10.95 (s, 1H), 9.63 (s, 1H), 7.95 (m, 1H), 7.76 (m, 1H), 7.67–7.55 (m, 3H), 7.34 (m, 1H), 7.27 (m, 1H), 7.06 (m, 1H), 6.96 (m, 1H), 4.55 (s, 2H). *m*/*z*: calcd for C_19_H_14_F_3_N_4_O [M + H]^+^, 371.1; found, 371.0.

#### 4.2.39 3-((6-(2-(Trifluoromethoxy)phenyl)-1,2,4-triazin-3-yl)methyl)-1*H-*indole (**27**)

Prepared according to method E. Daicel AD-H column 4.6 × 250 mm^2^, 15% methanol, 5 mL/min, retention time 4.9 min, the title compound **27** (3 mg, 2%). ^1^H NMR (400 MHz, DMSO-*d*_6_) δ 10.96 (s, 1H), 9.05 (s, 1H), 7.91 (m, 1H), 7.72 (m, 1H), 7.66–7.58 (m, 2H), 7.55 (m, 1H), 7.35 (m, 1H), 7.30 (m, 1H), 7.07 (m, 1H), 6.96 (m, 1H), 4.57 (s, 2H); *m*/*z*: calcd for C_19_H_14_F_3_N_4_O [M + H]^+^, 371.1; found, 371.0.

#### 4.2.40 3-[(6-Methyl-5-phenyl-1,2,4-triazin-3-yl)methyl]-1*H*-indole (**28**)

Prepared according to method E. 48 mg, 28%. Analytical RP-HPLC method A, retention time 2.48 min. ^1^H NMR (400 MHz, CDCl_3_) δ 2.79 (s, 3H), 4.64 (s, 2H), 7.11–7.17 (m, 1H), 7.17–7.24 (m, 1H), 7.24–7.28 (m, 1H), 7.32–7.40 (m, 1H), 7.54 (d, *J* = 1.76 Hz, 3H), 7.63–7.73 (m, 2H), 7.84–7.95 (m, 1H), 8.06–8.27 (m, 1H). *m*/*z*: calcd for C_19_H_17_N_4_ [M + H]^+^, 301.1; found, 301.2.

#### 4.2.41 3-[(5-Methyl-6-phenyl-1,2,4-triazin-3-yl)methyl]-1*H*-indole (**29**)

Prepared according to method E. 4.3 mg, 3%. Analytical RP-HPLC method A, retention time 2.45 min. ^1^H NMR (400 MHz, CDCl_3_) δ 2.58 (s, 3H), 4.62 (s, 2H), 7.11–7.18 (m, 1H), 7.18–7.25 (m, 1H), 7.30–7.33 (m, 1H), 7.34–7.41 (m, 1H), 7.51 (d, *J* = 1.76 Hz, 3H), 7.59–7.66 (m, 2H), 7.85–7.93 (m, 1H), 8.01–8.17 (m, 1H). *m*/*z*: calcd for C_19_H_17_N_4_ [M + H]^+^, 301.1; found, 301.2.

#### 4.2.42 3-((6-Phenyl-5-(pyridazin-3-yl)-1,2,4-triazin-3-yl)methyl)-1*H*-indole (**30**)

Prepared according to method E. 14.7 mg, 6%. Analytical RP-HPLC method A, retention time 2.31 min. ^1^H NMR (400 MHz, CDCl_3_) δ 9.17–9.25 (m, 1H), 8.04–8.18 (m, 2H), 7.87–7.94 (m, 1H), 7.60–7.69 (m, 1H), 7.46 (d, *J* = 1.5 Hz, 2H), 7.28–7.43 (m, 5H), 7.12–7.24 (m, 2H), 4.76 (s, 2H). *m*/*z*: calcd for C_22_H_17_N_6_ [M + H]^+^, 365.1; found, 365.0.

#### 4.2.43 3-((5-Phenyl-6-(pyridazin-3-yl)-1,2,4-triazin-3-yl)methyl)-1*H*-indole (**31**)

Prepared according to method E. 7 mg, 3%. Analytical RP-HPLC method A, retention time 2.30 min. ^1^H NMR (400 MHz, CDCl_3_) δ 9.18–9.25 (m, 1H), 8.07–8.20 (m, 1H), 7.81–7.92 (m, 2H), 7.51–7.58 (m, 1H), 7.43–7.49 (m, 2H), 7.29–7.40 (m, 5H), 7.09–7.24 (m, 2H), 4.78 (s, 2H). *m*/*z*: calcd for C_22_H_17_N_6_ [M + H]^+^, 365.1; found, 365.0.

#### 4.2.44 3-((5-Phenyl-6-(pyrimidin-2-yl)-1,2,4-triazin-3-yl)methyl)-1*H*-indole or 3-((6-phenyl-5-(pyrimidin-2-yl)-1,2,4-triazin-3-yl)methyl)-1*H*-indole (**32**)

Prepared according to method E. Analytical RP-HPLC method A, retention time 2.34 min. ^1^H NMR (400 MHz, CDCl_3_) δ 8.75–8.88 (m, 2H), 8.07–8.18 (m, 1H), 7.88–7.94 (m, 1H), 7.28–7.47 (m, 8H), 7.11–7.24 (m, 2H), 4.77 (s, 2H); *m*/*z*: calcd for C_22_H_17_N_6_ [M + H]^+^, 365.1; found, 365.0.

#### 4.2.45 4-(3-((1*H*-Indol-3-yl)methyl)-6-phenyl-1,2,4-triazin-5-yl)-*N*-methylbenzenesulfonamide (**33**)

Prepared according to method E. 3.6 mg, 5%. Analytical RP-HPLC method A, retention time 2.35 min. ^1^H NMR (400 MHz, DMSO-*d*_6_) δ 10.97 (s, 1H), 7.78–7.69 (m, 3H), 7.69–7.62 (m, 2H), 7.54 (d, *J* = 5.0, 1H), 7.50–7.31 (m, 7H), 7.12–7.04 (m, 1H), 7.00 (m, 1H), 4.60 (s, 2H), 2.40 (d, *J* = 4.9, 3H). *m*/*z*: calcd for C_25_H_22_N_5_O_2_S [M + H]^+^, 456.1; found, 456.0.

#### 4.2.46 4-(3-((1*H*-Indol-3-yl)methyl)-5-phenyl-1,2,4-triazin-6-yl)-*N*-methylbenzenesulfonamide (**34**)

Prepared according to method E. 3.8 mg, 5%. Analytical RP-HPLC method A, retention time 2.39 min. ^1^H NMR (400 MHz, DMSO-*d*_6_) δ 10.97 (s, 1H), 7.80–7.73 (m, 2H), 7.73–7.64 (m, 3H), 7.56–7.50 (m, 1H), 7.49–7.43 (m, 3H), 7.42–7.31 (m, 4H), 7.12–7.04 (m, 1H), 7.03–6.96 (m, 1H), 4.60 (s, 2H), 2.41 (d, *J* = 4.9, 3H). *m*/*z*: calcd for C_25_H_22_N_5_O_2_S [M + H]^+^, 456.1; found, 456.0.

#### 4.2.47 3-((5-(4-(Methylsulfonyl)phenyl)-6-phenyl-1,2,4-triazin-3-yl)methyl)-1*H*-indole (**35**)

Prepared according to method E. 3.7 mg, 2%. Analytical RP-HPLC method A, retention time 2.34 min. ^1^H NMR (400 MHz, DMSO-*d*_6_) δ 10.96 (s, 1H), 7.94–7.88 (m, 2H), 7.74–7.67 (m, 3H), 7.51–7.47 (m, 2H), 7.46–7.38 (m, 3H), 7.36–7.32 (m, 2H), 7.08 (m, 1H), 7.00 (m, 1H), 4.60 (s, 2H), 3.24 (s, 3H); *m*/*z*: calcd for C_25_H_21_N_4_O_2_S [M + H]^+^, 441.1; found, 441.0.

#### 4.2.48 3-((6-(4-(Methylsulfonyl)phenyl)-5-phenyl-1,2,4-triazin-3-yl)methyl)-1*H*-indole (**36**)

Prepared according to method E. 5.6 mg, 3%. ^1^H NMR (400 MHz, DMSO-*d*_6_) δ 10.97 (s, 1H), 7.96–7.91 (m, 2H), 7.77–7.70 (m, 3H), 7.50–7.45 (m, 3H), 7.42–7.38 (m, 2H), 7.38–7.33 (m, 2H), 7.08 (m, 1H), 7.00 (m, 1H), 4.60 (s, 2H), 3.25 (s, 3H). *m*/*z*: calcd for C_25_H_21_N_4_O_2_S [M + H]^+^, 441.1; found, 441.0.

#### 4.2.49 4-(3-((1*H*-Indol-3-yl)methyl)-6-phenyl-1,2,4-triazin-5-yl)benzyl Acetate (**37**)

Prepared according to method E. 102 mg, 14%. Analytical RP-HPLC method A, retention time 2.73 min. ^1^H NMR (400 MHz, CDCl_3_) δ 8.14–8.09 (m, 1H), 7.97–7.94 (m, 1H), 7.56–7.51 (m, 4H), 7.45–7.29 (m, 7H), 7.25–7.15 (m, 2H), 5.13 (s, 2H), 4.72–4.71 (m, 2H), 2.14–2.14 (m, 3H); *m*/*z*: calcd for C_27_H_23_N_4_O_2_ [M + H]^+^, 435.2; found, 435.0.

#### 4.2.50 4-(3-((1*H*-Indol-3-yl)methyl)-5-phenyl-1,2,4-triazin-6-yl)benzyl Acetate (**38**)

Prepared according to method E. 99 mg, 14%. Analytical RP-HPLC method A, retention time 2.76 min. ^1^H NMR (400 MHz, CDCl_3_) δ 8.13–8.10 (m, 1H), 7.97–7.94 (m, 1H), 7.56–7.52 (m, 4H), 7.47–7.32 (m, 7H), 7.25–7.15 (m, 2H), 5.15–5.14 (m, 2H), 4.72 (s, 2H), 2.14 (s, 3H); *m*/*z*: calcd for C_27_H_23_N_4_O_2_ [M + H]^+^, 435.2; found, 435.0.

#### 4.2.51 (4-(3-((1*H*-Indol-3-yl)methyl)-6-phenyl-1,2,4-triazin-5-yl)phenyl)methanol (**39**)

4-(3-((1*H*-Indol-3-yl)methyl)-6-phenyl-1,2,4-triazin-5-yl)benzyl acetate (**37**) (102 mg, 0.235 mmol) was suspended/dissolved in methanol (10 mL), K_2_CO_3_ (95 mg, 0.690 mmol) was added, and the reaction mixture was stirred at room temperature for 4 h. The reaction was concentrated to dryness and partitioned between DCM and 0.5 M HCl. The phases were mixed, separated, and the aq. layer was back-extracted with DCM (2×). Combined organics were washed with sat. NaCl solution, dried over sodium sulfate, filtered, and concentrated to dryness. The resulting residues were purified by flash chromatography using 0–3.5% methanol in DCM. The appropriate fractions were collected and concentrated to dryness to give (4-(3-((1*H*-indol-3-yl)methyl)-6-phenyl-1,2,4-triazin-5-yl)phenyl)methanol (**39**) (92 mg, 90%). ^1^H NMR (400 MHz, CDCl_3_) δ 8.13–8.09 (m, 1H), 7.97–7.95 (m, 1H), 7.56–7.51 (m, 4H), 7.44–7.32 (m, 7H), 7.25–7.15 (m, 2H), 4.76–4.71 (m, 4H), 1.75 (t, *J* = 5.9 Hz, 1H). *m*/*z*: calcd for C_25_H_21_N_4_O [M + H]^+^, 393.2; found, 393.0.

#### 4.2.52 (4-(3-((1*H*-Indol-3-yl)methyl)-5-phenyl-1,2,4-triazin-6-yl)phenyl)methanol (**40**)

4-(3-((1*H*-Indol-3-yl)methyl)-5-phenyl-1,2,4-triazin-6-yl)benzyl acetate (**38**) (100 mg, 0.230 mmol) was suspended/dissolved in methanol (10 mL), K_2_CO_3_ (93 mg, 0.670 mmol) added, and the reaction mixture stirred at room temperature for 4 h. The reaction was concentrated to dryness and partitioned between DCM and 0.5 M HCl. The phases were mixed and separated, and the aq. layer was back-extracted with DCM (2×). Combined organics were washed with sat. NaCl solution, dried over sodium sulfate, filtered, and concentrated to dryness. The resulting residue was purified by flash column chromatography using 0–3.5% methanol in DCM. The appropriate fractions were collected to give (4-(3-((1*H*-indol-3-yl)methyl)-5-phenyl-1,2,4-triazin-6-yl)phenyl)methanol (**40**) (90 mg, 90%). ^1^H NMR (400 MHz, CDCl_3_) δ 8.12 (s, 1H), 7.97–7.95 (m, 1H), 7.56–7.52 (m, 4H), 7.46–7.32 (m, 7H), 7.25–7.15 (m, 2H), 4.76–4.72 (m, 4H), 1.77–1.72 (m, 1H).

#### 4.2.53 4-(4-(3-((1*H*-Indol-3-yl)methyl)-6-phenyl-1,2,4-triazin-5-yl)benzyl)morpholine (4**1**)

Prepared according to method E. 2 mg, 3%. Analytical RP-HPLC method A, retention time 1.42 min. ^1^H NMR (400 MHz, CDCl_3_) δ 8.10 (s, 1H), 7.97–7.94 (m, 1H), 7.54–7.49 (m, 4H), 7.43–7.33 (m, 5H), 7.32–7.29 (m, 2H), 7.25–7.15 (m, 2H), 4.72 (s, 2H), 3.72 (t, *J* = 4.5, 4H), 3.52–3.51 (m, 2H), 2.45 (t, *J* = 4.5, 4H); *m*/*z*: calcd for C_29_H_28_N_5_O [M + H]^+^, 462.2; found, 462.2.

#### 4.2.54 4-(4-(3-((1*H*-Indol-3-yl)methyl)-5-phenyl-1,2,4-triazin-6-yl)benzyl)morpholine (**42**)

Prepared according to method E. 5 mg, 7%. Analytical RP-HPLC method A, retention time 1.45 min. ^1^H NMR (400 MHz, CDCl_3_) δ 8.14–8.10 (m, 1H), 7.97–7.94 (m, 1H), 7.57–7.53 (m, 2H), 7.48 (td, *J* = 1.8, 8.4 Hz, 2H), 7.45–7.37 (m, 2H), 7.36–7.31 (m, 5H), 7.25–7.14 (m, 2H), 4.72 (s, 2H), 3.75–3.71 (m, 4H), 3.53 (s, 2H), 2.48–2.43 (m, 4H); *m*/*z*: calcd for C_29_H_28_N_5_O [M + H]^+^, 462.2; found, 462.2.

#### 4.2.55 1-(4-(3-((1*H*-Indol-3-yl)methyl)-6-phenyl-1,2,4-triazin-5-yl)phenyl)-*N*,*N*-dimethylmethanamine (**43**)

Prepared according to method E. 7 mg, 6%. Analytical RP-HPLC method A, retention time 1.82 min. ^1^H NMR (400 MHz, CDCl_3_) δ 8.11 (d, *J* = 0.5 Hz, 1H), 7.98–7.95 (m, 1H), 7.54–7.48 (m, 4H), 7.43–7.33 (m, 5H), 7.27 (s, 2H), 7.24–7.15 (m, 2H), 4.72 (s, 2H), 3.44 (s, 2H), 2.25 (s, 6H); *m*/*z*: calcd for C_27_H_25_N_5_ [M + H]^+^, 420.2; found, 420.2.

#### 4.2.56 1-(4-(3-((1*H*-Indol-3-yl)methyl)-5-phenyl-1,2,4-triazin-6-yl)phenyl)-*N*,*N*-dimethylmethanamine (**44**)

Prepared according to method E. 6 mg, 5%. Analytical RP-HPLC method A, retention time 1.88 min. ^1^H NMR (400 MHz, CDCl_3_) δ 8.12 (s, 1H), 7.97–7.94 (m, 1H), 7.55–7.53 (m, 2H), 7.49–7.46 (m, 2H), 7.45–7.29 (m, 7H), 7.25–7.14 (m, 2H), 4.72 (s, 2H), 3.45 (s, 2H), 2.26 (s, 6H); *m*/*z*: calcd for C_27_H_25_N_5_ [M + H]^+^, 420.2; found, 420.2.

#### 4.2.57 2-[[4-[3-(1*H*-Indol-3-ylmethyl)-5-phenyl-1,2,4-triazin-6-yl]phenyl]methyl]isoindoline-1,3-dione and 2-(4-(3-((1*H*-Indol-3-yl)methyl)-6-phenyl-1,2,4-triazin-5-yl)benzyl)isoindoline-1,3-dione (**45a** and **46a**)

Prepared according to method D. The solvents were removed under reduced pressure, and the mixture was purified by flash chromatography using 0–50% ethyl acetate in heptane. The appropriate fractions were collected and concentrated to dryness to give 2-[[4-[3-(1*H*-indol-3-ylmethyl)-5-phenyl-1,2,4-triazin-6-yl]phenyl]methyl]isoindoline-1,3-dione and 2-(4-(3-((1*H*-indol-3-yl)methyl)-6-phenyl-1,2,4-triazin-5-yl)benzyl)isoindoline-1,3-dione, 155 mg of which was used in the next step without further purification.

#### 4.2.58 *tert*-Butyl 3-((6-(4-((1,3-Dioxoisoindolin-2-yl)methyl)phenyl)-5-phenyl-1,2,4-triazin-3-yl)methyl)-1*H*-indole-1-carboxylate and *tert*-Butyl 3-((5-(4-((1,3-Dioxoisoindolin-2-yl)methyl)phenyl)-6-phenyl-1,2,4-triazin-3-yl)methyl)-1*H*-indole-1-carboxylate (**45b** and **46b**)

Prepared according to method H. The resulting residues were purified by flash chromatography using 0–50% ethyl acetate in heptane to give *tert*-butyl 3-((6-(4-((1,3-dioxoisoindolin-2-yl)methyl)phenyl)-5-phenyl-1,2,4-triazin-3-yl)methyl)-1*H*-indole-1-carboxylate and *tert*-butyl 3-((5-(4-((1,3-dioxoisoindolin-2-yl)methyl)phenyl)-6-phenyl-1,2,4-triazin-3-yl)methyl)-1*H*-indole-1-carboxylate, 97 mg of which was used without further purification.

#### 4.2.59 *tert*-Butyl 3-((6-(4-(Aminomethyl)phenyl)-5-phenyl-1,2,4-triazin-3-yl)methyl)-1*H*-indole-1-carboxylate and *tert*-Butyl 3-((5-(4-(Aminomethyl)phenyl)-6-phenyl-1,2,4-triazin-3-yl)methyl)-1*H*-indole-1-carboxylate (**45c** and **46c**)

To a solution of *tert*-butyl 3-((6-(4-((1,3-dioxoisoindolin-2-yl)methyl)phenyl)-5-phenyl-1,2,4-triazin-3-yl)methyl)-1*H*-indole-1-carboxylate and *tert*-butyl 3-((5-(4-((1,3-dioxoisoindolin-2-yl)methyl)phenyl)-6-phenyl-1,2,4-triazin-3-yl)methyl)-1*H*-indole-1-carboxylate (97.0 mg, 0.186 mmol) in ethanol (4 mL) was added hydrazine hydrate (0.060 mL), and the mixture esd stirred at 40 °C for 18 h, and then concentrated to dryness. The resulting residues were purified by flash chromatography using 0–10% methanol in DCM to give *tert*-butyl 3-((6-(4-(aminomethyl)phenyl)-5-phenyl-1,2,4-triazin-3-yl)methyl)-1*H*-indole-1-carboxylate and *tert*-butyl 3-((5-(4-(aminomethyl)phenyl)-6-phenyl-1,2,4-triazin-3-yl)methyl)-1*H*-indole-1-carboxylate, 65 mg of which was used without further purification.

#### 4.2.60 *tert*-Butyl 3-((6-(4-(Acetamidomethyl)phenyl)-5-phenyl-1,2,4-triazin-3-yl)methyl)-1*H*-indole-1-carboxylate and *tert*-Butyl 3-((5-(4-(Acetamidomethyl)phenyl)-6-phenyl-1,2,4-triazin-3-yl)methyl)-1*H*-indole-1-carboxylate (**45d** and **46d**)

To a solution of *tert*-butyl 3-((6-(4-(aminomethyl)phenyl)-5-phenyl-1,2,4-triazin-3-yl)methyl)-1*H*-indole-1-carboxylate and *tert*-butyl 3-((5-(4-(aminomethyl)phenyl)-6-phenyl-1,2,4-triazin-3-yl)methyl)-1*H*-indole-1-carboxylate (36.0 mg, 0.0732 mmol) in DCM (5.00 mL) were added triethylamine (0.102 mL, 0.732 mmol) and acetyl chloride (0.0631 mL, 0.884 mmol). The mixture was stirred at 40 °C for 3 h, after which the reaction mixture was concentrated to dryness. The resulting residues were purified by flash column chromatography using 0–10% methanol in DCM. The appropriate fractions were collected and concentrated to dryness to give *tert*-butyl 3-((6-(4-(acetamidomethyl)phenyl)-5-phenyl-1,2,4-triazin-3-yl)methyl)-1*H*-indole-1-carboxylate and *tert*-butyl 3-((5-(4-(acetamidomethyl)phenyl)-6-phenyl-1,2,4-triazin-3-yl)methyl)-1*H*-indole-1-carboxylate, 16 mg of which was used without further purification.

#### 4.2.61 Preparation of *N*-(4-(3-((1*H*-Indol-3-yl)methyl)-6-phenyl-1,2,4-triazin-5-yl)benzyl)acetamide (**45**) and *N*-(4-(3-((1*H*-Indol-3-yl)methyl)-5-phenyl-1,2,4-triazin-6-yl)benzyl)acetamide (**46**)

A mixture of *tert*-butyl 3-((6-(4-(acetamidomethyl)phenyl)-5-phenyl-1,2,4-triazin-3-yl)methyl)-1*H*-indole-1-carboxylate and *tert*-butyl 3-((5-(4-(acetamidomethyl)phenyl)-6-phenyl-1,2,4-triazin-3-yl)methyl)-1*H*-indole-1-carboxylate (16 mg, 0.03 mmol) was stirred at 40 °C in DCM (5.00 mL) and trifluoroacetic acid was added (0.06 mL). The reaction was stirred for 16 h and then concentrated to dryness. The resulting residues were purified by preparative HPLC (basic conditions), and the crude regioisomers were isolated to give **45** and **46**.

##### 4.2.61.1 *N*-(4-(3-((1*H*-Indol-3-yl)methyl)-6-phenyl-1,2,4-triazin-5-yl)benzyl)acetamide (**45**)

5 mg, 39%. Analytical RP-HPLC method B, retention time 2.2 min. ^1^H NMR (400 MHz, DMSO-*d*_6_) δ 10.95 (s, 1H), 8.36 (t, *J* = 5.9 Hz, 1H), 7.71 (d, *J* = 8.1 Hz, 1H), 7.49–7.31 (m, 9H), 7.26–7.21 (m, 2H), 7.07 (m, 1H), 6.99 (m, 1H), 4.56 (s, 2H), 4.27 (m, 2H), 1.87 (s, 3H); *m*/*z*: calcd for C_27_H_24_N_5_O [M + H]^+^, 434.2; found, 434.2.

##### 4.2.61.2 *N*-(4-(3-((1*H*-Indol-3-yl)methyl)-5-phenyl-1,2,4-triazin-6-yl)benzyl)acetamide (**46**)

5 mg, 39%. Analytical RP-HPLC method B, retention time 2.16 min. ^1^H NMR (400 MHz, DMSO-*d*_6_) δ 10.95 (s, 1H), 8.35 (m, 1H), 7.72 (d, *J* = 7.8, 1H), 7.50–7.37 (m, 7H), 7.37–7.32 (m, 2H), 7.23–7.19 (m, 2H), 7.07 (m, 1H), 7.00 (m, 1H), 4.56 (s, 2H), 4.25 (m, 2H), 1.86 (s, 3H); *m*/*z*: calcd for C_27_H_24_N_5_O [M + H]^+^, 434.2; found, 434.2.

#### 4.2.62 *tert*-Butyl 3-[[5-[4-(Methanesulfonamidomethyl)phenyl]-6-phenyl-1,2,4-triazin-3-yl]methyl]-1*H*-indole-1-carboxylate and *tert*-Butyl 3-[[5-[4-(Methanesulfonamidomethyl)phenyl]-6-phenyl-1,2,4-triazin-3-yl]methyl]-1*H*-indole-1-carboxylate (**47a** and **47b**)

To a solution of *tert*-butyl 3-((6-(4-(aminomethyl)phenyl)-5-phenyl-1,2,4-triazin-3-yl)methyl)-1*H*-indole-1-carboxylate and *tert*-butyl 3-((5-(4-(aminomethyl)phenyl)-6-phenyl-1,2,4-triazin-3-yl)methyl)-1*H*-indole-1-carboxylate (20 mg, 0.0407 mmol) at 0 °C in DCM (5 mL) were added triethylamine (0.0567 mL, 0.407 mmol) and methanesulfonyl chloride (0.00472 mL, 0.0610 mmol), and the reaction was stirred at room temperature for 20 h. After this time, a further portion of methanesulfonyl chloride (0.00472 mL, 0.0610 mmol) was added and the mixture was stirred for 24 h. The reaction mixture was concentrated, and the resulting residues were purified by flash chromatography using 0–10% methanol in DCM to give *tert*-butyl 3-[[5-[4-(methanesulfonamidomethyl)phenyl]-6-phenyl-1,2,4-triazin-3-yl]methyl]-1*H*-indole-1-carboxylate and *tert*-butyl 3-[[5-[4-(methanesulfonamidomethyl)phenyl]-6-phenyl-1,2,4-triazin-3-yl]methyl]-1*H*-indole-1-carboxylate, 22 mg of which was used without further purification.

#### 4.2.63 Preparation of *N*-(4-(3-((1*H*-Indol-3-yl)methyl)-6-phenyl-1,2,4-triazin-5-yl)benzyl)methanesulfonamide (**47**) and *N*-(4-(3-((1*H*-Indol-3-yl)methyl)-5-phenyl-1,2,4-triazin-6-yl)benzyl)methanesulfonamide (**48**)

A mixture of regioisomers *tert*-butyl 3-[[5-[4-(methanesulfonamidomethyl)phenyl]-6-phenyl-1,2,4-triazin-3-yl]methyl]-1*H*-indole-1-carboxylate (8.00 mg, 0.0140 mmol) and *tert*-butyl 3-[[6-[4-(methanesulfonamidomethyl)phenyl]-5-phenyl-1,2,4-triazin-3-yl]methyl]-1*H*-indole-1-carboxylate (8.00 mg, 0.0140 mmol) was stirred at 40 °C in DCM (5.00 mL) and TFA (0.06 mL) was added. The mixture was stirred for 24 h and then concentrated to dryness. The resulting residues were purified by preparative HPLC (basic conditions) to give **47** and **48**.

##### 4.2.63.1 *N*-(4-(3-((1*H*-indol-3-yl)methyl)-6-phenyl-1,2,4-triazin-5-yl)benzyl)methanesulfonamide (**47**)

2.1 mg, 11%. Analytical RP-HPLC method B, retention time 2.3 min. ^1^H NMR (400 MHz, DMSO-*d*_6_) δ 10.95 (s, 1H), 7.72 (d, *J* = 7.9 Hz, 1H), 7.61 (t, *J* = 6.3 Hz, 1H), 7.50–7.41 (m, 5H), 7.39–7.31 (m, 6H), 7.07 (ddd, *J* = 8.1, 6.9, 1.2 Hz, 1H), 6.99 (ddd, *J* = 8.1, 6.8, 1.0 Hz, 1H), 4.57 (s, 2H), 4.18 (d, *J* = 6.3 Hz, 2H), 2.83 (s, 3H); *m*/*z*: calcd for C_26_H_23_N_5_O_2_S [M + H]^+^, 470.2; found, 470.2.

##### 4.2.63.2 *N*-(4-(3-((1*H*-Indol-3-yl)methyl)-5-phenyl-1,2,4-triazin-6-yl)benzyl)methanesulfonamide (**48**)

3.3 mg, 17%. Analytical RP-HPLC method B, retention time 2.26 min. ^1^H NMR (400 MHz, DMSO-*d*_6_) δ 10.95 (s, 1H), 7.72 (d, *J* = 7.9 Hz, 1H), 7.59 (t, *J* = 6.4 Hz, 1H), 7.49–7.28 (m, 11H), 7.10–7.05 (m, 1H), 7.02–6.97 (m, 1H), 4.56 (s, 2H), 4.16 (d, *J* = 6.4 Hz, 2H), 2.83 (s, 3H); *m*/*z*: calcd for C_26_H_23_N_5_O_2_S [M + H]^+^, 470.2; found, 470.2.

#### 4.2.64 Methyl 4-(3-((1*H*-Indol-3-yl)methyl)-6-phenyl-1,2,4-triazin-5-yl)benzoate (**49**)

Prepared according to method E. 80 mg, 21%. Analytical RP-HPLC method A, retention time 2.82 min. ^1^H NMR (400 MHz, CDCl_3_) δ 8.15–8.22 (m, 1H), 8.02 (d, *J* = 8.5 Hz, 2H), 7.92–7.97 (m, 1H), 7.61 (s, 2H), 7.48–7.53 (m, 2H), 7.40–7.46 (m, 1H), 7.33 (s, 4H), 7.18–7.24 (m, 1H), 7.13–7.18 (m, 1H), 4.72 (s, 2H), 3.93 (s, 3H); *m*/*z*: calcd for C_26_H_21_N_4_O_2_ [M + H]^+^, 421.0; found, 421.0.

#### 4.2.65 Methyl 4-(3-((1*H*-Indol-3-yl)methyl)-5-phenyl-1,2,4-triazin-6-yl)benzoate (**50**)

Prepared according to method E. 69 mg, 18%. Analytical RP-HPLC method A, retention time 2.82 min. ^1^H NMR (400 MHz, CDCl_3_) δ 8.09–8.16 (m, 1H), 7.98 (d, *J* = 8.3 Hz, 2H), 7.91–7.96 (m, 1H), 7.59 (d, *J* = 8.5 Hz, 2H), 7.48 (s, 2H), 7.31–7.44 (m, 5H), 7.19–7.25 (m, 1H), 7.12–7.18 (m, 1H), 4.70–4.75 (m, 2H), 3.93 (s, 3H). *m*/*z*: calcd for C_26_H_21_N_4_O_2_ [M + H]^+^, 421.0; found, 421.0.

#### 4.2.66 4-(3-((1*H*-Indol-3-yl)methyl)-6-phenyl-1,2,4-triazin-5-yl)benzoic Acid (**51**)

Methyl 4-(3-((1*H*-indol-3-yl)methyl)-6-phenyl-1,2,4-triazin-5-yl)benzoate (**49**) (10 mg, 0.24 mmol) was suspended in methanol (0.500 mL) and stirred at room temperature, while NaOH (2.00 M, 35.7 μL, 0.0713 mmol) was added in one portion. The suspension was heated at reflux for 1 h during which time the solid dissolved to give an orange solution. Worked up by concentrating reaction mixture to dryness, dissolving the residue in water (2 mL), and acidifying by addition of 1 M HCl. The resulting suspension was filtered off and purified by preparative HPLC (acidic conditions). The relevant fractions were combined and concentrated to dryness to give 4-(3-((1*H*-indol-3-yl)methyl)-6-phenyl-1,2,4-triazin-5-yl)benzoic acid (**51**) (3 mg, 31%). Analytical RP-HPLC method A, retention time 2.50 min. ^1^H NMR (400 MHz, DMSO-*d*_6_) δ 13.03–13.20 (m, 1H), 10.90–11.03 (m, 1H), 7.93 (d, *J* = 8.3, 2H), 7.69–7.75 (m, 1H), 7.56–7.63 (m, 2H), 7.47 (d, *J* = 8.0, 3H), 7.34 (s, 4H), 7.04–7.11 (m, 1H), 6.96–7.03 (m, 1H), 4.54–4.64 (m, 2H); *m*/*z*: calcd for C_25_H_19_N_4_O_2_ [M + H]^+^, 407.1; found, 407.0.

#### 4.2.67 4-(3-((1*H*-Indol-3-yl)methyl)-5-phenyl-1,2,4-triazin-6-yl)benzoic Acid (**52**)

Methyl 4-(3-((1*H*-indol-3-yl)methyl)-5-phenyl-1,2,4-triazin-6-yl)benzoate (**50**) (10 mg, 0.24) was suspended in methanol (0.5 mL) and stirred at room temperature, while NaOH (2.00 M, 17.8 μL, 0.0357 mmol) was added in one portion. The suspension was heated at reflux for 3 h. After this time, a further portion of NaOH (2.00 M, 17.8 μL, 0.0357 mmol) was added and the suspension was stirred at reflux for a further 1 h, resulting in dissolution to give a brown solution. Worked up by concentrating reaction mixture to dryness, dissolving the residue in water (2 mL), and acidifying by the addition of 1 M HCl. The resulting suspension was filtered and purified by preparative HPLC (acidic conditions). The relevant fractions were combined and concentrated to dryness to give 4-(3-((1*H*-indol-3-yl)methyl)-5-phenyl-1,2,4-triazin-6-yl)benzoic acid (**52**) (1.2 mg, 13%). Analytical RP-HPLC method A, retention time 2.45 min. ^1^H NMR (400 MHz, DMSO-*d*_6_) δ 13.12–13.24 (m, 1H), 10.92–11.00 (m, 1H), 7.86–7.92 (m, 2H), 7.70–7.74 (m, 1H), 7.54–7.60 (m, 2H), 7.33–7.49 (m, 7H), 7.05–7.10 (m, 1H), 6.97–7.03 (m, 1H), 4.56–4.62 (m, 2H); *m*/*z*: calcd for C_25_H_19_N_4_O_2_ [M + H]^+^, 407.0; found, 407.0.

#### 4.2.68 Methyl 3-(3-((1*H*-Indol-3-yl)methyl)-5-phenyl-1,2,4-triazin-6-yl)benzoate (**53**)

Prepared according to method E. 27 mg, 31%. Analytical RP-HPLC method A, retention time 2.66 min. ^1^H NMR (400 MHz, CDCl_3_) δ 8.27–8.36 (m, 1H), 8.02–8.22 (m, 2H), 7.89–8.00 (m, 1H), 7.59–7.69 (m, 1H), 7.49–7.56 (m, 2H), 7.34 (m, 6H), 7.14–7.26 (m, 2H), 4.65–4.80 (m, 2H), 3.90 (s, 3H); *m*/*z*: calcd for C_26_H_21_N_4_O_2_ [M + H]^+^, 421.2; found, 421.2.

#### 4.2.69 Methyl 3-(3-((1*H*-Indol-3-yl)methyl)-6-phenyl-1,2,4-triazin-5-yl)benzoate (**54**)

Prepared according to method E. 14 mg, 16%. Analytical RP-HPLC method A, retention time 2.65 min. ^1^H NMR (400 MHz, CDCl_3_) δ 8.31–8.38 (m, 1H), 8.05–8.16 (m, 2H), 7.93–8.02 (m, 1H), 7.56–7.63 (m, 1H), 7.46–7.53 (m, 2H), 7.32–7.45 (m, 6H), 7.14–7.27 (m, 2H), 4.74 (s, 2H), 3.93 (s, 3H); *m*/*z*: calcd for C_26_H_21_N_4_O_2_ [M + H]^+^, 421.2; found, 421.0.

#### 4.2.70 3-(3-((1*H*-Indol-3-yl)methyl)-6-phenyl-1,2,4-triazin-5-yl)benzoic Acid (**55**)

Methyl 3-(3-((1*H*-indol-3-yl)methyl)-5-phenyl-1,2,4-triazin-6-yl)benzoate (**53**) (20 mg, 0.048 mmol) was stirred in tetrahydrofuran (THF). Lithium hydroxide hydrate (24.0 mg, 0.571 mmol) and water (1 mL) were added, and the reaction was heated to 50 °C overnight and then refluxed for 8 h. The solvent was removed at reduced pressure. The residue was taken up in ethyl acetate and acidified with 1 M HCl. The layers were separated. The organics were washed with water and dried over Na_2_SO_4_, filtered, and concentrated under reduced pressure. The resulting residue was purified using flash chromatography eluting with a gradient 0–95/5/0.5 DCM/methanol/AcOH in DCM. The appropriate fractions were collected and concentrated to dryness. The resulting material was further purified by preparative HPLC (acidic conditions) to give 3-(3-((1*H*-indol-3-yl)methyl)-6-phenyl-1,2,4-triazin-5-yl)benzoic acid (**55**) (16 mg, 86%). Analytical RP-HPLC method B, retention time 2.35 min. ^1^H NMR (400 MHz, CDCl_3_) δ 8.31–8.26 (m, 1H), 8.10 (m, 2H), 7.93 (d, *J* = 7.8 Hz, 1H), 7.73–7.65 (m, 1H), 7.54–7.47 (m, 2H), 7.47–7.39 (m, 2H), 7.39–7.30 (m, 4H), 7.24–7.12 (m, 2H), 4.72 (d, *J* = 0.9, 2H); *m*/*z*: calcd for C_25_H_19_N_4_O_2_ [M + H]^+^, 407.1; found, 407.0.

#### 4.2.71 3-(3-((1*H*-Indol-3-yl)methyl)-5-phenyl-1,2,4-triazin-6-yl)benzoic Acid (**56**)

Methyl 3-(3-((1*H*-indol-3-yl)methyl)-6-phenyl-1,2,4-triazin-5-yl)benzoate (**54**) (33 mg, 0.0761 mmol) was stirred in THF. Lithium hydroxide hydrate (24.0 mg, 0.571 mmol) and water (1 mL) were added, and the reaction was heated to reflux overnight. The solvent was removed at reduced pressure. The residue was taken up in ethyl acetate and acidified with 1 M HCl. The layers were separated. The organics were washed with water and dried over Na_2_SO_4_, and the solvent was removed at reduced pressure. The resulting material was further purified by preparative HPLC (acidic conditions) to give 3-(3-((1*H*-indol-3-yl)methyl)-5-phenyl-1,2,4-triazin-6-yl)benzoic acid (**56**) (13 mg, 57%). ^1^H NMR (400 MHz, CDCl_3_) δ 8.37–8.45 (m, 1H), 8.09–8.21 (m, 2H), 7.92–8.03 (m, 1H), 7.68 (m, 1H), 7.34–7.54 (m, 8H), 7.15–7.27 (m, 2H), 4.77 (s, 2H); *m*/*z*: calcd for C_25_H_19_N_4_O_2_ [M + H]^+^, 407.1; found, 407.0.

#### 4.2.72 3-(3-((1*H*-Indol-3-yl)methyl)-6-phenyl-1,2,4-triazin-5-yl)-*N*-methylbenzamide (**57**)

Prepared according to method G. The resulting residue was purified by flash chromatography eluting with a gradient 0–5% methanol in DCM. 12 mg, 6%. Analytical RP-HPLC method B, retention time 1.75 min. ^1^H NMR (400 MHz, CDCl_3_) δ 8.05–8.22 (m, 1H), 7.79–8.01 (m, 3H), 7.49–7.56 (m, 3H), 7.42–7.49 (m, 1H), 7.31–7.42 (m, 5H), 7.14–7.26 (m, 2H), 5.96–6.13 (m, 1H), 4.73 (s, 2H), 2.99 (d, *J* = 4.8, 3H); *m*/*z*: calcd for C_26_H_22_N_5_O [M + H]^+^, 420.2; found, 420.2.

#### 4.2.73 3-(3-((1*H*-Indol-3-yl)methyl)-5-phenyl-1,2,4-triazin-6-yl)-*N*-methylbenzamide (**58**)

Prepared according to method G. The resulting residue was purified by flash chromatography eluting with a gradient 0–5% methanol in DCM. 13 mg, 6%. Analytical RP-HPLC method B, retention time 2.21 min. ^1^H NMR (400 MHz, CDCl_3_) δ 8.09–8.25 (m, 1H), 7.93–8.00 (m, 1H), 7.88 (d, *J* = 0.8 Hz, 2H), 7.51 (d, *J* = 7.0 Hz, 3H), 7.31–7.46 (m, 6H), 7.14–7.28 (m, 2H), 5.72–5.94 (m, 1H), 4.65–4.82 (m, 2H), 2.90–3.07 (m, 3H); *m*/*z*: calcd for C_26_H_22_N_5_O [M + H]^+^, 420.2; found, 420.2.

#### 4.2.74 3-((6-Phenyl-5-(pyrazin-2-yl)-1,2,4-triazin-3-yl)methyl)-1*H*-pyrrolo[2,3-*c*]pyridine (**59**)

Prepared according to method F. 17 mg, 13%. Analytical RP-HPLC method B, retention time 1.25 min. ^1^H NMR (400 MHz, CDCl_3_) δ 9.23 (d, *J* = 1.5, 1H), 8.82 (s, 1H), 8.65 (d, *J* = 2.3, 2H), 8.48 (dd, *J* = 2.4, 1.6 Hz, 1H), 8.31 (s, 1H), 7.82 (d, *J* = 5.3, 1H), 7.52 (s, 1H), 7.46 (d, *J* = 6.8, 3H), 7.37 (d, *J* = 6.8, 2H), 4.76 (s, 2H); *m*/*z*: calcd for C_21_H_16_N_7_ [M + H]^+^, 366.1; found, 366.0.

#### 4.2.75 3-((5-Phenyl-6-(pyrazin-2-yl)-1,2,4-triazin-3-yl)methyl)-1*H*-pyrrolo[2,3-*c*]pyridine (**60**)

Prepared according to method F. 9 mg, 7%. Analytical RP-HPLC method B, retention time 1.25 min. ^1^H NMR (400 MHz, CDCl_3_) δ 8.97–9.08 (m, 1H), 8.75–8.88 (m, 1H), 8.60–8.70 (m, 1H), 8.47–8.56 (m, 1H), 8.24–8.36 (m, 1H), 7.77–7.87 (m, 1H), 7.53 (s, 1H), 7.35–7.50 (m, 5H), 4.78 (s, 2H). *m*/*z*: calcd for C_21_H_16_N_7_ [M + H]^+^, 366.1; found, 366.0.

#### 4.2.76 3-((6-Phenyl-5-(pyridazin-4-yl)-1,2,4-triazin-3-yl)methyl)-1*H*-pyrrolo[2,3-*c*]pyridine (**61**)

Prepared according to method F. 2 mg, 3%. Analytical RP-HPLC method A, retention time 1.42 min. ^1^H NMR (400 MHz, CDCl_3_) δ 9.24–9.30 (m, 2H), 8.80–8.84 (m, 1H), 8.55–8.75 (m, 1H), 8.28–8.33 (m, 1H), 7.78–7.82 (m, 1H), 7.71–7.75 (m, 1H), 7.40–7.56 (m, 6H), 4.75 (s, 2H); *m*/*z*: calcd for C_21_H_16_N_7_ [M + H]^+^, 366.1; found, 366.0.

#### 4.2.77 3-((5-Phenyl-6-(pyridazin-4-yl)-1,2,4-triazin-3-yl)methyl)-1*H*-pyrrolo[2,3-*c*]pyridine (**62**)

Prepared according to method F. 4 mg, 5%. Analytical RP-HPLC method A, retention time 1.43 min. ^1^H NMR (400 MHz, CDCl_3_) δ 9.29–9.34 (m, 1H), 9.19–9.24 (m, 1H), 8.85–9.04 (m, 1H), 8.79–8.84 (m, 1H), 8.27–8.33 (m, 1H), 7.75–7.80 (m, 1H), 7.41–7.57 (m, 7H), 4.76 (s, 2H); *m*/*z*: calcd for C_21_H_16_N_7_ [M + H]^+^, 366.1; found, 366.0.

#### 4.2.78 4-(4-(3-((1*H*-Pyrrolo[2,3-*c*]pyridin-3-yl)methyl)-6-phenyl-1,2,4-triazin-5-yl)benzyl)morpholine (**63**)

Prepared according to method F. 18 mg, 12%. Analytical RP-HPLC method B, retention time 1.29 min. ^1^H NMR (400 MHz, CDCl_3_) δ 8.94–9.28 (m, 1H), 8.78 (s, 1H), 8.24–8.32 (m, 1H), 7.77–7.84 (m, 1H), 7.38–7.56 (m, 6H), 7.33 (dd, *J* = 7.9, 3.1 Hz, 4H), 4.69 (s, 2H), 3.67–3.75 (m, 4H), 3.52 (s, 2H), 2.38–2.49 (m, 4H); *m*/*z*: calcd for C_28_H_27_N_6_O [M + H]^+^, 463.2; found, 463.2.

#### 4.2.79 4-(4-(3-((1*H*-Pyrrolo[2,3-*c*]pyridin-3-yl)methyl)-5-phenyl-1,2,4-triazin-6-yl)benzyl)morpholine (**64**)

Prepared according to method F. 19 mg, 13%. Analytical RP-HPLC method A, retention time 1.36 min. ^1^H NMR (400 MHz, CDCl_3_) δ 9.06–9.51 (m, 1H), 8.78 (s, 1H), 8.28 (d, *J* = 5.5 Hz, 1H), 7.81 (d, *J* = 5.3 Hz, 1H), 7.45–7.59 (m, 5H), 7.36 (d, *J* = 7.5 Hz, 3H), 7.30 (d, *J* = 8.3 Hz, 2H), 4.69 (s, 2H), 3.67–3.77 (m, 4H), 3.46–3.54 (m, 2H), 2.39–2.49 (m, 4H); *m*/*z*: calcd for C_28_H_27_N_6_O [M + H]^+^, 463.2; found, 463.2.

#### 4.2.80 Methyl 4-(3-((1*H*-Pyrrolo[2,3-*c*]pyridin-3-yl)methyl)-6-phenyl-1,2,4-triazin-5-yl)benzoate (**65**)

Prepared according to method F. 14 mg, 6%. Analytical RP-HPLC method C, retention time 1.38 min. ^1^H NMR (400 MHz, CD_3_OD) δ 8.61–8.71 (m, 1H), 8.02–8.12 (m, 1H), 7.91–8.00 (m, 2H), 7.75–7.85 (m, 1H), 7.56–7.66 (m, 3H), 7.42–7.49 (m, 3H), 7.35–7.41 (m, 2H), 4.67–4.73 (m, 2H), 3.90 (s, 3H); *m*/*z*: calcd for C_25_H20N_5_O_2_ [M + H]^+^, 422.2; found, 422.2.

#### 4.2.81 Methyl 4-(3-((1*H*-Pyrrolo[2,3-*c*]pyridin-3-yl)methyl)-5-phenyl-1,2,4-triazin-6-yl)benzoate (**66**)

Prepared according to method F. 22 mg, 9%. Analytical RP-HPLC method C, retention time 1.4 min. ^1^H NMR (400 MHz, CD_3_OD) δ 8.59–8.74 (m, 1H), 8.03–8.10 (m, 1H), 8.00 (d, *J* = 8.5 Hz, 2H), 7.76–7.85 (m, 1H), 7.57–7.65 (m, 3H), 7.48 (d, *J* = 1.3 Hz, 3H), 7.34 (s, 2H), 4.69 (s, 2H), 3.91 (s, 3H); *m*/*z*: calcd for C_25_H_20_N_5_O_2_ [M + H]^+^, 422.2; found, 422.2.

#### 4.2.82 4-(3-((1*H*-Pyrrolo[2,3-*c*]pyridin-3-yl)methyl)-6-phenyl-1,2,4-triazin-5-yl)-*N*-methylbenzamide (**67**)

Methyl 4-(3-((1*H*-pyrrolo[2,3-*c*]pyridin-3-yl)methyl)-6-phenyl-1,2,4-triazin-5-yl)benzoate (**65**) (14 mg, 0.033 mmol) was transferred to a microwave vial in 0.5 mL of methanol. To this was added 2 mL of 40% methylamine in water. The reaction mixture was then microwaved at 100 °C for 1 h. The reaction mixture was extracted twice with DCM, and the combined organics were concentrated to dryness. The resulting residues were purified by preparative HPLC (basic conditions) to give 4-(3-((1*H*-pyrrolo[2,3-*c*]pyridin-3-yl)methyl)-6-phenyl-1,2,4-triazin-5-yl)-*N*-methylbenzamide (**67**) (9 mg, 64%). Analytical RP-HPLC method C, retention time 1.33 min. ^1^H NMR (400 MHz, CD_3_OD) δ 8.62–8.69 (m, 1H), 8.02–8.10 (m, 1H), 7.72–7.82 (m, 3H), 7.61–7.65 (m, 1H), 7.59 (s, 2H), 7.32–7.51 (m, 5H), 4.69 (s, 2H), 2.90 (s, 3H); *m*/*z*: calcd for C_25_H_21_N_6_O [M + H]^+^, 421.2; found, 421.2.

#### 4.2.83 4-(3-((1*H*-Pyrrolo[2,3-*c*]pyridin-3-yl)methyl)-5-phenyl-1,2,4-triazin-6-yl)-*N*-methylbenzamide (**68**)

Methyl 4-(3-((1*H*-pyrrolo[2,3-*c*]pyridin-3-yl)methyl)-5-phenyl-1,2,4-triazin-6-yl)benzoate (**66**) (22 mg, 0.052 mmol) was transferred to a microwave vial in 0.5 mL of methanol. To this was added 2 mL of 40% methylamine in water. The reaction mixture was then heated under microwave radiation at 100 °C for 1 h. The reaction mixture was extracted twice with DCM, and the combined organics were concentrated to dryness. The resulting residues were purified by preparative HPLC (basic conditions) to give 4-(3-((1*H*-pyrrolo[2,3-*c*]pyridin-3-yl)methyl)-5-phenyl-1,2,4-triazin-6-yl)-*N*-methylbenzamide (**68**) (11 mg, 50%). Analytical RP-HPLC method C, retention time 1.35 min. ^1^H NMR (400 MHz, CD_3_OD) δ 8.65 (s, 1H), 8.06 (d, *J* = 5.8, 1H), 7.76–7.84 (m, 3H), 7.54–7.65 (m, 3H), 7.48–7.53 (m, 2H), 7.41–7.47 (m, 1H), 7.28–7.38 (m, 2H), 4.69 (s, 2H), 2.91 (s, 3H); *m*/*z*: calcd for C_25_H_21_N_6_O [M + H]^+^, 421.2; found, 421.2.

#### 4.2.84 4-(3-((1*H*-Pyrrolo[2,3-*c*]pyridin-3-yl)methyl)-6-phenyl-1,2,4-triazin-5-yl)-*N*-(2-hydroxyethyl)benzamide (**69**)

Methyl 4-(3-((1*H*-pyrrolo[2,3-*c*]pyridin-3-yl)methyl)-6-phenyl-1,2,4-triazin-5-yl)benzoate (**65**) (10 mg, 0.024 mmol) was transferred to a 2 mL microwave vial, suspended in 2-aminoethanol (100 μL, 1.66 mmol), the vial was capped, and the mixture was heated at 100 °C for 20 min resulting in a brown solution. Worked up by cooling to room temperature and diluting with methanol (0.2 mL) and DMSO (0.2 mL) and purifying directly by preparative HPLC (basic conditions) to give 4-(3-((1*H*-pyrrolo[2,3-*c*]pyridin-3-yl)methyl)-6-phenyl-1,2,4-triazin-5-yl)-*N*-(2-hydroxyethyl)benzamide (**69**) (5 mg, 50%). Analytical RP-HPLC method B, retention time 1.26 min. ^1^H NMR (400 MHz, CD_3_OD) δ 8.62–8.68 (m, 1H), 8.03–8.09 (m, 1H), 7.78 (d, *J* = 8.3, 3H), 7.61 (d, *J* = 12.3, 3H), 7.47 (d, *J* = 1.5, 3H), 7.38 (d, *J* = 7.5, 2H), 4.69 (s, 2H), 3.65–3.73 (m, 2H), 3.48 (s, 2H); *m*/*z*: calcd for C_26_H_23_N_6_O_2_ [M + H]^+^, 451.2; found, 451.2.

#### 4.2.85 4-(3-((1*H*-Pyrrolo[2,3-*c*]pyridin-3-yl)methyl)-5-phenyl-1,2,4-triazin-6-yl)-*N*-(2-hydroxyethyl)benzamide (**70**)

Methyl 4-(3-((1*H*-pyrrolo[2,3-*c*]pyridin-3-yl)methyl)-5-phenyl-1,2,4-triazin-6-yl)benzoate (**66**) (5 mg, 0.012 mmol) was transferred to a 2 mL microwave vial, suspended in 2-aminoethanol (50.0 μL, 0.828 mmol), the vial was capped, and the mixture was heated at 100 °C for 20 min. Worked up by cooling reaction mixture to room temperature diluting with methanol (0.2 mL) and DMSO (0.2 mL) and purifying directly by preparative HPLC (basic conditions) to give 4-(3-((1*H*-pyrrolo[2,3-*c*]pyridin-3-yl)methyl)-5-phenyl-1,2,4-triazin-6-yl)-*N*-(2-hydroxyethyl)benzamide (**70**) (5 mg, 99%). Analytical RP-HPLC method B, retention time 1.29 min. ^1^H NMR (400 MHz, CD_3_OD) δ 8.64–8.67 (m, 1H), 8.04–8.08 (m, 1H), 7.81–7.86 (m, 2H), 7.77–7.81 (m, 1H), 7.62 (s, 1H), 7.59 (s, 2H), 7.48–7.52 (m, 2H), 7.40–7.47 (m, 1H), 7.30–7.36 (m, 2H), 4.67–4.71 (m, 2H), 3.67–3.72 (m, 2H), 3.47–3.52 (m, 2H); *m*/*z*: calcd for C_26_H_23_N_6_O_2_ [M + H]^+^, 451.2; found, 451.2.

#### 4.2.86 Methyl 4-(3-((1*H*-Pyrrolo[3,2-*c*]pyridin-3-yl)methyl)-5-phenyl-1,2,4-triazin-6-yl)benzoate and Methyl 4-(3-((1*H*-Pyrrolo[3,2-*c*]pyridin-3-yl)methyl)-6-phenyl-1,2,4-triazin-5-yl)benzoate (**71a** and **72b**)

Prepared according to method D. The resulting residues were purified by preparative HPLC (basic conditions) to give methyl 4-(3-((1*H*-pyrrolo[3,2-*c*]pyridin-3-yl)methyl)-5-phenyl-1,2,4-triazin-6-yl)benzoate and methyl 4-(3-((1*H*-pyrrolo[3,2-*c*]pyridin-3-yl)methyl)-6-phenyl-1,2,4-triazin-5-yl)benzoate, 14 mg of which was used directly in the next step.

#### 4.2.87 Preparation of 4-(3-((1*H*-Pyrrolo[3,2-*c*]pyridin-3-yl)methyl)-6-phenyl-1,2,4-triazin-5-yl)-*N*-methylbenzamide (**71**) and 4-(3-((1*H*-Pyrrolo[3,2-*c*]pyridin-3-yl)methyl)-5-phenyl-1,2,4-triazin-6-yl)-*N*-methylbenzamide (**72**)

Methyl 4-(3-((1*H*-pyrrolo[3,2-*c*]pyridin-3-yl)methyl)-5-phenyl-1,2,4-triazin-6-yl)benzoate and methyl 4-(3-((1*H*-pyrrolo[3,2-*c*]pyridin-3-yl)methyl)-6-phenyl-1,2,4-triazin-5-yl)benzoate (14 mg, 0.033 mmol) were transferred to a microwave vial in 0.5 mL of methanol. To this was added 2 mL of 40% methylamine in water. The reaction mixture was then microwaved at 100 °C for 1 h. The reaction mixture was concentrated to dryness. The regioisomers were separated by SFC using a Daicel OD-H (10 × 250 mm^2^, 30% methanol + 0.25% diethylamine, 15 mL/min) to give **71** and **72**.

##### 4.2.87.1 4-(3-((1*H*-Pyrrolo[3,2-*c*]pyridin-3-yl)methyl)-6-phenyl-1,2,4-triazin-5-yl)-*N*-methylbenzamide (**71**)

5 mg, 64%. Analytical RP-HPLC method B, retention time 1.31 min. ^1^H NMR (400 MHz, CD_3_OD) δ 9.00–9.10 (m, 1H), 8.07–8.17 (m, 1H), 7.77–7.83 (m, 2H), 7.58 (s, 2H), 7.48–7.53 (m, 2H), 7.39–7.47 (m, 3H), 7.30–7.37 (m, 2H), 4.73 (s, 2H), 2.91 (s, 3H); *m*/*z*: calcd for C_25_H_21_N_6_O [M + H]^+^, 421.2; found, 421.2.

##### 4.2.87.2 4-(3-((1*H*-Pyrrolo[3,2-*c*]pyridin-3-yl)methyl)-5-phenyl-1,2,4-triazin-6-yl)-*N*-methylbenzamide (**72**)

4 mg, 50%. Analytical RP-HPLC method B, retention time 1.32 min. ^1^H NMR (400 MHz, CD_3_OD) δ 9.02–9.20 (m, 1H), 8.12–8.25 (m, 1H), 7.75 (d, *J* = 8.5 Hz, 2H), 7.58 (d, *J* = 8.5 Hz, 2H), 7.54 (s, 1H), 7.41–7.52 (m, 4H), 7.34–7.40 (m, 2H), 4.75 (s, 2H), 3.35 (s, 1H), 2.90 (s, 3H); *m*/*z*: calcd for C_25_H_21_N_6_O [M + H]^+^, 421.2; found, 421.2.

#### 4.2.88 4-(3-((1*H*-Indol-3-yl)methyl)-5-phenyl-1,2,4-triazin-6-yl)-*N*-methylbenzamide (**73**)

Prepared according to method G. The resulting residues were further purified by flash chromatography eluting with 50–100% ethyl acetate in heptane. The appropriate fractions were combined to give 4-(3-((1*H*-indol-3-yl)methyl)-5-phenyl-1,2,4-triazin-6-yl)-*N*-methylbenzamide (**73**) (13 mg, 45%). Analytical RP-HPLC method A, retention time 2.38 min. ^1^H NMR (400 MHz, DMSO-*d*_6_) δ 10.92–11.01 (m, 1H), 8.42–8.54 (m, 1H), 7.78–7.85 (m, 2H), 7.67–7.74 (m, 1H), 7.52–7.59 (m, 2H), 7.42–7.50 (m, 3H), 7.34 (s, 4H), 7.04–7.11 (m, 1H), 6.97–7.03 (m, 1H), 4.58 (s, 2H), 2.71–2.83 (m, 3H); *m*/*z*: calcd for C_26_H_22_N_5_O [M + H]^+^, 420.2; found, 420.0.

#### 4.2.89 (4-(3-((1*H*-Indol-3-yl)methyl)-5-phenyl-1,2,4-triazin-6-yl)phenyl)(morpholino)methanone (**74**)

Prepared according to method G. The resulting residues were purified by flash chromatography eluting with 70–100% ethyl acetate in heptane. The relevant fractions were combined to afford a solid. The solid was further purified by preparative HPLC (basic conditions) to give (4-(3-((1*H*-indol-3-yl)methyl)-5-phenyl-1,2,4-triazin-6-yl)phenyl)(morpholino)methanone (**74**) (5 mg, 50%). Analytical RP-HPLC method A, retention time 2.27 min. ^1^H NMR (400 MHz, CDCl_3_) δ 8.07–8.16 (m, 1H), 7.88–7.95 (m, 1H), 7.59 (d, *J* = 8.3, 2H), 7.47–7.54 (m, 2H), 7.31–7.45 (m, 7H), 7.16 (s, 2H), 4.72 (s, 2H), 3.55–3.87 (m, 6H), 3.29–3.51 (m, 2H); *m*/*z*: calcd for C_29_H_26_N_5_O_2_ [M + H]^+^, 476.2; found, 476.0.

#### 4.2.90 4-(3-((1*H*-Indol-3-yl)methyl)-5-phenyl-1,2,4-triazin-6-yl)-*N*-(2-(dimethylamino)ethyl)benzamide (**75**)

Prepared according to method G. The resulting residues were purified by preparative HPLC (basic conditions) to give 4-(3-((1*H*-indol-3-yl)methyl)-5-phenyl-1,2,4-triazin-6-yl)-*N*-(2-(dimethylamino)ethyl)benzamide (**75**) (4 mg, 33%). Analytical RP-HPLC method B, retention time 1.56 min. ^1^H NMR (400 MHz, CDCl_3_) δ 8.10–8.19 (m, 1H), 7.88–7.97 (m, 1H), 7.69–7.78 (m, 2H), 7.56–7.63 (m, 2H), 7.47–7.52 (m, 2H), 7.31–7.44 (m, 5H), 7.10–7.24 (m, 2H), 6.81–6.89 (m, 1H), 4.66–4.77 (m, 2H), 3.45–3.58 (m, 2H), 2.46–2.57 (m, 2H), 2.27 (s, 6H); *m*/*z*: calcd for C_29_H_29_N_6_O [M + H]^+^, 477.2; found, 477.0.

#### 4.2.91 4-(3-((1*H*-Indol-3-yl)methyl)-5-phenyl-1,2,4-triazin-6-yl)-*N*-(2-hydroxyethyl)benzamide (**76**)

Prepared according to method G. The resulting residues were purified by flash chromatography eluting with 0–5% methanol in DCM. The relevant fractions were combined and concentrated to dryness to give 4-(3-((1*H*-indol-3-yl)methyl)-5-phenyl-1,2,4-triazin-6-yl)-*N*-(2-hydroxyethyl)benzamide (**76**) (8 mg, 72%). Analytical RP-HPLC method A, retention time 2.22 min. ^1^H NMR (400 MHz, CDCl_3_) δ 8.09–8.16 (m, 1H), 7.90–7.96 (m, 1H), 7.67–7.74 (m, 2H), 7.55–7.61 (m, 2H), 7.45–7.51 (m, 2H), 7.30–7.44 (m, 5H), 7.12–7.24 (m, 2H), 6.55–6.64 (m, 1H), 4.72 (s, 2H), 3.80–3.91 (m, 2H), 3.59–3.71 (m, 2H), 2.28–2.44 (m, 1H); *m*/*z*: calcd for C_27_H_24_N_5_O_2_ [M + H]^+^, 450.2; found, 450.0.

#### 4.2.92 (4-(3-((1*H*-Indol-3-yl)methyl)-5-phenyl-1,2,4-triazin-6-yl)phenyl)(4-methylpiperazin-1-yl)methanone (**77**)

Prepared according to method G. The resulting residues were purified by flash chromatography eluting with 0–10% methanol in DCM. The relevant fractions were combined to afford a yellow solid. The solid was further purified by preparative HPLC (basic conditions) to give (4-(3-((1*H*-indol-3-yl)methyl)-5-phenyl-1,2,4-triazin-6-yl)phenyl)(4-methylpiperazin-1-yl)methanone (**77**) (4.1 mg, 10%). Analytical RP-HPLC method B, retention time 1.57 min. ^1^H NMR (400 MHz, CDCl_3_) δ 8.08–8.16 (m, 1H), 7.89–7.95 (m, 1H), 7.55–7.61 (m, 2H), 7.48–7.53 (m, 2H), 7.31–7.44 (m, 7H), 7.12–7.25 (m, 2H), 4.72 (s, 2H), 3.69–3.89 (m, 2H), 3.25–3.49 (m, 2H), 2.40–2.54 (m, 2H), 2.32 (s, 5H); *m*/*z*: calcd for C_30_H_29_N_6_O [M + H]^+^, 489.2; found, 489.0.

#### 4.2.93 4-(3-((1*H*-Indol-3-yl)methyl)-5-phenyl-1,2,4-triazin-6-yl)-*N*-(2-methoxyethyl)benzamide (**78**)

Prepared according to method G. The resulting residues were purified by flash chromatography eluting with 0–5% methanol in DCM. The relevant fractions were combined to afford 4-(3-((1*H*-indol-3-yl)methyl)-5-phenyl-1,2,4-triazin-6-yl)-*N*-(2-methoxyethyl)benzamide (**78**) (4 mg, 36%). Analytical RP-HPLC method B, retention time 2.24 min. ^1^H NMR (400 MHz, CDCl_3_) δ 8.07–8.14 (m, 1H), 7.91–7.96 (m, 1H), 7.70–7.75 (m, 2H), 7.57–7.62 (m, 2H), 7.46–7.52 (m, 2H), 7.31–7.45 (m, 5H), 7.12–7.25 (m, 2H), 6.46–6.54 (m, 1H), 4.72 (s, 2H), 3.63–3.69 (m, 2H), 3.54–3.59 (m, 2H), 3.40 (s, 3H); *m*/*z*: calcd for C_28_H_26_N_5_O_2_ [M + H]^+^, 464.2; found, 464.0.

#### 4.2.94 4-(3-((1*H*-Indol-3-yl)methyl)-5-phenyl-1,2,4-triazin-6-yl)-*N*-(2-acetamidoethyl)benzamide (**79**)

Prepared according to method G. The resulting residues were purified by preparative HPLC (basic conditions) to give 4-(3-((1*H*-indol-3-yl)methyl)-5-phenyl-1,2,4-triazin-6-yl)-*N*-(2-acetamidoethyl)benzamide (**79**) (4 mg, 14%). Analytical RP-HPLC method B, retention time 2.02 min. ^1^H NMR (400 MHz, CDCl_3_) δ 8.15–8.23 (m, 1H), 7.89–7.95 (m, 1H), 7.71–7.78 (m, 2H), 7.56–7.62 (m, 2H), 7.45–7.51 (m, 2H), 7.30–7.45 (m, 6H), 7.12–7.23 (m, 2H), 6.11–6.25 (m, 1H), 4.71 (s, 2H), 3.57 (dd, *J* = 6.4, 4.6 Hz, 2H), 3.46–3.54 (m, 2H), 2.01 (s, 3H); *m*/*z*: calcd for C_29_H_26_N_6_O_2_ [M + H]^+^, 491.2; found, 491.0.

#### 4.2.95 4-(4-(3-((1*H*-Indol-3-yl)methyl)-5-phenyl-1,2,4-triazin-6-yl)benzoyl)-1-methylpiperazin-2-one (**80**)

Prepared according to method G. The resulting residue was purified by flash chromatography eluting with 0–5% methanol in DCM to give 4-(4-(3-((1*H*-indol-3-yl)methyl)-5-phenyl-1,2,4-triazin-6-yl)benzoyl)-1-methylpiperazin-2-one (**80**) (5 mg, 42%). Analytical RP-HPLC method B, retention time 2.07 min. ^1^H NMR (400 MHz, CDCl_3_) δ 8.08–8.15 (m, 1H), 7.90–7.95 (m, 1H), 7.58–7.63 (m, 2H), 7.48–7.53 (m, 2H), 7.32–7.45 (m, 7H), 7.13–7.24 (m, 2H), 4.72 (s, 2H), 3.86–4.29 (m, 4H), 3.34–3.50 (m, 2H), 3.02 (s, 3H); *m*/*z*: calcd for C_30_H_27_N_6_O_2_ [M + H]^+^, 503.2; found, 503.0.

#### 4.2.96 4-(3-((1*H*-Indol-3-yl)methyl)-5-phenyl-1,2,4-triazin-6-yl)-*N*-(2-amino-2-oxoethyl)benzamide (**81**)

Prepared according to method G. The resulting residues were purified by preparative HPLC (basic conditions) to give 4-(3-((1*H*-indol-3-yl)methyl)-5-phenyl-1,2,4-triazin-6-yl)-*N*-(2-amino-2-oxoethyl)benzamide (**81**) (2 mg, 18%). Analytical RP-HPLC method B, retention time 1.95 min. ^1^H NMR (400 MHz, CDCl_3_) δ 8.07–8.13 (m, 1H), 7.91–7.96 (m, 1H), 7.78–7.79 (m, 1H), 7.76 (d, *J* = 8.3, 2H), 7.58–7.63 (m, 2H), 7.46–7.51 (m, 2H), 7.32–7.44 (m, 4H), 7.13–7.24 (m, 2H), 6.87–6.93 (m, 1H), 5.80–5.89 (m, 1H), 5.42–5.51 (m, 1H), 4.72 (s, 2H), 4.15–4.19 (m, 2H); *m*/*z*: calcd for C_27_H_22_N_6_O_2_ [M + H]^+^, 463.2; found, 463.0.

#### 4.2.97 4-(3-((1*H*-Indol-3-yl)methyl)-6-phenyl-1,2,4-triazin-5-yl)-*N*-methylbenzamide (**82**)

Prepared according to method G. The resulting residues were purified by flash chromatography using 0–5% methanol in DCM. The relevant fractions were combined and concentrated to dryness. The resulting residues were further purified by flash chromatography and eluting with 50–100% ethyl acetate in heptane. The relevant fractions were combined to give 4-(3-((1*H*-indol-3-yl)methyl)-6-phenyl-1,2,4-triazin-5-yl)-*N*-methylbenzamide (**82**) (10 mg, 34%). Analytical RP-HPLC method A, retention time 2.34 min. ^1^H NMR (400 MHz, DMSO-*d*_6_) δ 10.94–11.01 (m, 1H), 8.44–8.55 (m, 1H), 7.77 (s, 2H), 7.70–7.75 (m, 1H), 7.50–7.56 (m, 2H), 7.32–7.49 (m, 7H), 7.05–7.11 (m, 1H), 6.97–7.03 (m, 1H), 4.54–4.63 (m, 2H), 2.72–2.80 (m, 3H); *m*/*z*: calcd for C_26_H_22_N_5_O [M + H]^+^, 420.2; found, 420.0.

#### 4.2.98 (4-(3-((1*H*-Indol-3-yl)methyl)-6-phenyl-1,2,4-triazin-5-yl)phenyl)(morpholino)methanone (**83**)

Prepared according to method G. The resulting residue was purified by preparative HPLC (basic conditions) to give (4-(3-((1*H*-indol-3-yl)methyl)-6-phenyl-1,2,4-triazin-5-yl)phenyl)(morpholino)methanone (**83**) (6 mg, 50%). Analytical RP-HPLC method B, retention time 2.28 min. ^1^H NMR (400 MHz, CDCl_3_) δ 8.09–8.17 (m, 1H), 7.91–7.96 (m, 1H), 7.55–7.61 (m, 2H), 7.49–7.55 (m, 2H), 7.30–7.47 (m, 7H), 7.12–7.24 (m, 2H), 4.72 (s, 2H), 3.33–3.87 (m, 8H); *m*/*z*: calcd for C_29_H_26_N_5_O_2_ [M + H]^+^, 476.2; found, 476.0.

#### 4.2.99 4-(3-((1*H*-Indol-3-yl)methyl)-6-phenyl-1,2,4-triazin-5-yl)-*N*-(2-(dimethylamino)ethyl)benzamide (**84**)

Prepared according to method G. The resulting residues were purified by preparative HPLC (basic conditions) to give 4-(3-((1*H*-indol-3-yl)methyl)-6-phenyl-1,2,4-triazin-5-yl)-*N*-(2-(dimethylamino)ethyl)benzamide (**84**) (4 mg, 33%). Analytical RP-HPLC method B, retention time 1.67 min. ^1^H NMR (400 MHz, CDCl_3_) δ 8.08–8.17 (m, 1H), 7.91–7.98 (m, 1H), 7.78 (d, *J* = 8.3, 2H), 7.59 (d, *J* = 8.3, 2H), 7.47–7.54 (m, 2H), 7.29–7.46 (m, 5H), 7.13–7.24 (m, 2H), 6.81–6.92 (m, 1H), 4.71 (s, 2H), 3.48–3.59 (m, 2H), 2.48–2.57 (m, 2H), 2.19–2.36 (m, 6H); *m*/*z*: calcd for C_29_H_29_N_6_O [M + H]^+^, 477.2; found, 477.0.

#### 4.2.100 4-(3-((1*H*-Indol-3-yl)methyl)-6-phenyl-1,2,4-triazin-5-yl)-*N*-(2-hydroxyethyl)benzamide (**85**)

Prepared according to method G. The resulting residue was purified by preparative HPLC (basic conditions) to give 4-(3-((1*H*-indol-3-yl)methyl)-6-phenyl-1,2,4-triazin-5-yl)-*N*-(2-hydroxyethyl)benzamide (**85**) (6 mg, 55%). Analytical RP-HPLC method B, retention time 2.08 min. ^1^H NMR (400 MHz, CDCl_3_) δ 8.12–8.18 (m, 1H), 7.90–7.95 (m, 1H), 7.70–7.77 (m, 2H), 7.52–7.58 (m, 2H), 7.46–7.52 (m, 2H), 7.39–7.45 (m, 1H), 7.29–7.39 (m, 4H), 7.12–7.24 (m, 2H), 6.69–6.76 (m, 1H), 4.71 (s, 2H), 3.76–3.88 (m, 2H), 3.56–3.69 (m, 2H), 2.45–2.75 (m, 1H); *m*/*z*: calcd for C_27_H_24_N_5_O_2_ [M + H]^+^, 450.2; found, 450.0.

#### 4.2.101 (4-(3-((1*H*-Indol-3-yl)methyl)-6-phenyl-1,2,4-triazin-5-yl)phenyl)(4-methylpiperazin-1-yl)methanone (**86**)

Prepared according to method G. The resulting residues were purified by preparative HPLC (basic conditions) to give (4-(3-((1*H*-indol-3-yl)methyl)-6-phenyl-1,2,4-triazin-5-yl)phenyl)(4-methylpiperazin-1-yl)methanone (**86**) (5 mg, 42%). Analytical RP-HPLC method B, retention time 1.65 min. ^1^H NMR (400 MHz, CDCl_3_) δ 8.09–8.17 (m, 1H), 7.91–7.97 (m, 1H), 7.55–7.60 (m, 2H), 7.50–7.55 (m, 2H), 7.30–7.47 (m, 7H), 7.12–7.24 (m, 2H), 4.71 (s, 2H), 3.69–3.89 (m, 2H), 3.30–3.51 (m, 2H), 2.40–2.56 (m, 2H), 2.32 (s, 5H); *m*/*z*: calcd for C_30_H_29_N_6_O [M + H]^+^, 489.2; found, 489.0.

#### 4.2.102 4-(3-((1*H*-Indol-3-yl)methyl)-6-phenyl-1,2,4-triazin-5-yl)-*N*-(2-methoxyethyl)benzamide (**87**)

Prepared according to method G. The resulting residues were purified by preparative HPLC (basic conditions) to give 4-(3-((1*H*-indol-3-yl)methyl)-6-phenyl-1,2,4-triazin-5-yl)-*N*-(2-methoxyethyl)benzamide (**87**) (5 mg, 46%). Analytical RP-HPLC method B, retention time 2.28 min. ^1^H NMR (400 MHz, CDCl_3_) δ 8.08–8.16 (m, 1H), 7.91–7.96 (m, 1H), 7.73–7.79 (m, 2H), 7.57–7.62 (m, 2H), 7.47–7.53 (m, 2H), 7.40–7.46 (m, 1H), 7.30–7.39 (m, 4H), 7.13–7.24 (m, 2H), 6.49–6.57 (m, 1H), 4.64–4.76 (m, 2H), 3.63–3.69 (m, 2H), 3.54–3.60 (m, 2H), 3.40 (s, 3H); *m*/*z*: calcd for C_28_H_26_N_5_O_2_ [M + H]^+^, 464.2; found, 464.0.

#### 4.2.103 4-(3-((1*H*-Indol-3-yl)methyl)-6-phenyl-1,2,4-triazin-5-yl)-*N*-(2-acetamidoethyl)benzamide (**88**)

Prepared according to method G. The resulting residues were purified by preparative HPLC (basic conditions) to give 4-(3-((1*H*-indol-3-yl)methyl)-6-phenyl-1,2,4-triazin-5-yl)-*N*-(2-acetamidoethyl)benzamide (**88**) (5 mg, 42%). Analytical RP-HPLC method B, retention time 2.08 min. ^1^H NMR (400 MHz, CDCl_3_) δ 8.10–8.17 (m, 1H), 7.91–7.97 (m, 1H), 7.75–7.82 (m, 2H), 7.56–7.62 (m, 2H), 7.47–7.53 (m, 2H), 7.40–7.45 (m, 1H), 7.29–7.39 (m, 5H), 7.11–7.24 (m, 2H), 6.15–6.23 (m, 1H), 4.71 (s, 2H), 3.55–3.61 (m, 2H), 3.47–3.54 (m, 2H), 2.01 (s, 3H); *m*/*z*: calcd for C_29_H_26_N_6_O_2_ [M + H]^+^, 491.2; found, 491.0.

#### 4.2.104 4-(4-(3-((1*H*-Indol-3-yl)methyl)-6-phenyl-1,2,4-triazin-5-yl)benzoyl)-1-methylpiperazin-2-one (**89**)

Prepared according to method G. The resulting residues were purified by preparative HPLC (basic conditions) to give 4-(4-(3-((1*H*-indol-3-yl)methyl)-6-phenyl-1,2,4-triazin-5-yl)benzoyl)-1-methylpiperazin-2-one (**89**) (4 mg, 33%). Analytical RP-HPLC method B, retention time 2.13 min. ^1^H NMR (400 MHz, CDCl_3_) δ 8.08–8.15 (m, 1H), 7.91–7.97 (m, 1H), 7.57–7.63 (m, 2H), 7.48–7.55 (m, 2H), 7.31–7.47 (m, 7H), 7.12–7.24 (m, 2H), 4.72 (s, 2H), 3.85–4.38 (m, 4H), 3.33–3.50 (m, 2H), 3.02 (s, 3H); *m*/*z*: calcd for C_30_H_27_N_6_O_2_ [M + H]^+^, 503.2; found, 503.0.

#### 4.2.105 4-(3-((1*H*-Indol-3-yl)methyl)-6-phenyl-1,2,4-triazin-5-yl)-*N*-(2-amino-2-oxoethyl)benzamide (**90**)

Prepared according to method G. The resulting residues were purified by preparative HPLC (basic conditions) to give 4-(3-((1*H*-indol-3-yl)methyl)-6-phenyl-1,2,4-triazin-5-yl)-*N*-(2-amino-2-oxoethyl)benzamide (**90**) (3 mg, 25%). Analytical RP-HPLC method B, retention time 2.02 min. ^1^H NMR (400 MHz, CDCl_3_) δ 8.08–8.15 (m, 1H), 7.91–7.97 (m, 1H), 7.77–7.83 (m, 2H), 7.58–7.64 (m, 2H), 7.47–7.54 (m, 2H), 7.41–7.46 (m, 1H), 7.30–7.40 (m, 4H), 7.12–7.24 (m, 2H), 6.91–6.98 (m, 1H), 5.89–6.00 (m, 1H), 5.42–5.53 (m, 1H), 4.72 (s, 2H), 4.17 (d, *J* = 5.0 Hz, 2H); *m*/*z*: calcd for C_27_H_22_N_6_O_2_ [M + H]^+^, 463.2; found, 463.0.

### 4.3 Assay

#### 4.3.1 [^35^S]GTPγS Incorporation Assay

The GTPγS functional assay was performed according to the published procedures.^23^ A brief description of the binding assay is given below. Flp-ln TREx 293 cells (lnvitrogen) were maintained in Dulbecco’s modified Eagle’s medium without sodium pyruvate (lnvitrogen), supplemented with 10% (w/v) fetal calf serum, 1% penicillin/streptomycin mixture, and 10 μg/mL blasticidin at 37 °C in a 5% CO_2_ humidified atmosphere. To generate Flp-ln TREx 293 cells capable of inducibly expressing the GPR84 receptor constructs, the cells were transfected with a mixture containing the desired cDNA in pcDNA5/FRT/TO vector and pOG44 vector (1:9) using 1 mg/mL polyethylenimine (PEI) (molecular weight (MW)—25 000). Cells were grown until 60–80% confluent then transfected with 8 μg of required plasmid DNA and PEI (ratio 1:6 DNA/PEl) and diluted in 150 mM NaCl, pH 7.4. After incubation at room temperature for 10 min, the mixture was added to cells. After 48 h, the medium was changed to a medium supplemented with 200 μg/mL hygromycin B to initiate the selection of stably transfect cells. After isolation of resistant cells, expression of the appropriate construct from the Flp-ln TREx locus was induced by treatment with up to 100 ng/mL doxycycline for 24 h. Membrane proteins were generated from Flp-In T-REx HEK293 cells treated with 100 ng/mL doxycycline to induce expression of the receptor construct of interest. The cells were washed with ice-cold phosphate-buffered saline, removed from dishes by scraping, and centrifuged at 3000 rpm for 5 min at 4 °C. Pellets were resuspended in TE buffer (10 mM Tris–HCl, 0.1 mM ethylenediaminetetraacetic acid (EDTA); pH 7.5) containing a protease inhibitor mixture (Roche Applied Science, West Sussex, U.K.) and homogenized with a 5 mL hand-held homogenizer. This material was centrifuged at 1500 rpm for 5 min at 4 °C, and the supernatant was further centrifuged at 50 000 rpm for 45 min at 4 °C. The resulting pellet was resuspended in TE buffer, and the protein content was assessed using a bicinchoninic acid (BCA) protein assay kit (Pierce, Fisher Scientific, Loughborough, U.K.). The following assay can be used for the determination of GPR84 activation. The guanosine 5′-*O*-[γ-thio]triphosphate ([^35^S]GTPγS) functional assay measures the level of G protein activation following agonist occupation of a GPCR, by determining the binding of the poorly hydrolyzable analogue [^35^S]GTPγS to Gα subunits. Initially, 5 μg of generated membrane protein was preincubated for 15 min at 25 °C in assay buffer (20 mM *N*-(2-hydroxyethyl)piperazine-*N*′-ethanesulfonic acid (HEPES), 5 mM MgCl_2_; 160 mM NaCl; 1 μM GDP; 0.05% fatty acid-free bovine serum albumin; pH 7.5) containing the indicated ligand agonist concentrations. To assess inhibition of agonist stimulation, membrane preparations were preincubated with antagonist compound for 15 min at room temperature prior to the addition of agonist. The reaction was then initiated with the addition of [^35^S]GTPγS (50 nCi per tube), and the reaction was terminated after 45 min incubation at 30 °C by rapid filtration through GF/C glass filters using a 24-well Brandel cell harvester (Alpha Biotech, Glasgow, U.K.). Unbound radioligand was removed from filters by three washes with ice-cold phosphate-buffered saline (pH 7.4), and filters were dried for 2–3 h at room temperature. The dried filters were added to 3 mL of Ultima GoldTM XR (PerkinElmer Life Sciences, Beaconsfield, U.K.), and [^35^S]GTPγS binding was determined by liquid scintillation spectrometry

### 4.4 Molecular Docking

The obtained antagonists were docked into the AlphaFold model of the human GPR84^31,32^ using a standard precision docking protocol available in the Glide module of Schrodinger software (2020-1).^38,39^ The Alphafold structure of the receptor was obtained from the AlphaFold protein structure database (https://alphafold.ebi.ac.uk/). The protein structures were prepared with the protein preparation module, and the structure of antagonists was assessed with the ligand preparation module of Schrodinger software. Residues involving Tyr^69^, Phe^101^, Arg^172^, Phe^335^, and Trp^360^ were selected to center the docking box. Receptor docking grids with the receptor van der Waals radius scaling of 1.0, 0.9, and 0.8 were generated to probe the binding of bulky compound analogues. Docking poses were evaluated with the Glide docking score. The most frequent docking mode among compound analogues was selected as the most probable pose. Short minimization of docking complexes was carried out with the MacroModel module of Schrodinger software. A default protocol of minimization in implicit solvent was used to obtain the final complexes. The OPLS_2005 force field was used in all calculations. The three-dimensional (3D) images were created in Maestro 2020-1.

### 4.5 Method for In Vivo Pharmacokinetic Studies

Mouse pharmacokinetic studies were carried out by Xenogesis Ltd. (Nottingham, U.K.). All life phases were conducted in accordance with the local Ethics Review process.

#### 4.5.1 IV Dosing

Male C57BL/6J mice (*n* = 3, Charles River, U.K.) were intravenously administered with a dose of 1 mg/kg of the appropriate compound (formulation 5% v/v DMSO in 20% w/v HPCD in water). Blood was taken at 0.033, 0.1, 0.17, 0.25, 0.5, 1, 2, 4, 6, 8, 12, and 24 h after dosing. Plasma concentrations were determined by LC/MS/MS. LC/MS/MS analysis was performed using a Thermo TSQ Quantiva with Thermo Vanquish UPLC system on a Luna Omega C18 column. The plasma concentrations were simulated by nonparametric analysis from the plasma concentrations obtained in the PK study using Phoenix WinNonlin.

#### 4.5.2 PO Dosing

Male C57BL/6J mice (*n* = 3, Charles River, U.K.) were orally administered with a dose of 10 mg/kg of the appropriate compound (formulation 0.5% w/v methylcellulose in water). Blood was taken at pre-dose, 0.25, 0.5, 1, 2, 4, 6, 8, 12, and 24 h after dosing. Plasma concentrations were determined by LC/MS/MS. LC/MS/MS analysis was performed using a Thermo TSQ Quantiva with Thermo Vanquish UPLC system on a Luna Omega C18 column. The plasma concentrations were simulated by nonparametric analysis from the plasma concentrations obtained in the PK study using Phoenix WinNonlin.

## Supporting Information Available

Experimental procedures and characterization data for compounds; copies of ^1^H NMR spectra and LCMS for all final compounds (PDF)
Molecular formula strings and biological data (CSV)
GPR84-cmp1 complex; lack of activity of the compound series at mouse GPR84 (PDB)

## Supplementary Material

jm2c00804_si_001.csv

jm2c00804_si_002.pdb

## Abbreviations Used

6-OAU: 6-octylaminouracil
Clog *P*: logarithm of the partition coefficient for *n*-octanol/water
DIM: diindolylmethane
DMPK: drug metabolism and pharmacokinetics
GPR84: G-protein-coupled receptor 84
GTPγS: guanosine 5′-*O*-[γ-thio]triphosphate
HTS: high-throughput screen
IV: intravenous
LLE: ligand lipophilic efficiency
MCFAs: medium-chain free fatty acids
pIC_50_: negative log of the IC_50_ in molar
PPB: plasma protein binding
PO: per os
QSAR: quantitative structure–activity relationship
RP-HPLC: reversed-phase high-performance liquid chromatography
SAR: structure–activity relationship
SFC: supercritical fluid chromatography
