# Supplementary material for: Investigating the Structure–Activity Relationship of 1,2,4-Triazine G-Protein-Coupled Receptor 84 (GPR84) Antagonists
Source: J Med Chem. 2022 Aug 10;65(16):11270–90. doi: 10.1021/acs.jmedchem.2c00804 (PMC9421653; doi:10.1021/acs.jmedchem.2c00804)
Supplement: Supplementary file 3 — jm2c00804_si_003.pdf [file jm2c00804_si_003.pdf]

Supporting Information

Investigating the Structure-Activity-Relationship of 1,2,4-Triazine G Protein-Coupled Receptor 84 (GPR84) Antagonists

Amit Mahindra,[†] Laura Jenkins,[‡] Sara Marsango,[‡] Mark Huggett,[§#] Margaret Huggett,[§#] Lindsay Robinson,[§#] Jonathan Gillespie,[§#] Muralikrishnan Rajamanickam,[§#] Angus Morrison,[§#] Stuart McElroy,[§#] Irina G. Tikhonova,[$] Graeme Milligan[‡*] and Andrew G. Jamieson[†*]

[†]School of Chemistry, University of Glasgow, Joseph Black Building, University Avenue, Glasgow, G12 8QQ, U.K.

[‡]Centre for Translational Pharmacology, Institute of Molecular, Cell and Systems Biology, Davidson Building, University of Glasgow, Glasgow, G12 8QQ, U.K.

[§]BioAscent Discovery Ltd., Newhouse, Lanarkshire, ML1 5UH, U.K.

[#]University of Dundee, European Screening Centre, Newhouse, Lanarkshire ML1 5UH, U.K.

[$]School of Pharmacy, Medical Biology Centre, Queen's University Belfast, Belfast, BT9 7BL, U.K.

Corresponding Authors: * Andrew G. Jamieson - Email: andrew.jamieson.2@glasgow.ac.uk

* Graeme Milligan - Email: graeme.milligan@glasgow.ac.uk

Contents

I. DMPK S-9

II NMR and LC-MS spectra S-11

^{1}H NMR spectra for compound 1 .... S-11

LC-MS spectra for compound 1 .... S-12

^{1}H NMR spectra for compound 4 .... S-13

LC-MS spectra for compound 4 .... S-13

^{1}H NMR spectra for compound 5 .... S-14

LC-MS spectra for compound 5 .... S-15

^{1}H NMR spectra for compound 6 .... S-16

LCMS spectra for compound 6....S-16
^{1}H NMR spectra for compound 7....S-17
LCMS spectra for compound 7....S-18
^{1}H NMR spectra for compound 8....S-18
LCMS spectra for compound 8....S-19
^{1}H NMR spectra for compound 9....S-20
LCMS spectra for compound 9....S-21
^{1}H NMR spectra for compound 10....S-22
LCMS spectra for compound 10....S-23
^{1}H NMR spectra for compound 11....S-24
LCMS spectra for compound 11....S-24
^{1}H NMR spectra for compound 12....S-25
LCMS spectra for compound 12....S-25
^{1}H NMR spectra for compound 13....S-26
LCMS spectra for compound 13....S-27
^{1}H NMR spectra for compound 14....S-28
LCMS spectra for compound 14....S-28
^{1}H NMR spectra for compound 15....S29
LCMS spectra for compound 15....S-29
^{1}H NMR spectra for compound 16....S-30
LCMS spectra for compound 16....S-31
^{1}H NMR spectra for compound 17....S-32
LCMS spectra for compound 17....S-32
^{1}H spectra for compound 18....S-33
LCMS spectra for compound 18....S-33
^{1}H spectra for compound 19....S-34
LCMS spectra for compound 19....S-34

^{1}H spectra for compound 20 .... S-35
LCMS spectra for compound 20 .... S-36
^{1}H spectra for compound 21 .... S-37
LCMS spectra for compound 21 .... S-38
^{1}H spectra for compound 22 .... S-39
LCMS spectra for compound 22 .... S-40
^{1}H spectra for compound 23 .... S-41
LCMS spectra for compound 23 .... S-42
^{1}H spectra for compound 24 .... S-43
LCMS spectra for compound 24 .... S-44
^{1}H spectra for compound 25 .... S-45
LCMS spectra for compound 25 .... S-45
^{1}H spectra for compound 26 .... S-46
LCMS spectra for compound 26 .... S-47
^{1}H spectra for compound 27 .... S-48
LCMS spectra for compound 27 .... S-49
^{1}H NMR spectra for compound 28 .... S-50
LC-MS spectra for compound 28 .... S-50
^{1}H NMR spectra for compound 29 .... S-51
LC-MS spectra for compound 29 .... S-52
^{1}H NMR spectra for compound 30 .... S-53
LCMS spectra for compound 30 .... S-54
^{1}H NMR spectra for compound 31 .... S-55
LCMS spectra for compound 31 .... S-56
^{1}H NMR spectra for compound 32 .... S-57
LCMS spectra for compound 32 .... S-58
^{1}H NMR spectra for compound 33 .... S-59

LCMS spectra for compound 33 .... S-60
^{1}H NMR spectra for compound 34 .... S-61
LCMS spectra for compound 34 .... S-61
^{1}H NMR spectra for compound 35 .... S-62
LCMS spectra for compound 35 .... S-63
^{1}H NMR spectra for compound 36 .... S-64
LCMS spectra for compound 36 .... S-64
^{1}H NMR spectra for compound 37 .... S-65
LCMS spectra for compound 37 .... S-66
^{1}H NMR spectra for compound 38 .... S-67
LCMS spectra for compound 38 .... S-68
^{1}H NMR spectra for compound 39 .... S-69
LCMS spectra for compound 39 .... S-70
^{1}H NMR spectra for compound 40 .... S-71
LCMS spectra for compound 40 .... S-72
^{1}H NMR spectra for compound 41 .... S-73
LCMS spectra for compound 41 .... S-74
^{1}H NMR spectra for compound 42 .... S-75
LCMS spectra for compound 42 .... S-76
^{1}H NMR spectra for compound 43 .... S-77
LCMS spectra for compound 43 .... S-78
^{1}H spectra for compound 44 .... S-79
LCMS spectra for compound 44 .... S-80
^{1}H spectra for compound 45 .... S-81
LCMS spectra for compound 45 .... S-82
^{1}H spectra for compound 46 .... S-83
LCMS spectra for compound 46 .... S-84

^{1}H spectra for compound 47 .... S-85
LCMS spectra for compound 47 .... S-86
^{1}H spectra for compound 48 .... S-87
LCMS spectra for compound 48 .... S-88
^{1}H spectra for compound 49 .... S-89
LCMS spectra for compound 49 .... S-90
^{1}H spectra for compound 50 .... S-91
LCMS spectra for compound 50 .... S-92
^{1}H spectra for compound 51 .... S-93
LCMS spectra for compound 51 .... S-94
^{1}H spectra for compound 52 .... S-95
LCMS spectra for compound 52 .... S-96
^{1}H spectra for compound 53 .... S-97
LCMS spectra for compound 53 .... S-98
^{1}H spectra for compound 54 .... S-99
LCMS spectra for compound 54 .... S-100
^{1}H spectra for compound 55 .... S-101
LCMS spectra for compound 55 .... S-102
^{1}H spectra for compound 56 .... S-103
LCMS spectra for compound 56 .... S-104
^{1}H spectra for compound 57 .... S-105
LCMS spectra for compound 57 .... S-106
^{1}H spectra for compound 58 .... S-107
LCMS spectra for compound 58 .... S-108
^{1}H spectra for compound 59 .... S-109
LCMS spectra for compound 59 .... S-109
^{1}H spectra for compound 60 .... S-110

LCMS spectra for compound 60 .... S-111
^{1}H spectra for compound 61 .... S-112
LCMS spectra for compound 61 .... S-113
^{1}H spectra for compound 62 .... S-114
LCMS spectra for compound 62 .... S-115
^{1}H spectra for compound 63 .... S-116
LCMS spectra for compound 63 .... S-117
^{1}H spectra for compound 64 .... S-118
LCMS spectra for compound 64 .... S-119
^{1}H spectra for compound 65 .... S-120
LCMS spectra for compound 65 .... S-121
^{1}H spectra for compound 66 .... S-122
LCMS spectra for compound 66 .... S-123
^{1}H spectra for compound 67 .... S-124
LCMS spectra for compound 67 .... S-125
^{1}H spectra for compound 68 .... S-126
LCMS spectra for compound 68 .... S-127
^{1}H spectra for compound 69 .... S-128
LCMS spectra for compound 69 .... S-129
^{1}H spectra for compound 70 .... S-130
LCMS spectra for compound 70 .... S-131
^{1}H spectra for compound 71 .... S-132
LCMS spectra for compound 71 .... S-133
^{1}H spectra for compound 72 .... S-134
LCMS spectra for compound 72 .... S-135
^{1}H spectra for compound 73 .... S-136
LCMS spectra for compound 73 .... S-137

^{1}H spectra for compound 74 .... S-138
LCMS spectra for compound 74 .... S-139
^{1}H NMR spectra for compound 75 .... S-140
LC-MS spectra for compound 75 .... S-141
^{1}H NMR spectra for compound 76 .... S-142
LC-MS spectra for compound 76 .... S-143
^{1}H NMR spectra for compound 77 .... S-144
LC-MS spectra for compound 77 .... S-145
^{1}H NMR spectra for compound 78 .... S-146
LCMS spectra for compound 78 .... S-147
^{1}H NMR spectra for compound 79 .... S-148
LCMS spectra for compound 79 .... S-149
^{1}H NMR spectra for compound 80 .... S-150
LCMS spectra for compound 80 .... S-151
^{1}H NMR spectra for compound 81 .... S-152
LCMS spectra for compound 81 .... S-153
^{1}H NMR spectra for compound 82 .... S-154
LCMS spectra for compound 82 .... S-155
^{1}H NMR spectra for compound 83 .... S-156
LCMS spectra for compound 83 .... S-157
^{1}H NMR spectra for compound 84 .... S-158
LCMS spectra for compound 84 .... S-159
^{1}H NMR spectra for compound 85 .... S-160
LCMS spectra for compound 85 .... S-161
^{1}H NMR spectra for compound 86 .... S-162
LCMS spectra for compound 86 .... S-163
^{1}H NMR spectra for compound 87 .... S-164

LCMS spectra for compound 87 .... S-165
^{1}H NMR spectra for compound 88 .... S-166
LCMS spectra for compound 88 .... S-167
^{1}H NMR spectra for compound 89 .... S-168
LCMS spectra for compound 89 .... S-169
^{1}H NMR spectra for compound 90 .... S-170
LCMS spectra for compound 90 .... S-171
III. Antagonist activity at mouse GPR84 S-172
IV. PDB file of the GPR84-cmp1 complex S-173

I. DMPK

Kinetic aqueous solubility in PBS pH7.4 in 2% DMSO by LC-MS

Kinetic Solubility (µM)			
**Compound**	**R=1**	**R=2**	**Mean**
**4**	1.5	1.3	1.4
Diclofenac	>200	>200	>200
DPI	25	28	27
Ketoconazole	101	105	103

DPI = 4,5-Diphenylimidazole

Kinetic aqueous solubility in PBS pH7.4 in 2% DMSO by plate reader

Kinetic Solubility (µM)			
**Compound**	**R=1**	**R=2**	**Mean**
**18**	72	71	71
**42**	12	16	14
**68**	149	143	146
**76**	59	62	61
Diclofenac	>200	>200	>200
DPI	28	32	30
Ketoconazole	128	129	129

Compound 42 in Mouse (C57BL/6)

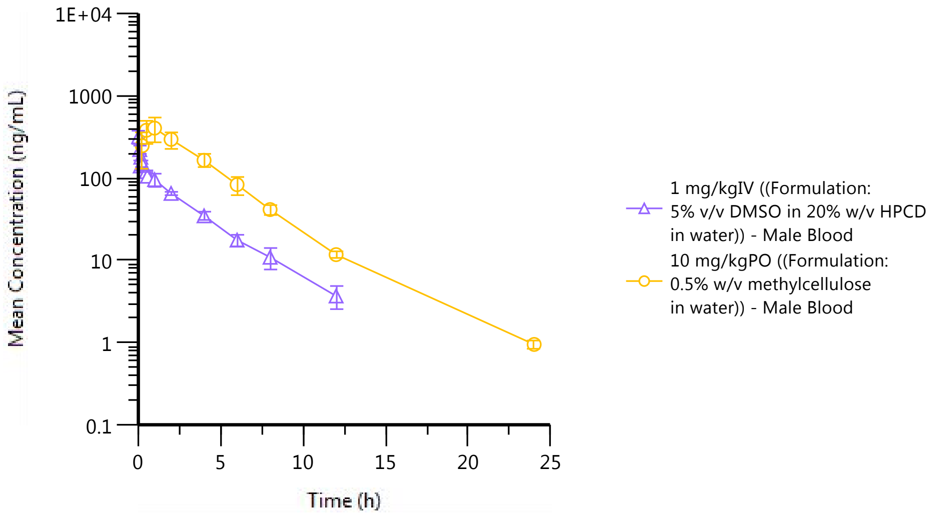

Compound 76 in Mouse (C57BL/6)

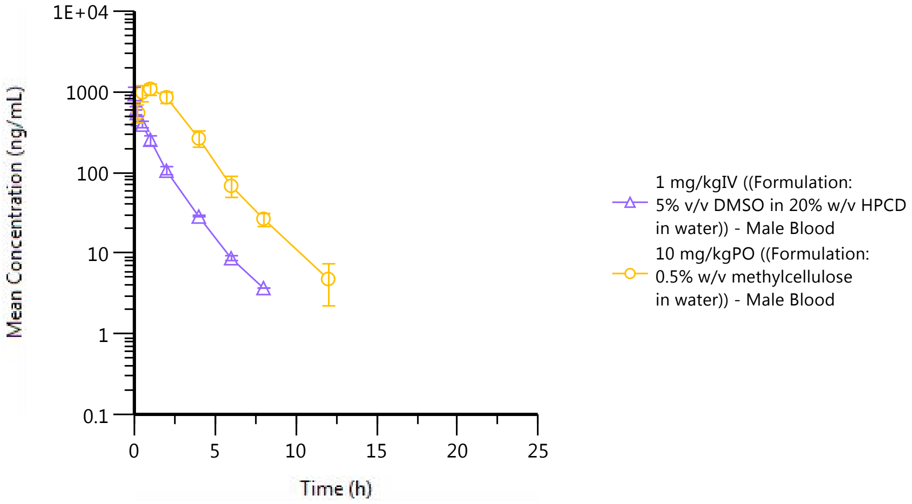

II NMR and LC-MS spectra

^{1}H NMR spectra for compound 1

^{1}H NMR (400 MHz, CHLOROFORM-*d*) δ ppm 3.85 (d, *J*=1.25 Hz, 7 H) 4.70 (s, 2 H) 6.79 - 6.95 (m, 4 H) 7.12 - 7.26 (m, 2 H) 7.33 - 7.42 (m, 2 H) 7.47 - 7.54 (m, 2 H) 7.55 - 7.63 (m, 2 H) 7.90 - 8.01 (m, 1 H) 8.07 - 8.21 (m, 1 H)

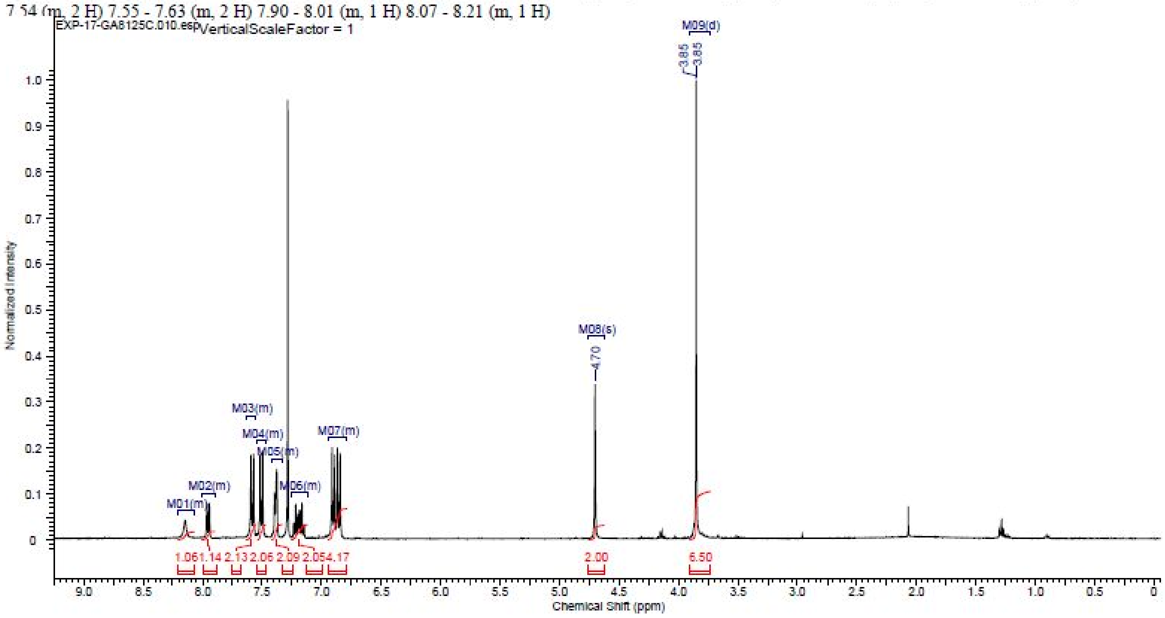

LC-MS spectra for compound 1

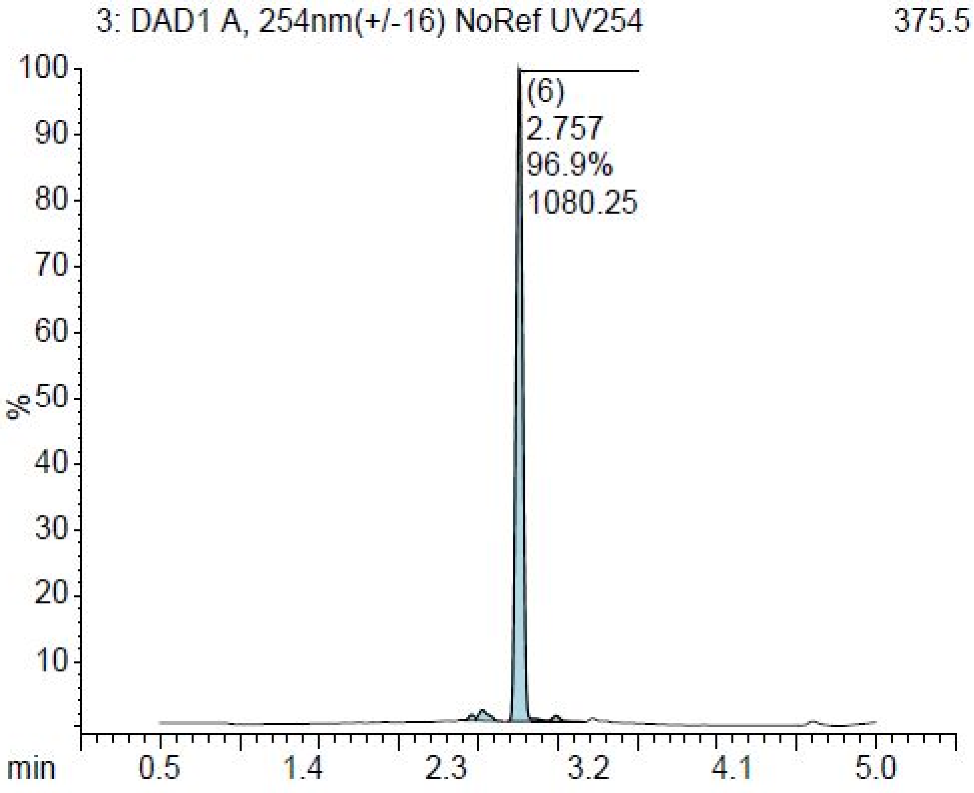

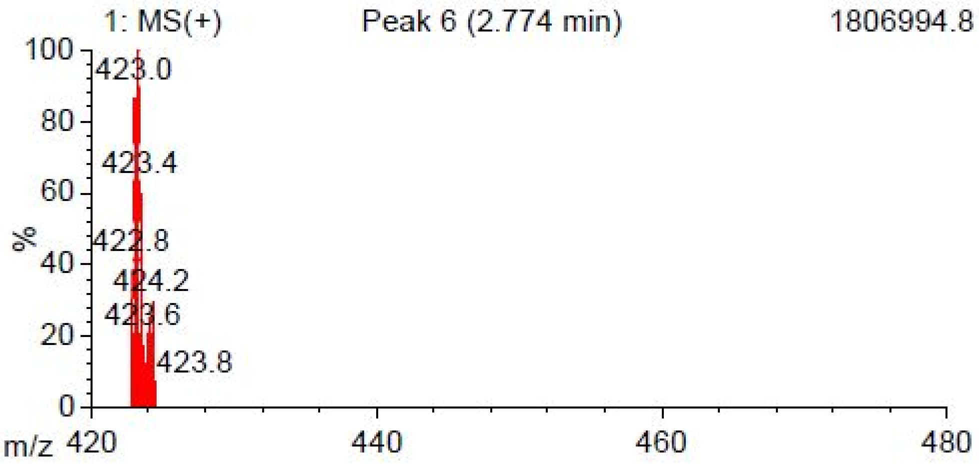

^{1}H NMR spectra for compound 4

1H NMR (400 MHz, CHLOROFORM-*d*) δ ppm 1.15 - 1.31 (m, 1 H) 3.40 - 3.58 (m, 1 H) 4.73 (s, 2 H) 7.10 - 7.26 (m, 2 H) 7.30 - 7.47 (m, 7 H) 7.54 (d, *J*=1.00 Hz, 2 H) 7.89 - 8.02 (m, 1 H) 8.08 - 8.26 (m, 1 H)

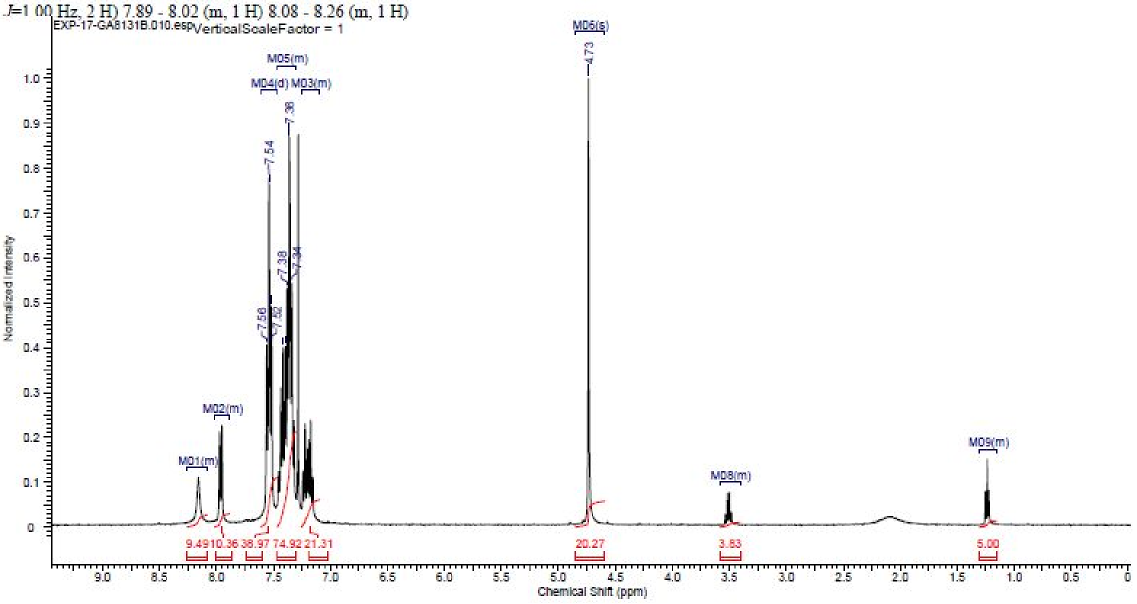

LC-MS spectra for compound 4

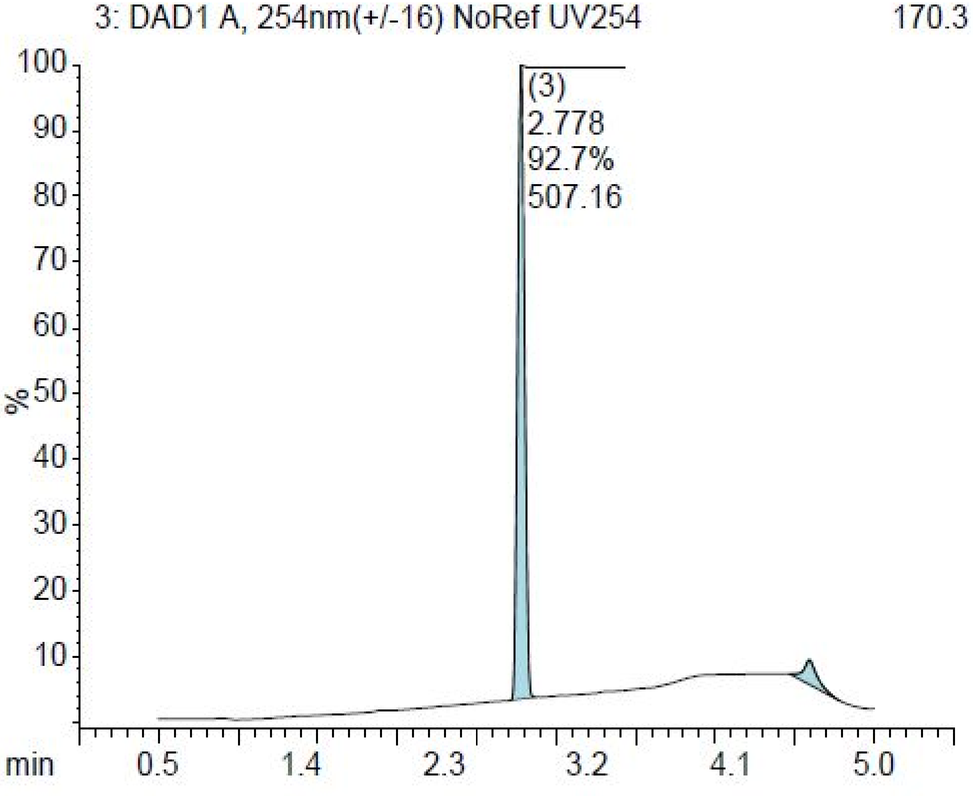

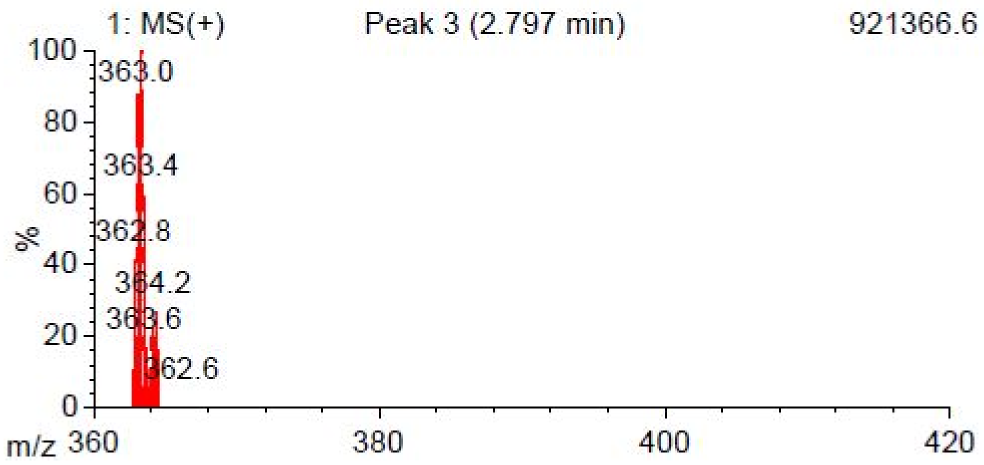

^{1}H NMR spectra for compound 5

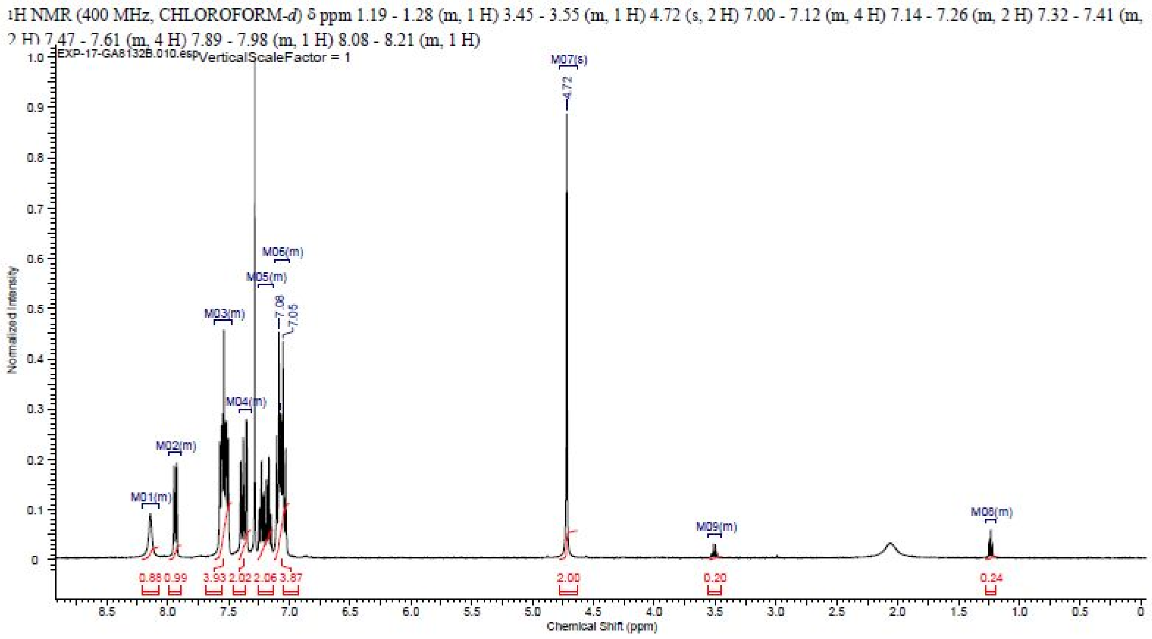

LC-MS spectra for compound 5

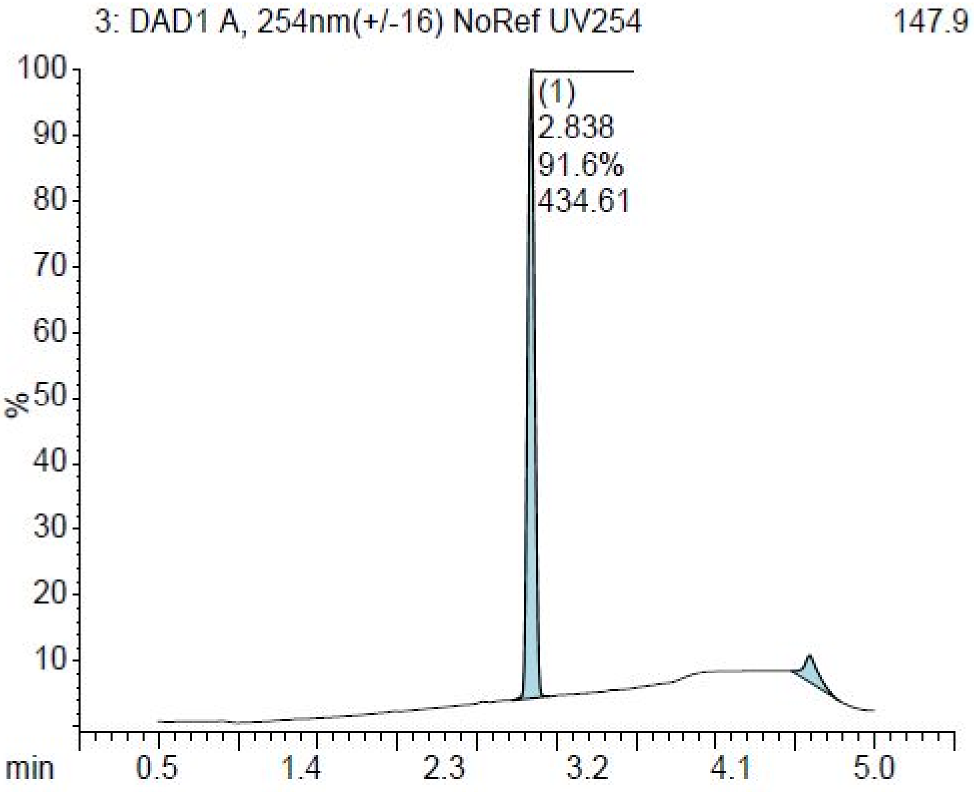

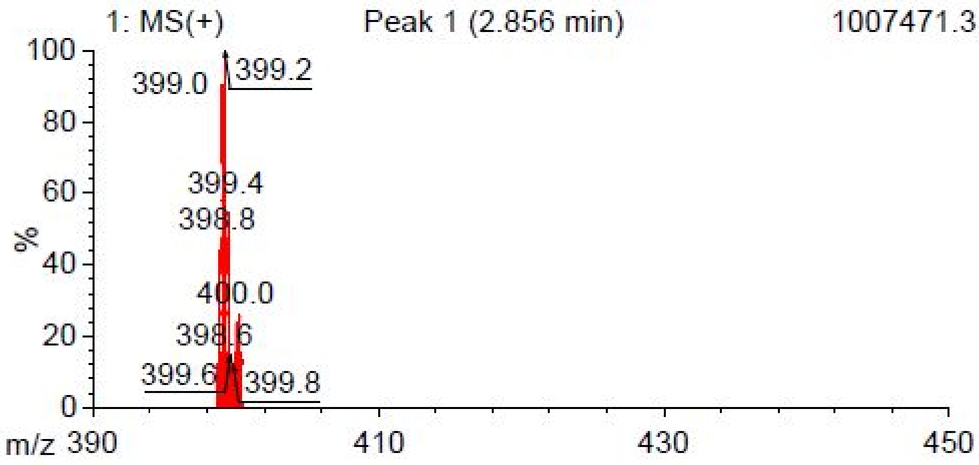

¹H NMR spectra for compound 6

¹H NMR (400 MHz, CHLOROFORM-*d*) δ ppm 4.78 (s, 2 H) 7.08 - 7.48 (m, 13 H) 7.80 - 7.93 (m, 1 H) 8.05 - 8.27 (m, 1 H)

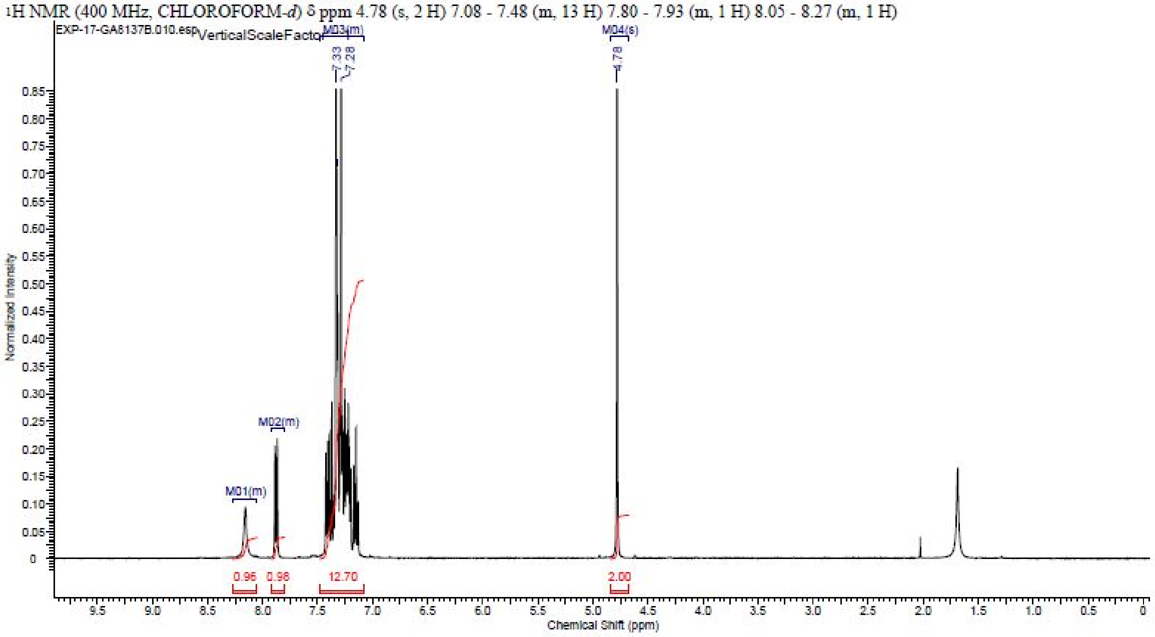

LCMS spectra for compound 6

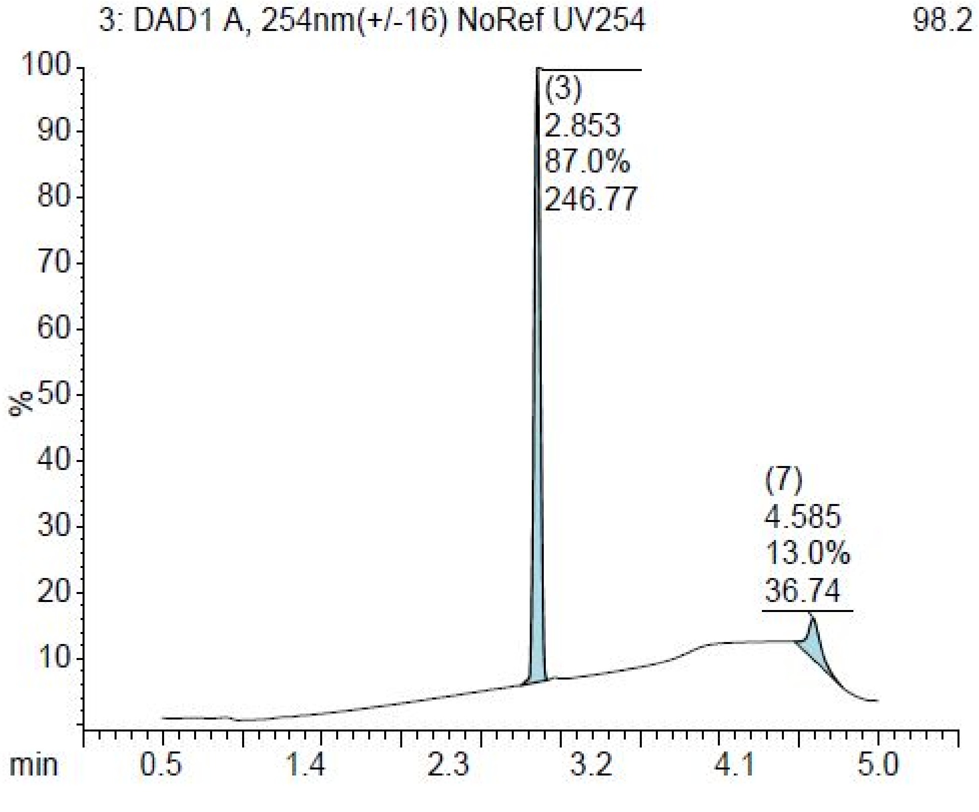

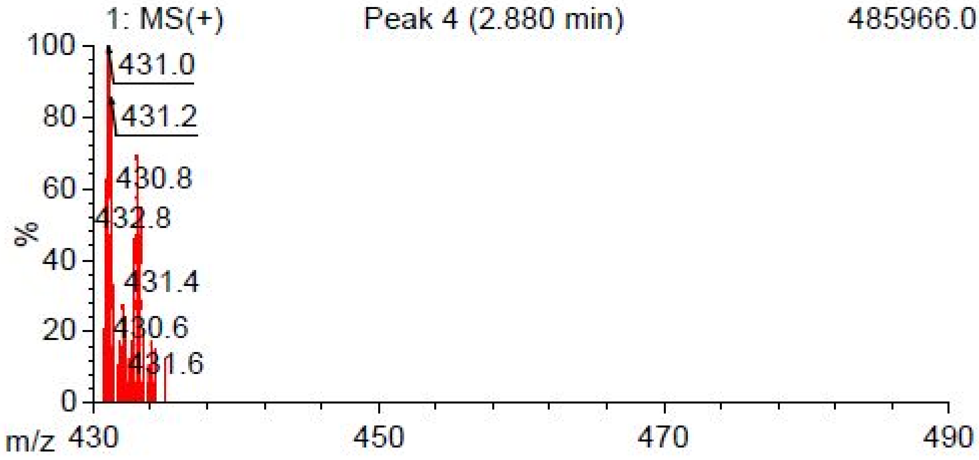

^{1}H NMR spectra for compound 7

^{1}H NMR (400 MHz, CHLOROFORM-*d*) δ ppm 4.71 (s, 2 H) 7.13 - 7.19 (m, 1 H) 7.20 - 7.26 (m, 1 H) 7.31 - 7.35 (m, 1 H) 7.35 - 7.45 (m, 5 H) 7.51 (d, *J*=10.04 Hz, 4 H) 7.89 - 7.96 (m, 1 H) 8.06 - 8.15 (m, 1 H)

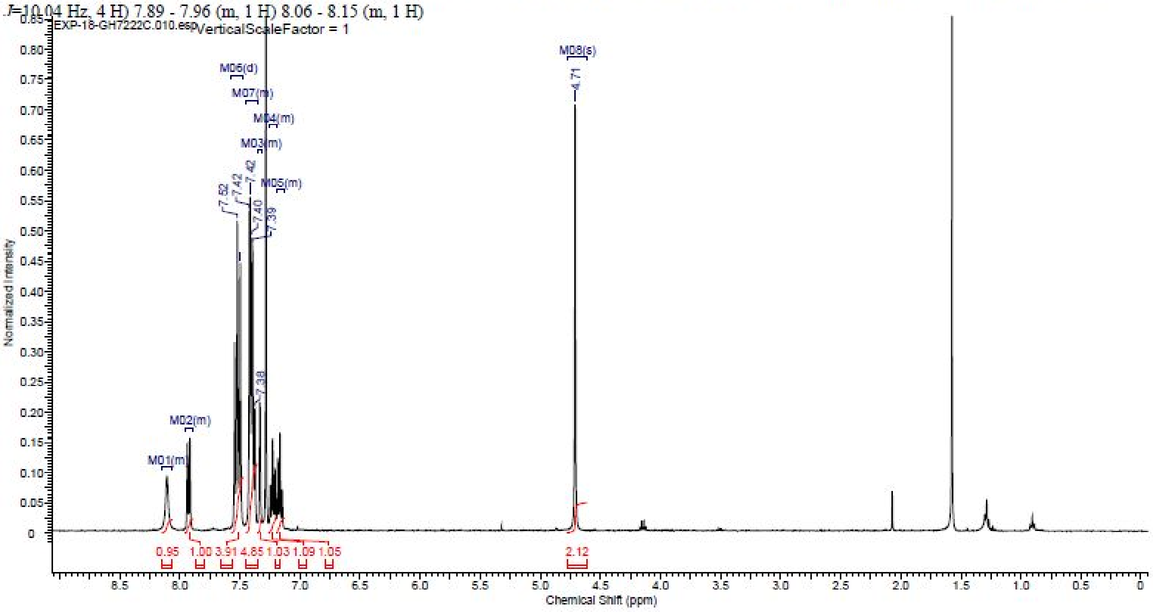

LCMS spectra for compound 7

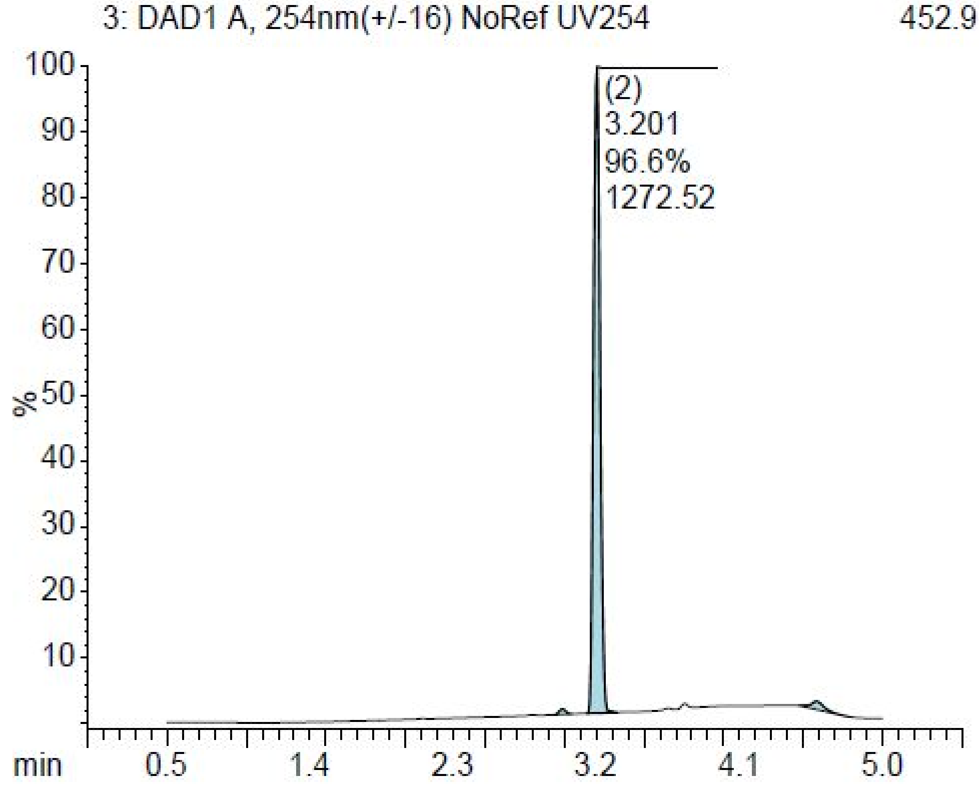

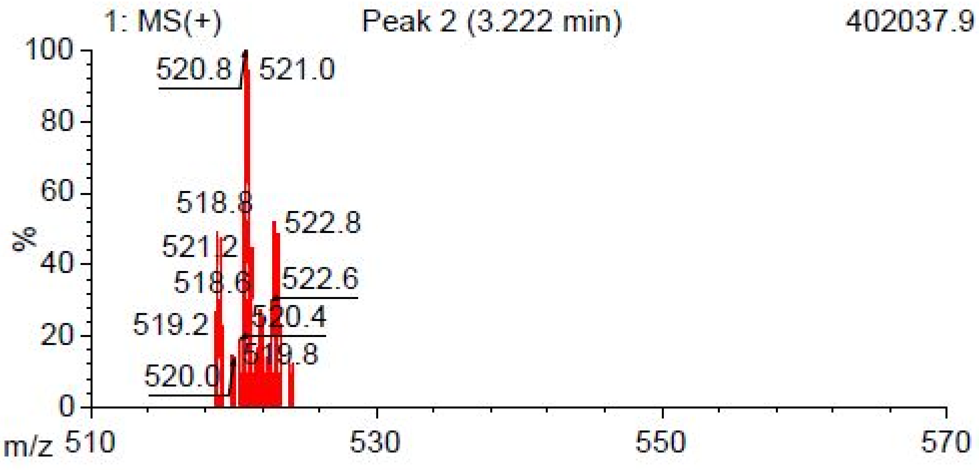

^{1}H NMR spectra for compound 8

1H NMR (400 MHz, CHLOROFORM-*d*) δ ppm 4.76 (s, 2 H) 7.28 (s, 5 H) 7.32 - 7.38 (m, 1 H) 7.73 - 7.95 (m, 3 H) 7.97 - 8.08 (m, 1 H) 8.09 - 8.19 (m, 1 H) 8.20 - 8.39 (m, 3 H)

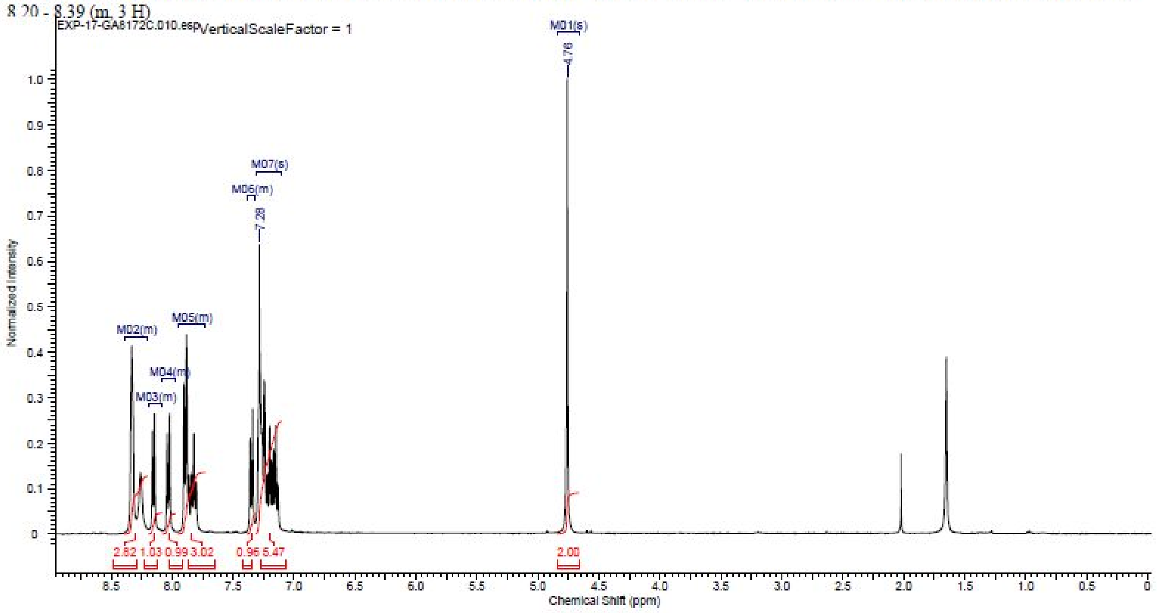

LCMS spectra for compound 8

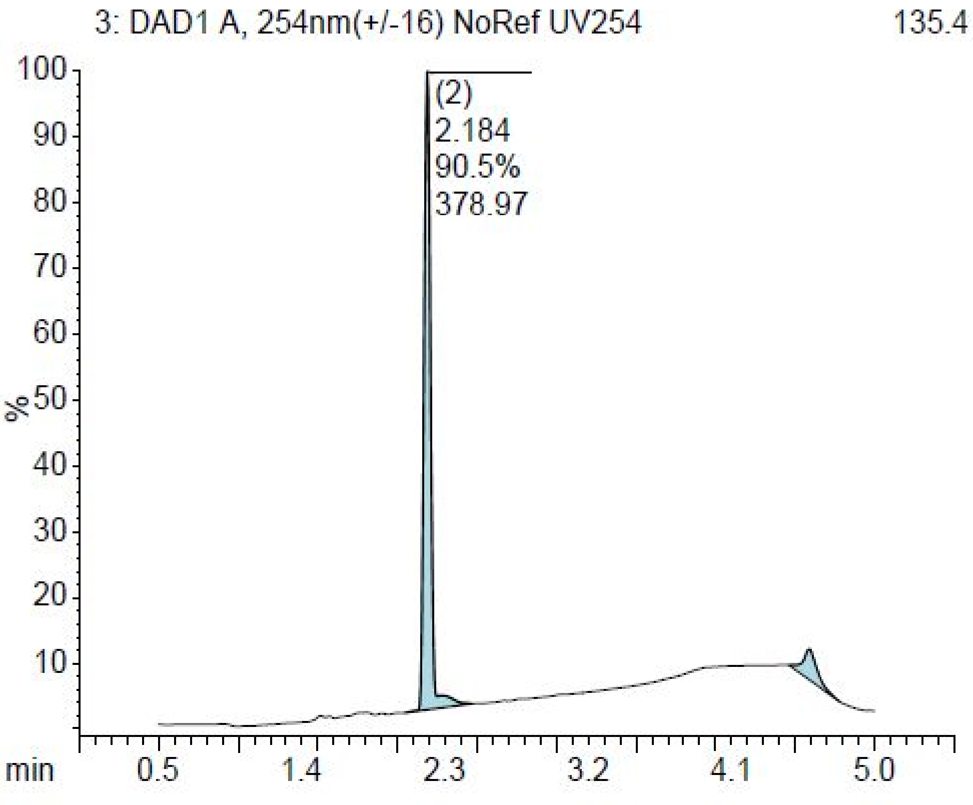

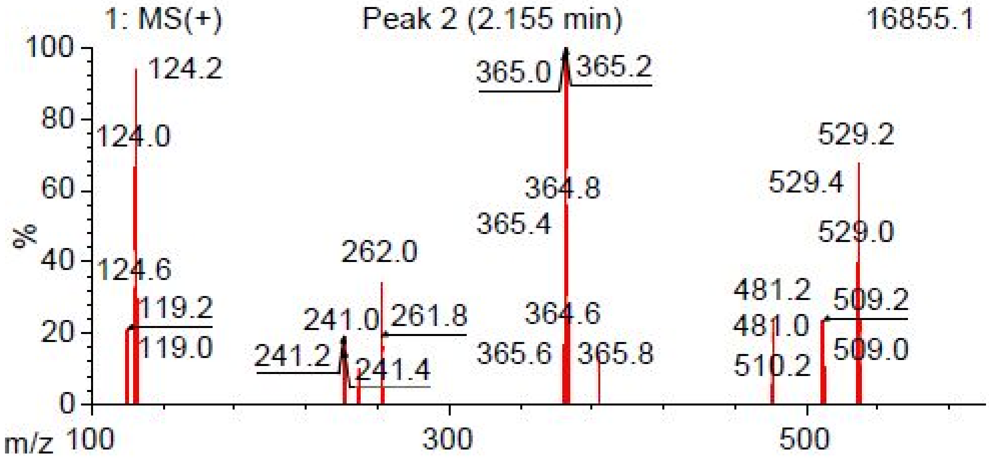

^{1}H NMR spectra for compound 9

^{1}H NMR (400 MHz, CHLOROFORM-*d*) δ ppm 1.22 - 1.44 (m, 12 H) 1.56 - 1.65 (m, 1 H) 3.15 - 3.41 (m, 2 H) 4.44 - 4.59 (m, 2 H) 7.10 - 7.24 (m, 2 H) 7.25 - 7.28 (m, 1 H) 7.30 - 7.43 (m, 1 H) 7.83 - 7.97 (m, 1 H) 8.00 - 8.18 (m, 1 H)

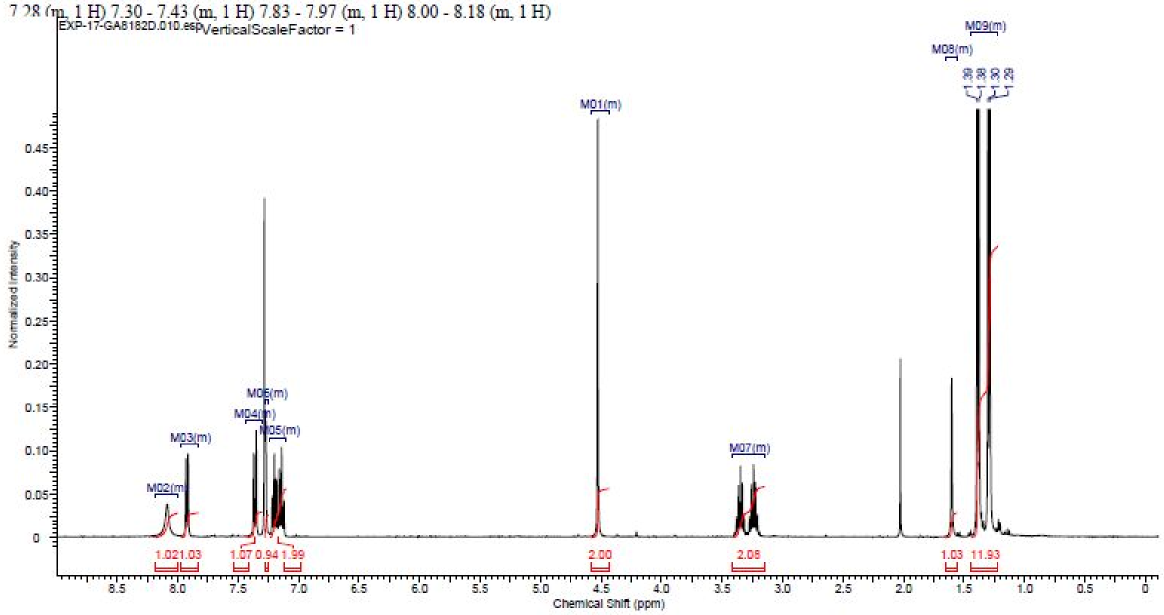

LCMS spectra for compound 9

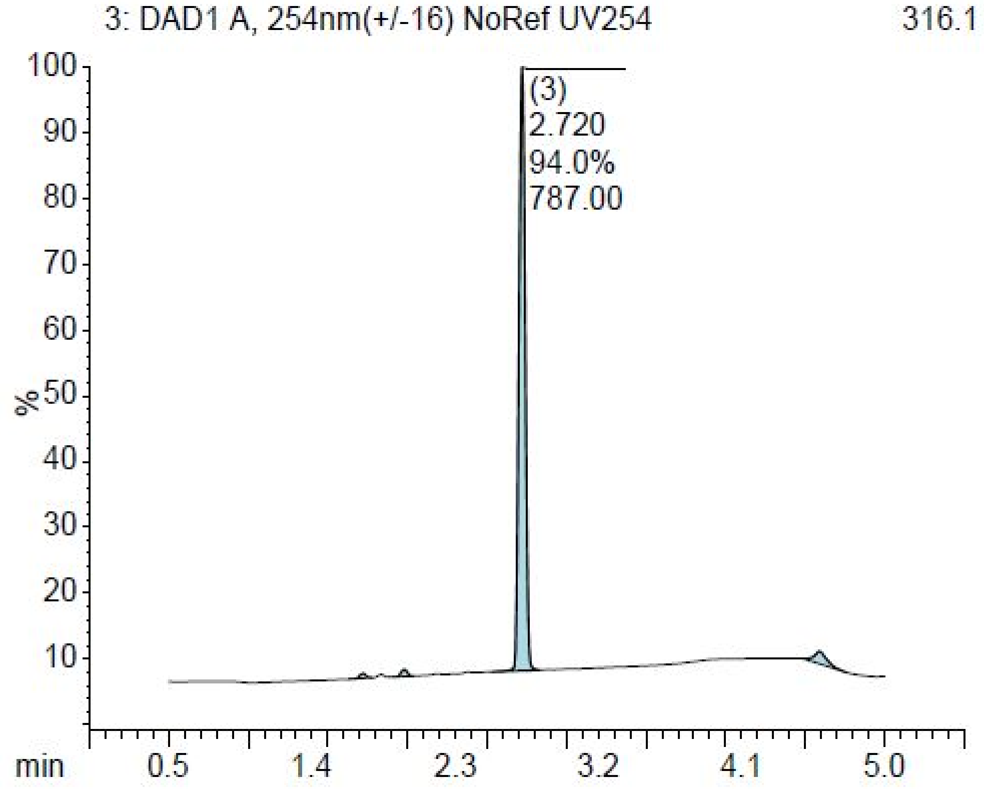

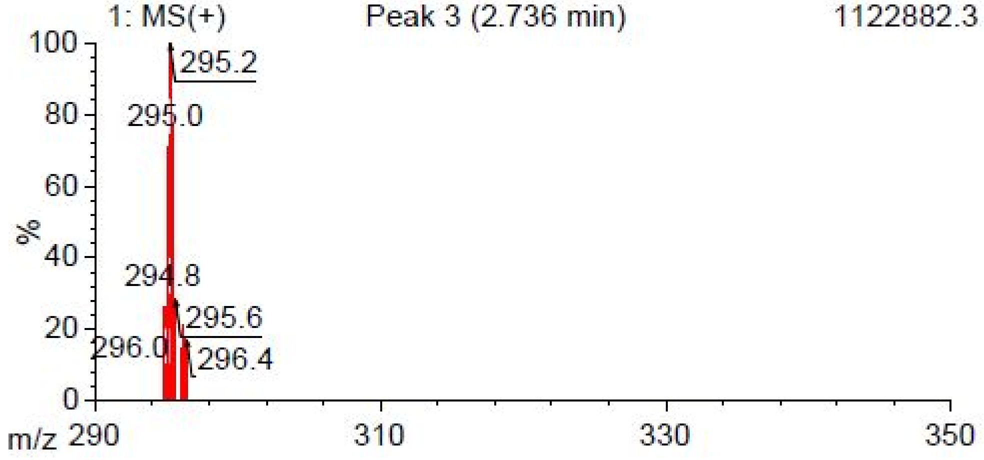

^{1}H NMR spectra for compound 10

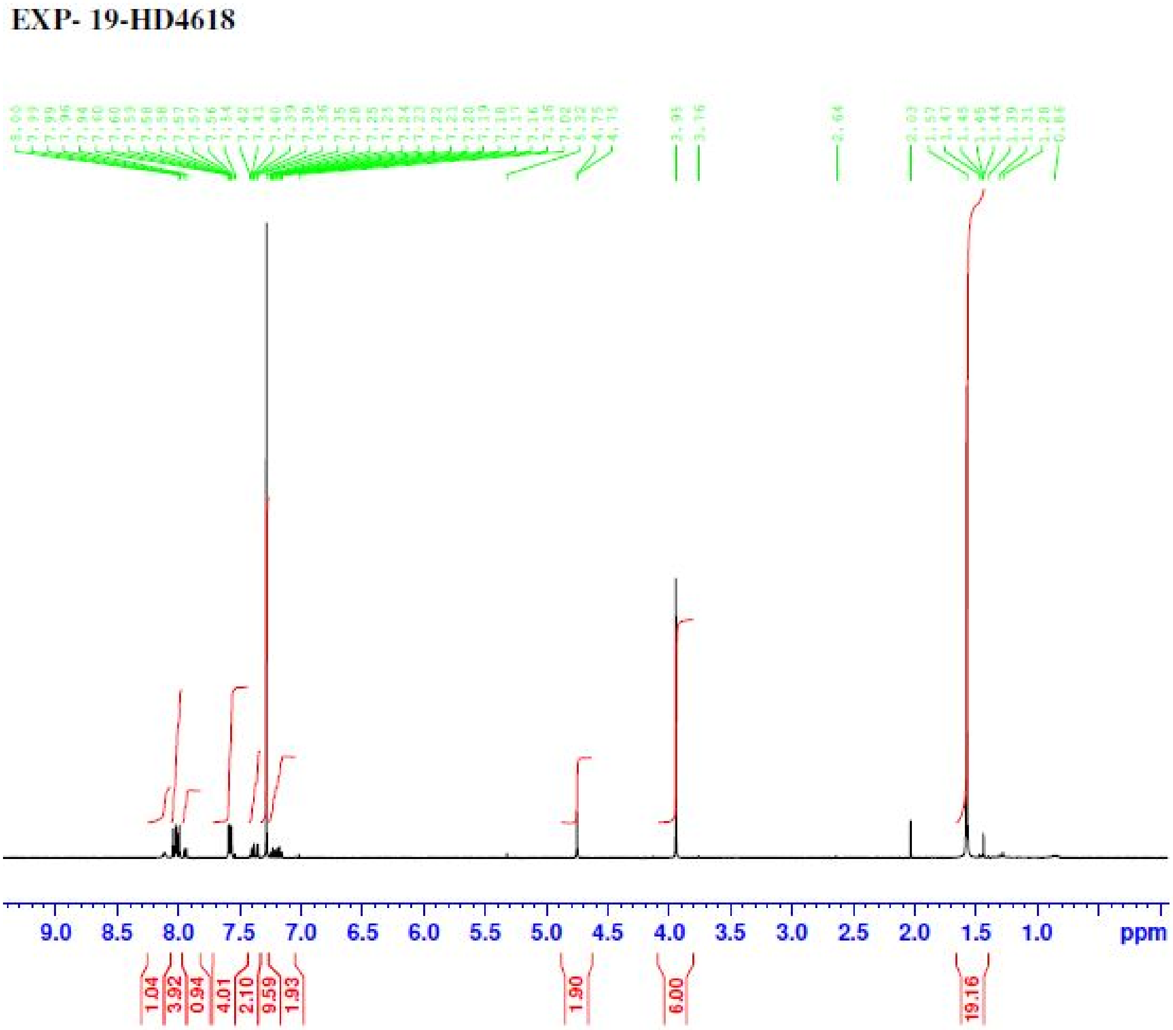

LCMS spectra for compound 10

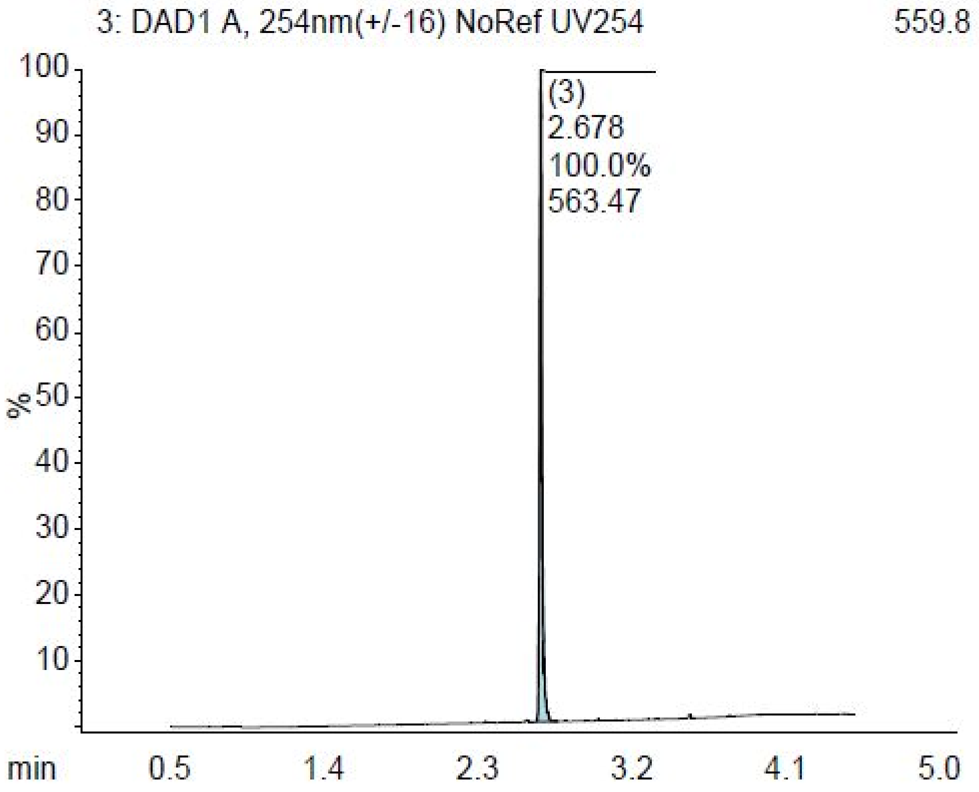

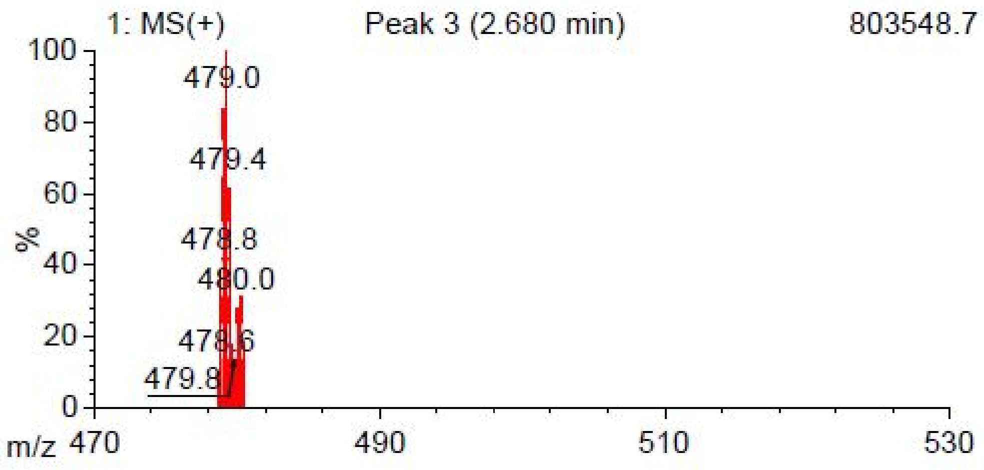

^{1}H NMR spectra for compound 11

^{1}H NMR (400 MHz, DMSO-d_6) δ 10.88 - 11.04 (m, 1H), 7.82 - 7.96 (m, 2H), 7.68 - 7.76 (m, 1H), 7.41 - 7.47 (m, 1H), 7.28 - 7.38 (m, 3H), 7.21 (dd, J = 3.76, 5.02 Hz, 1H), 6.93 - 7.16 (m, 3H), 4.49 (s, 2H)

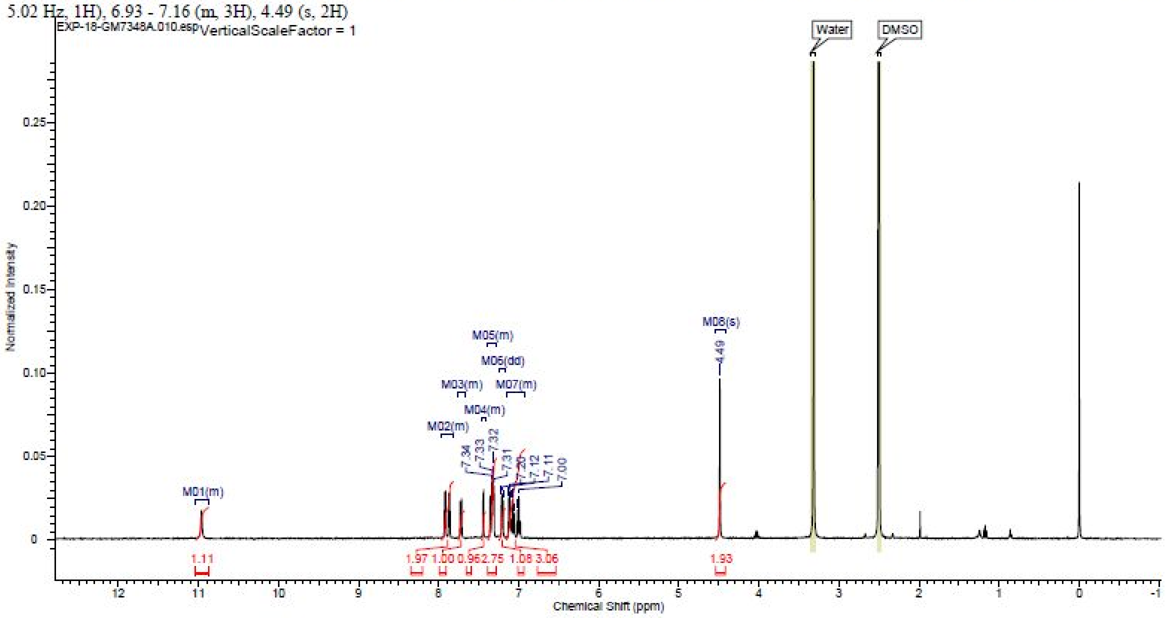

LCMS spectra for compound 11

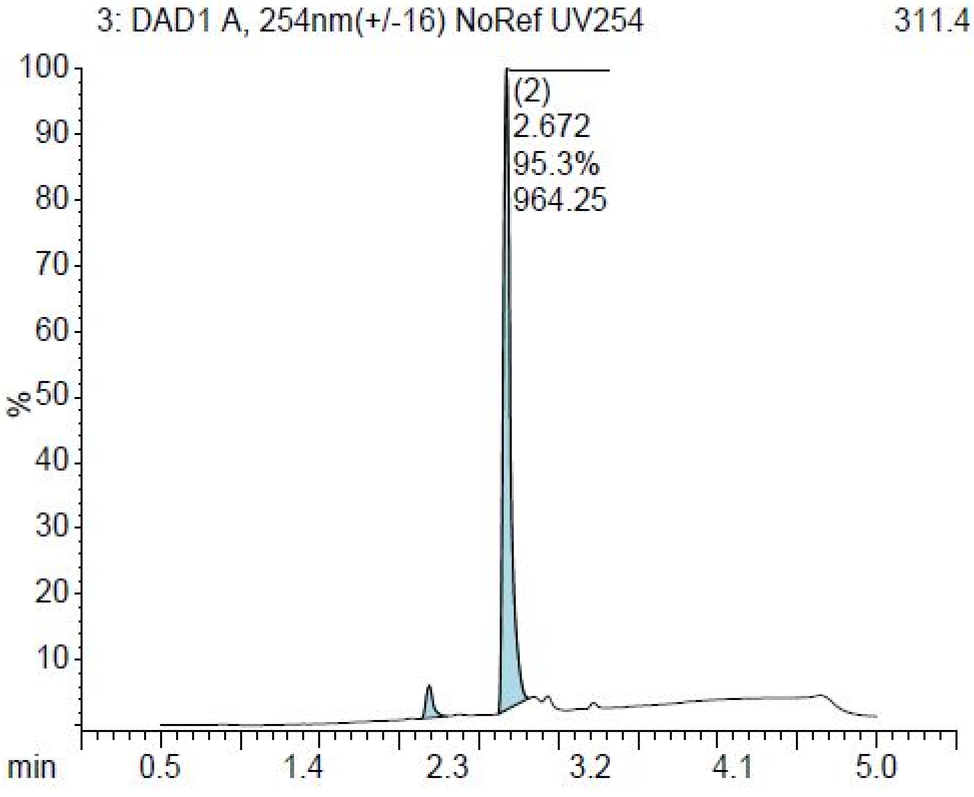

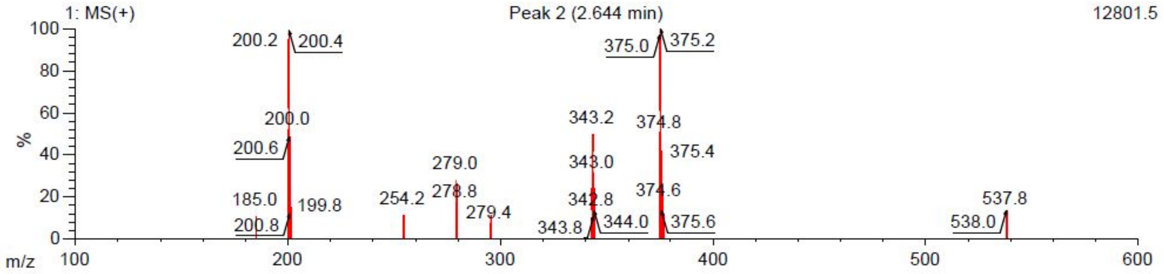

^{1}H NMR spectra for compound 12

1H NMR (400 MHz, CHLOROFORM-*d*) δ ppm 3.85 (d, *J*=1.00 Hz, 6 H) 4.62 (s, 2 H) 6.82 - 7.01 (m, 5 H) 7.28 (s, 4 H) 7.35 - 7.42 (m, 1 H) 7.47 - 7.66 (m, 5 H) 8.16 (br. s., 1 H)

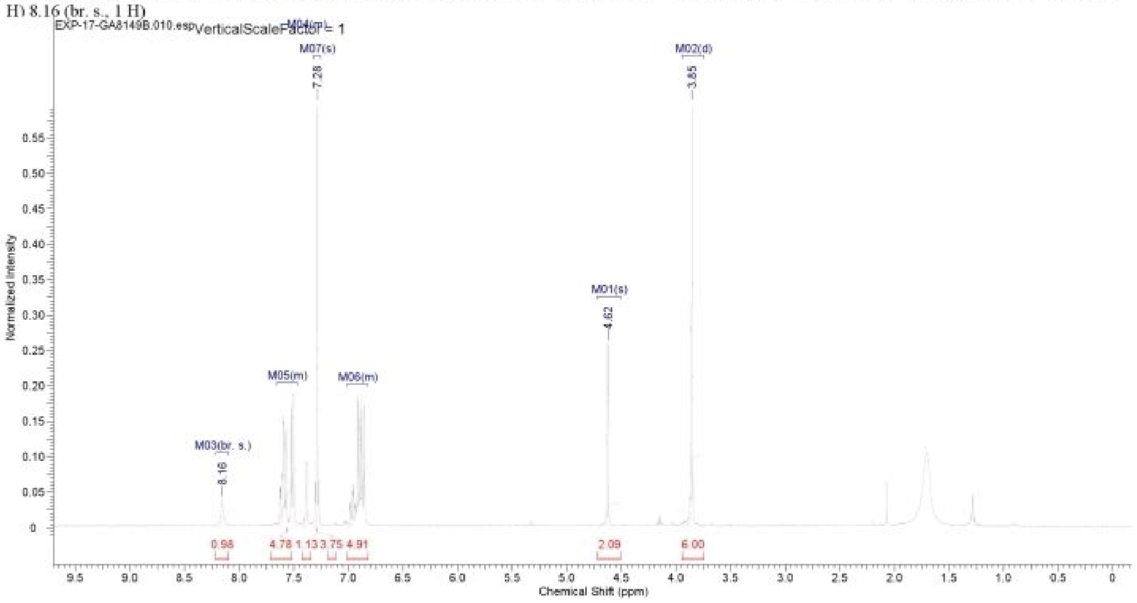

LCMS spectra for compound 12

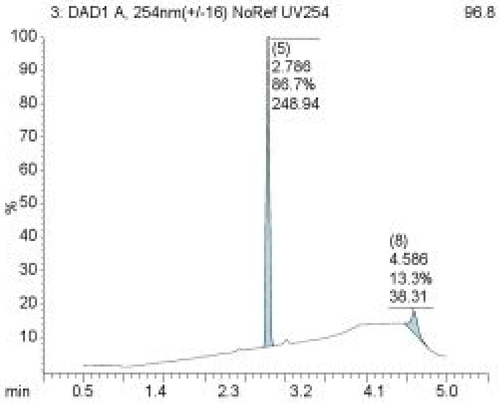

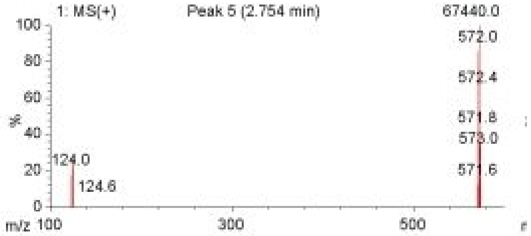

^{1}H NMR spectra for compound 13

^{1}H NMR (400 MHz, CHLOROFORM-*d*) δ ppm 3.86 (s, 3 H) 3.93 (d, *J*=2.01 Hz, 6 H) 4.74 (s, 2 H) 6.91 - 7.01 (m, 4 H) 7.21 - 7.27 (m, 1 H) 7.30 - 7.42 (m, 2 H) 7.55 - 7.69 (m, 4 H) 7.99 - 8.05 (m, 1 H)

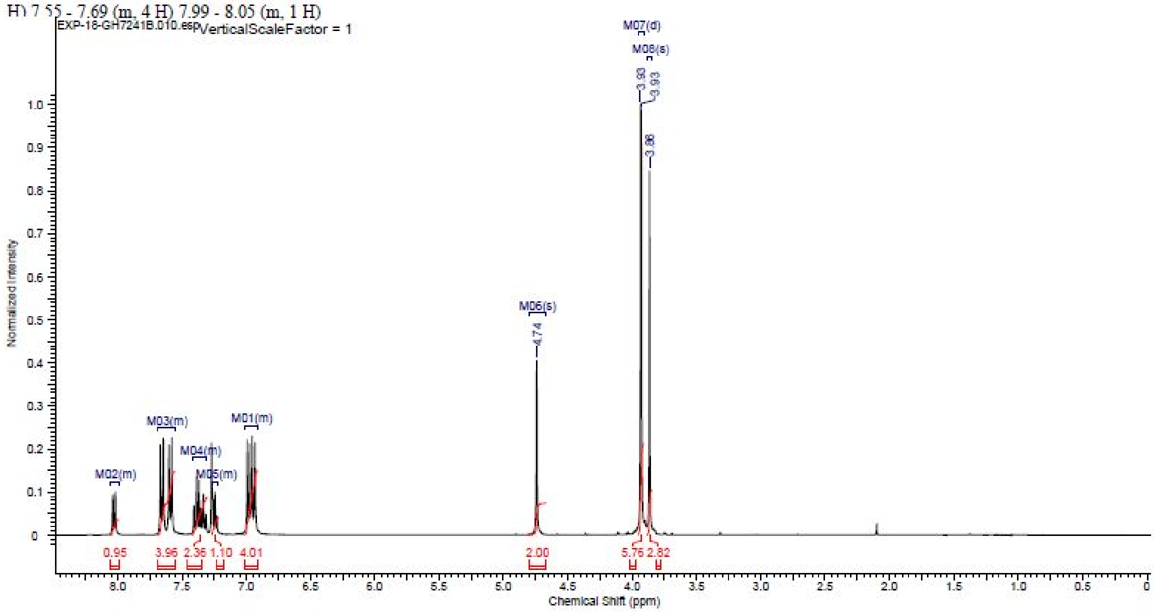

LCMS spectra for compound 13

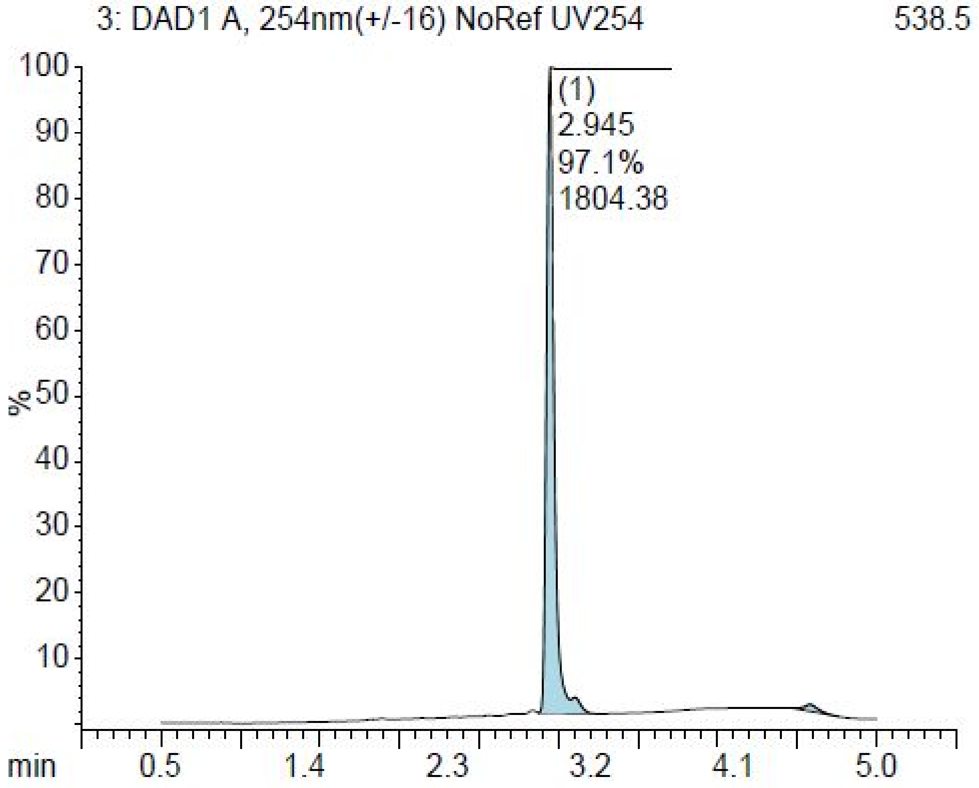

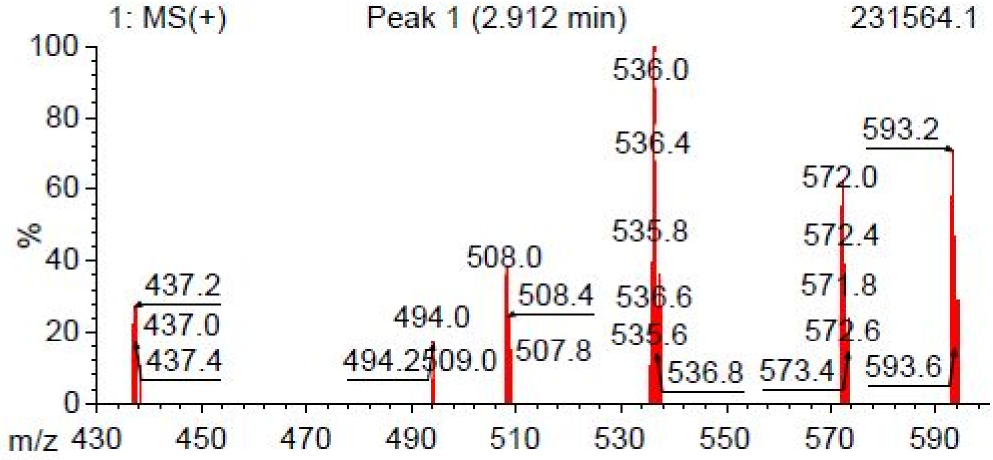

^{1}H NMR spectra for compound 14

^{1}H NMR (400 MHz, DMSO-d_6) δ ppm 3.78 (d, J=8.53 Hz, 6 H) 4.72 - 4.83 (m, 2 H) 6.92 (d, J=8.78 Hz, 2 H) 6.99 (s, 2 H) 7.06 - 7.13 (m, 1 H) 7.30 - 7.37 (m, 1 H) 7.45 (dd, J=8.66, 3.64 Hz, 5 H) 7.80 - 7.87 (m, 1 H) 12.81 - 12.87 (m, 1 H)

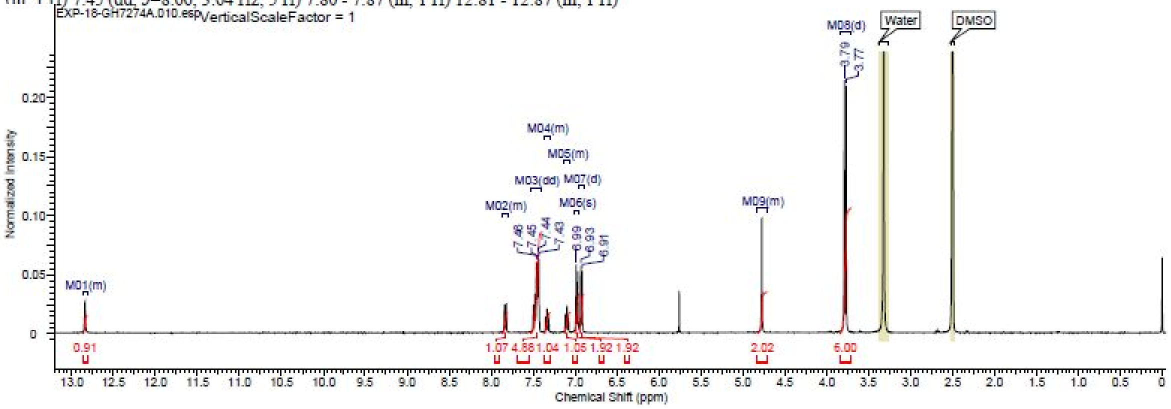

LCMS spectra for compound 14

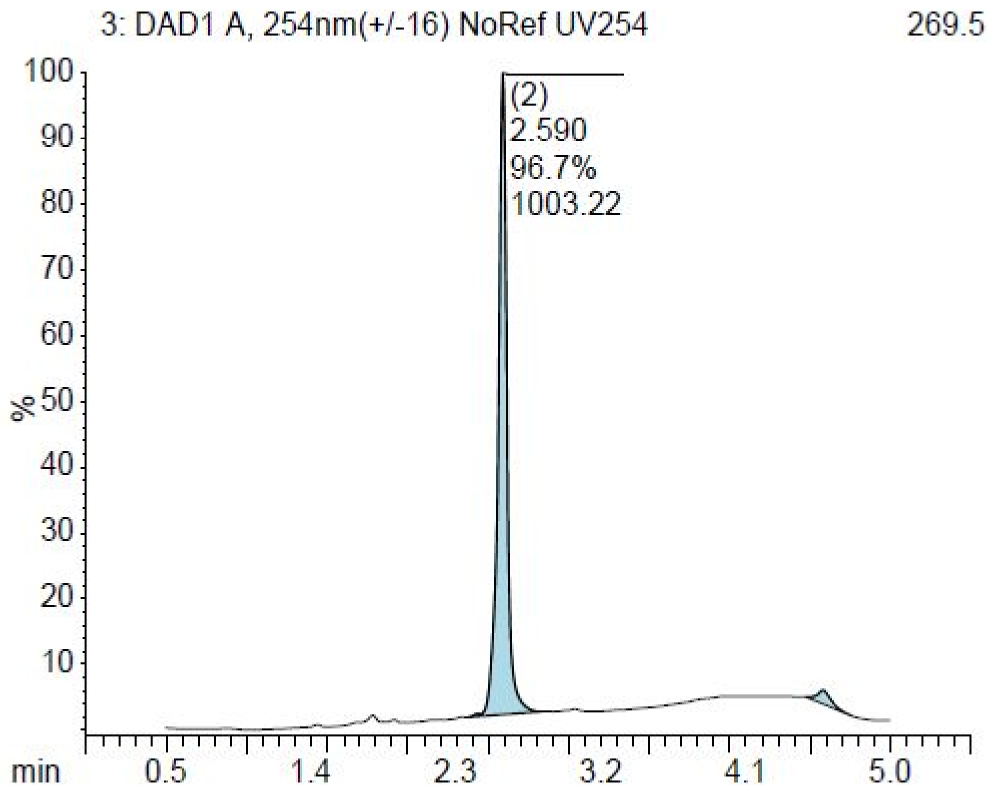

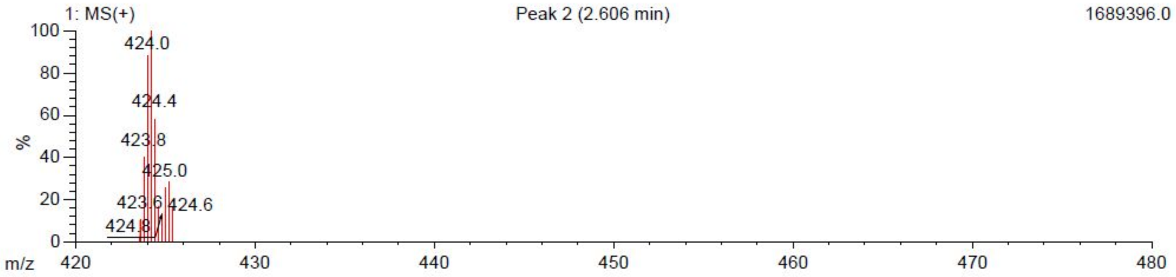

^{1}H NMR spectra for compound 15

^{1}H NMR (400 MHz, DMSO-d_6) δ 10.94 - 11.09 (m, 1H), 7.66 - 7.76 (m, 1H), 7.27 - 7.55 (m, 9H), 7.12 (dd, J = 2.26, 10.04 Hz, 1H), 6.79 - 6.93 (m, 1H), 4.56 (s, 2H)

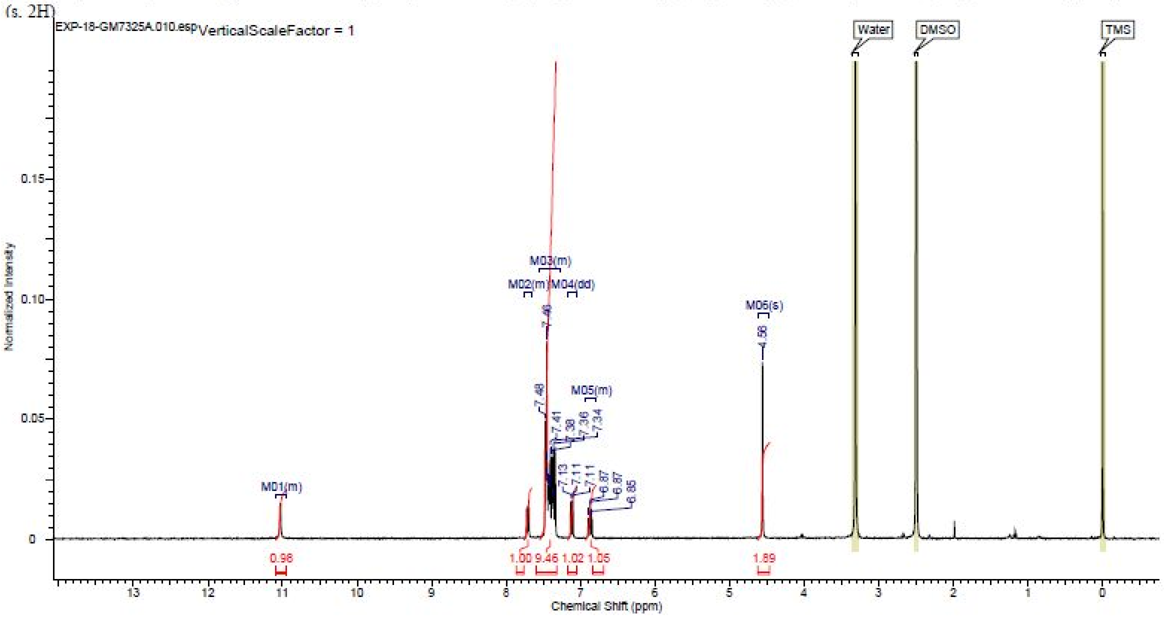

LCMS spectra for compound 15

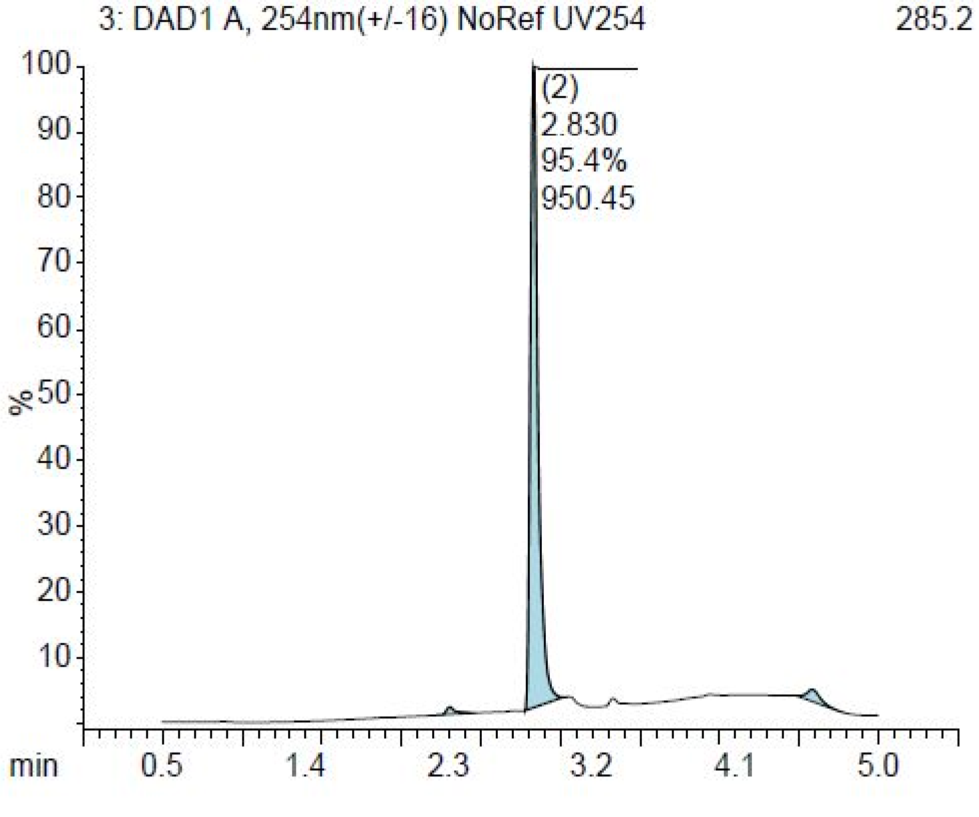

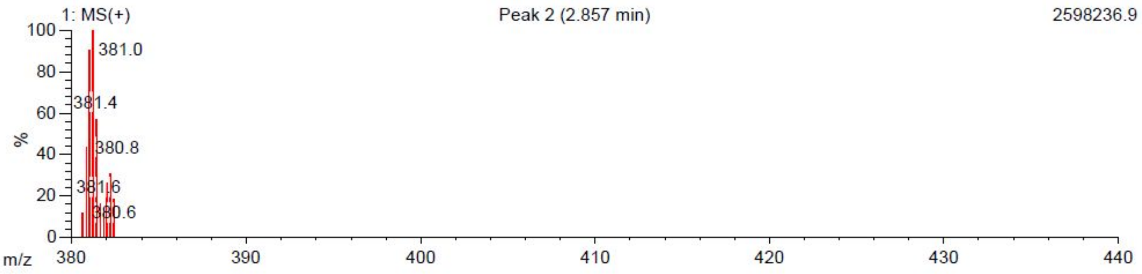

^{1}H NMR spectra for compound 16

^{1}H NMR (400 MHz, CHLOROFORM-d) δ 7.96 - 8.10 (m, 1H), 7.77 - 7.88 (m, 1H), 7.48 - 7.62 (m, 4H), 7.22 - 7.47 (m, 8H), 7.16 (s, 1H), 6.93 - 7.05 (m, 1H), 4.70 (s, 2H)

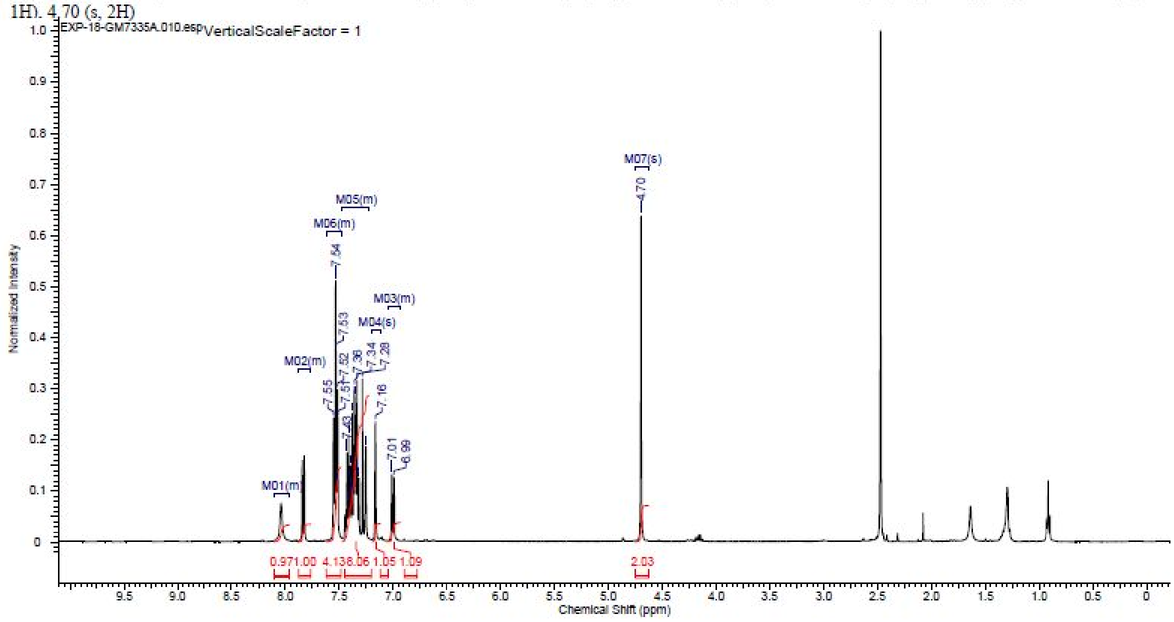

LCMS spectra for compound 16

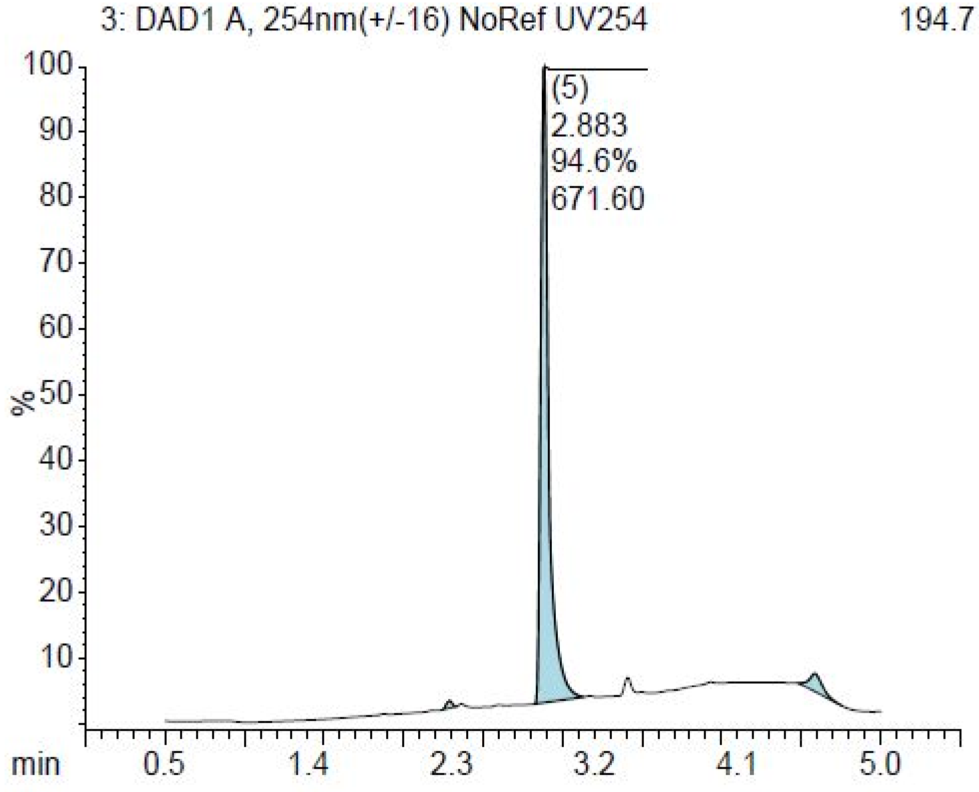

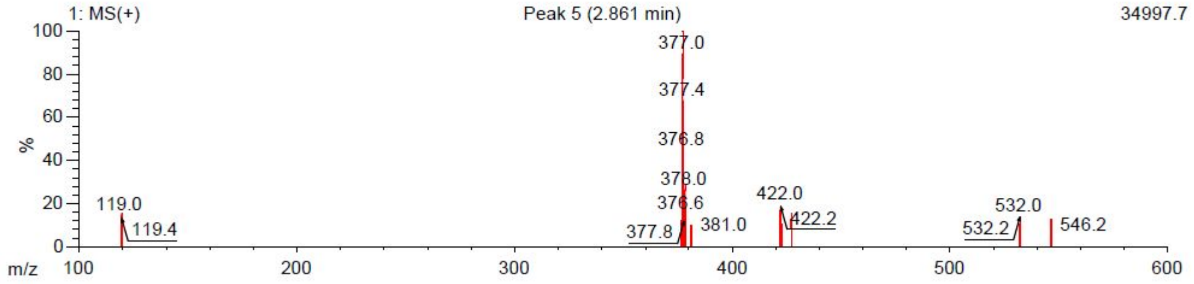

^{1}H NMR spectra for compound 17

^{1}H NMR (400 MHz, CHLOROFORM-d) δ 8.98 - 9.45 (m, 1H), 8.61 (s, 1H), 8.11 (d, J = 5.52 Hz, 1H), 7.65 (d, J = 5.27 Hz, 1H), 6.99 - 7.41 (m, 13H), 4.53 (s, 2H)

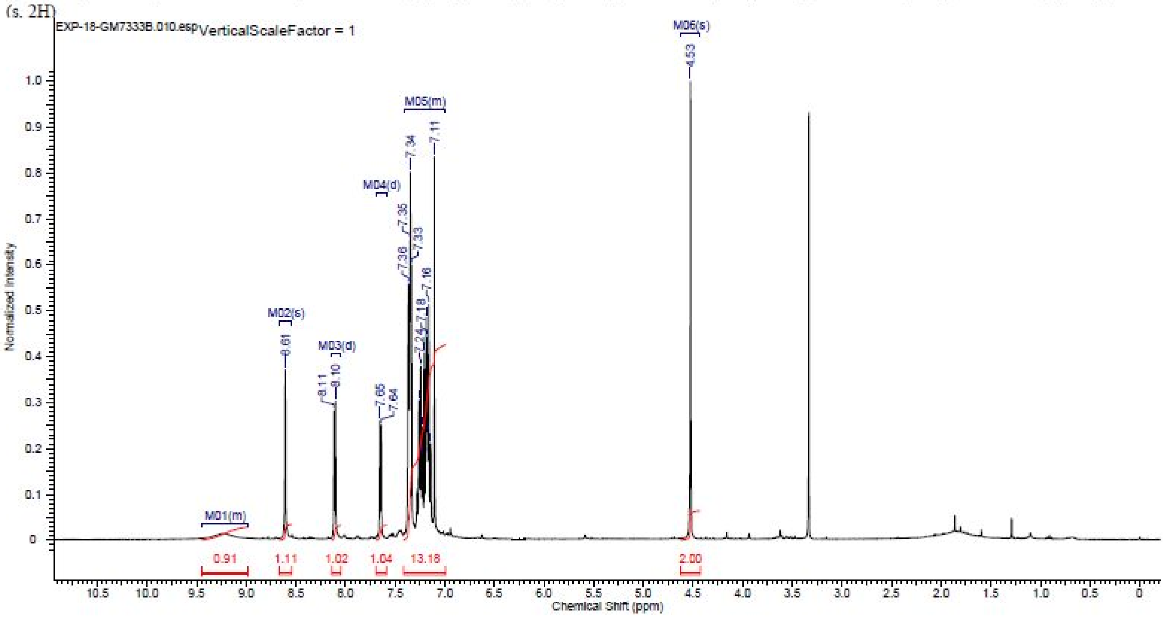

LCMS spectra for compound 17

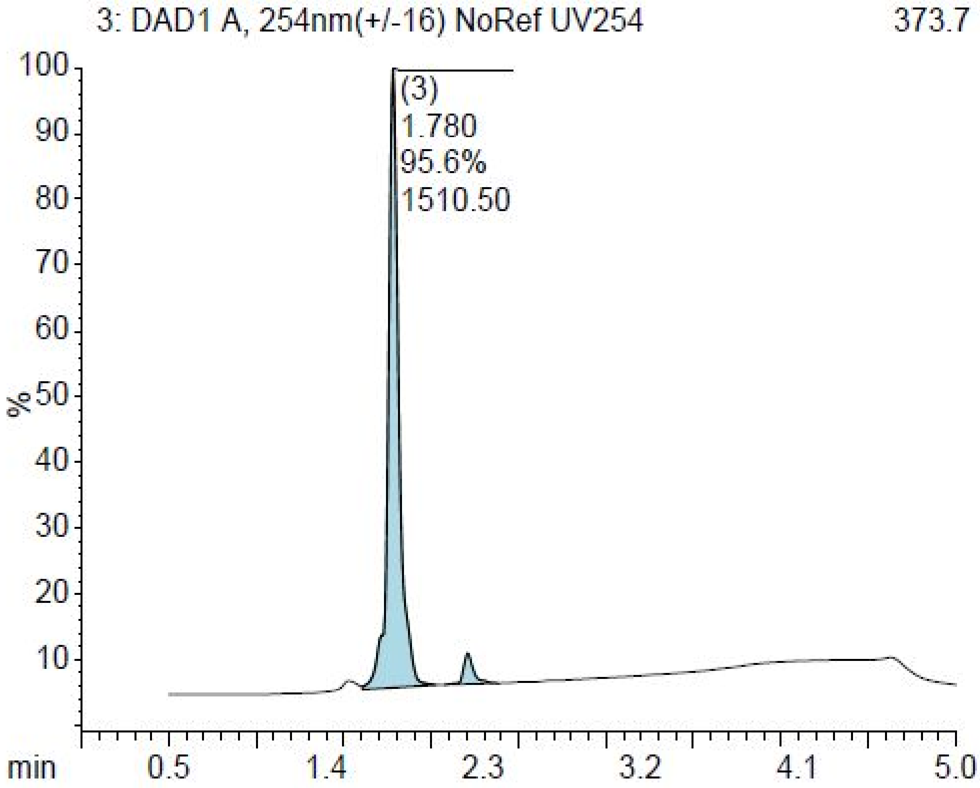

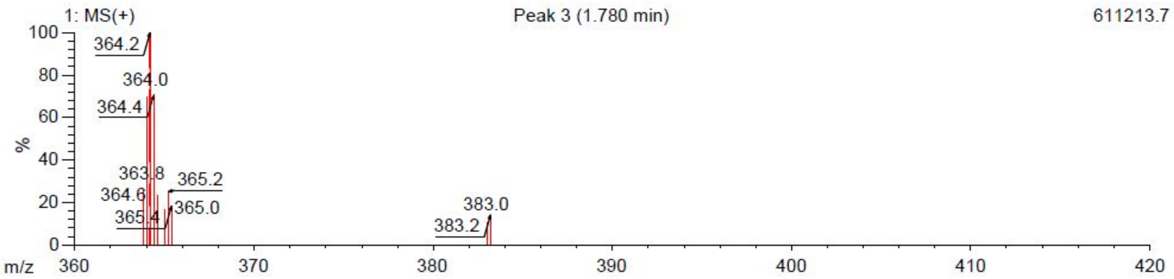

^{1}H spectra for compound 18

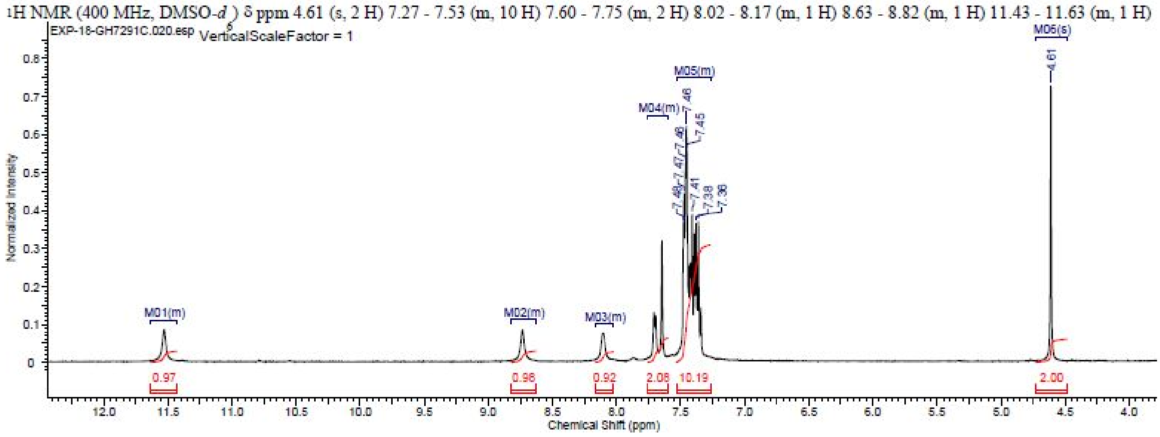

LCMS spectra for compound 18

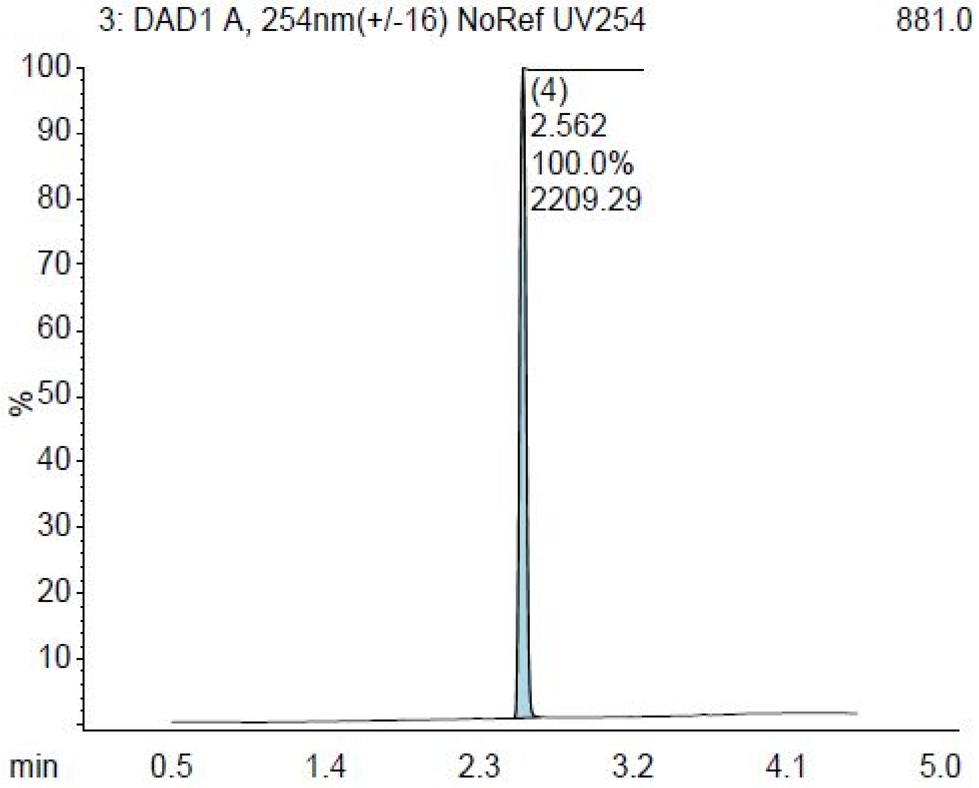

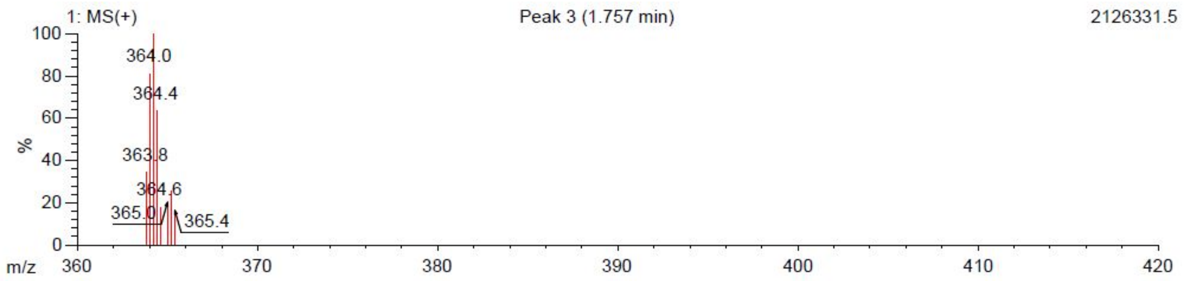

^{1}H spectra for compound 19

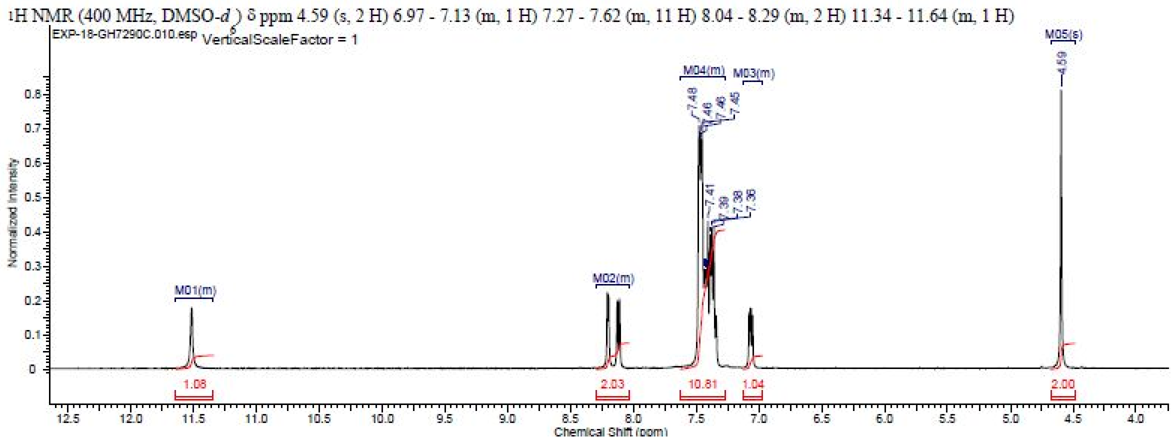

LCMS spectra for compound 19

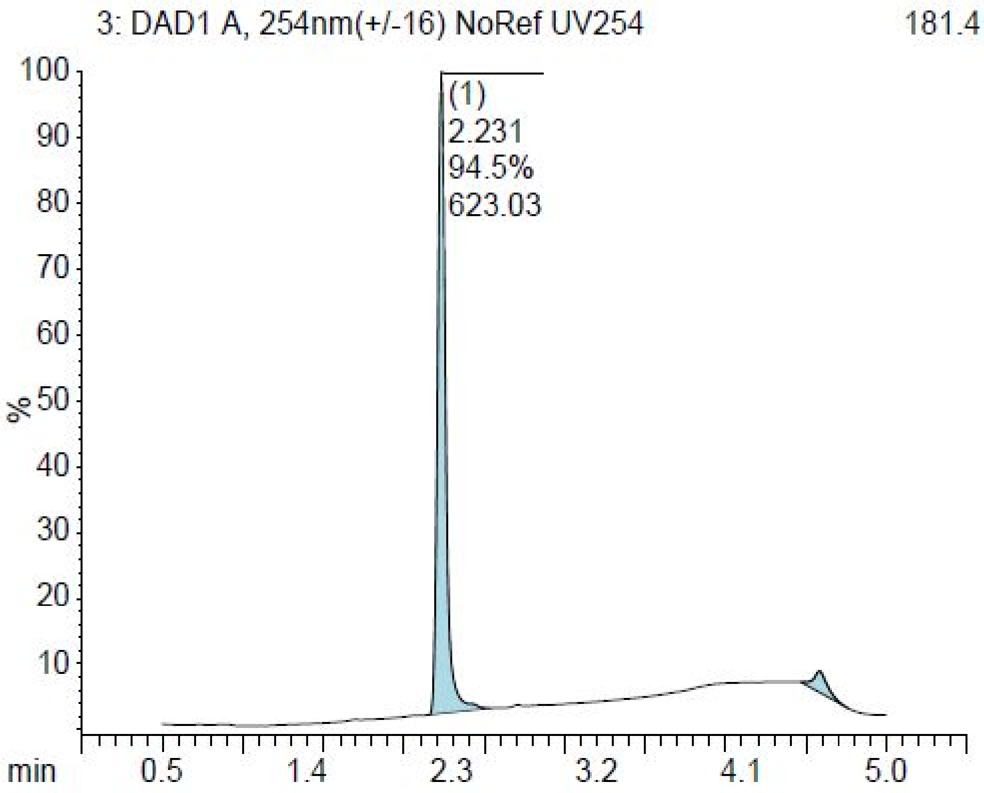

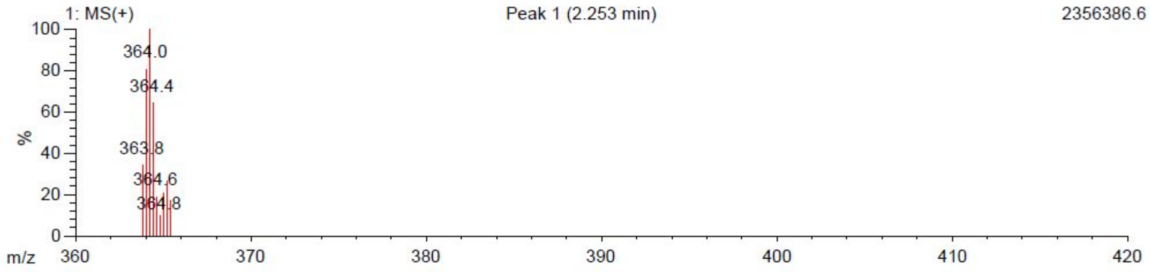

^{1}H spectra for compound 20

^{1}H NMR (400 MHz, DMSO-d_6) δ 13.42 - 13.52 (m, 1H), 8.43 - 8.55 (m, 1H), 8.20 (s, 1H), 7.03 - 7.29 (m, 9H), 6.98 (dd, J = 1.51, 7.78 Hz, 2H), 6.81 (d, J = 1.00 Hz, 1H)

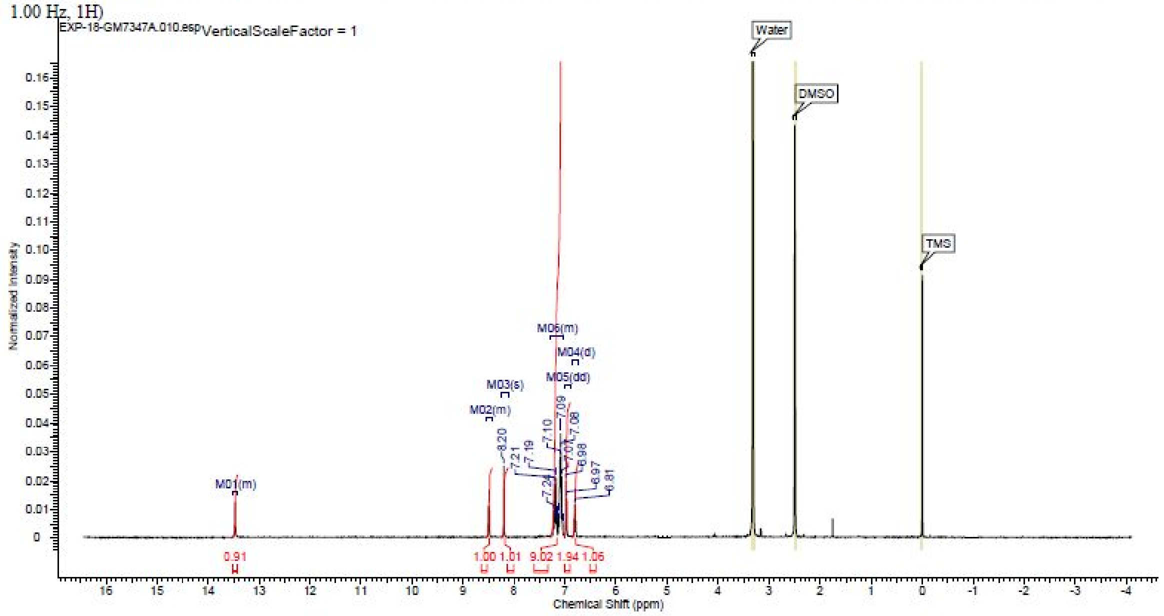

LCMS spectra for compound 20

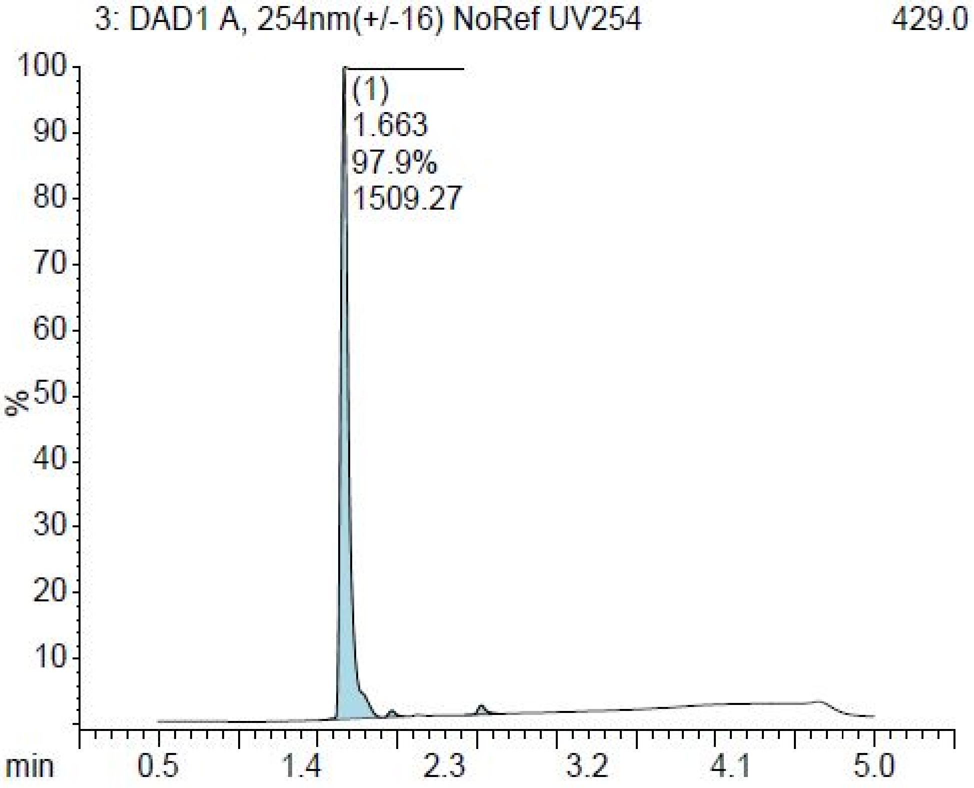

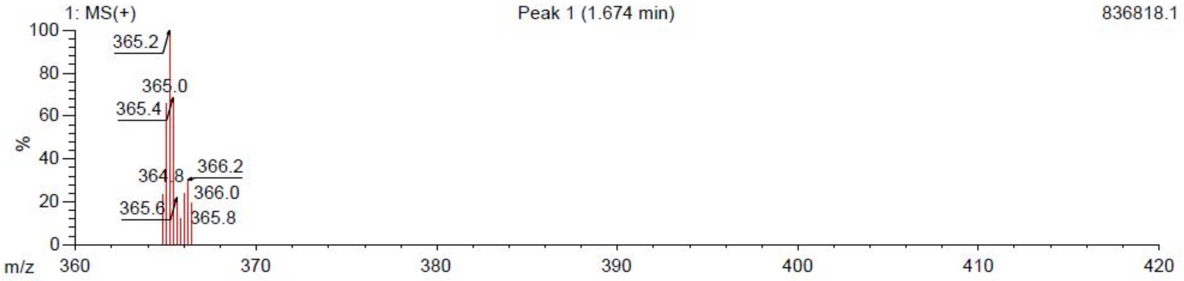

^{1}H spectra for compound 21

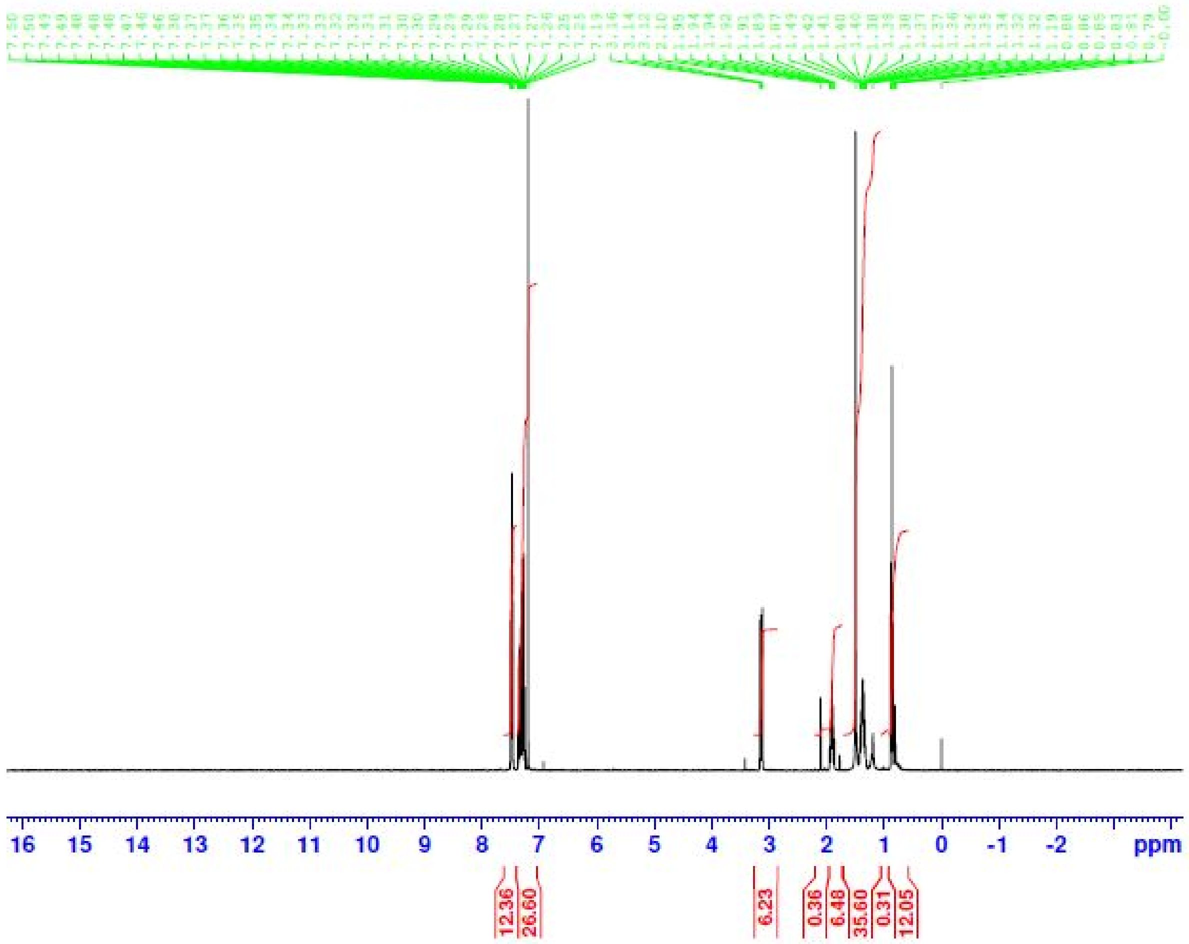

LCMS spectra for compound 21

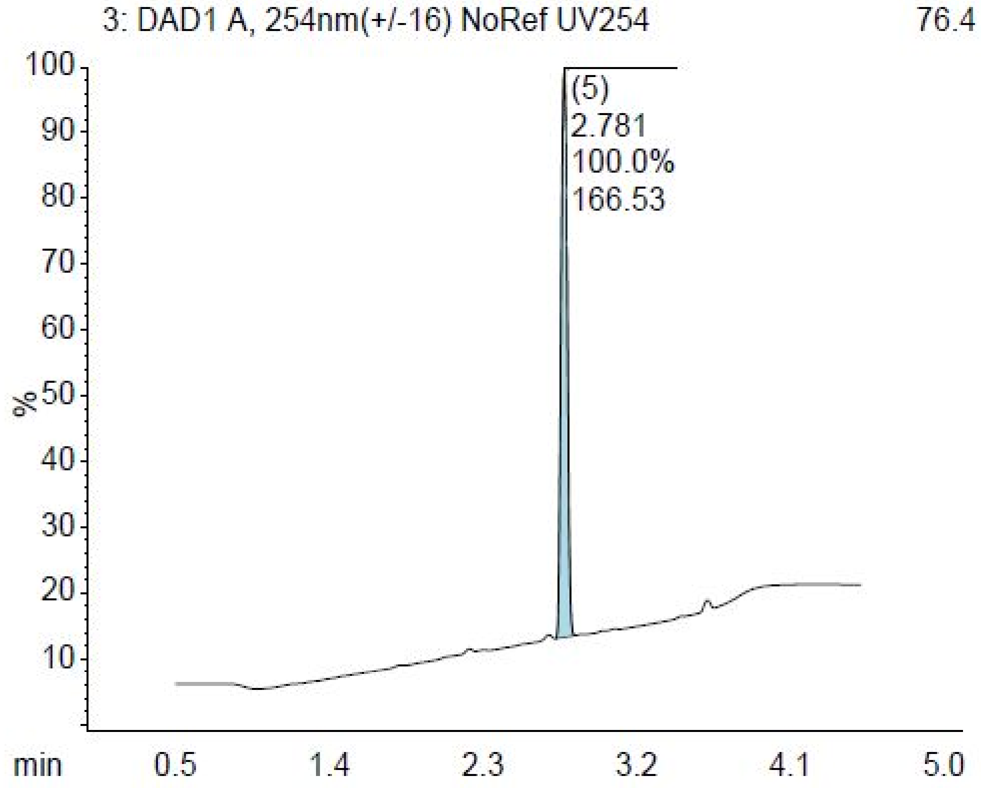

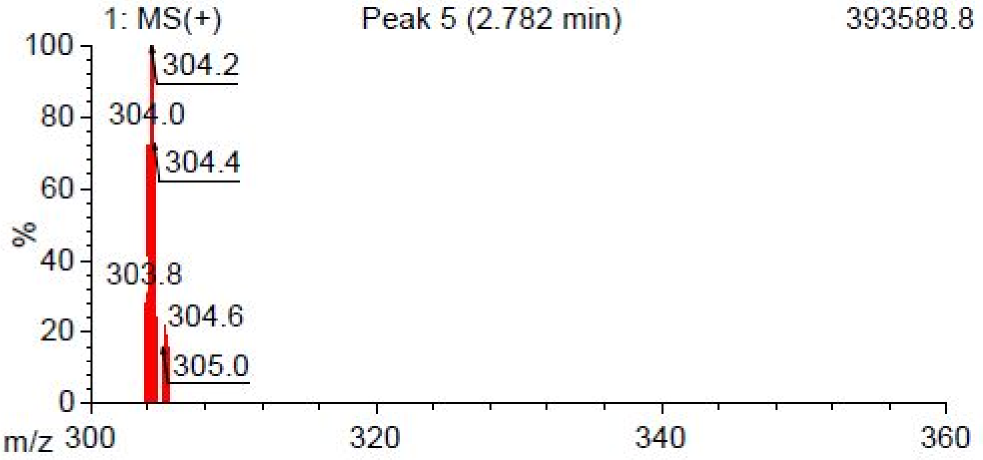

^{1}H spectra for compound 22

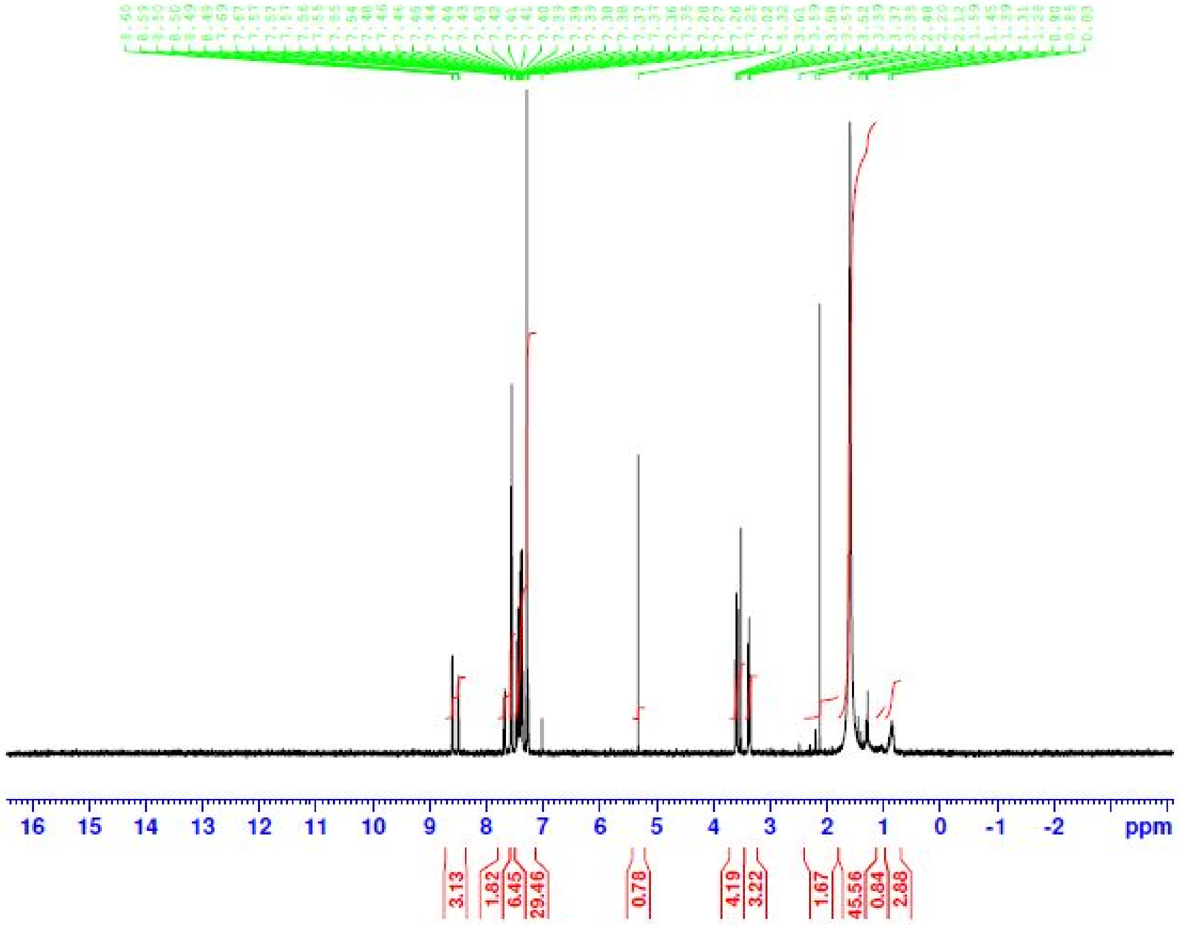

LCMS spectra for compound 22

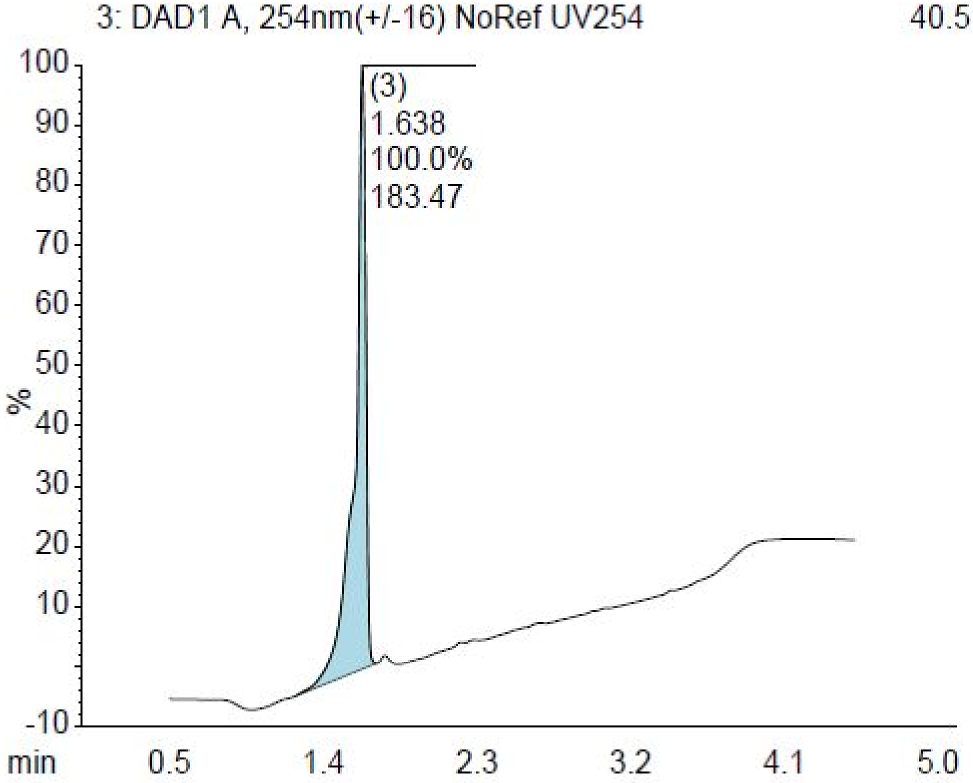

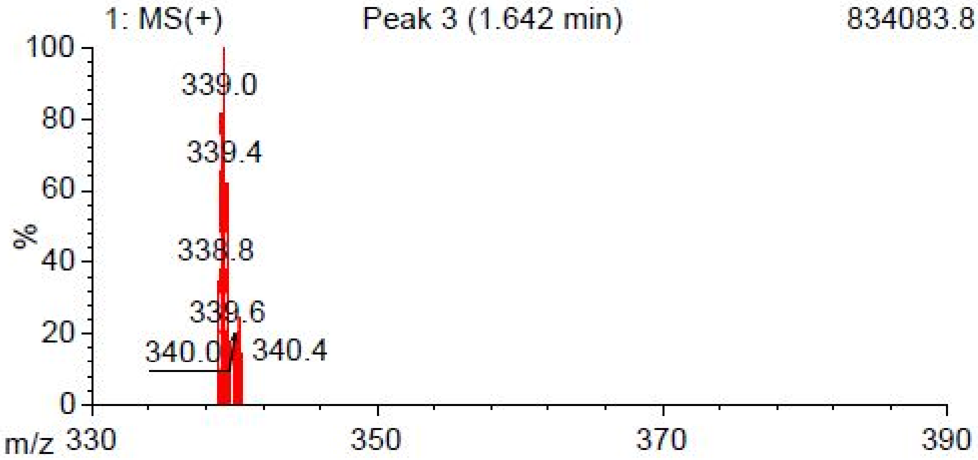

^{1}H spectra for compound 23

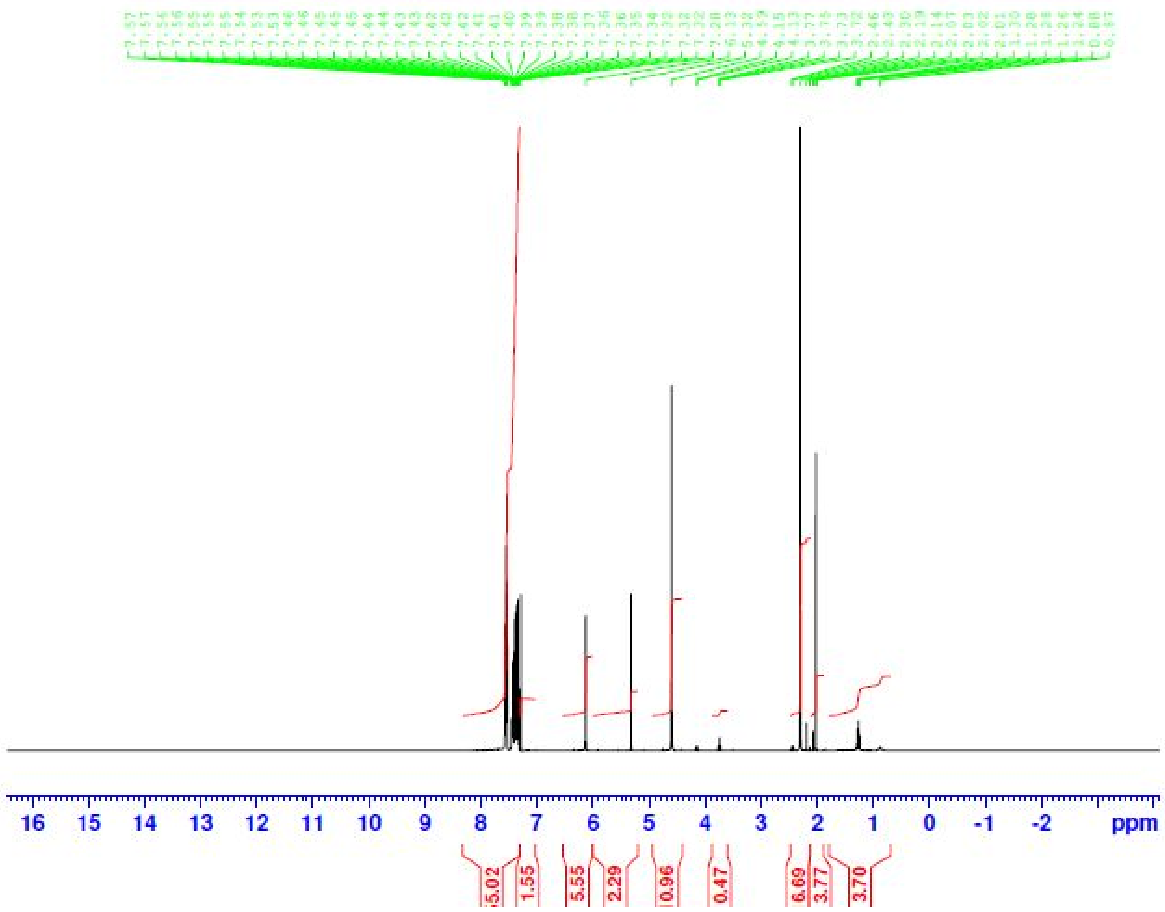

LCMS spectra for compound 23

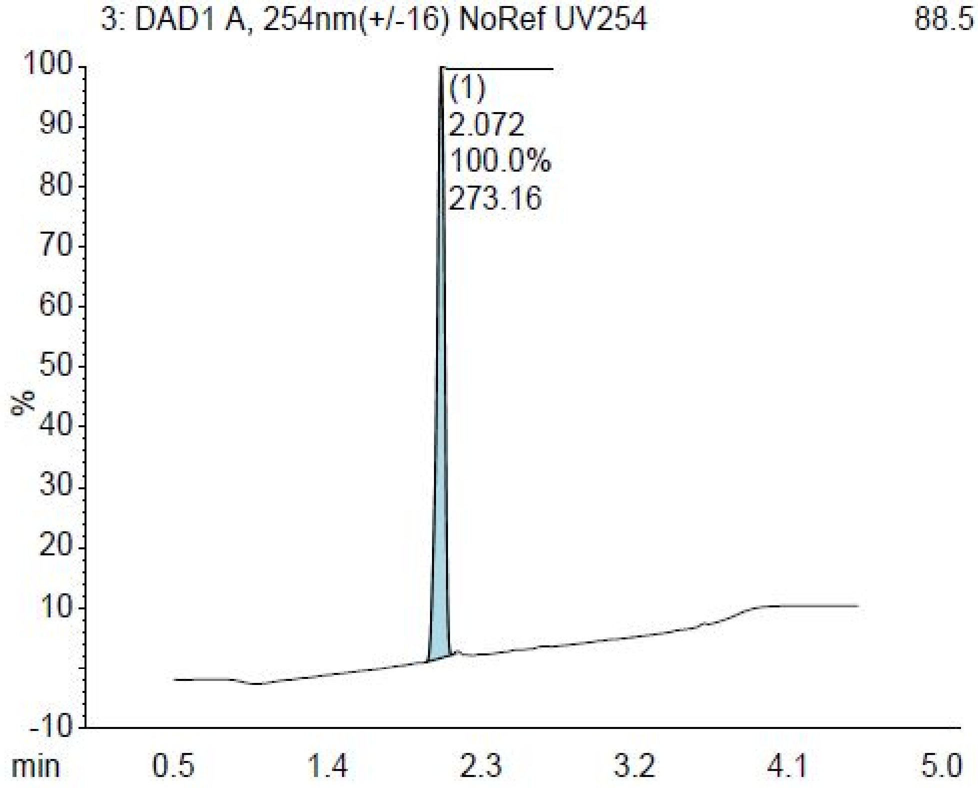

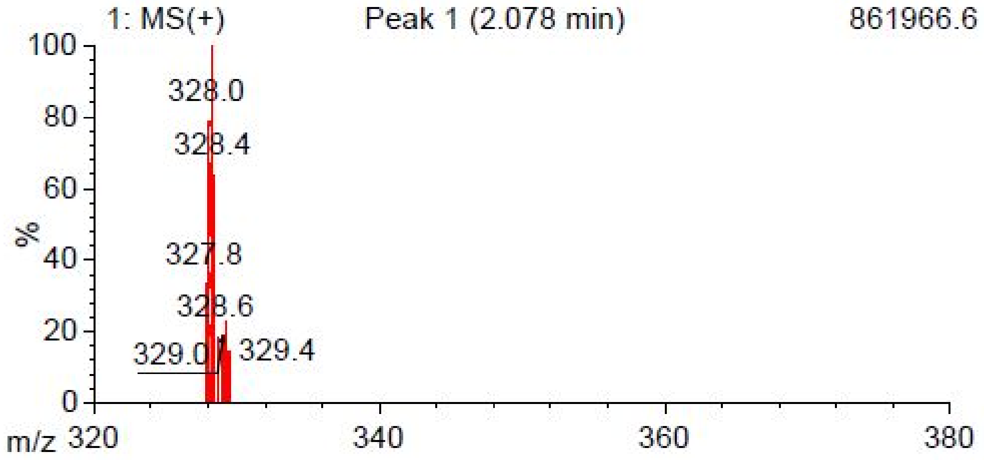

^{1}H spectra for compound 24

EXP-18- GM7381

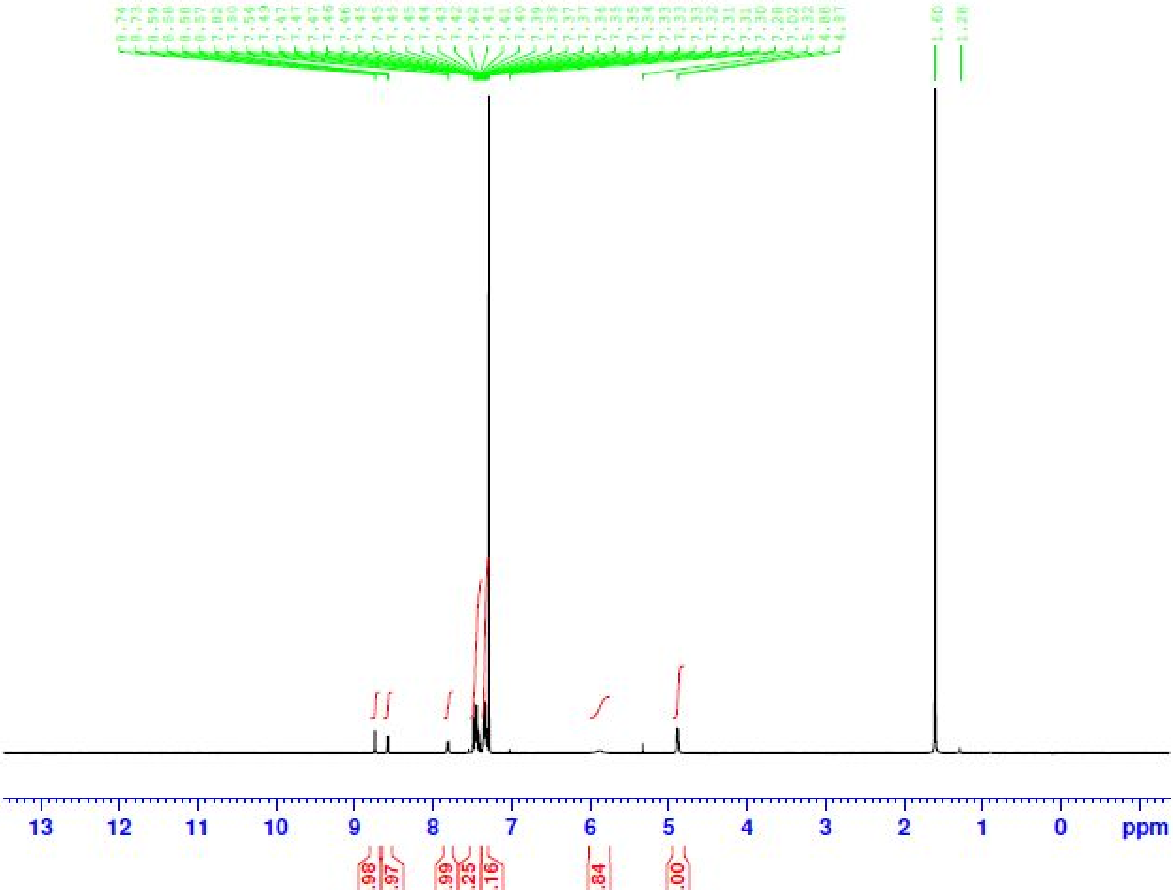

LCMS spectra for compound 24

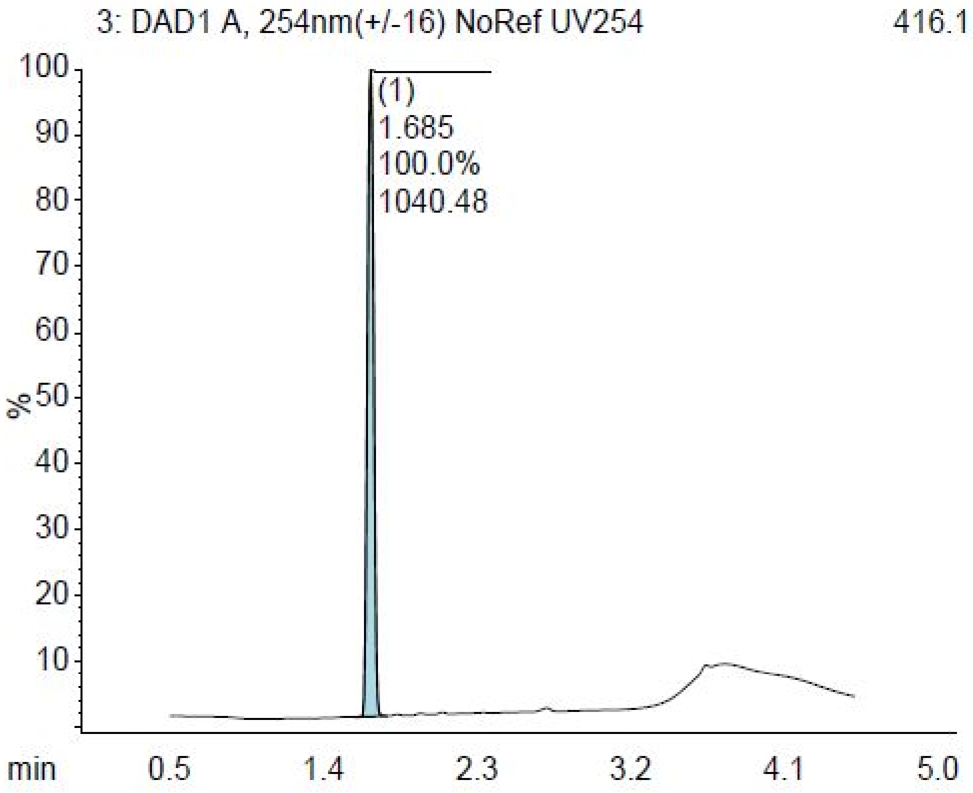

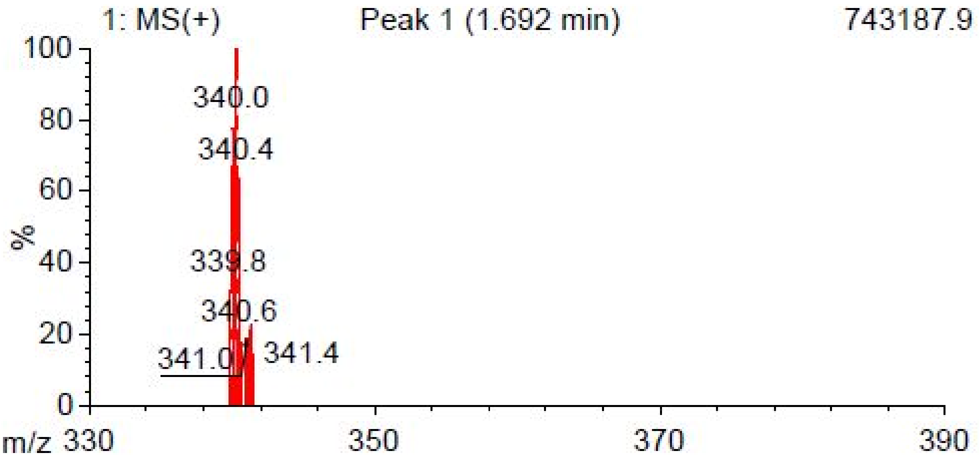

¹H spectra for compound 25

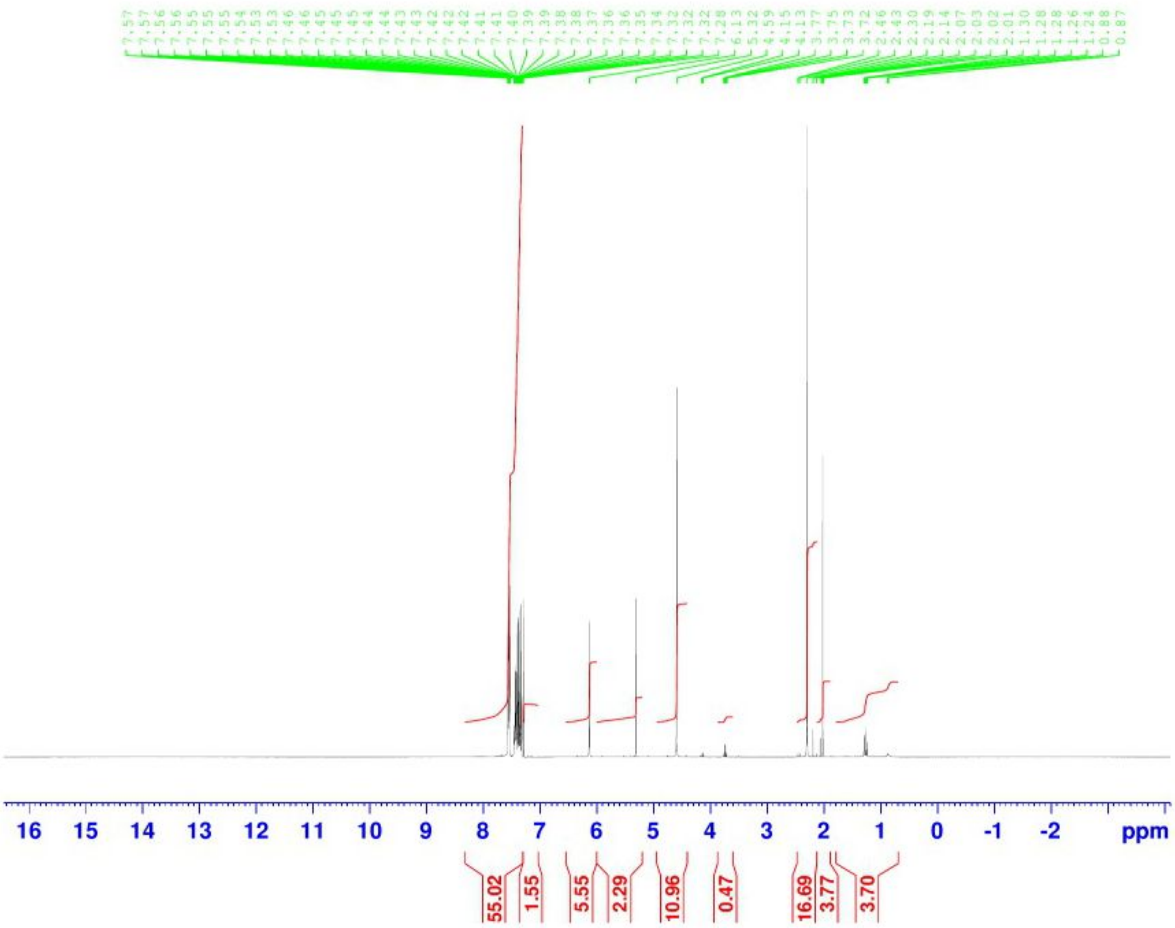

LCMS spectra for compound 25

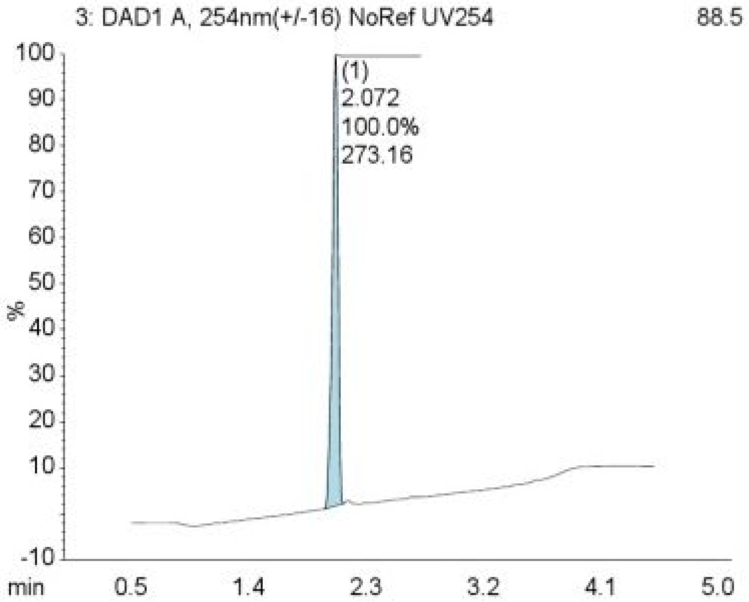

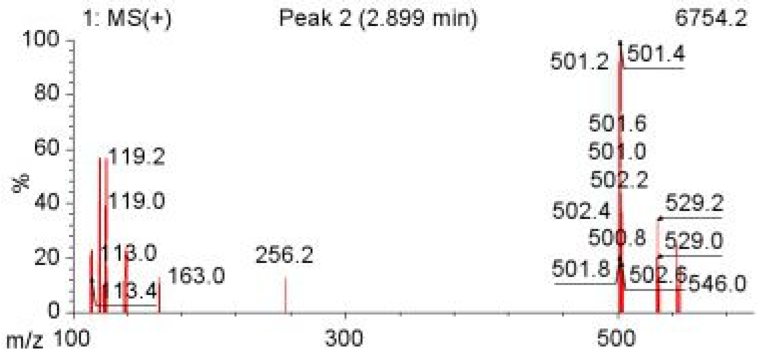

^{1}H spectra for compound 26

EXP- GX8588A

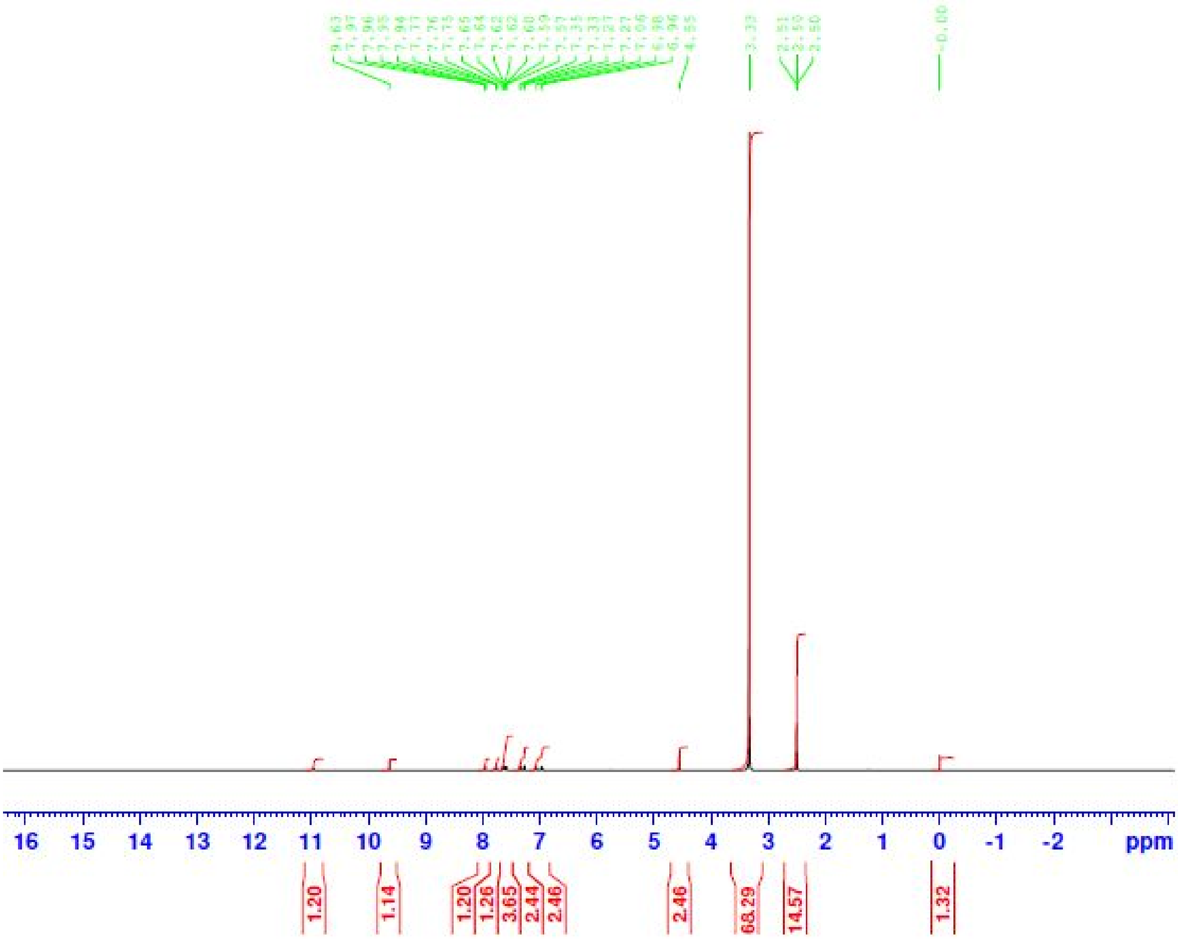

LCMS spectra for compound 26

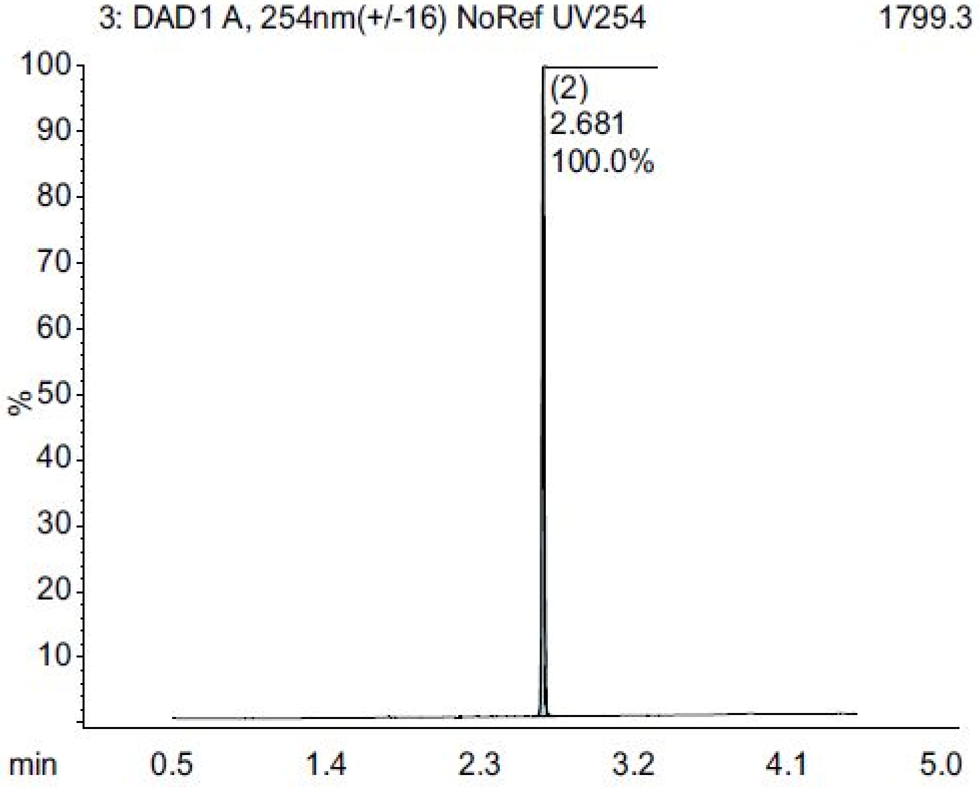

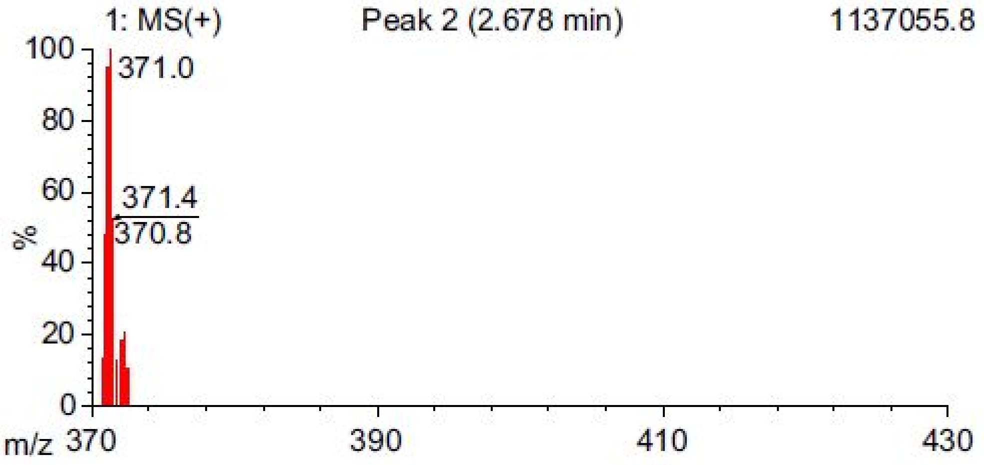

^{1}H spectra for compound 27

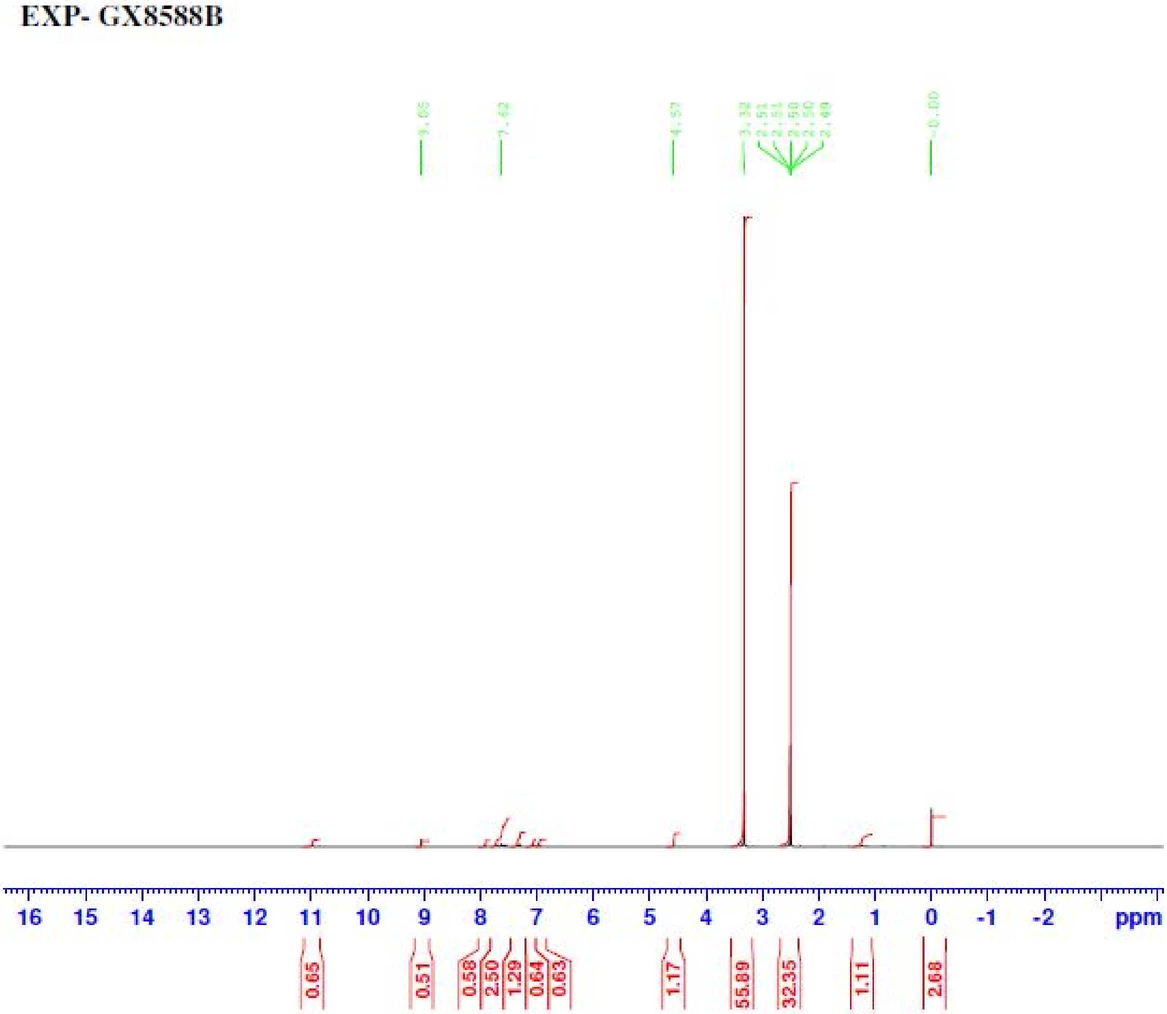

LCMS spectra for compound 27

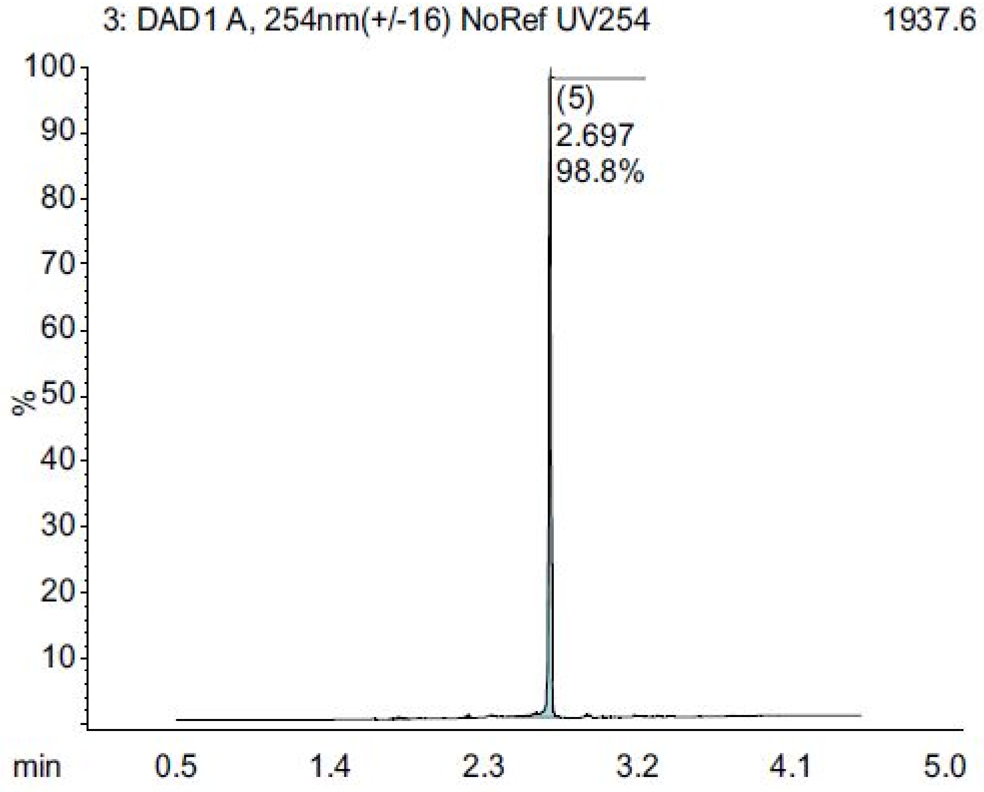

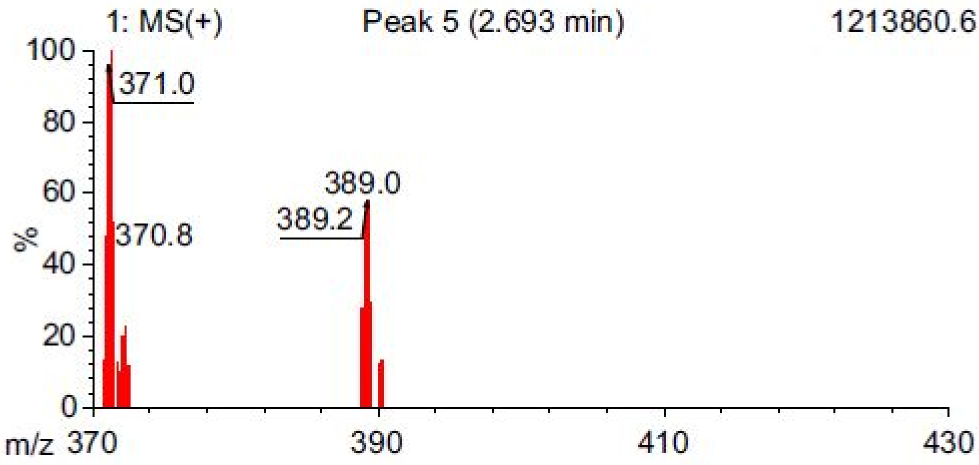

^{1}H NMR spectra for compound 28

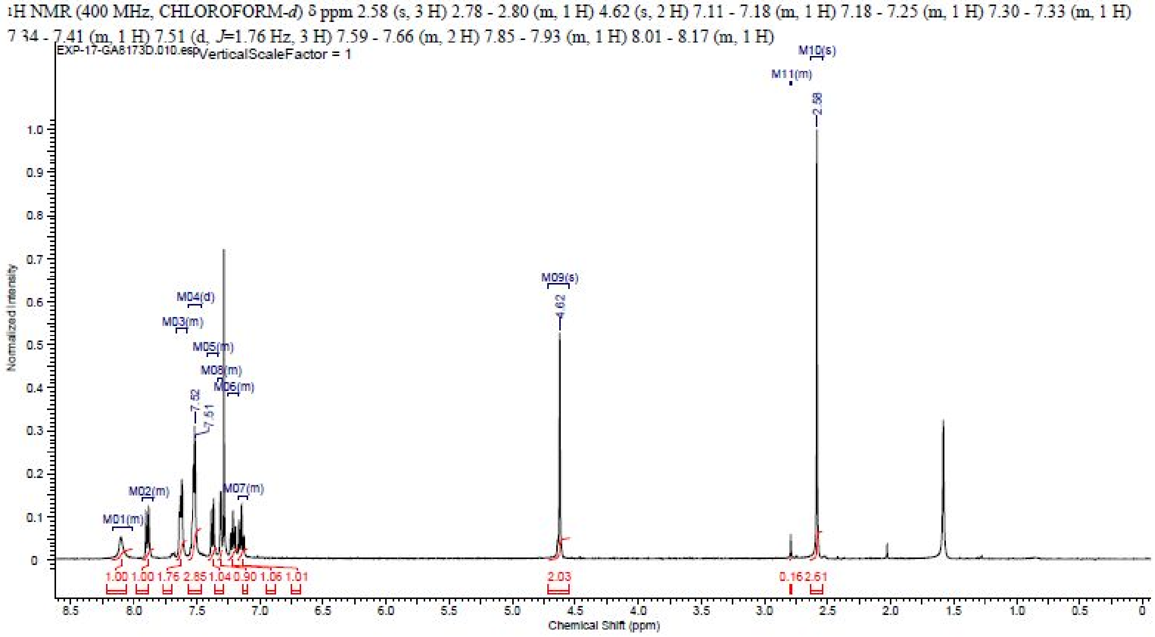

LC-MS spectra for compound 28

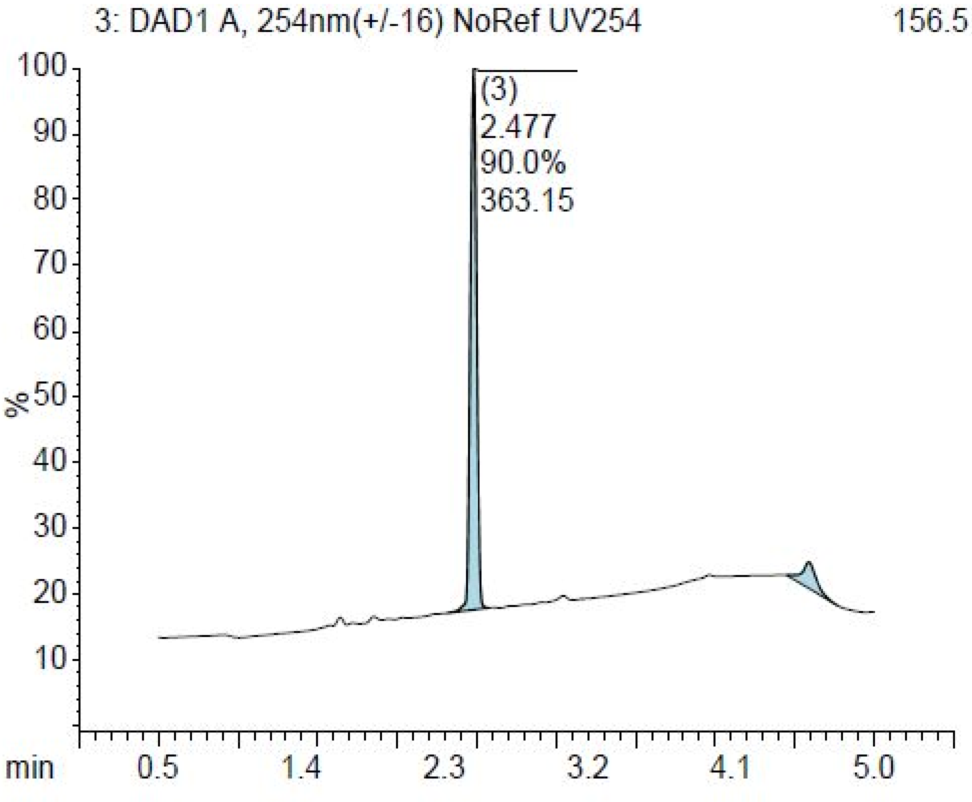

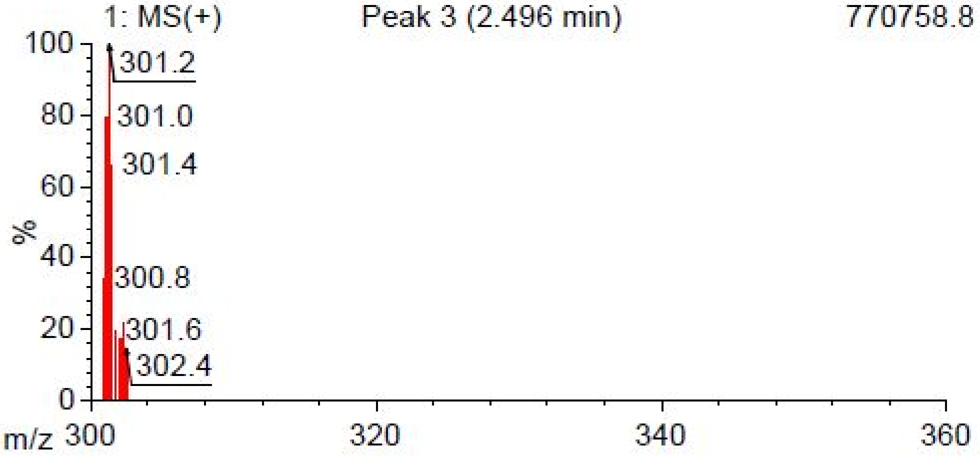

^{1}H NMR spectra for compound 29

^{1}H NMR (400 MHz, CHLOROFORM-*d*) δ ppm 2.79 (s, 3 H) 4.64 (s, 2 H) 7.11 - 7.17 (m, 1 H) 7.17 - 7.24 (m, 1 H) 7.24 - 7.28 (m, 1 H) 7.32 - 7.40 (m, 1 H) 7.54 (d, *J*=1.76 Hz, 3 H) 7.63 - 7.73 (m, 2 H) 7.84 - 7.95 (m, 1 H) 8.06 - 8.27 (m, 1 H)

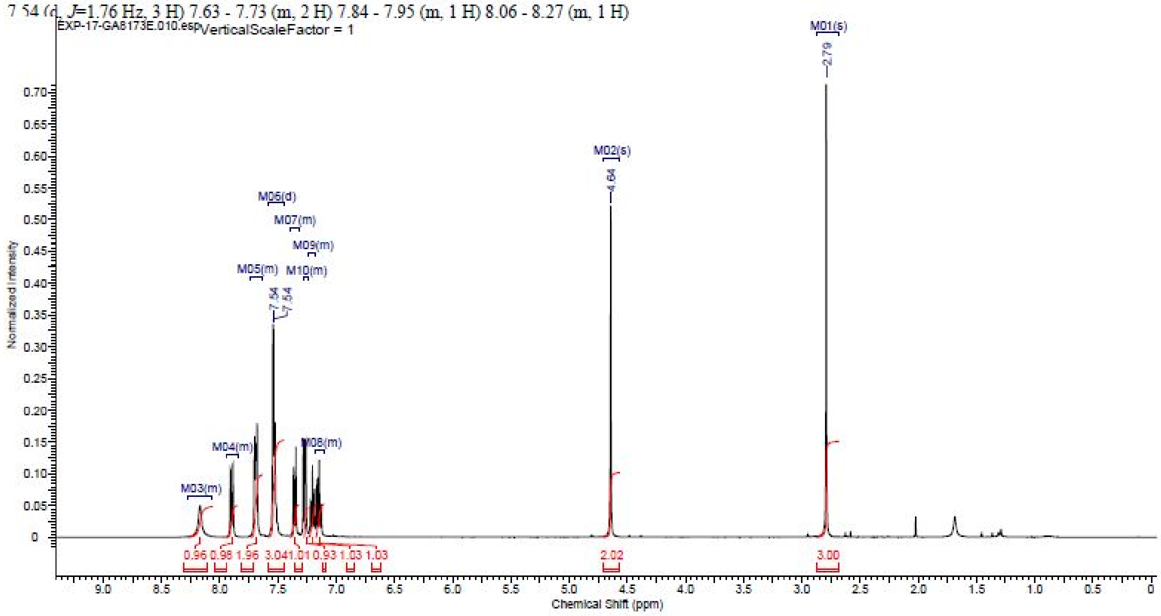

LC-MS spectra for compound 29

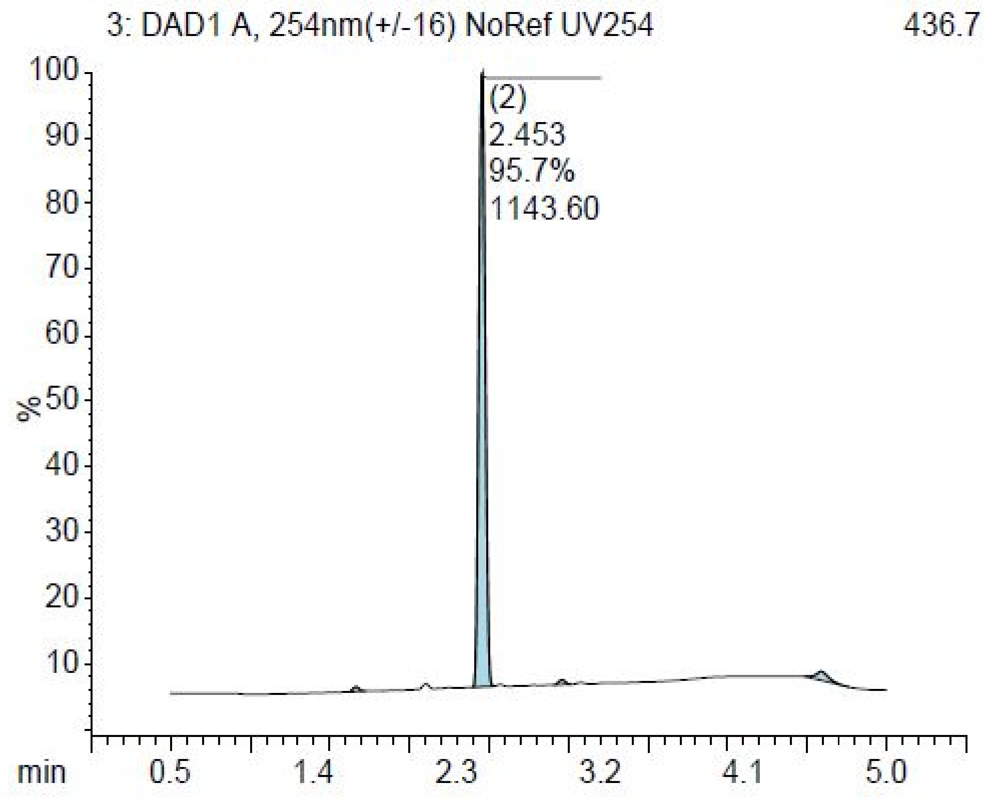

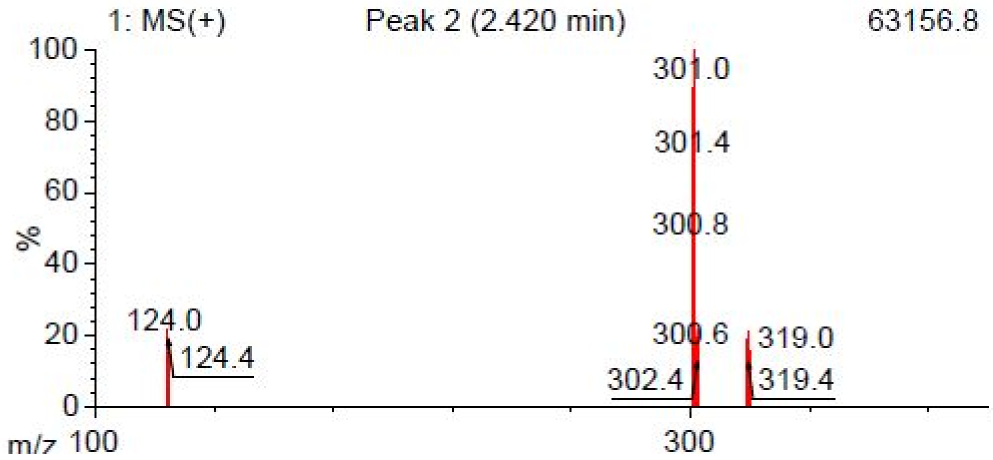

^{1}H NMR spectra for compound 30

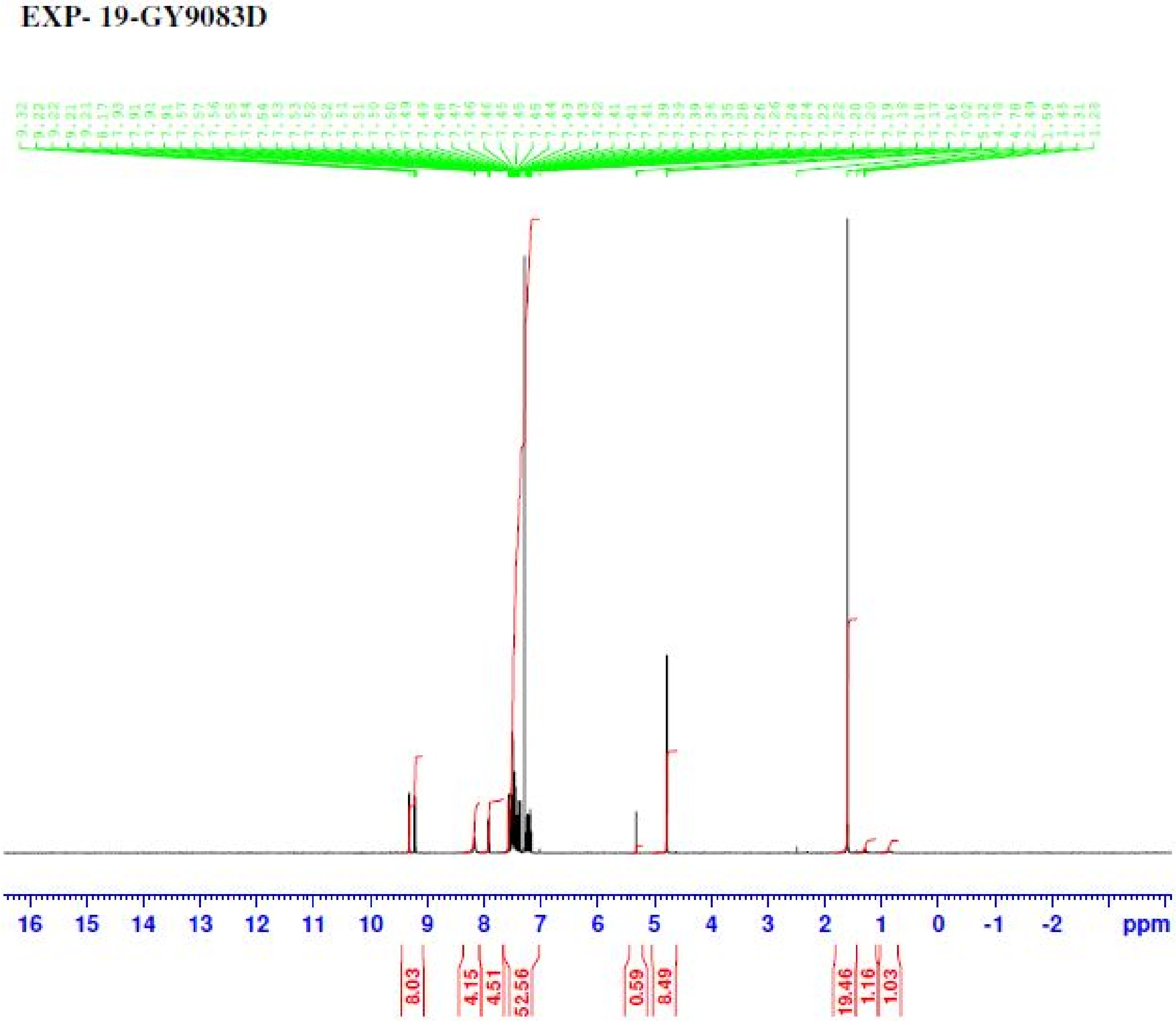

LCMS spectra for compound 30

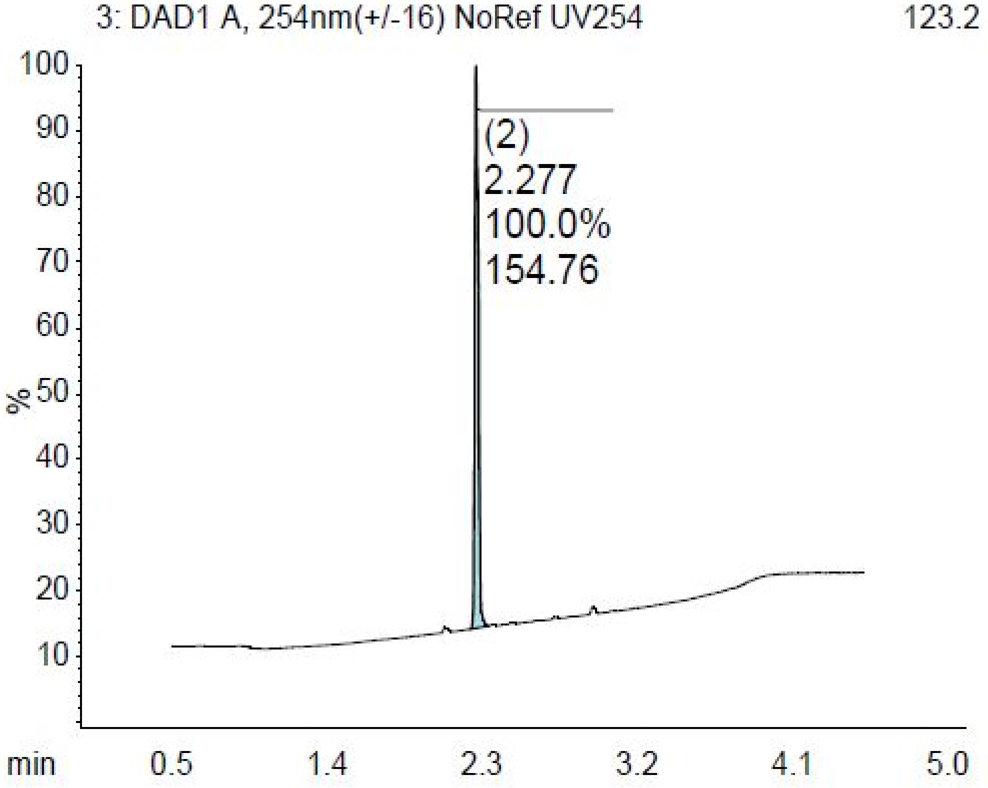

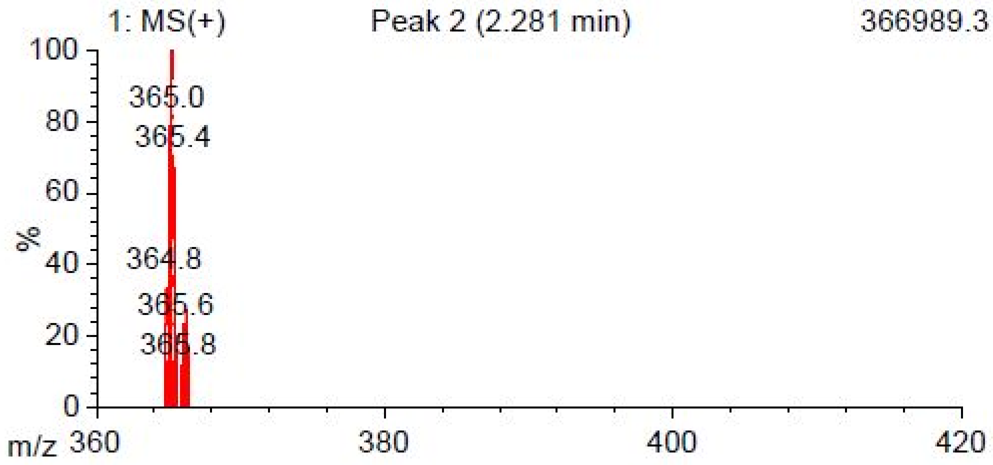

^{1}H NMR spectra for compound 31

EXP- 19-GY9082E

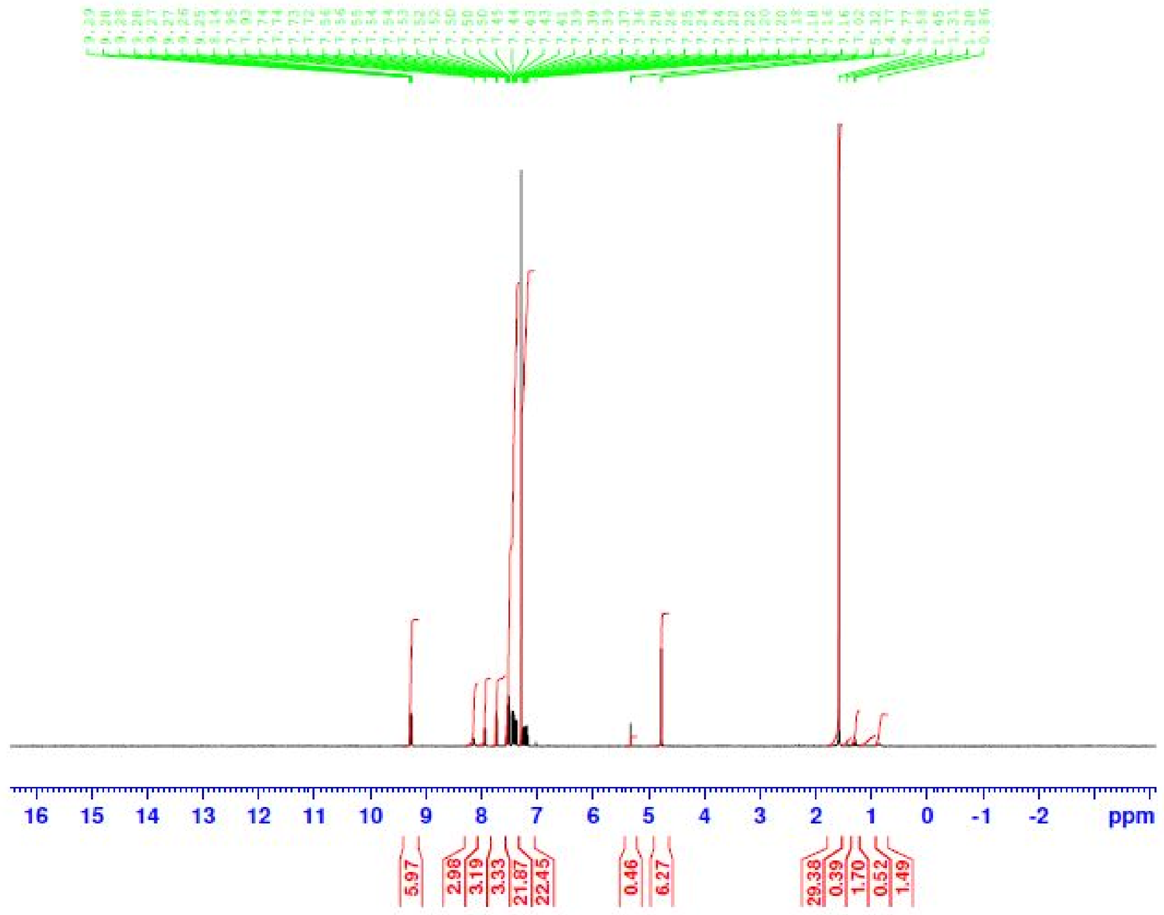

LCMS spectra for compound 31

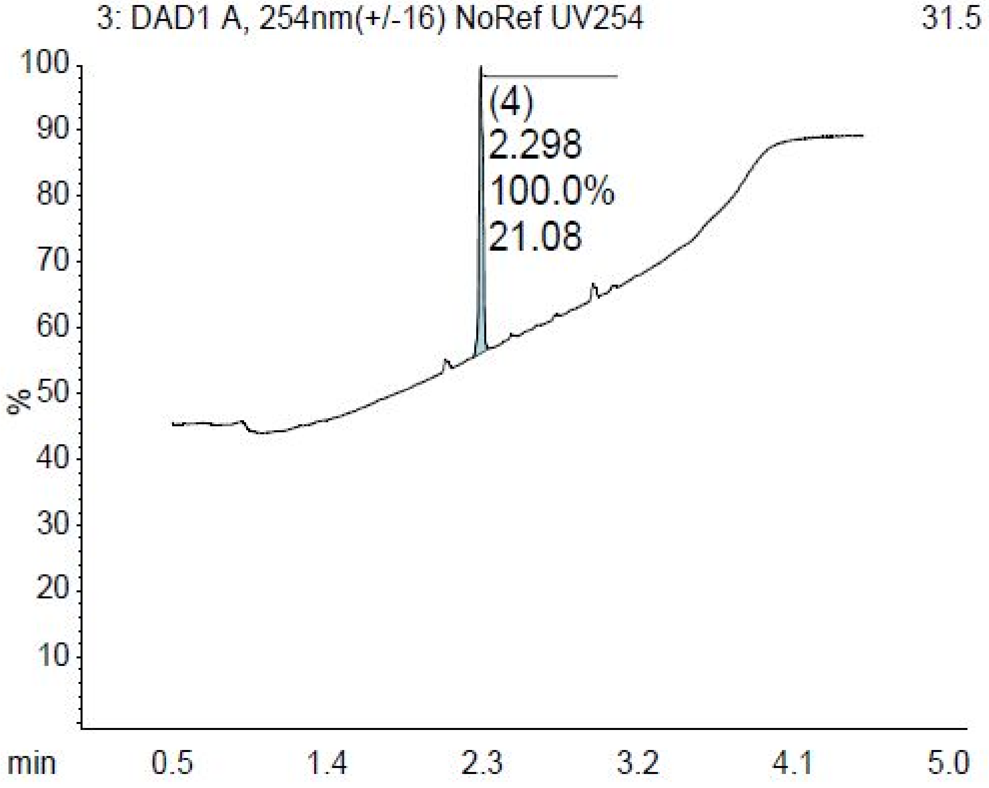

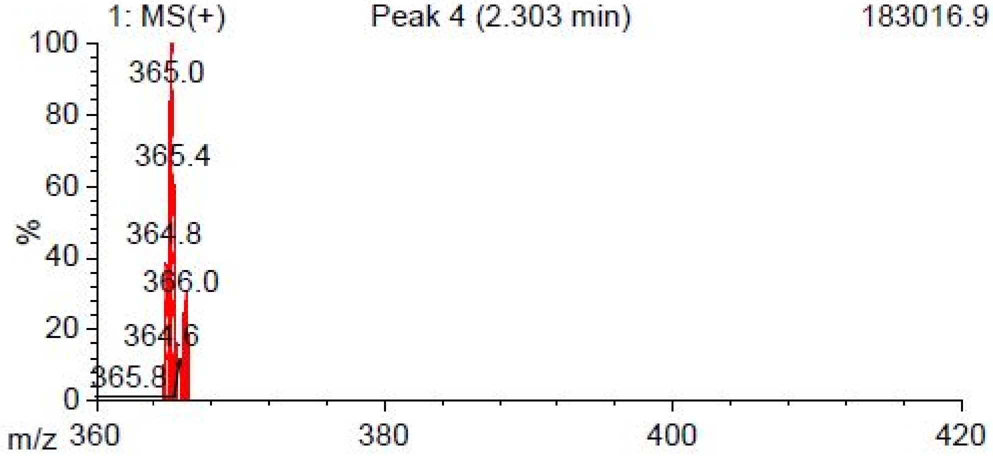

^{1}H NMR spectra for compound 32

NMR EXP-18-GY9064B

email_EXP-18-GY9064B_10_1.pdf

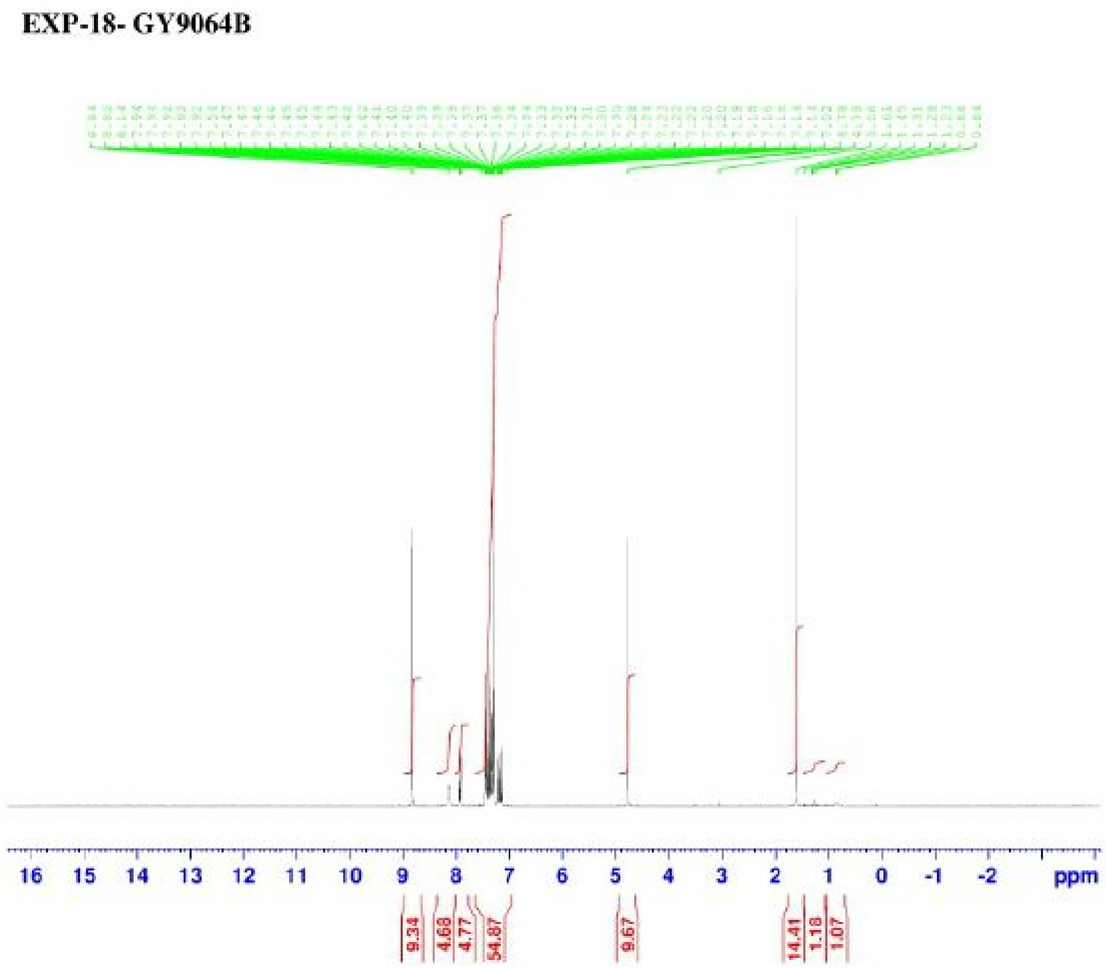

LCMS spectra for compound 32

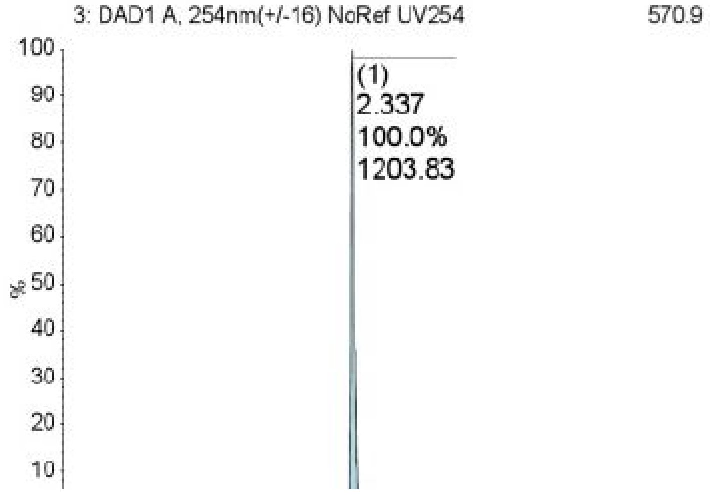

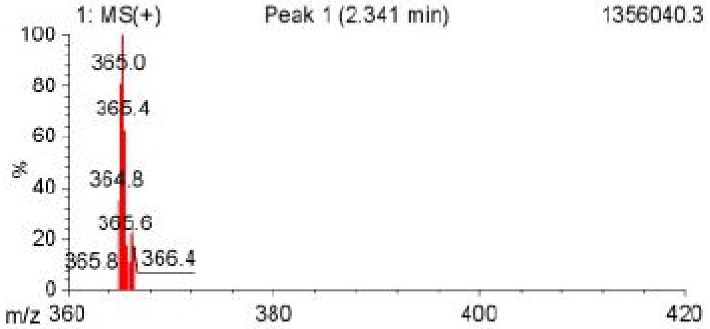

^{1}H NMR spectra for compound 33

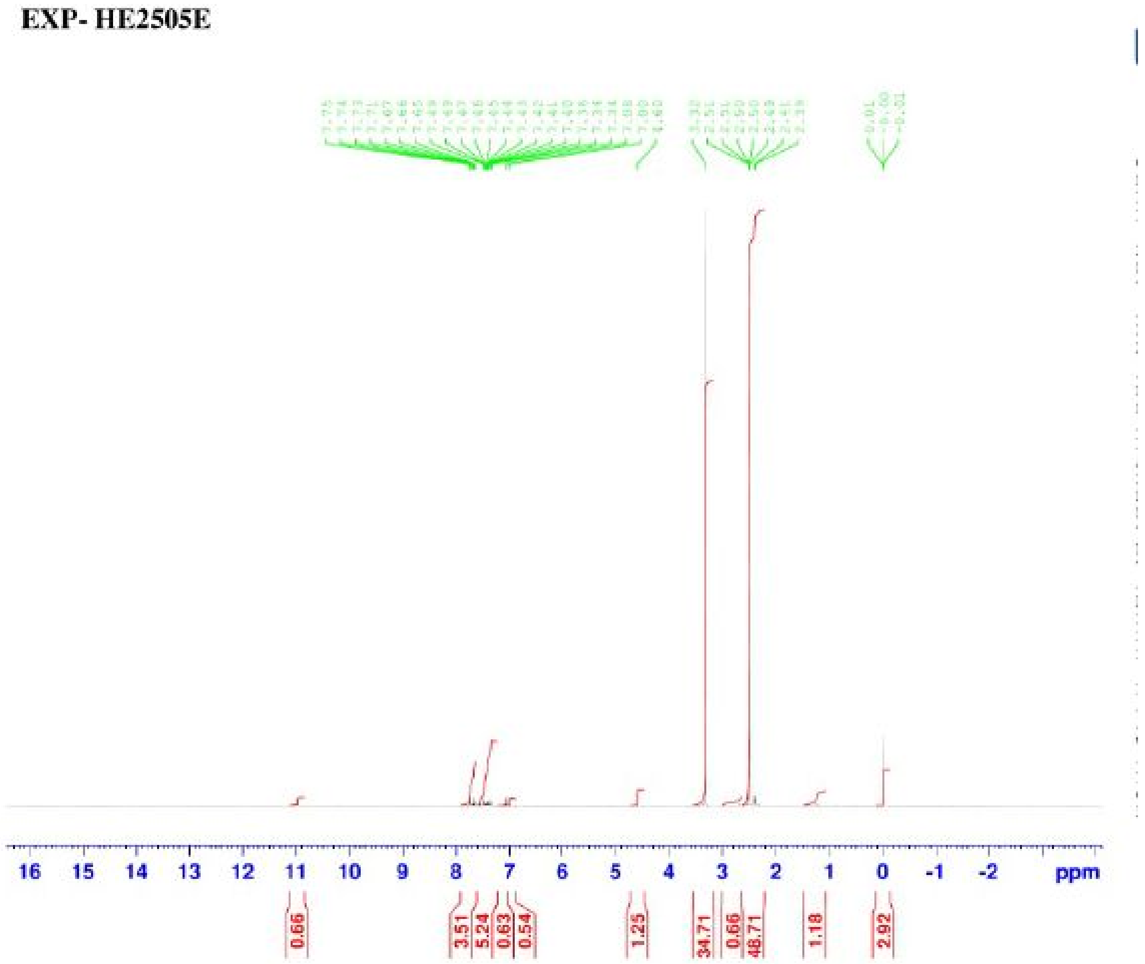

LCMS spectra for compound 33

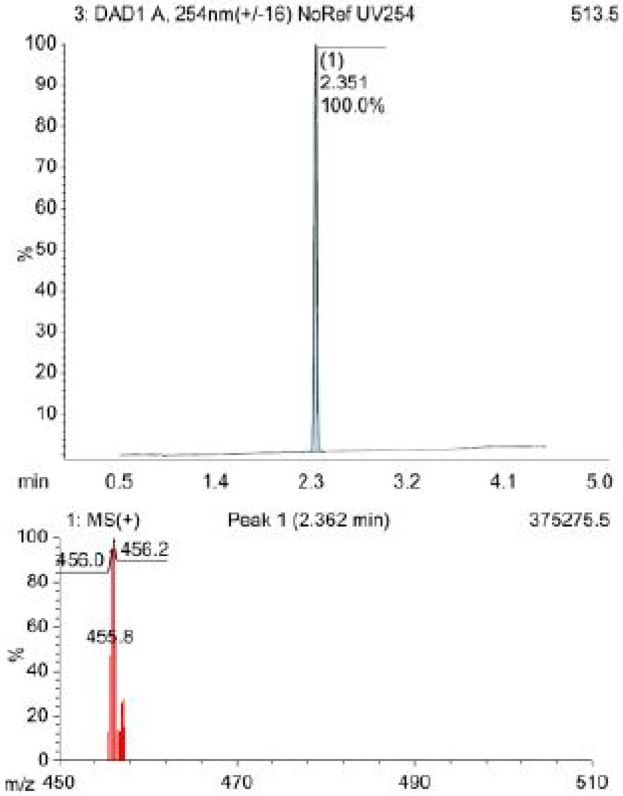

^{1}H NMR spectra for compound 34

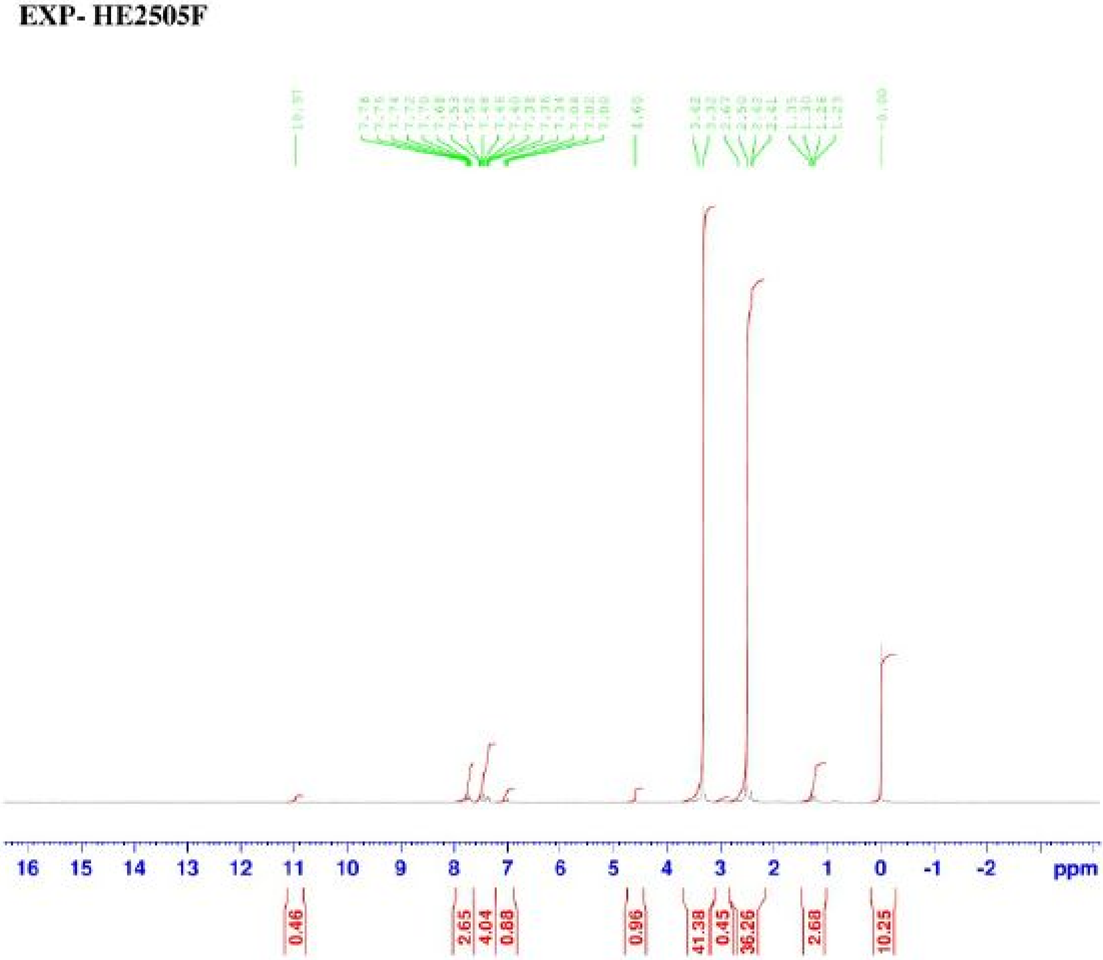

LCMS spectra for compound 34

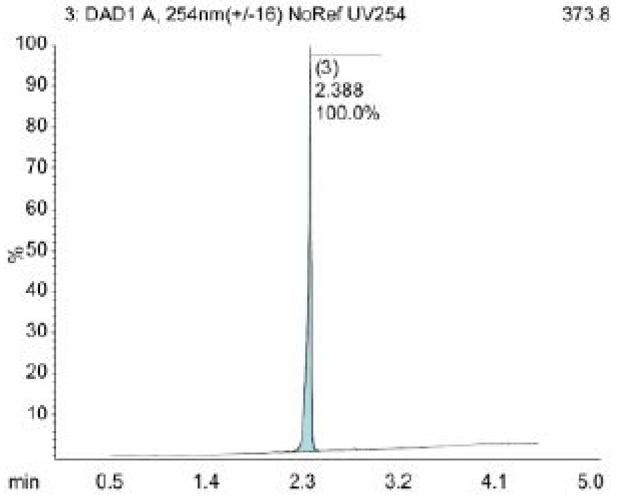

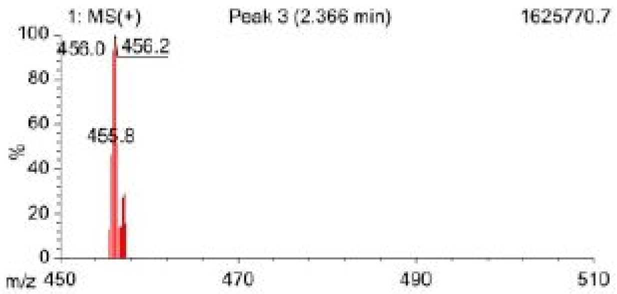

^{1}H NMR spectra for compound 35

EXP- HE2506E

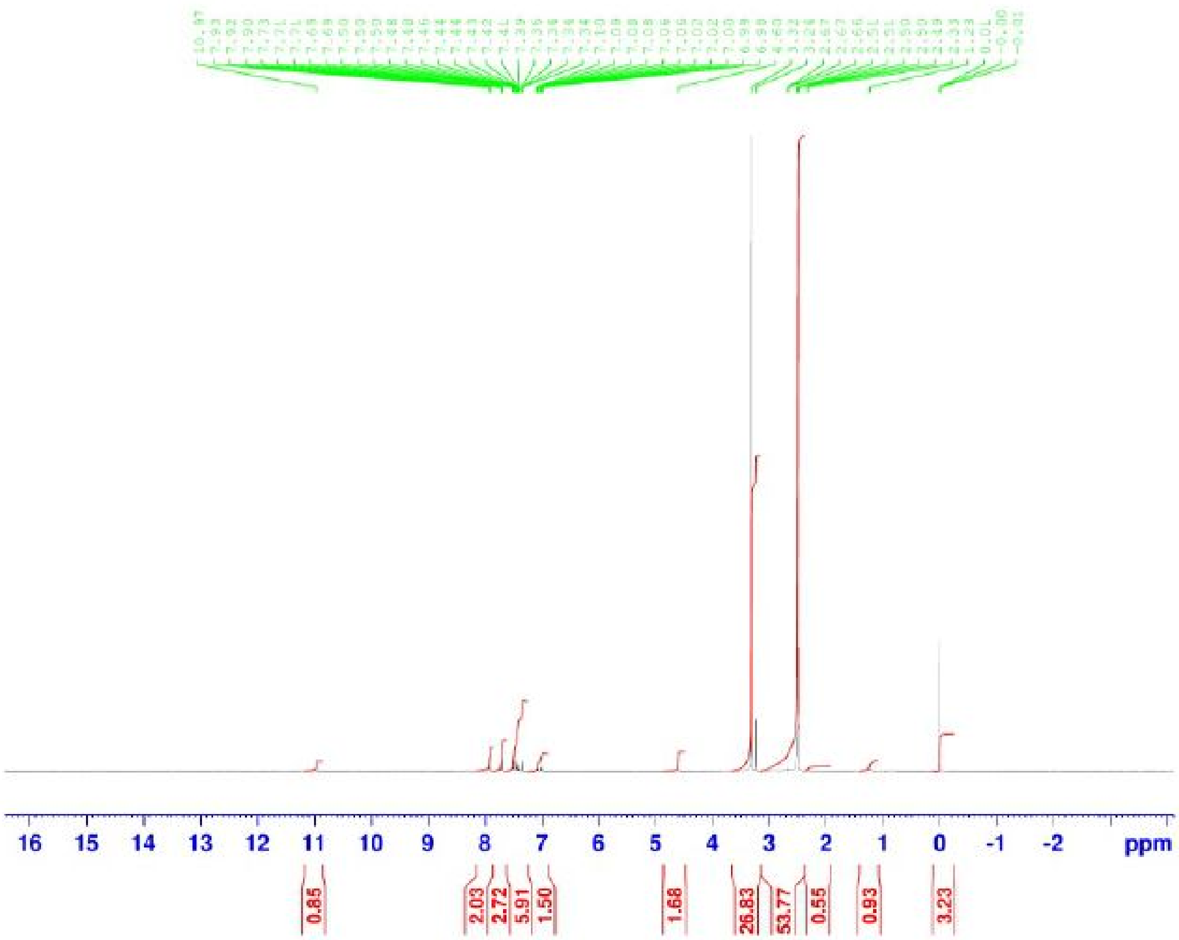

LCMS spectra for compound 35

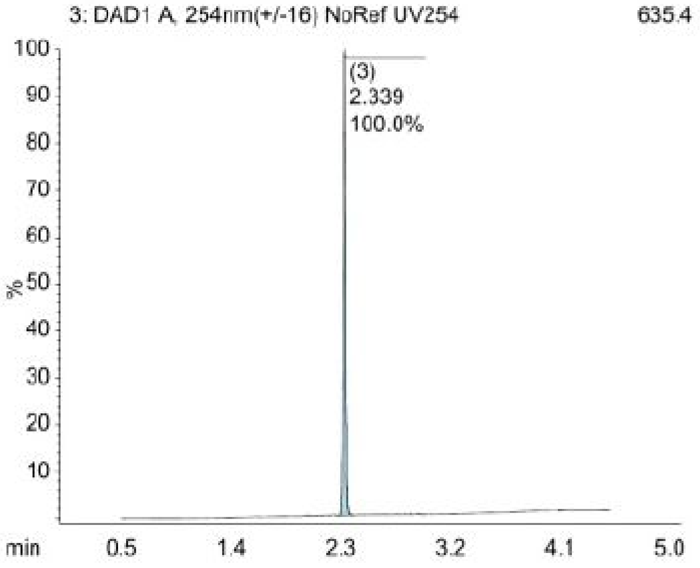

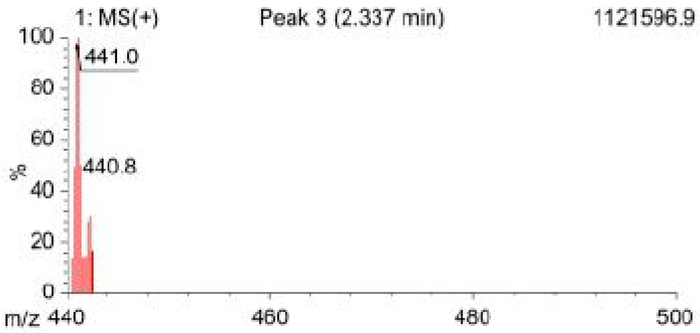

^{1}H NMR spectra for compound 36

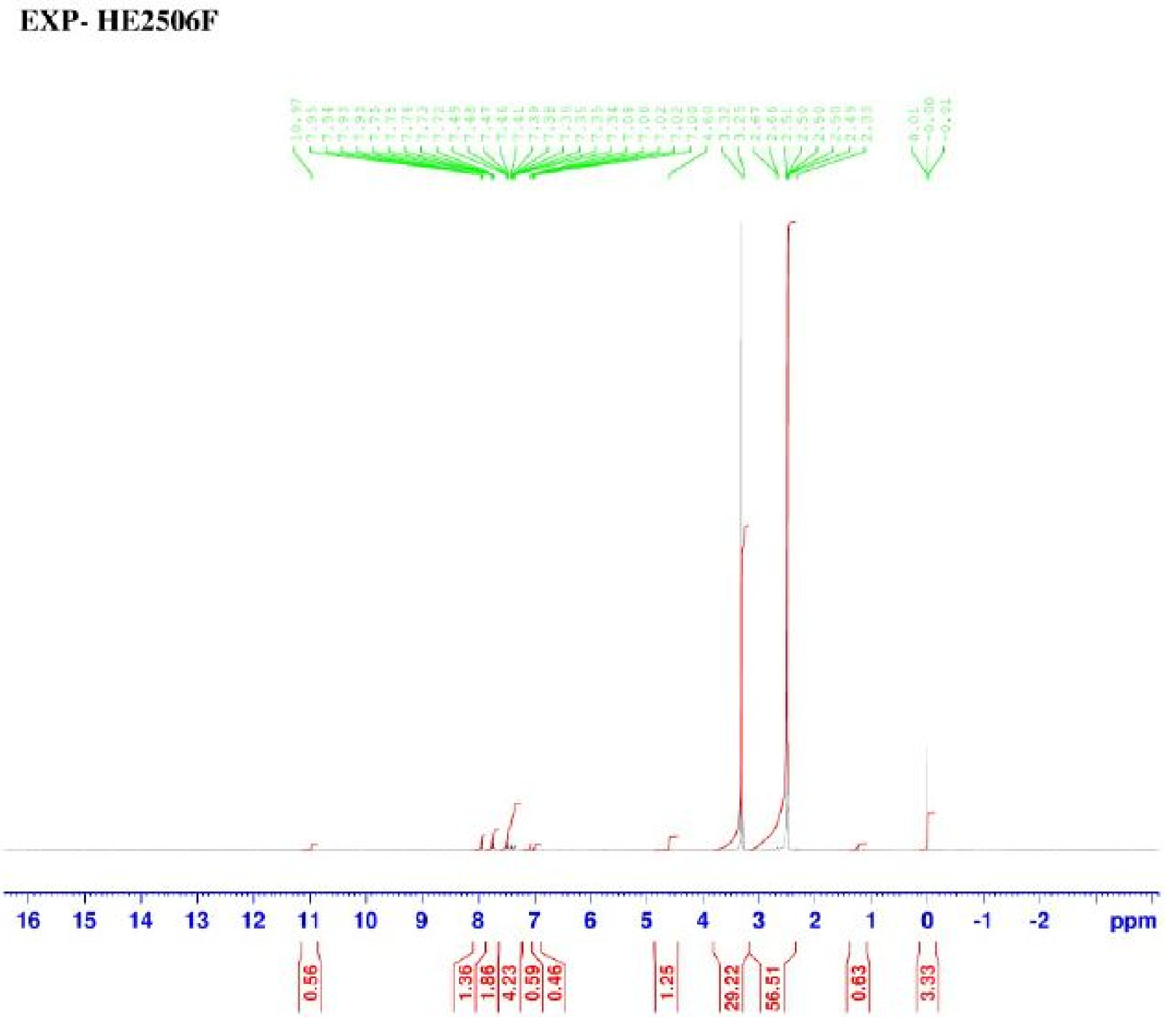

LCMS spectra for compound 36

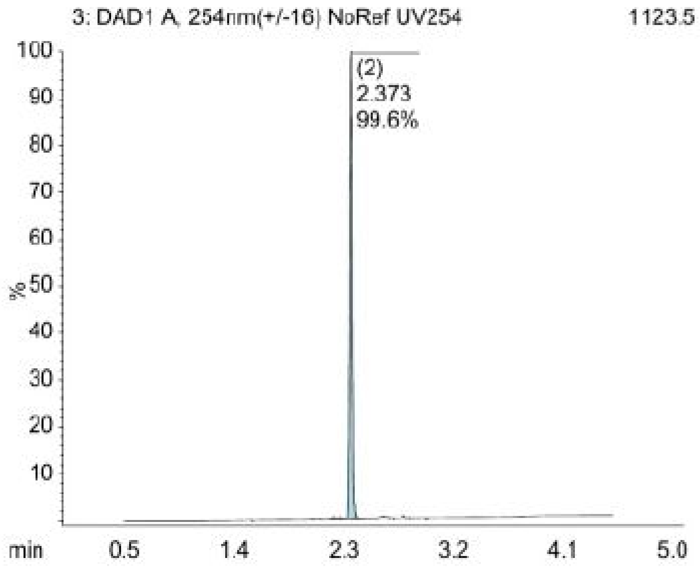

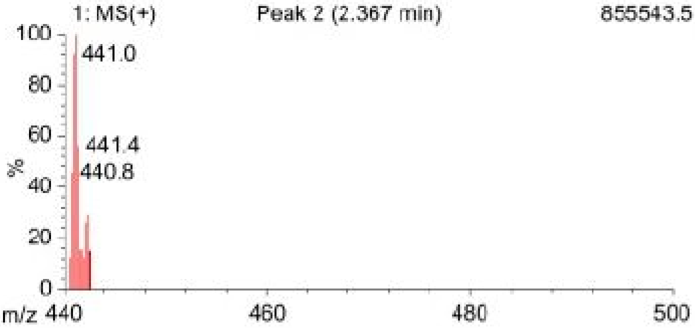

^{1}H NMR spectra for compound 37

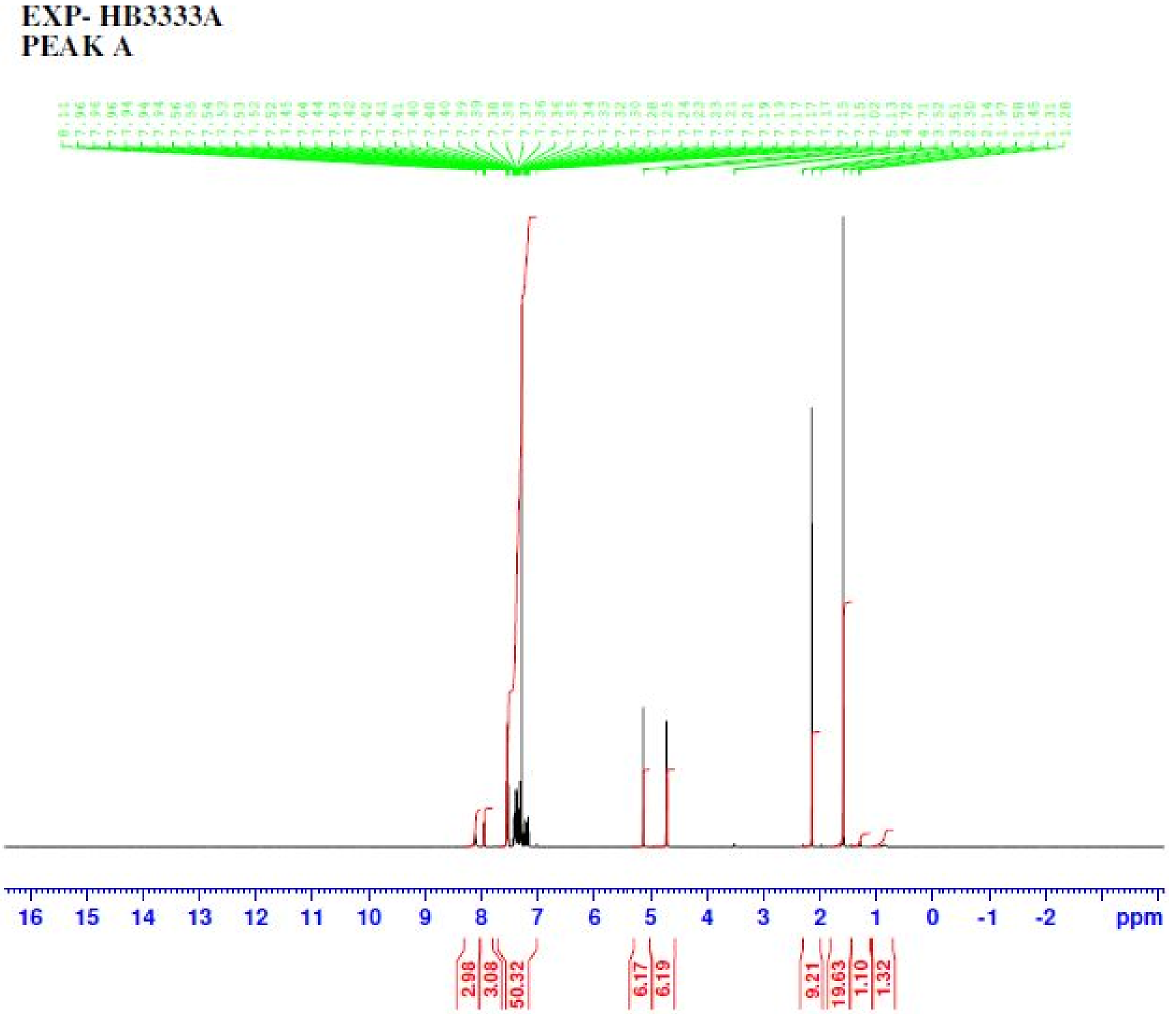

LCMS spectra for compound 37

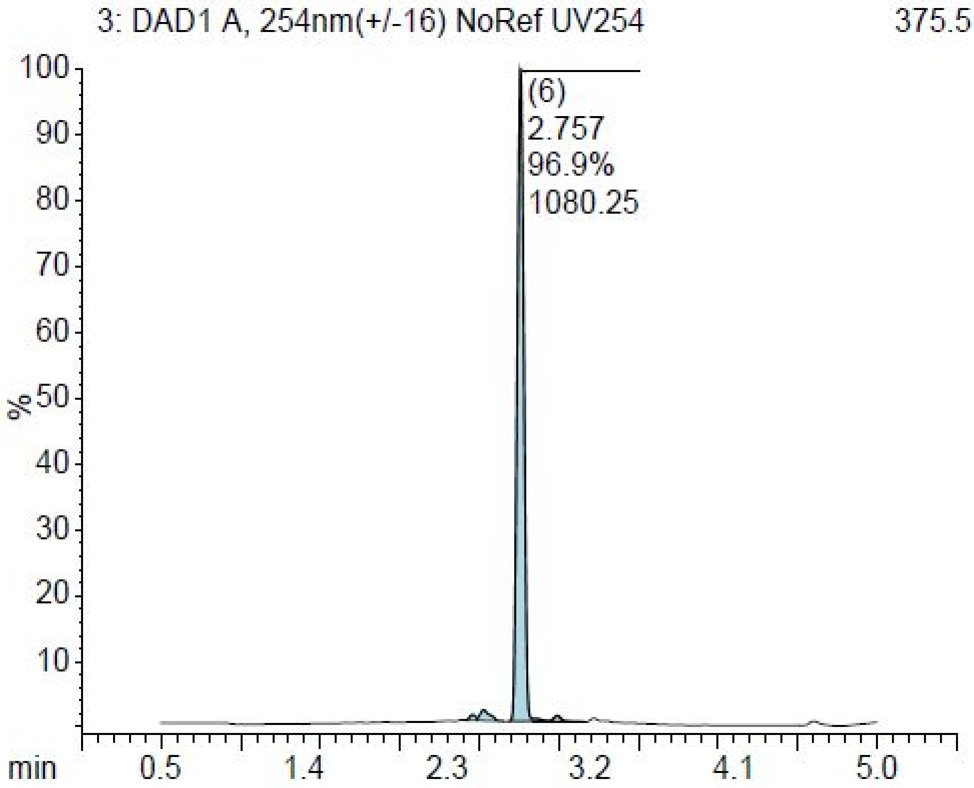

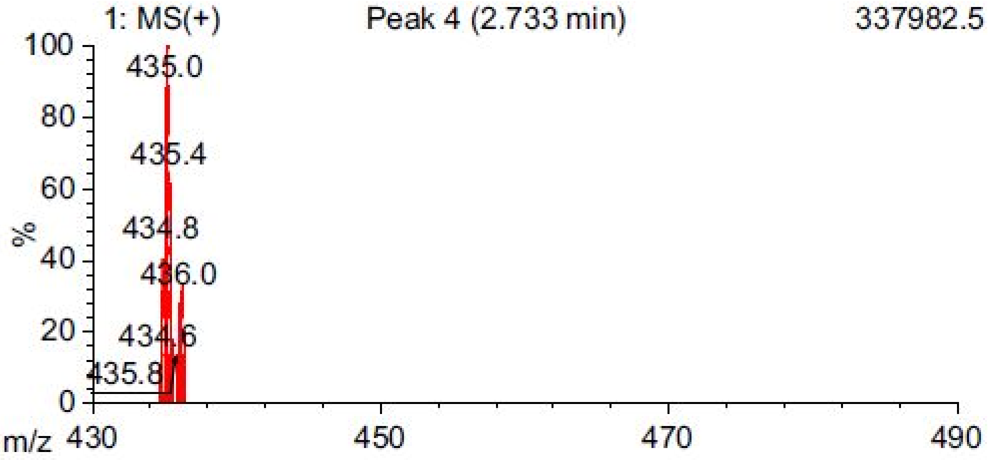

¹H NMR spectra for compound 38

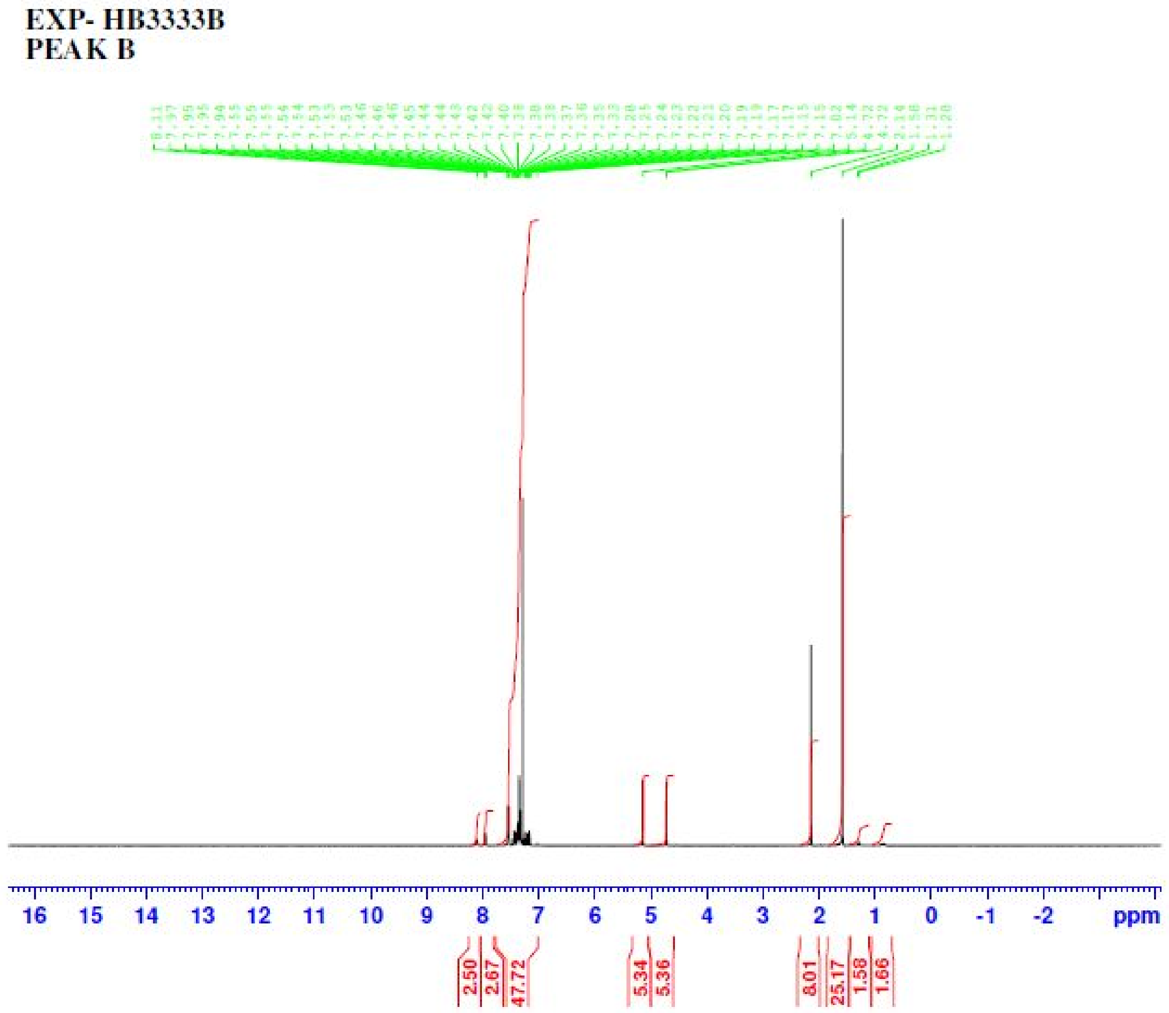

LCMS spectra for compound 38

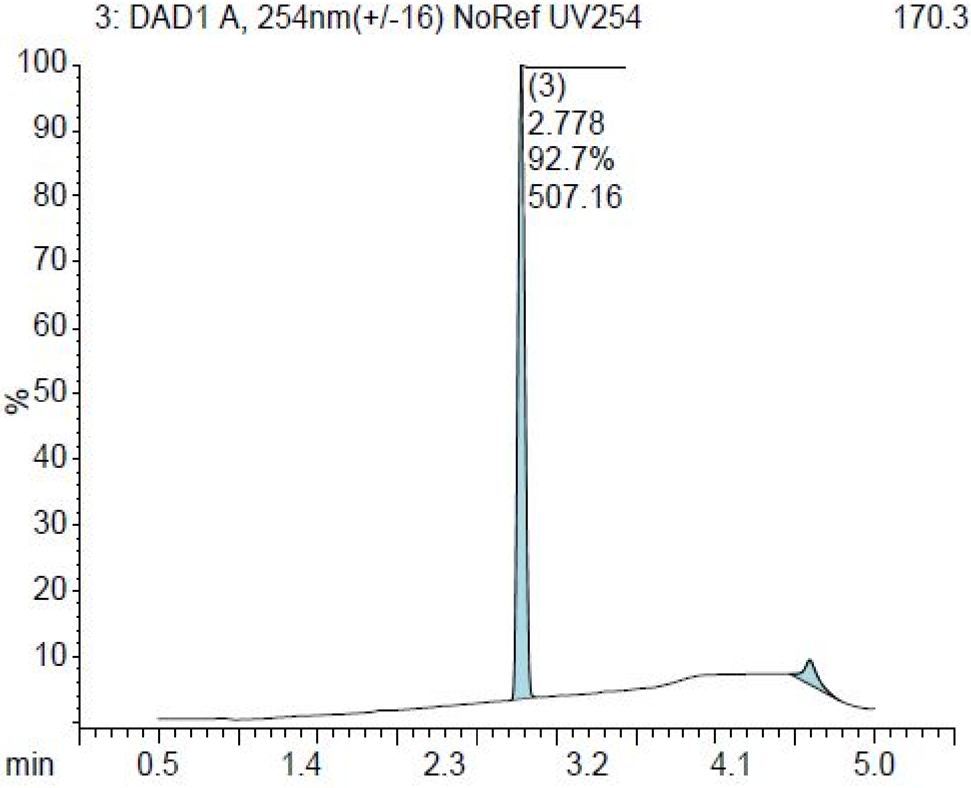

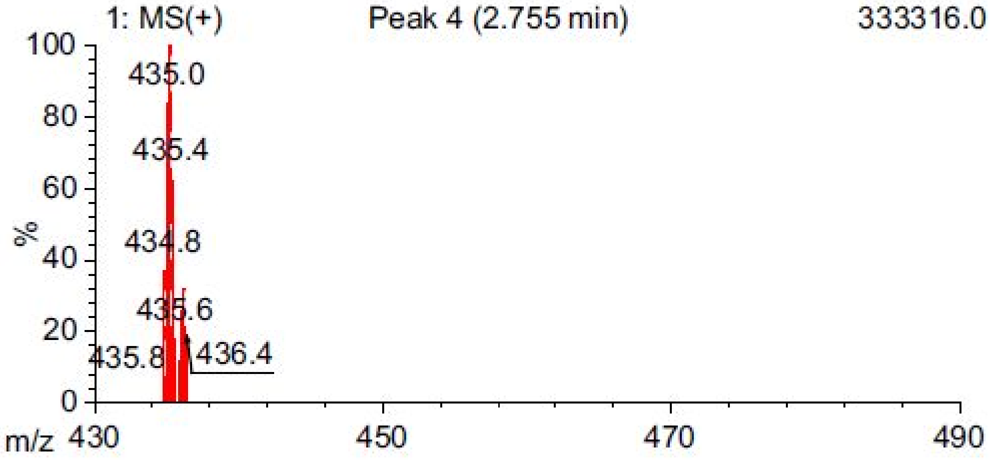

^{1}H NMR spectra for compound 39

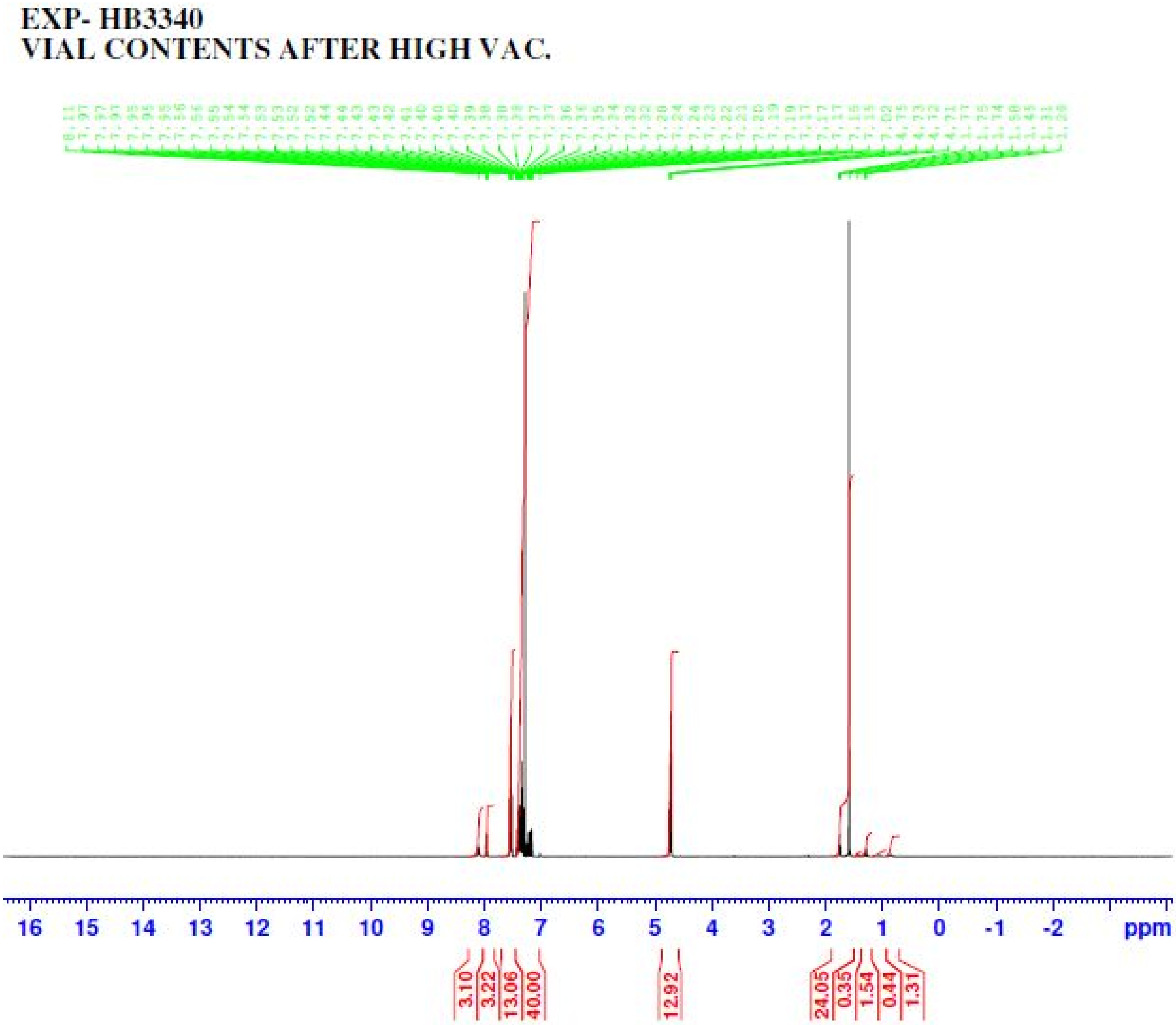

LCMS spectra for compound 39

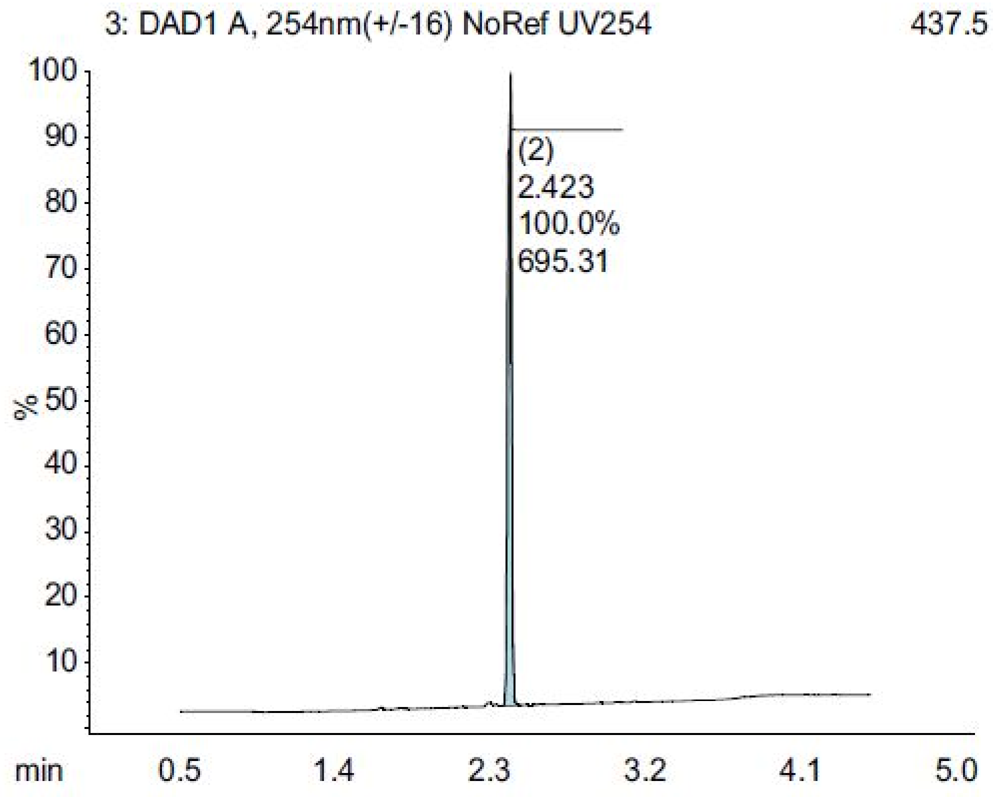

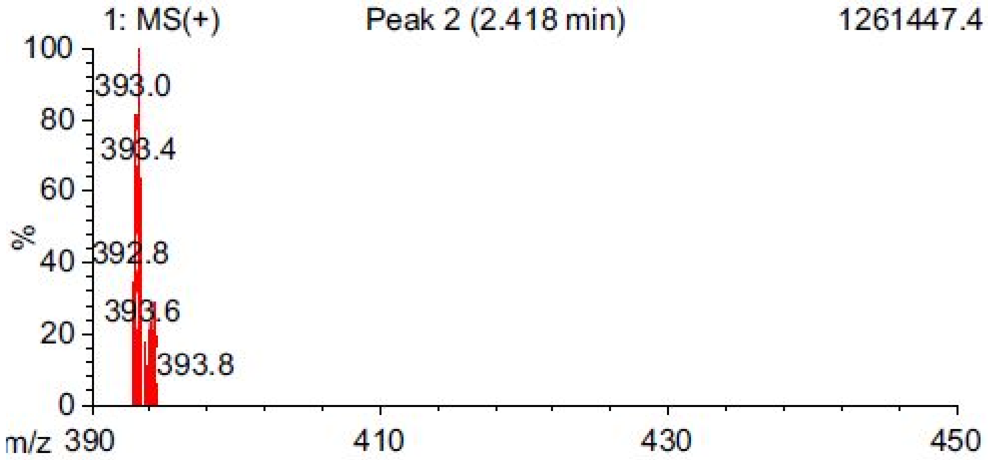

^{1}H NMR spectra for compound 40

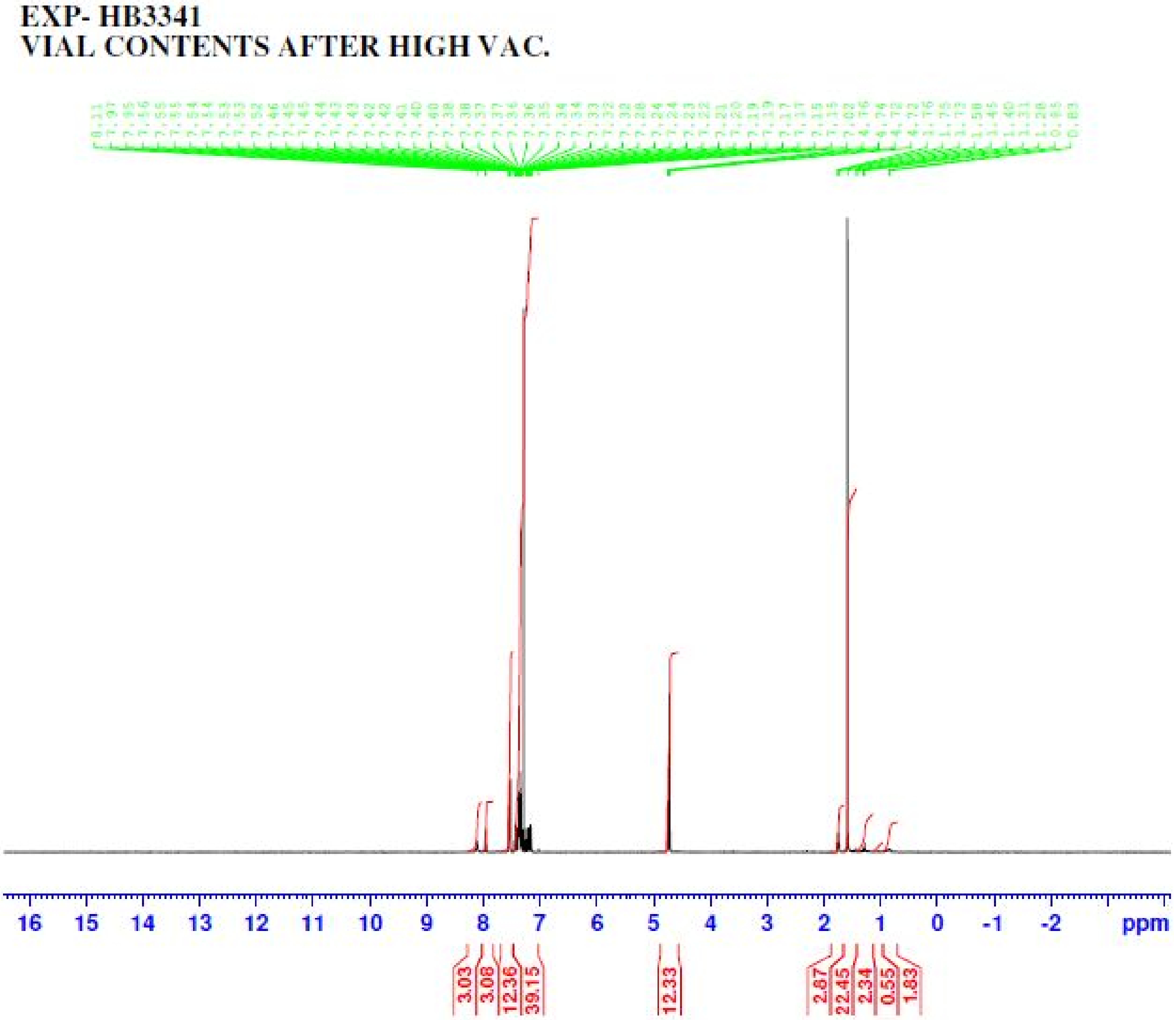

LCMS spectra for compound 40

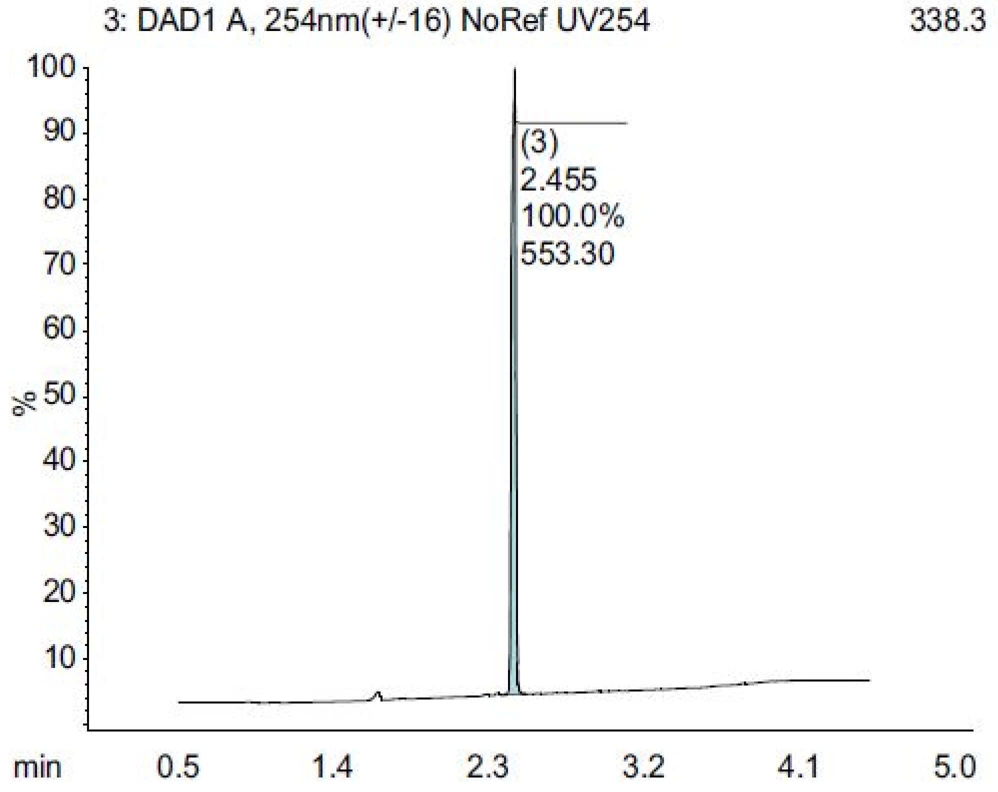

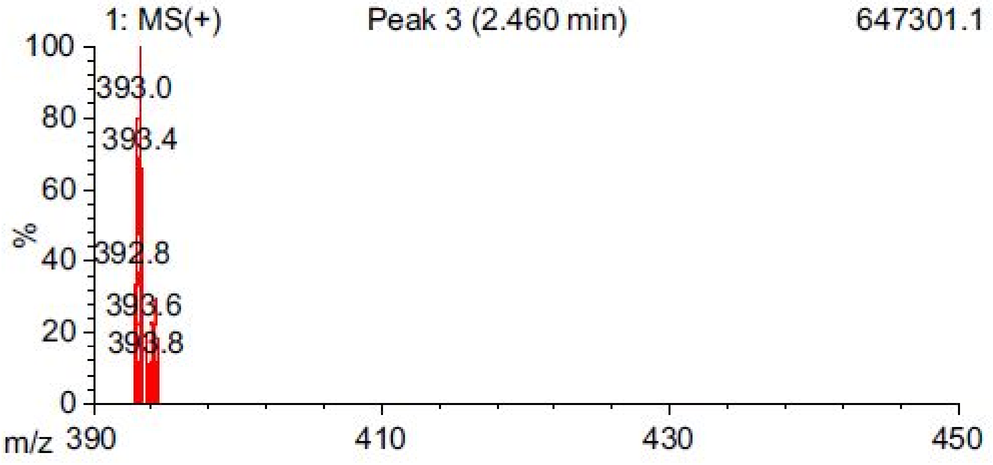

^{1}H NMR spectra for compound 41

EXP-19-HE9485A

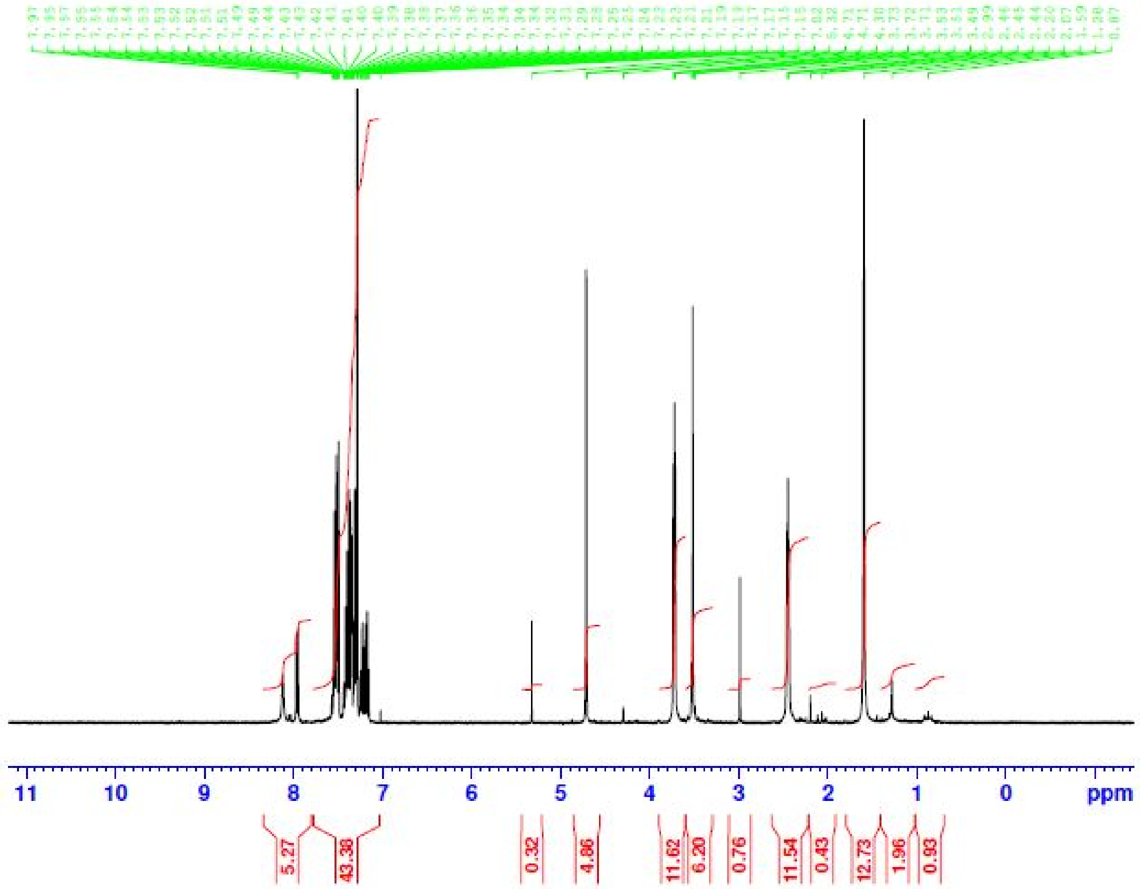

LCMS spectra for compound 41

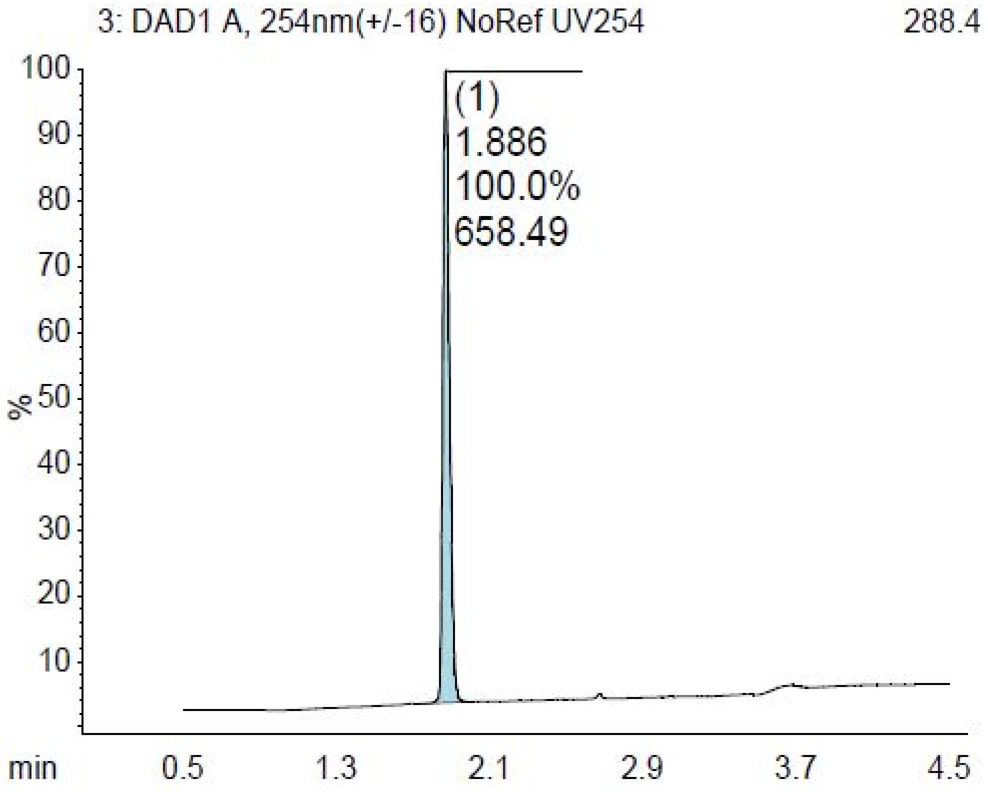

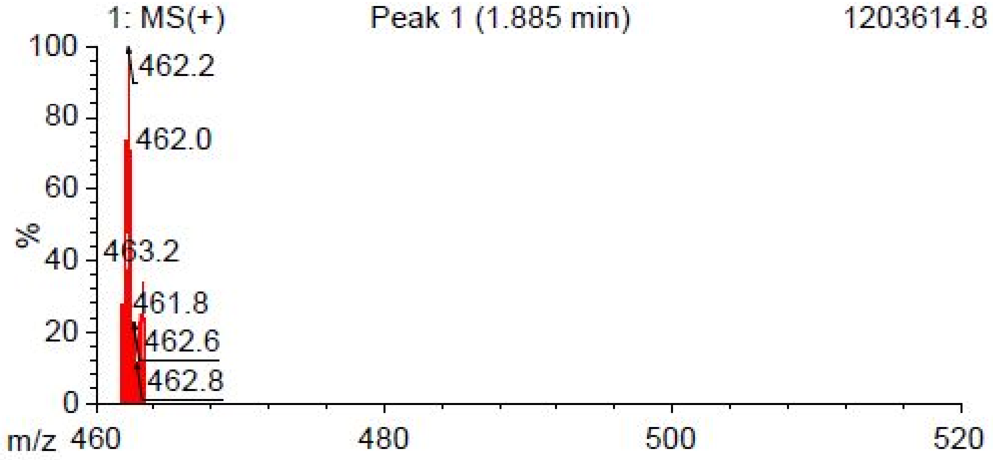

^{1}H NMR spectra for compound 42

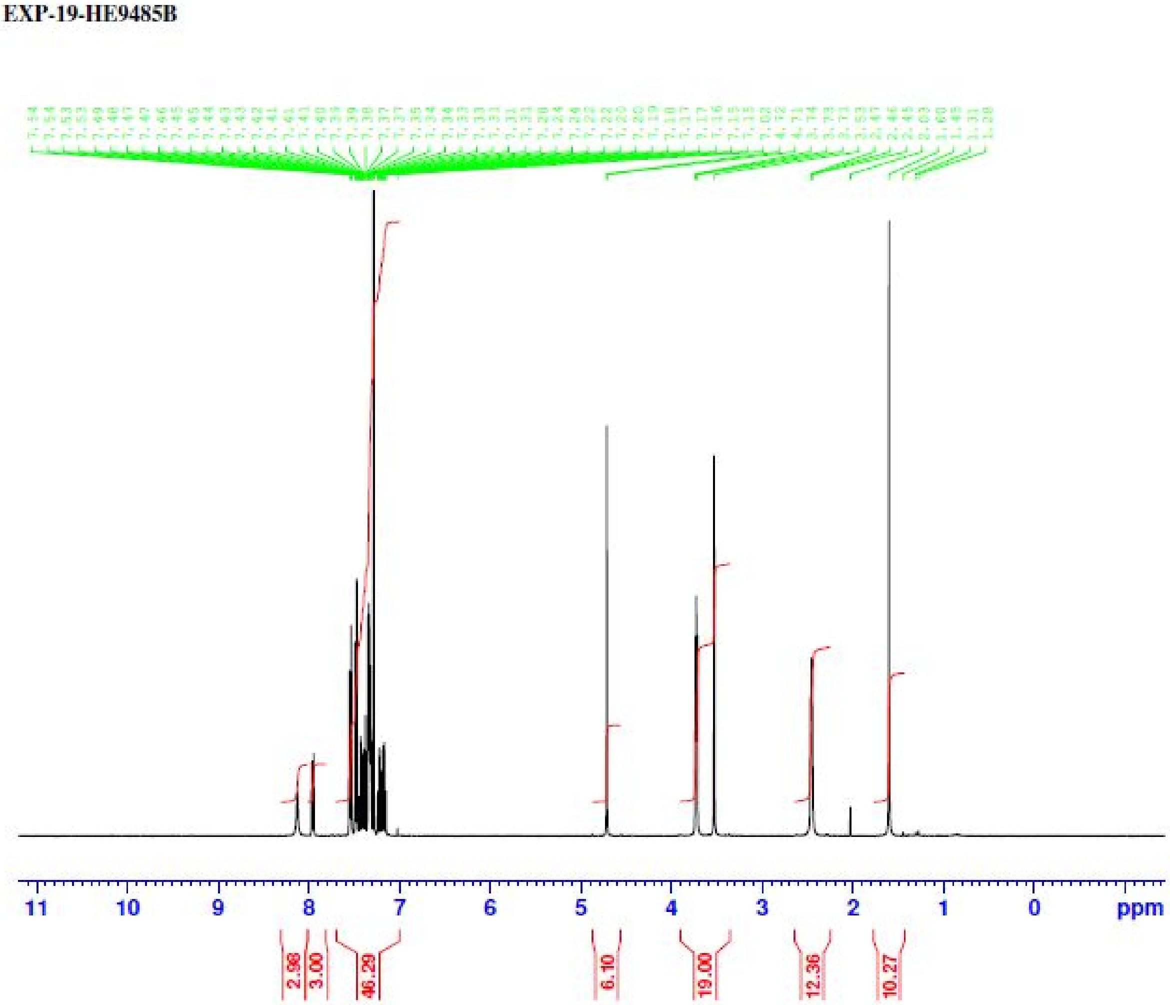

LCMS spectra for compound 42

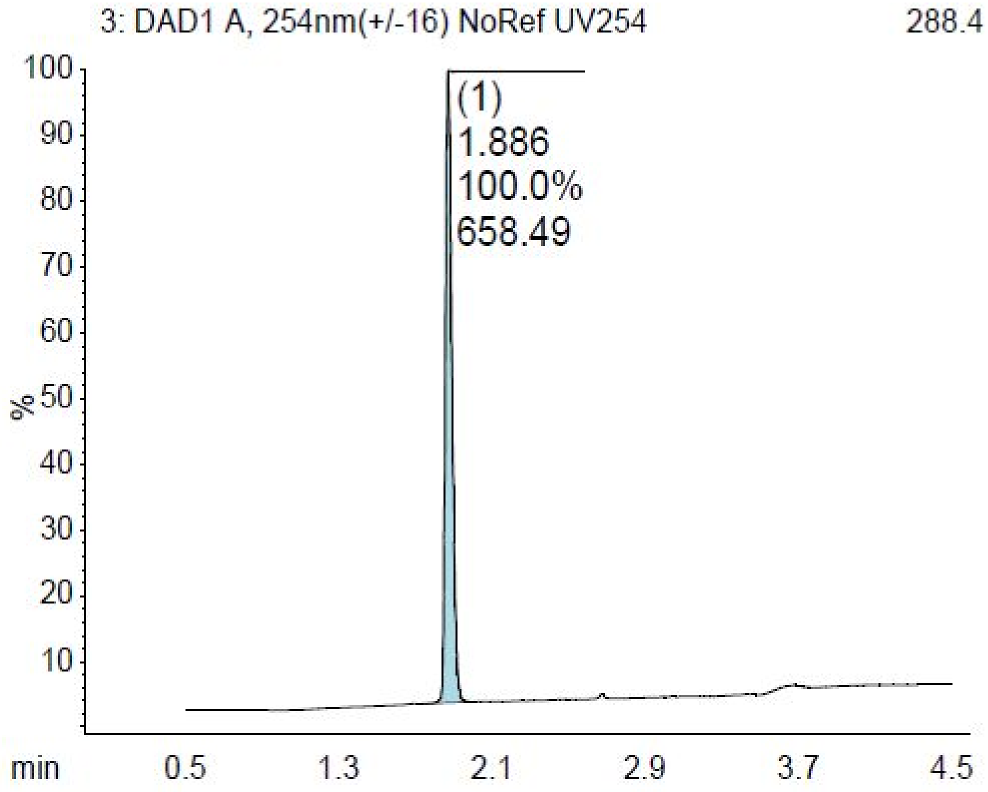

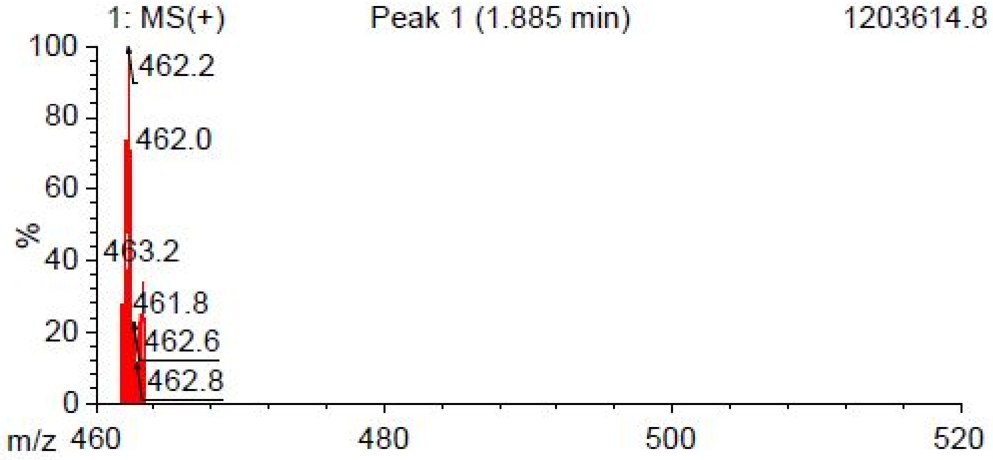

^{1}H NMR spectra for compound 43

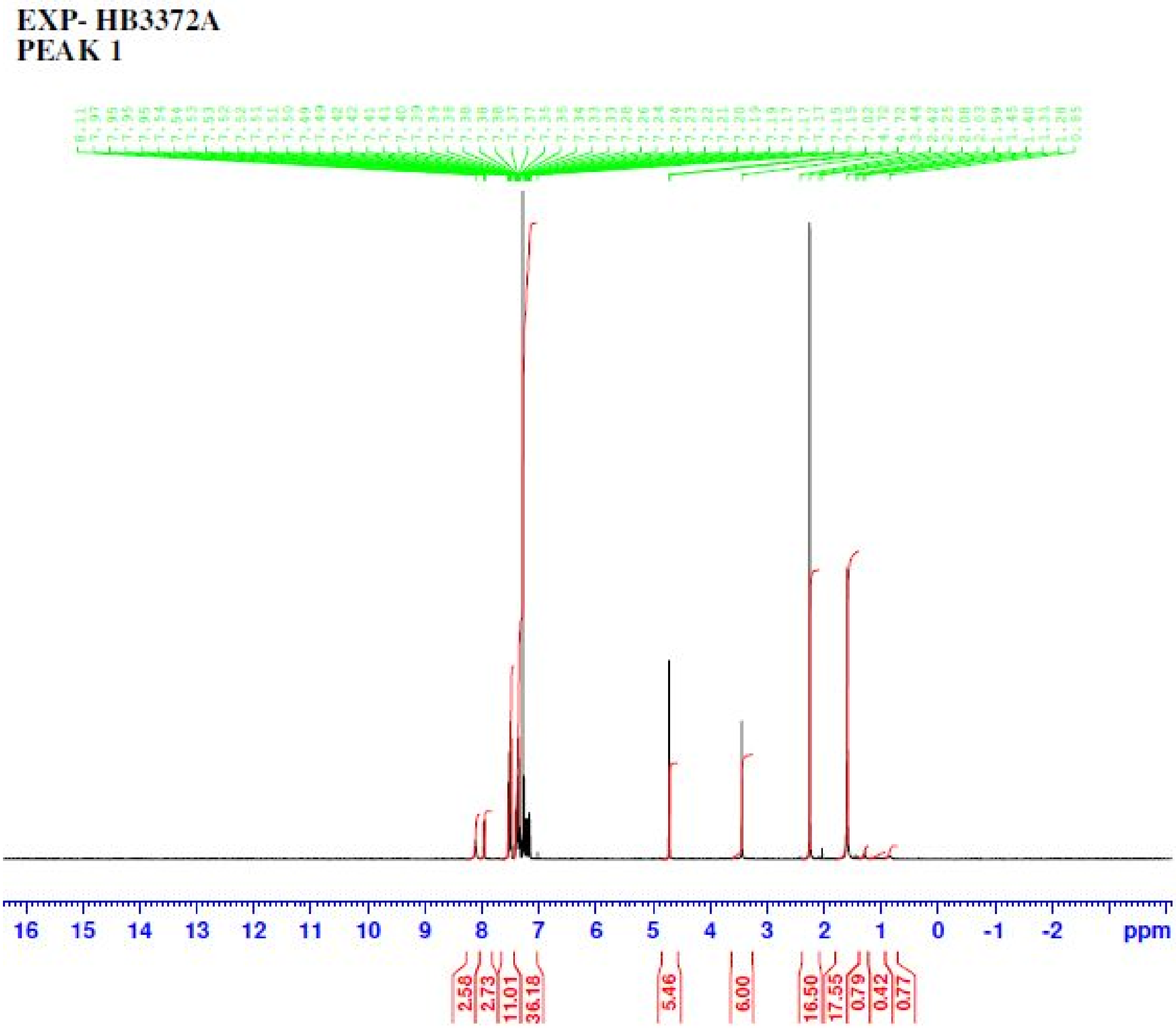

LCMS spectra for compound 43

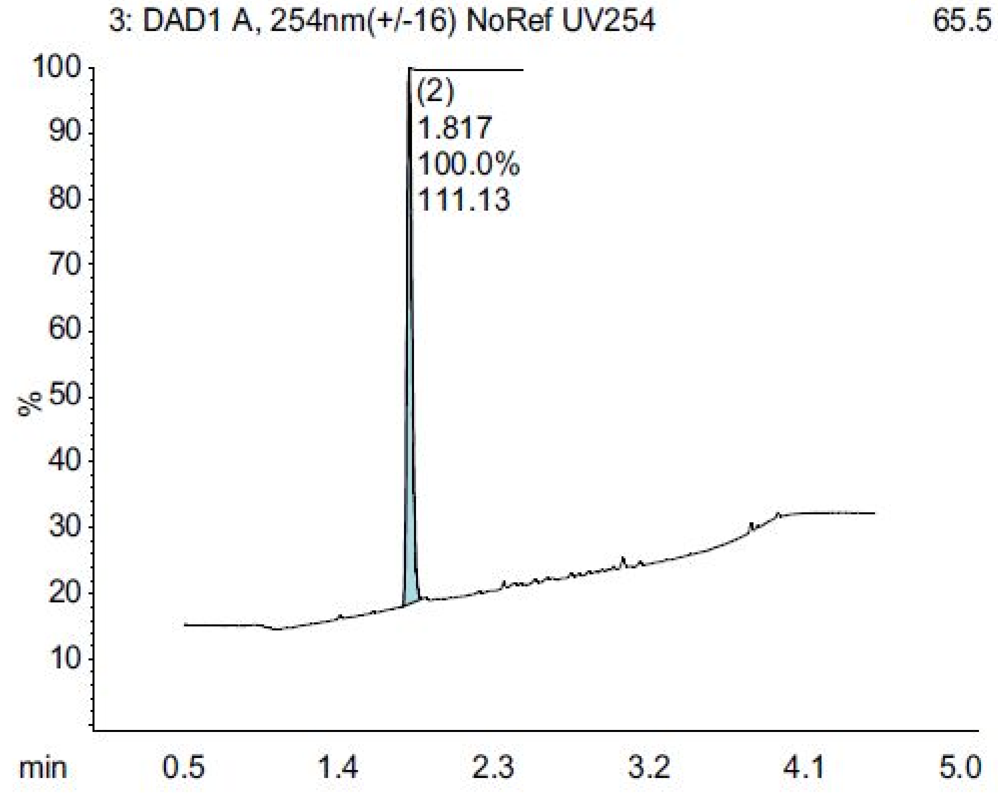

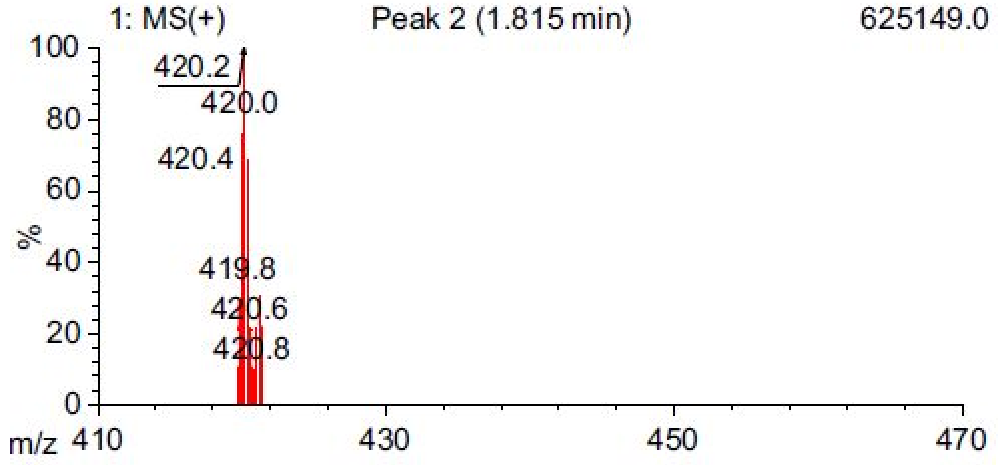

^{1}H spectra for compound 44

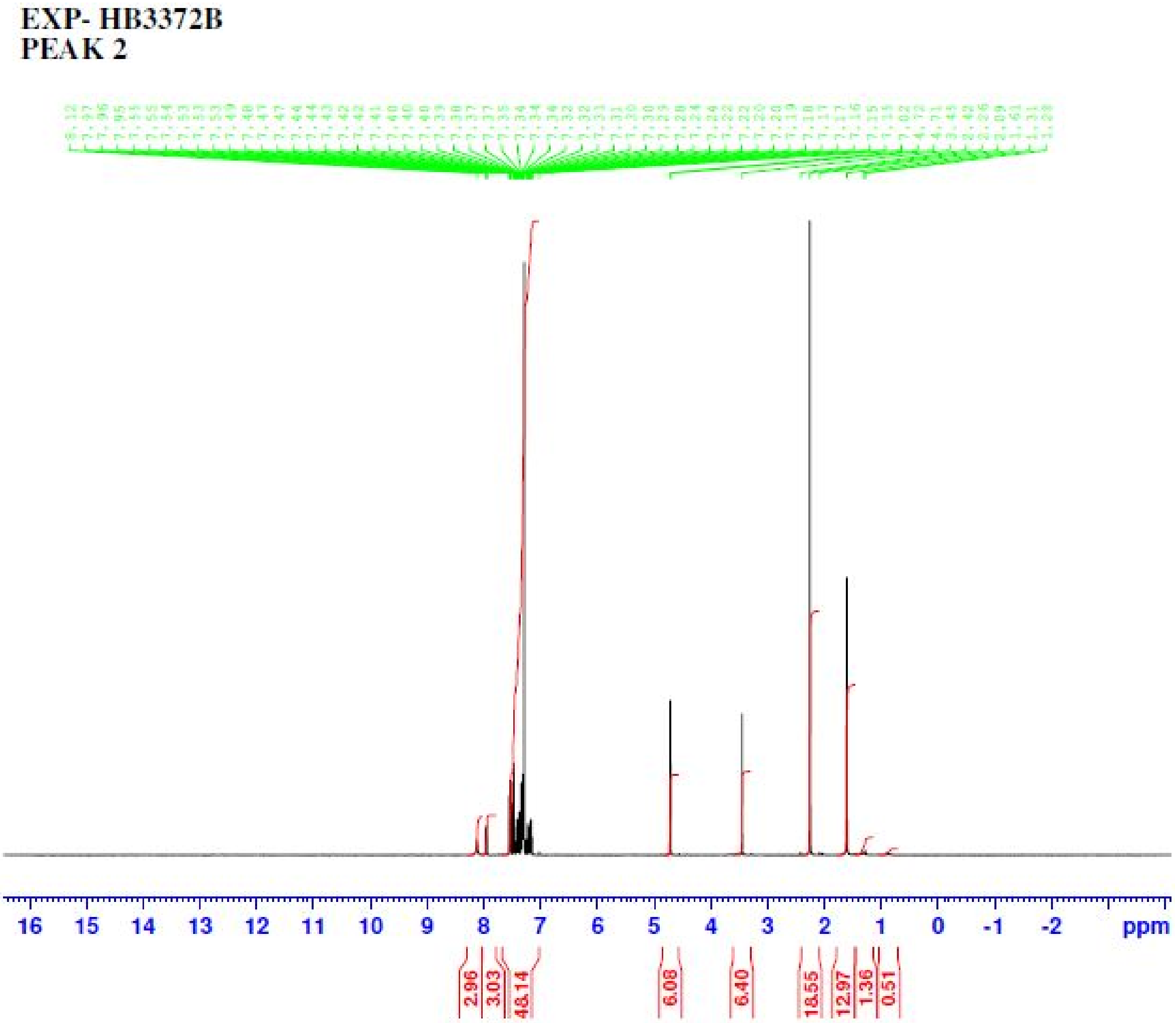

LCMS spectra for compound 44

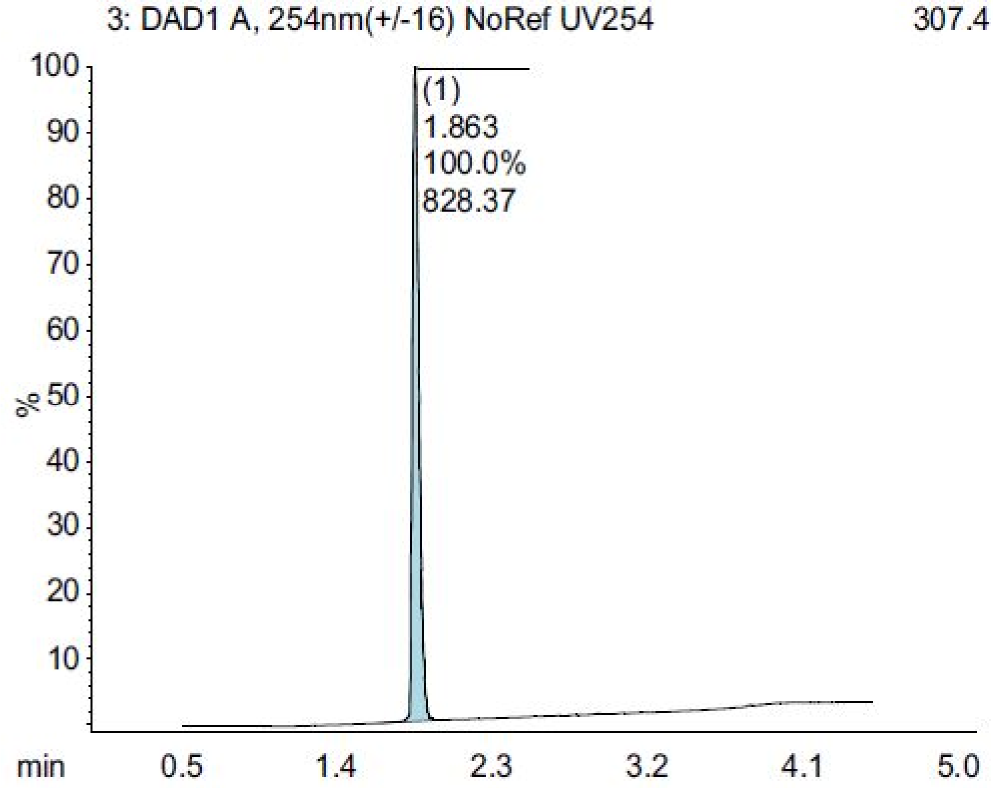

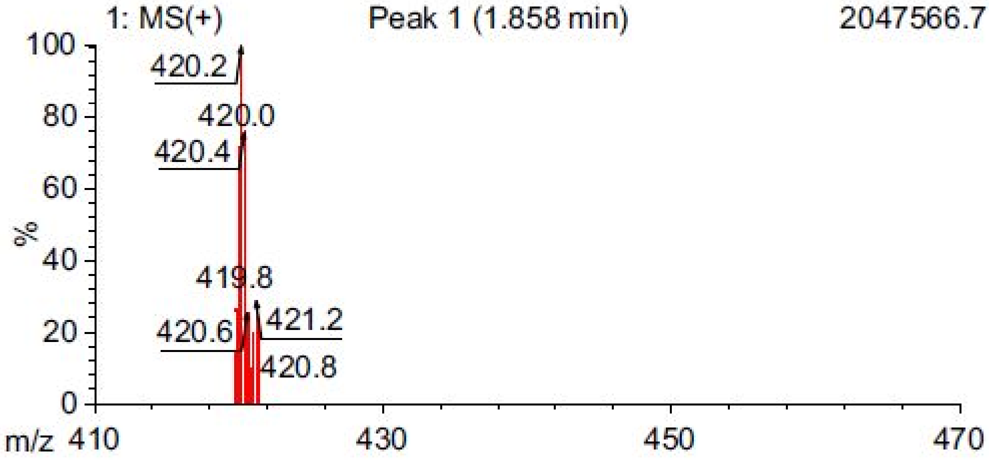

^{1}H spectra for compound 45

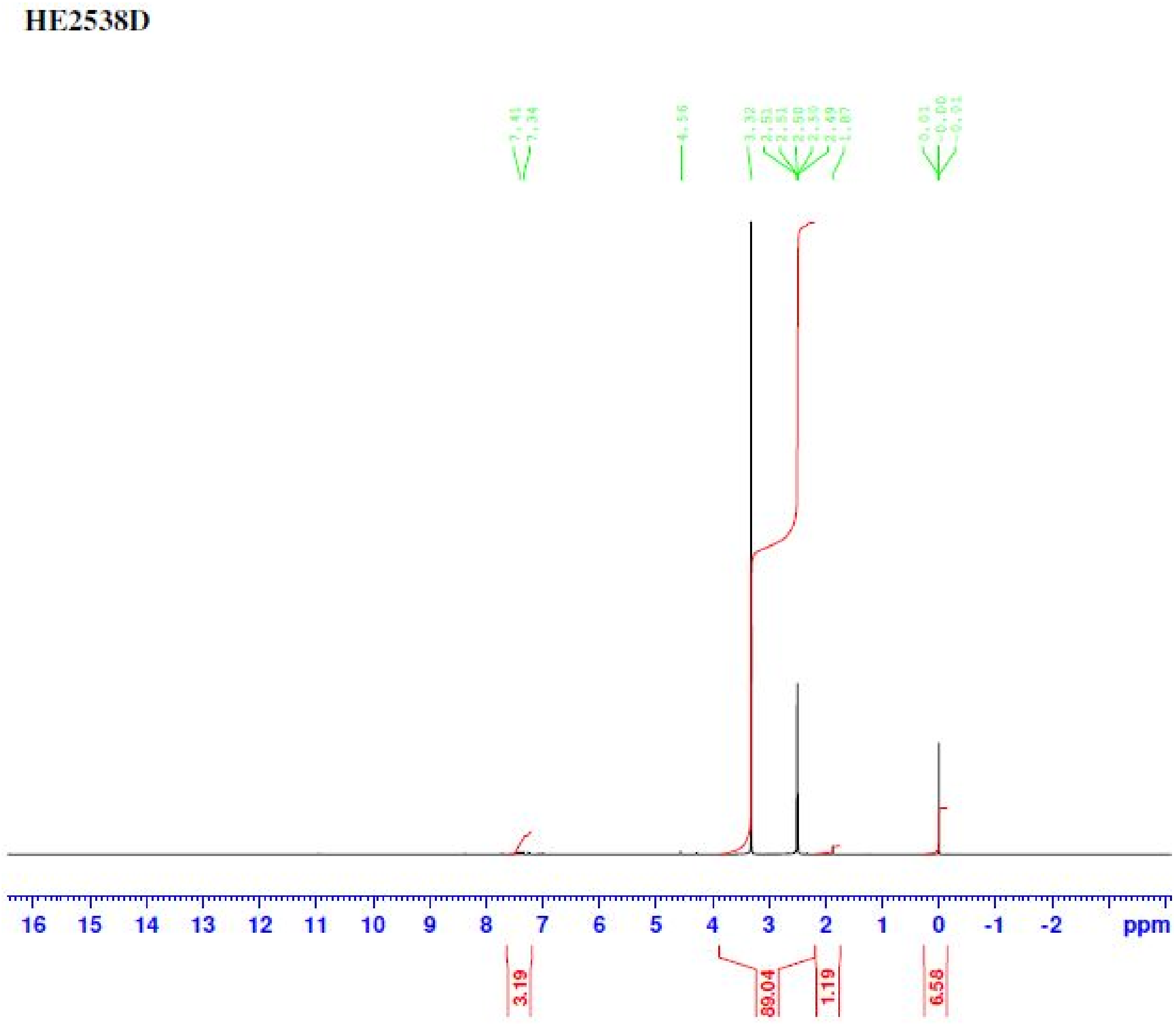

LCMS spectra for compound 45

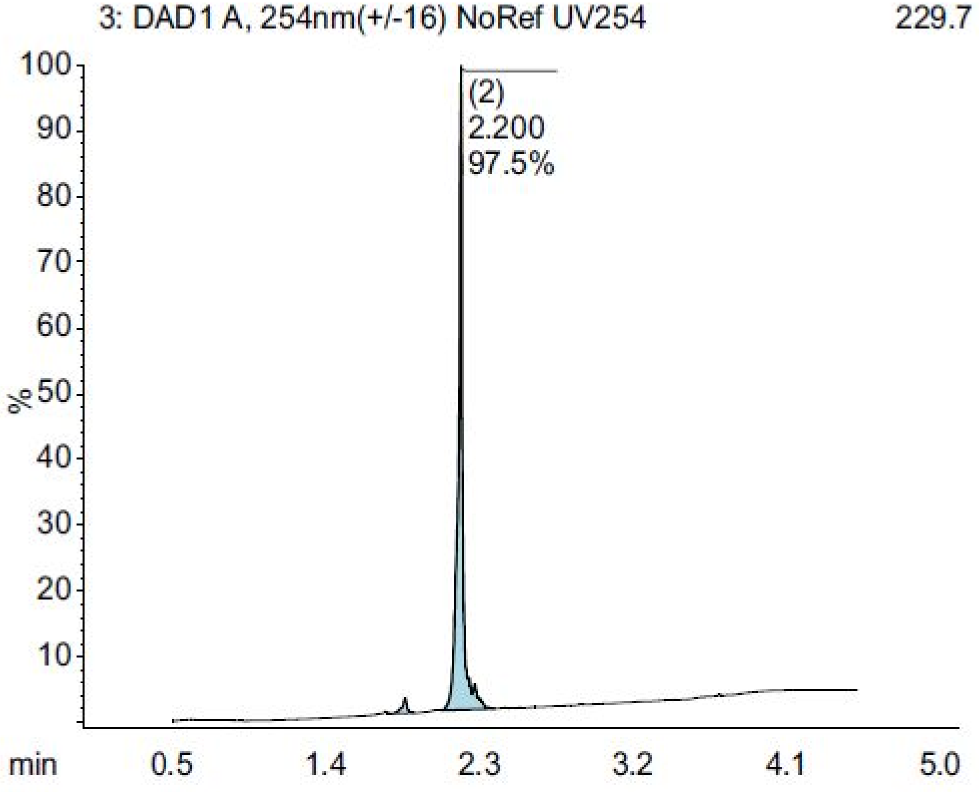

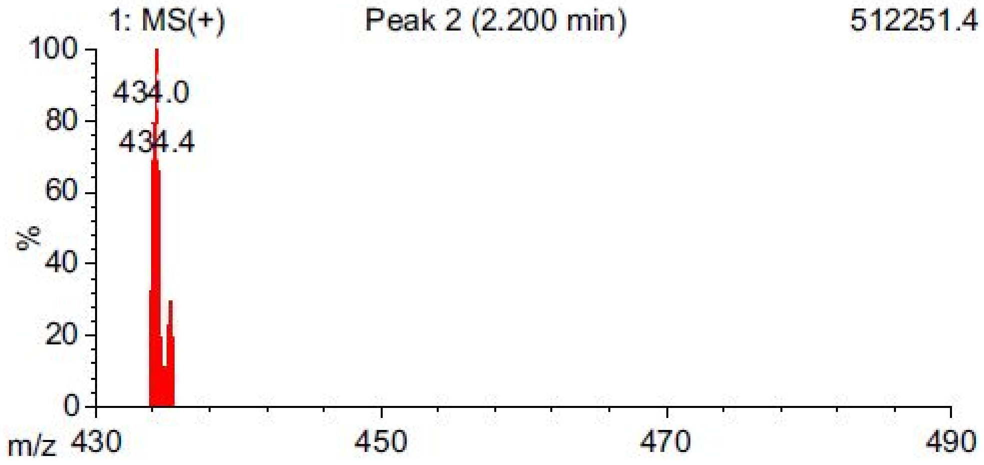

^{1}H spectra for compound 46

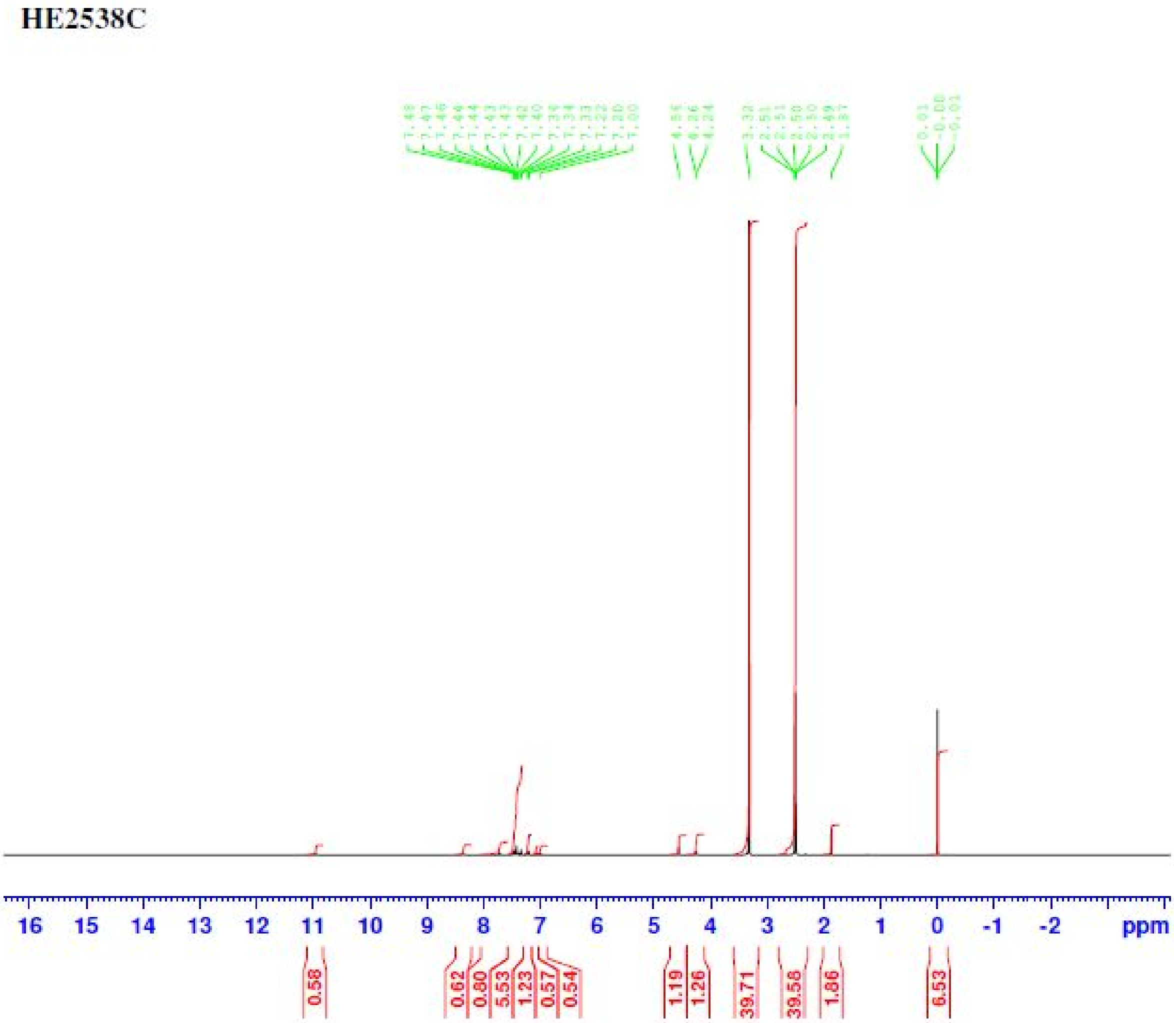

LCMS spectra for compound 46

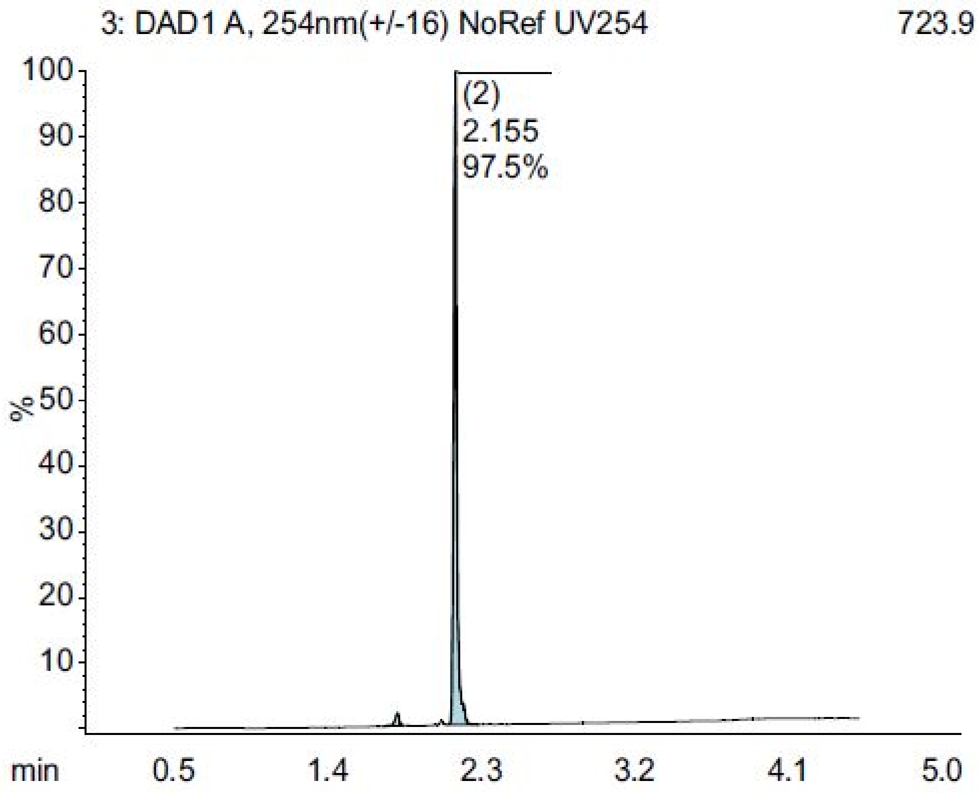

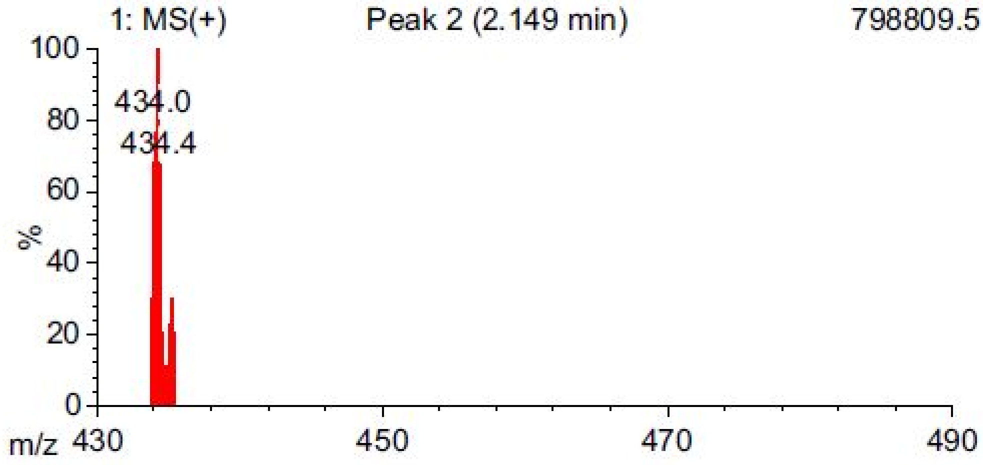

^{1}H spectra for compound 47

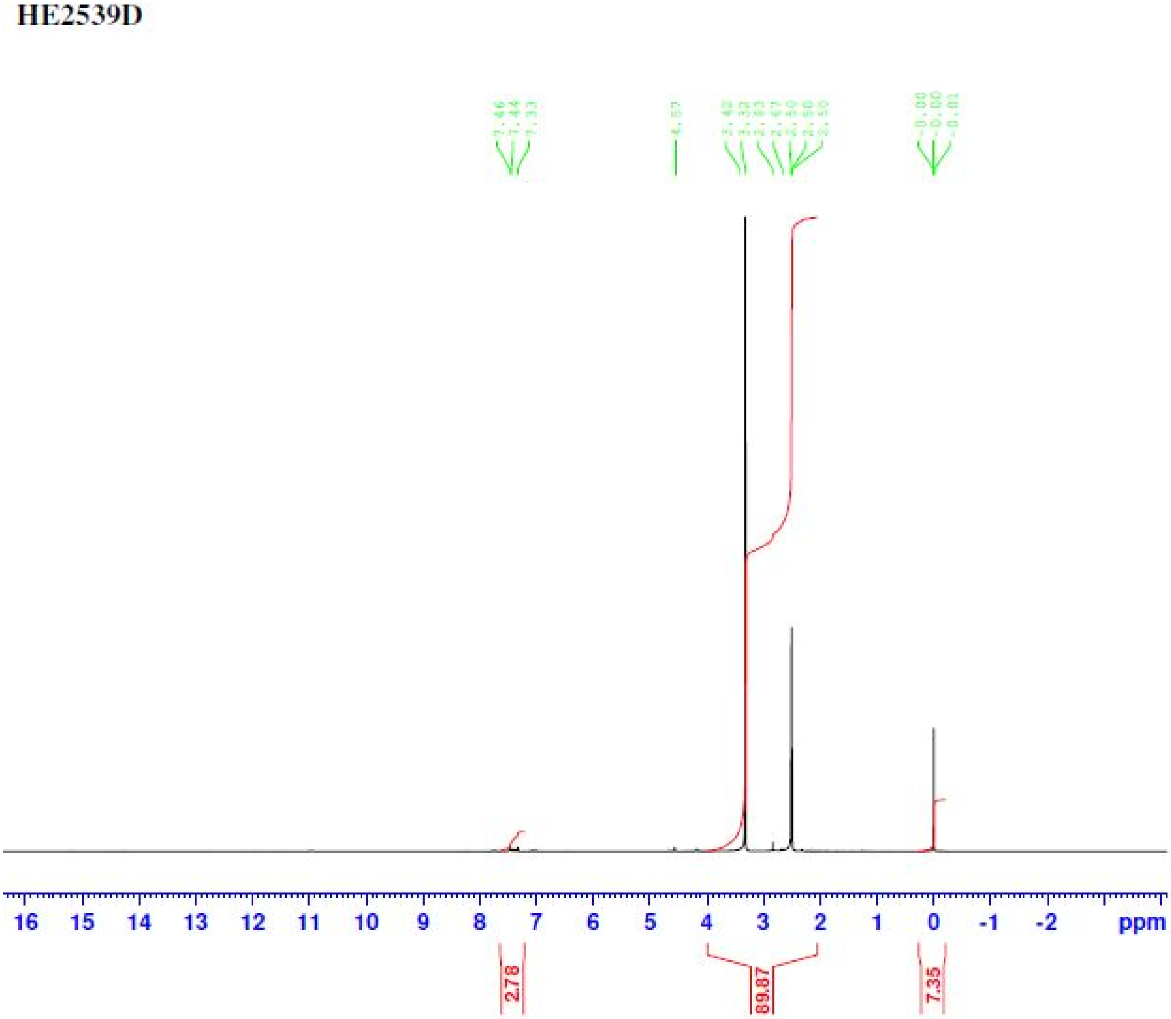

LCMS spectra for compound 47

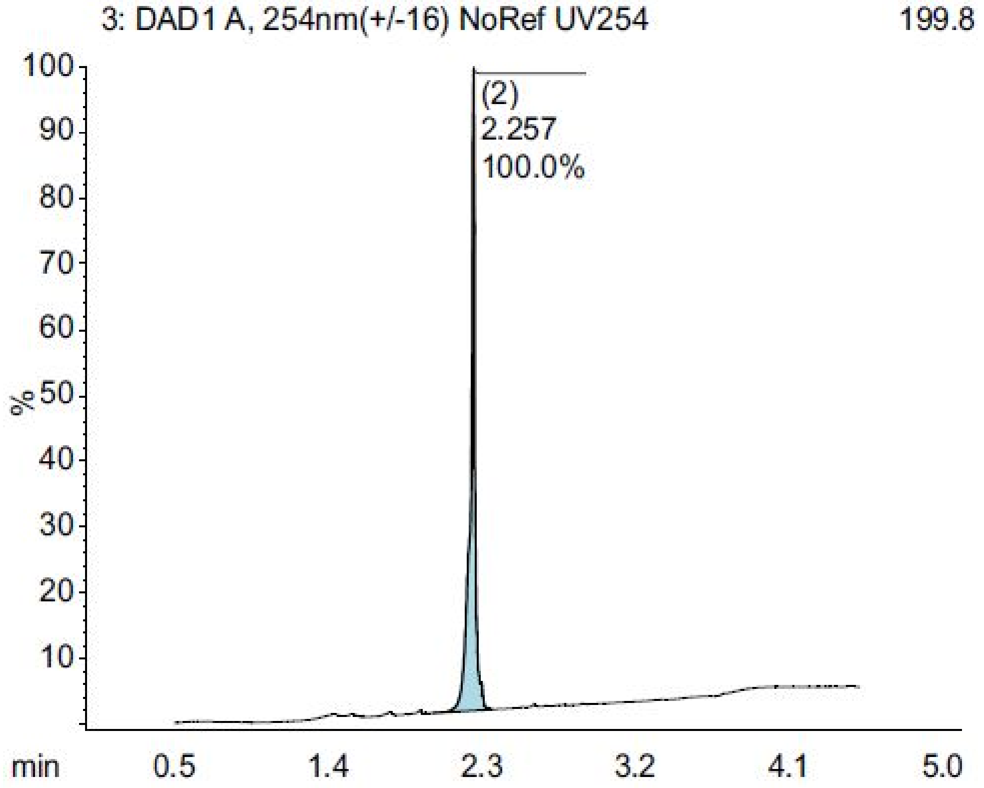

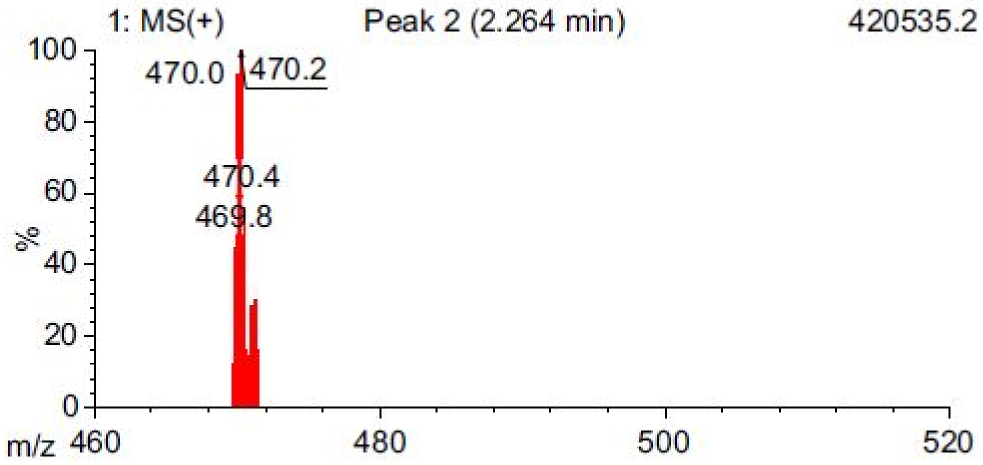

^{1}H spectra for compound 48

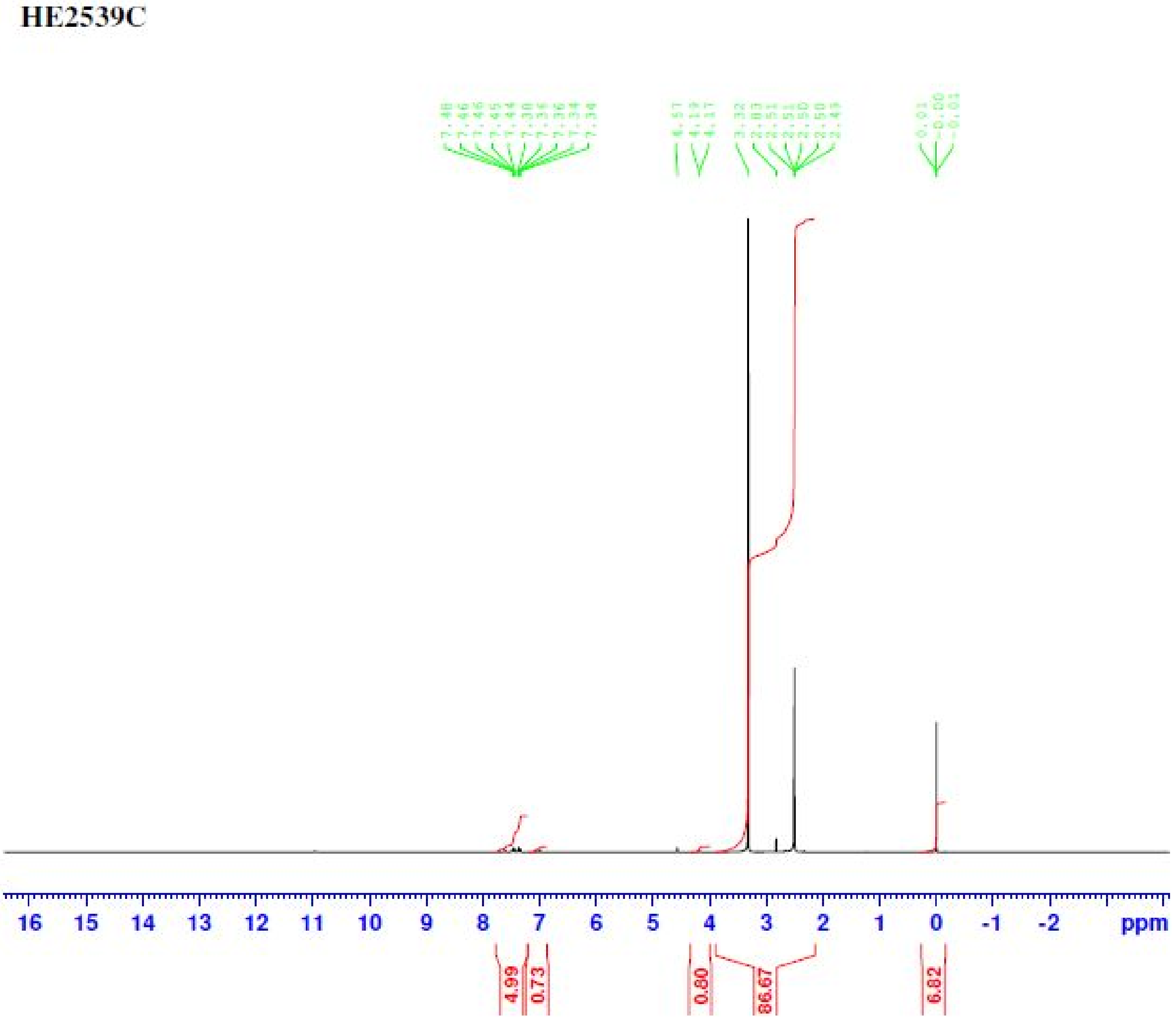

LCMS spectra for compound 48

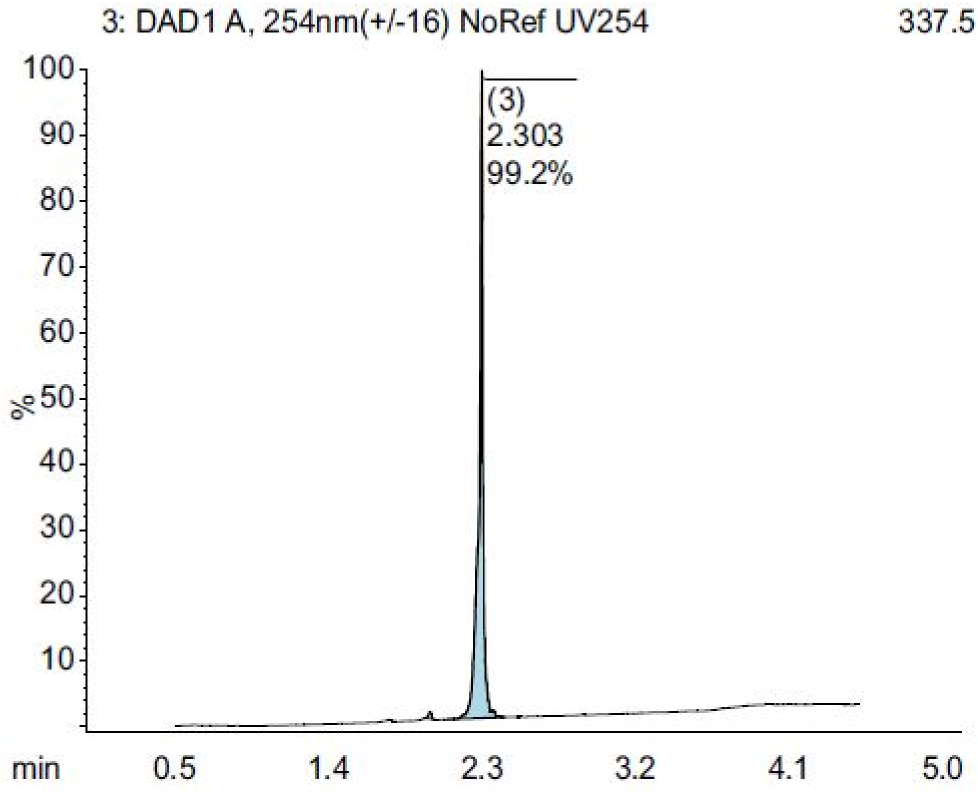

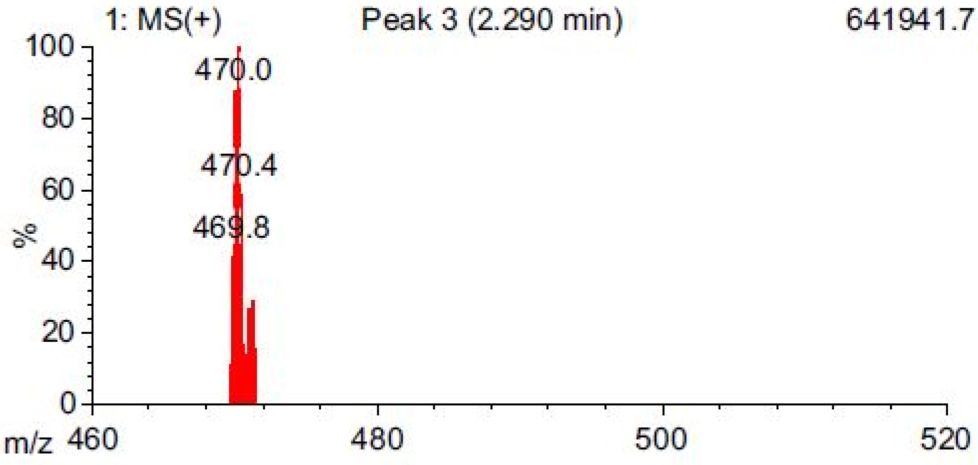

^{1}H spectra for compound 49

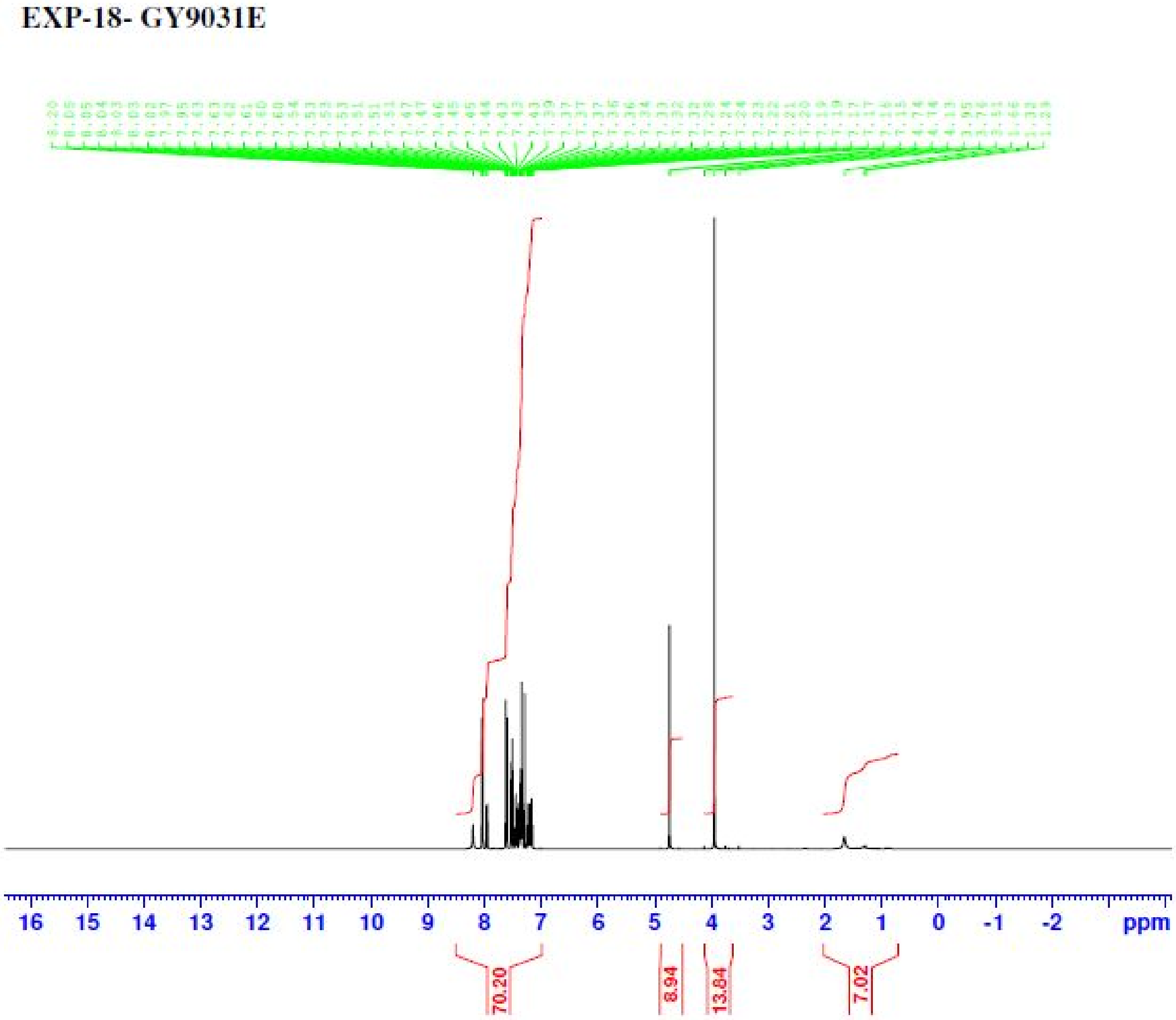

LCMS spectra for compound 49

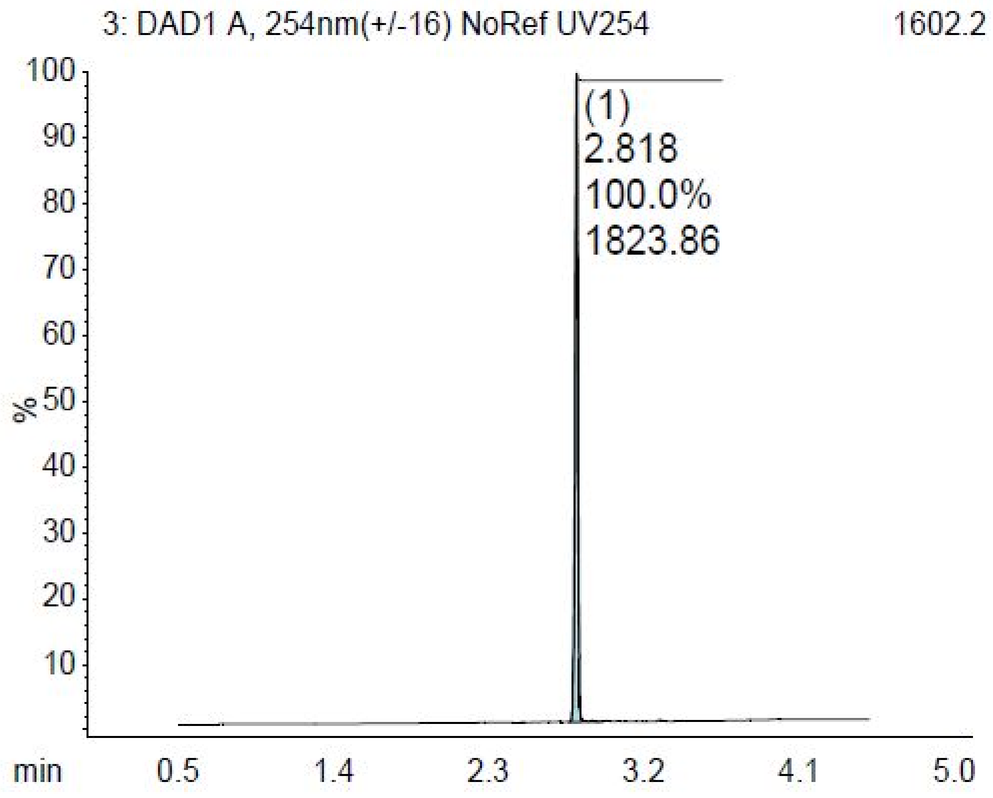

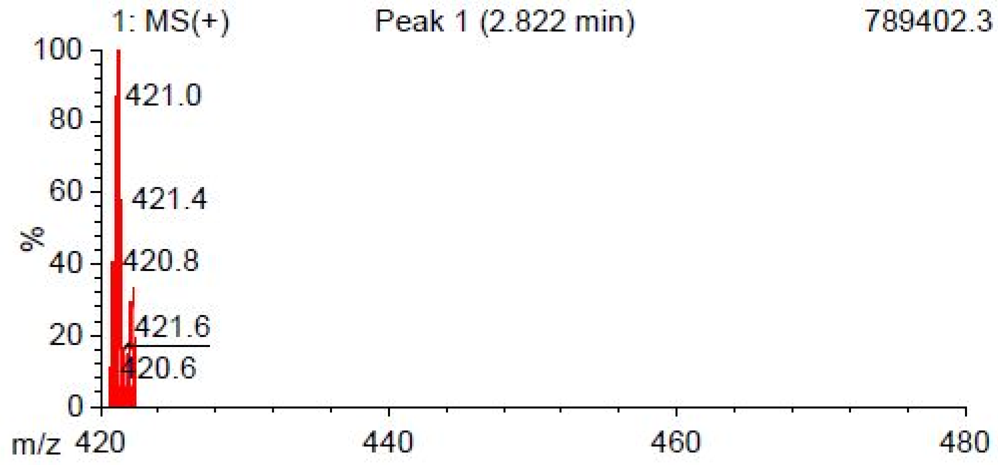

^{1}H spectra for compound 50

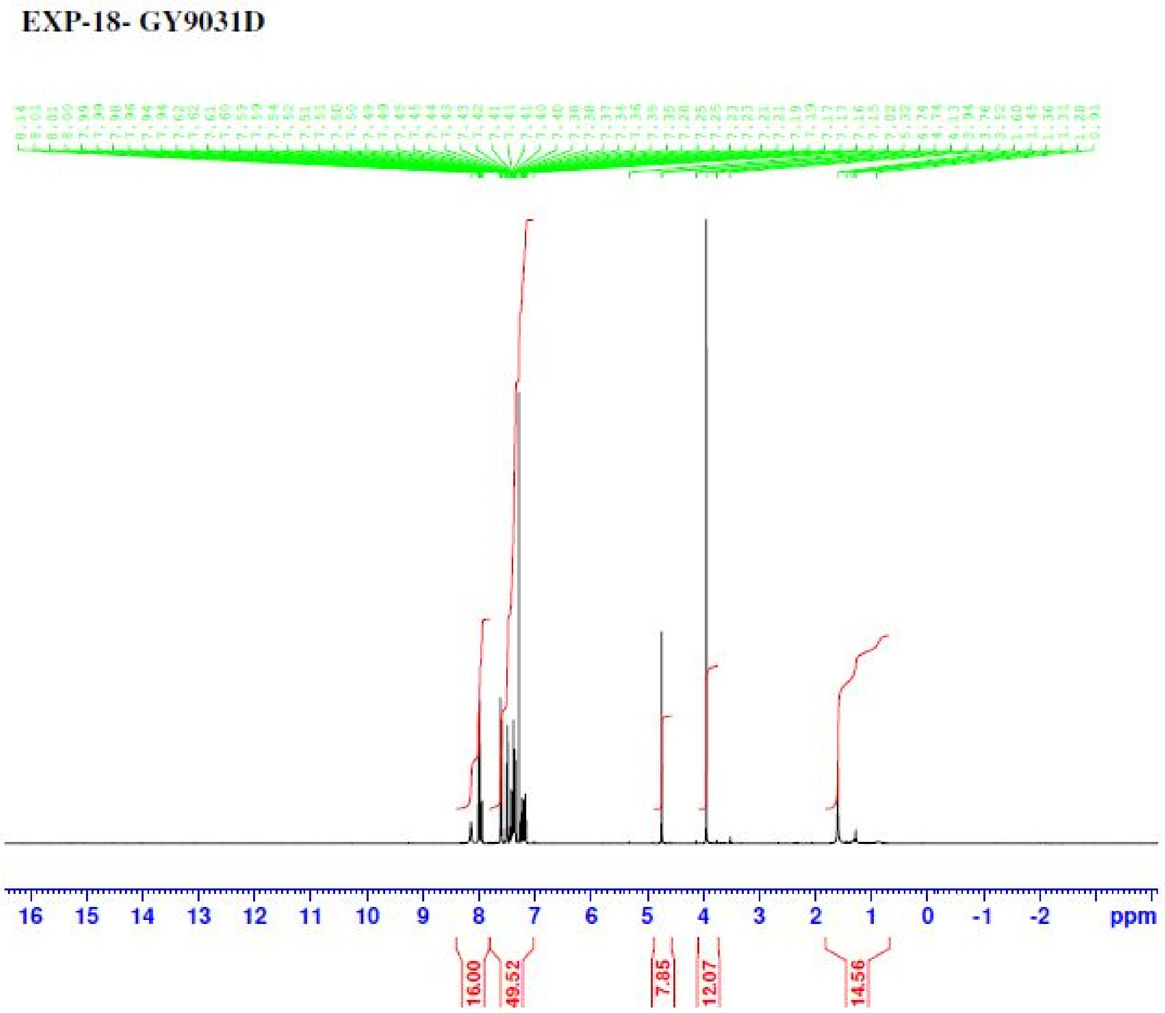

LCMS spectra for compound 50

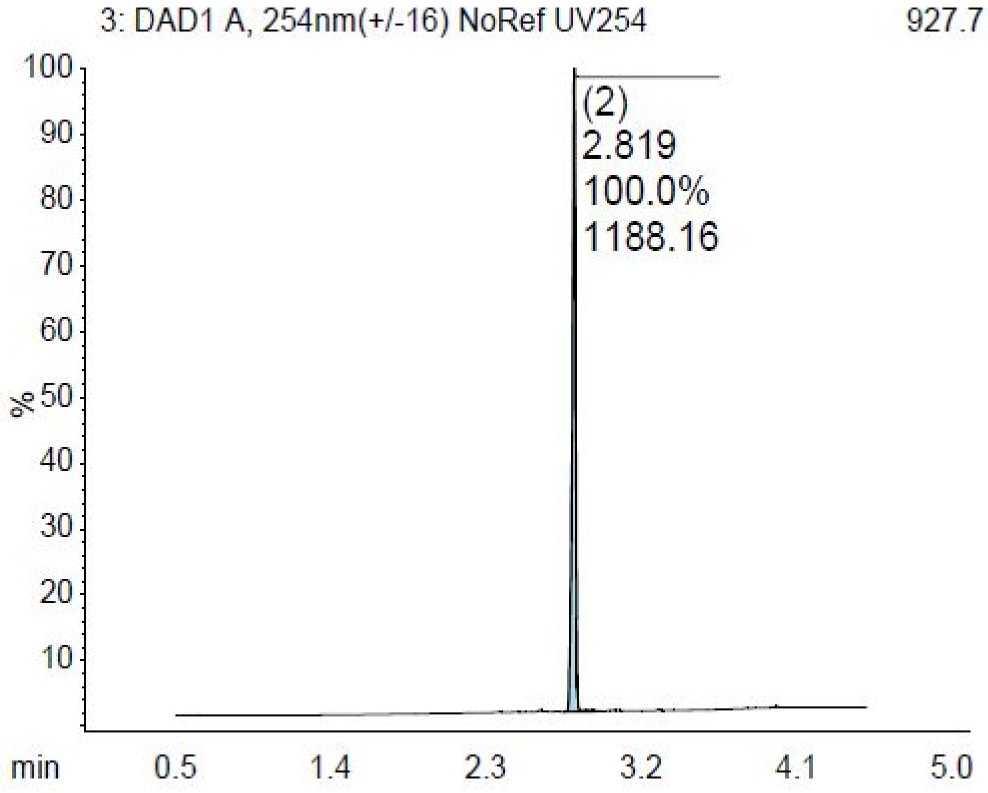

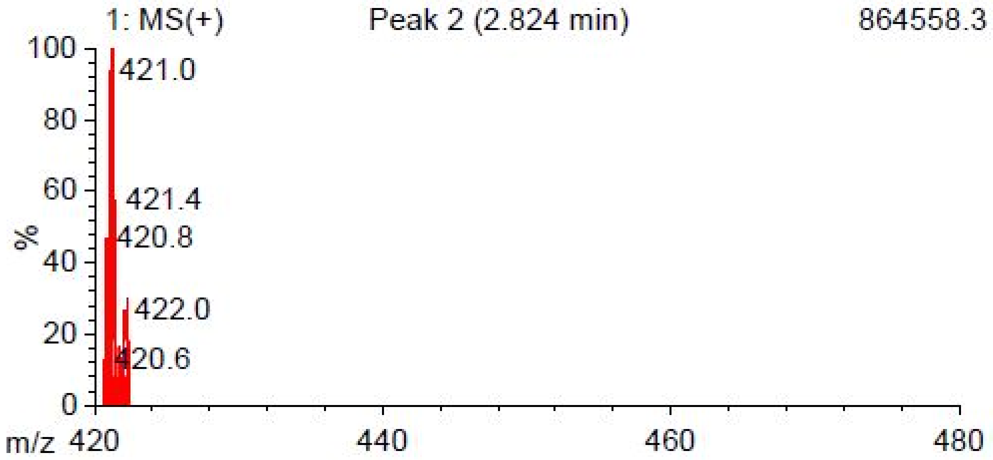

^{1}H spectra for compound 51

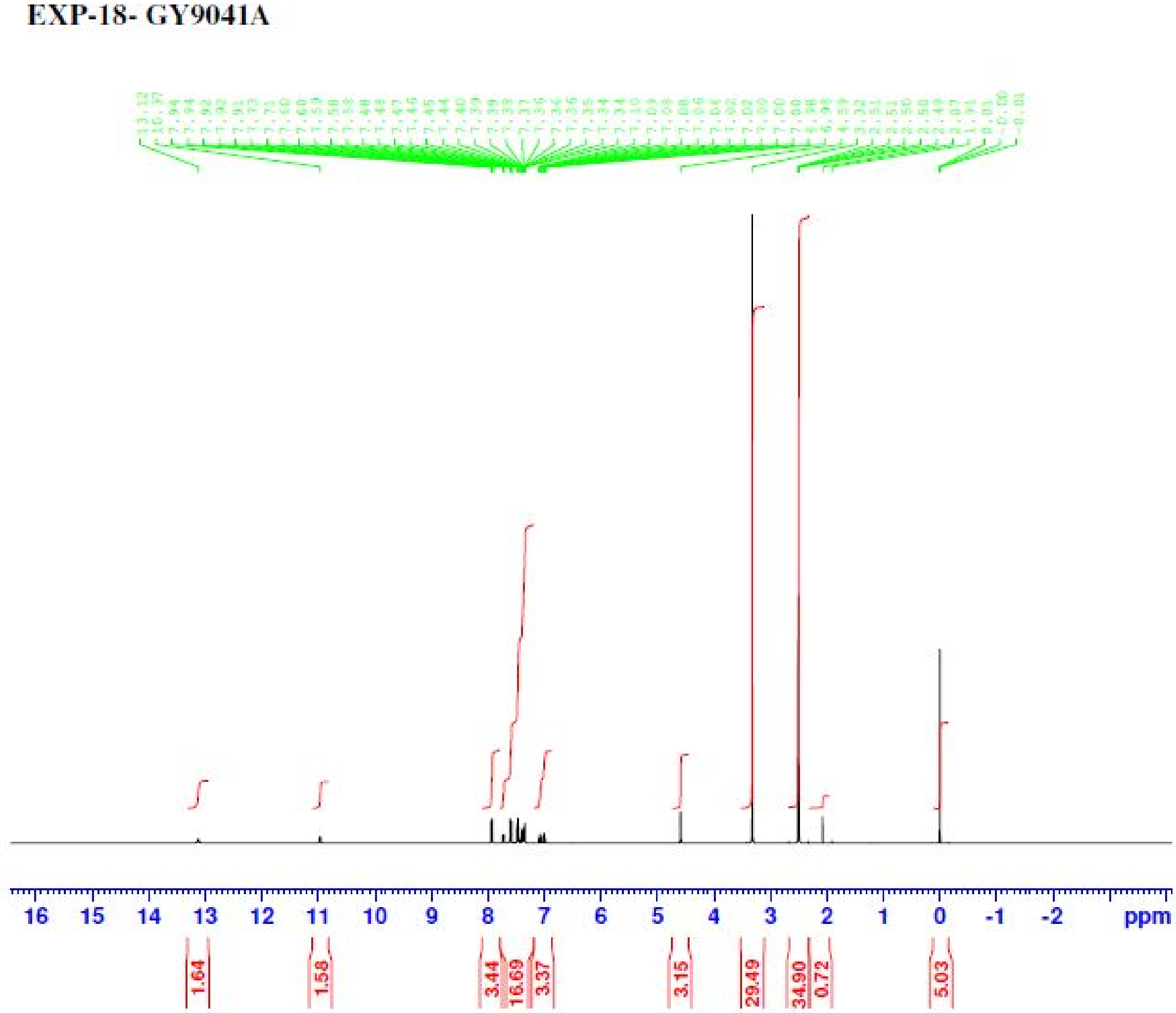

LCMS spectra for compound 51

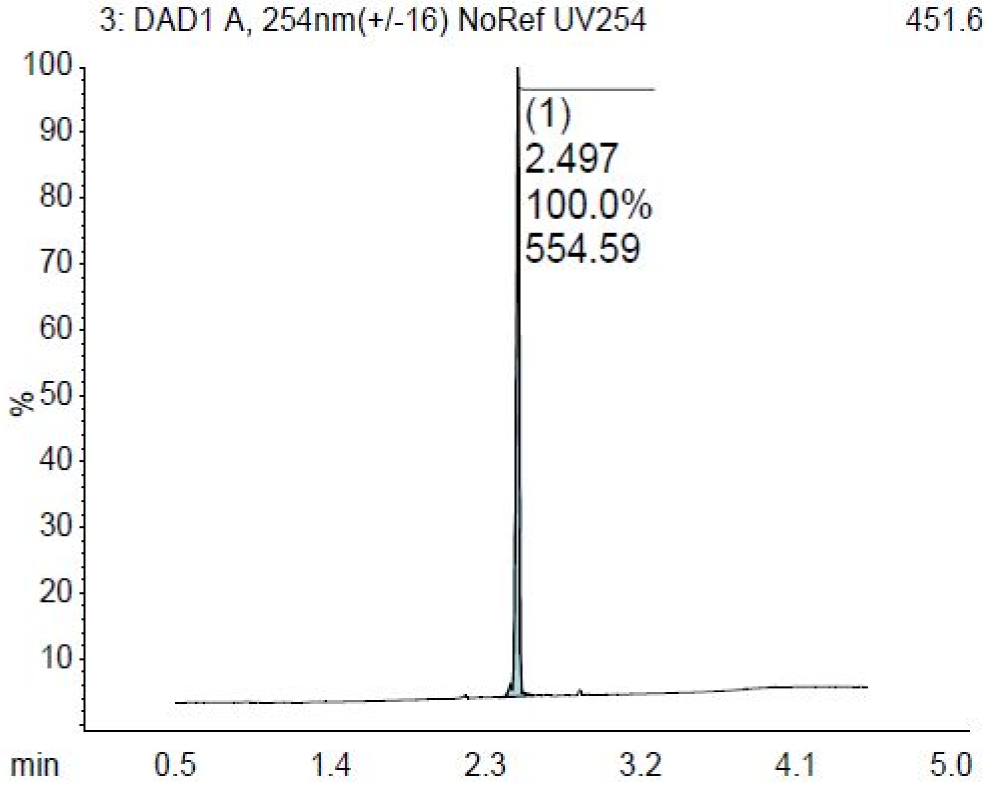

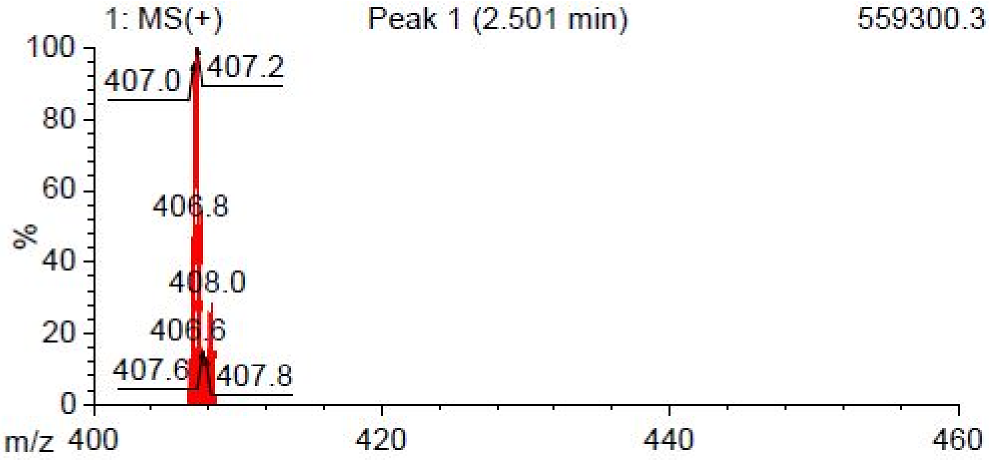

^{1}H spectra for compound 52

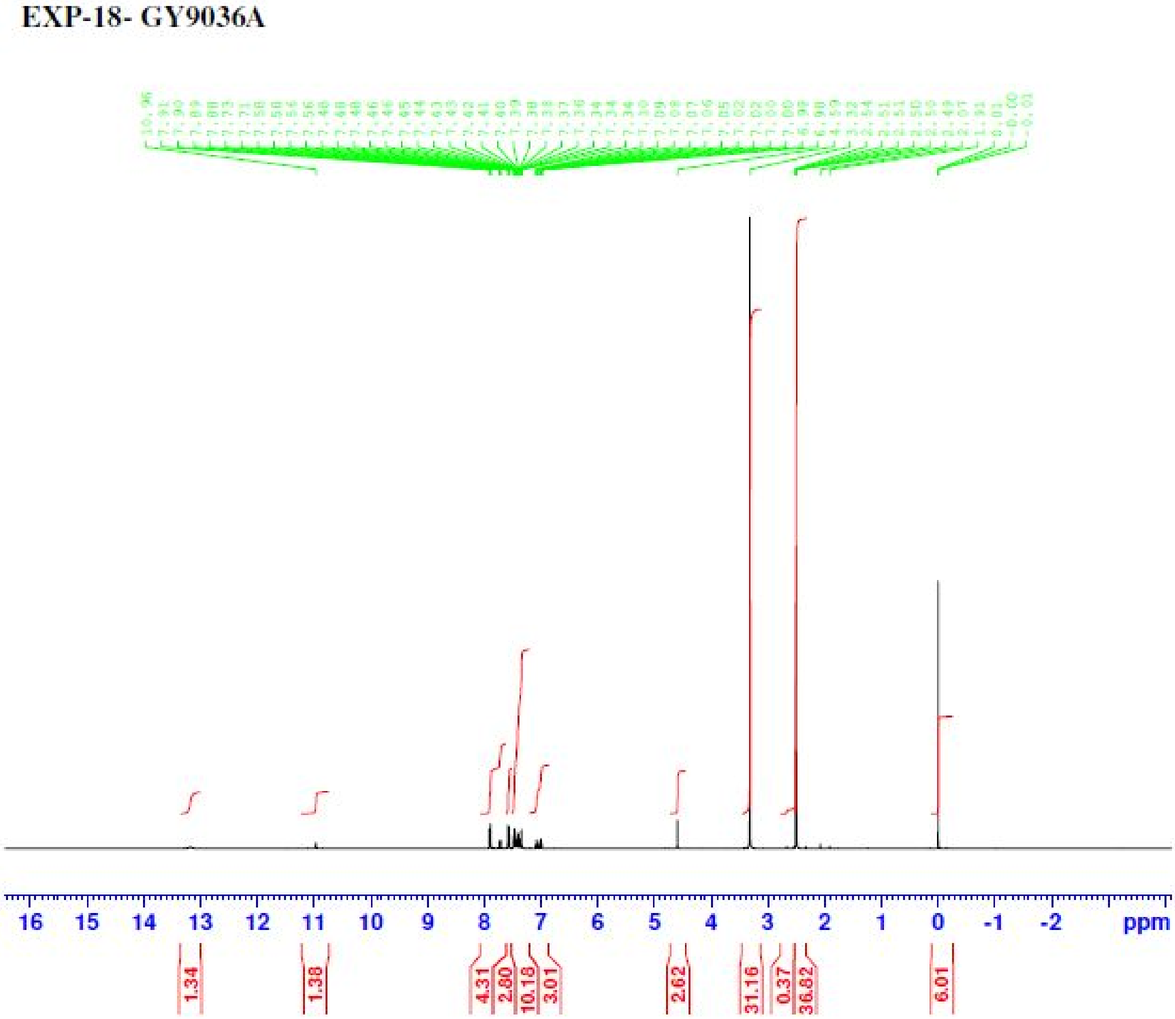

LCMS spectra for compound 52

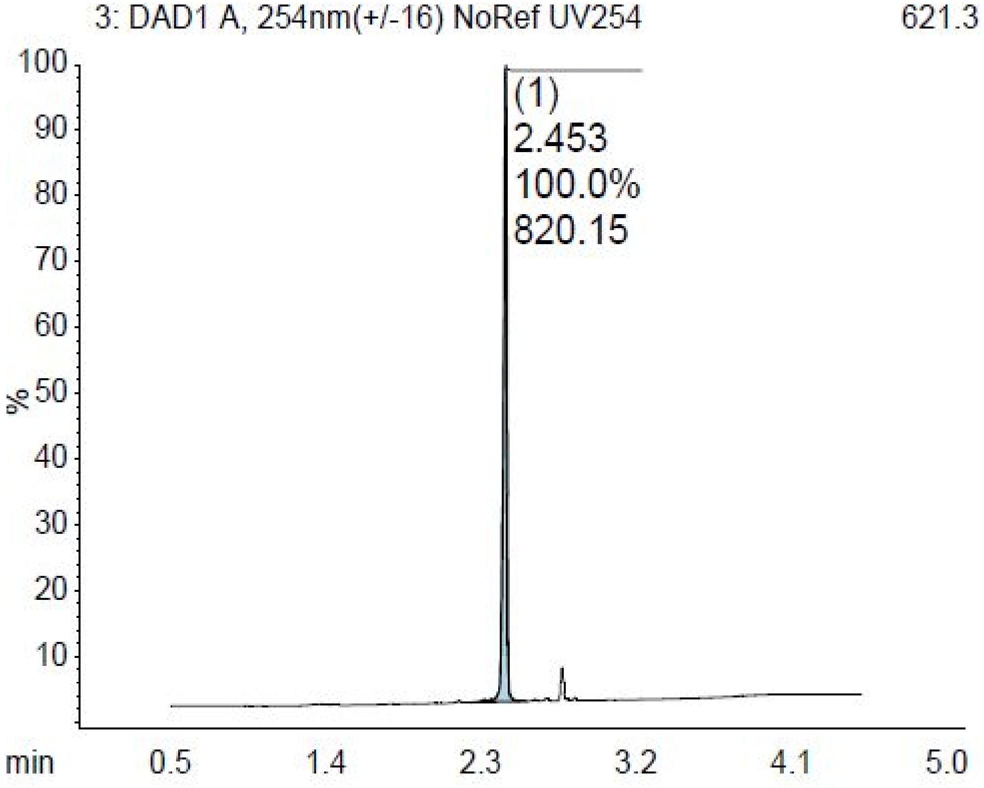

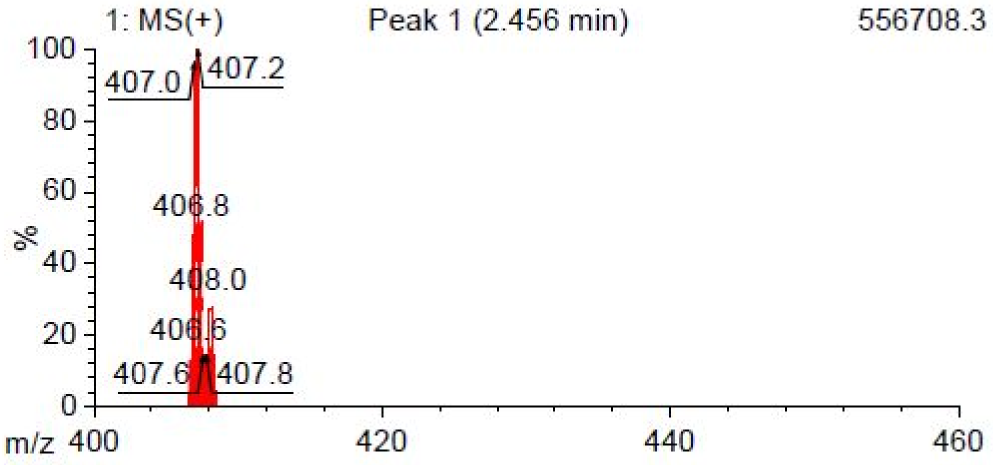

^1H spectra for compound 53

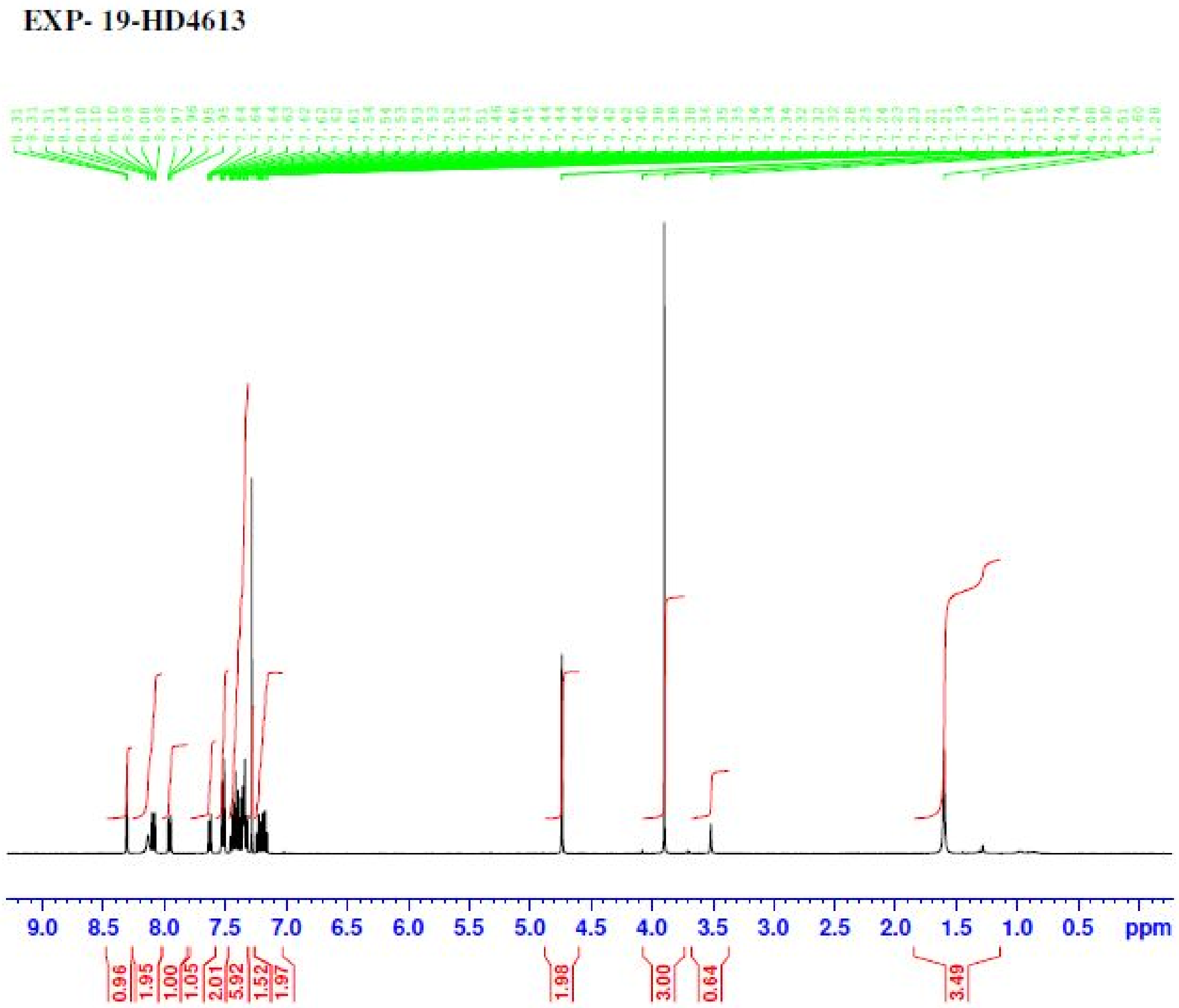

LCMS spectra for compound 53

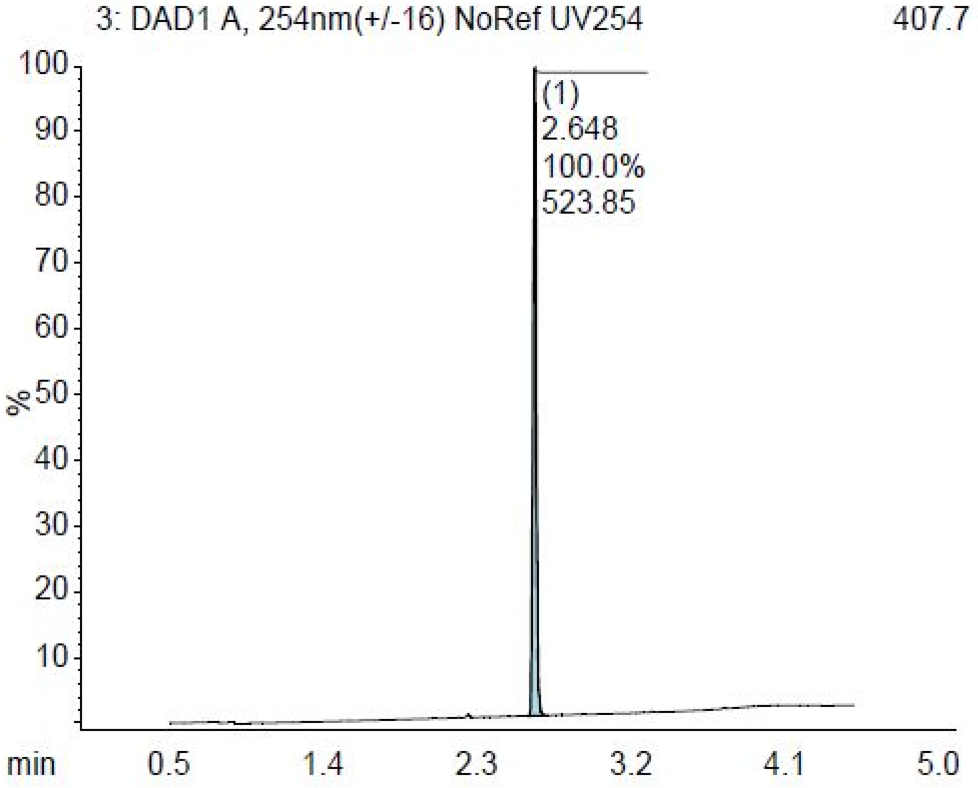

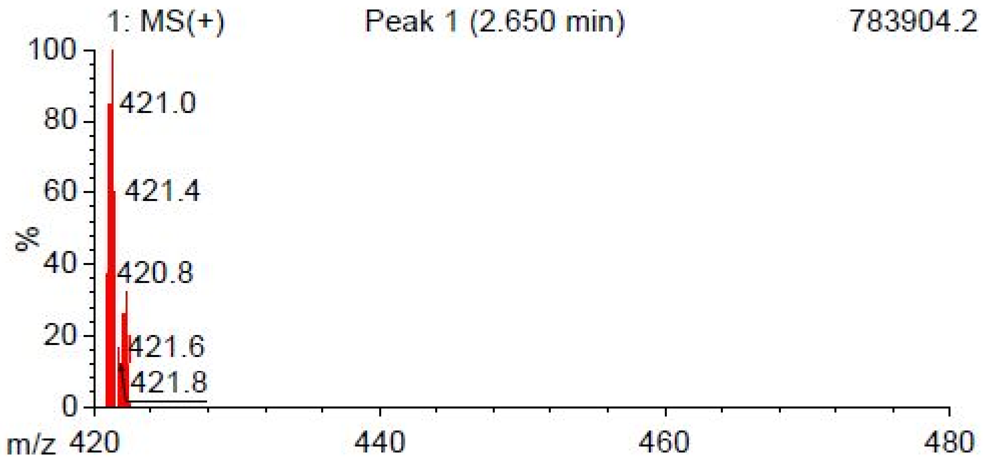

^{1}H spectra for compound 54

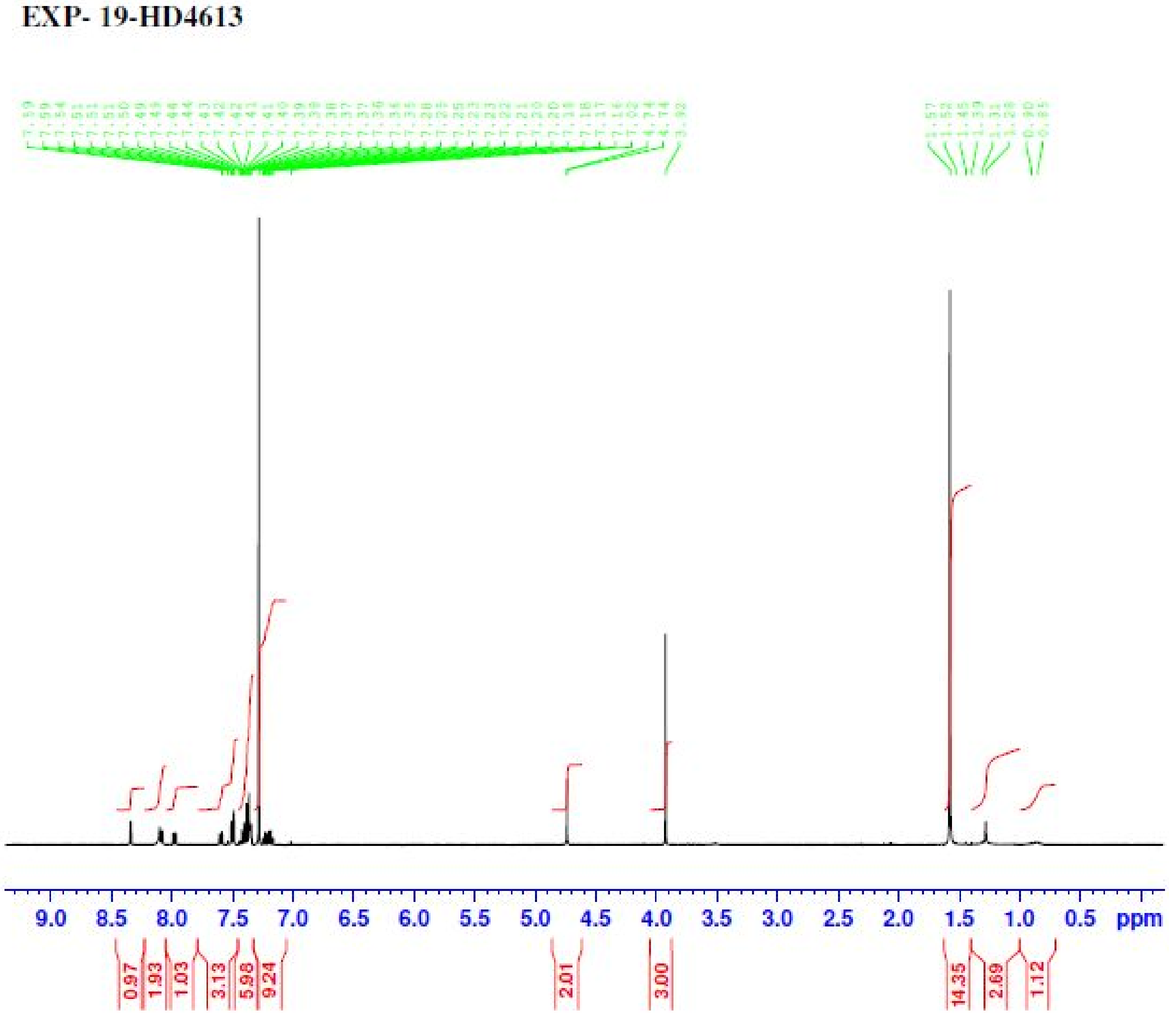

LCMS spectra for compound 54

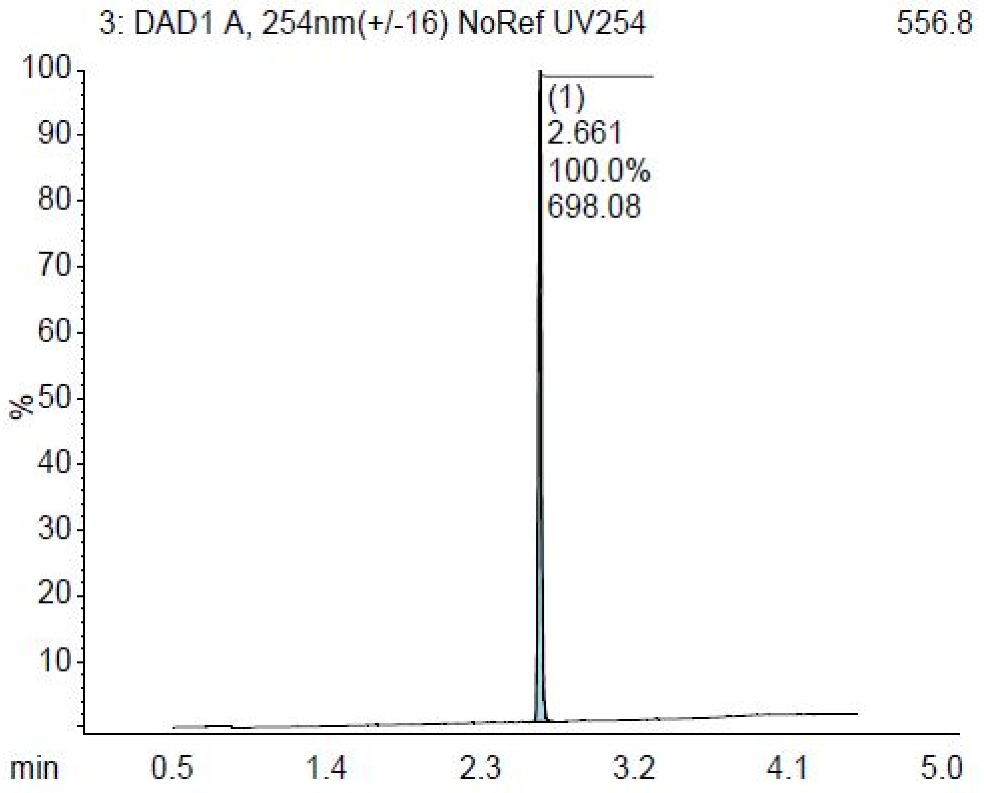

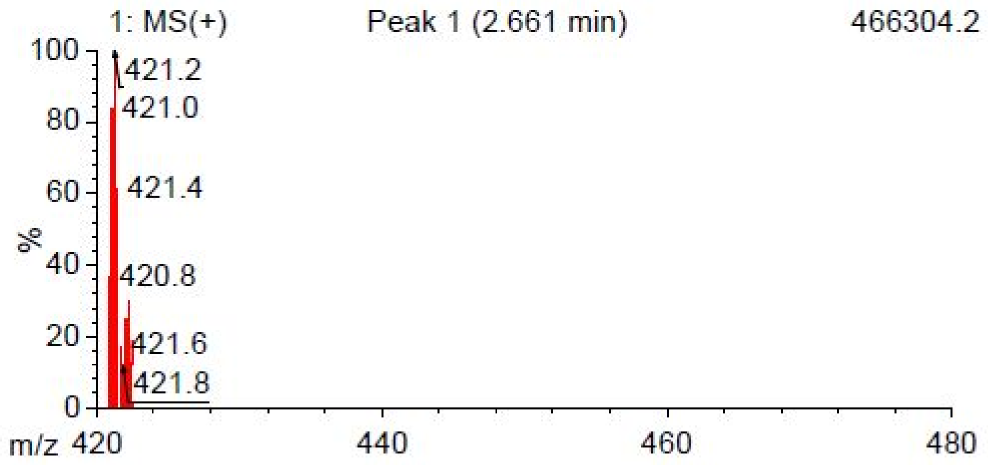

^{1}H spectra for compound 55

EXP-19-HD4622C

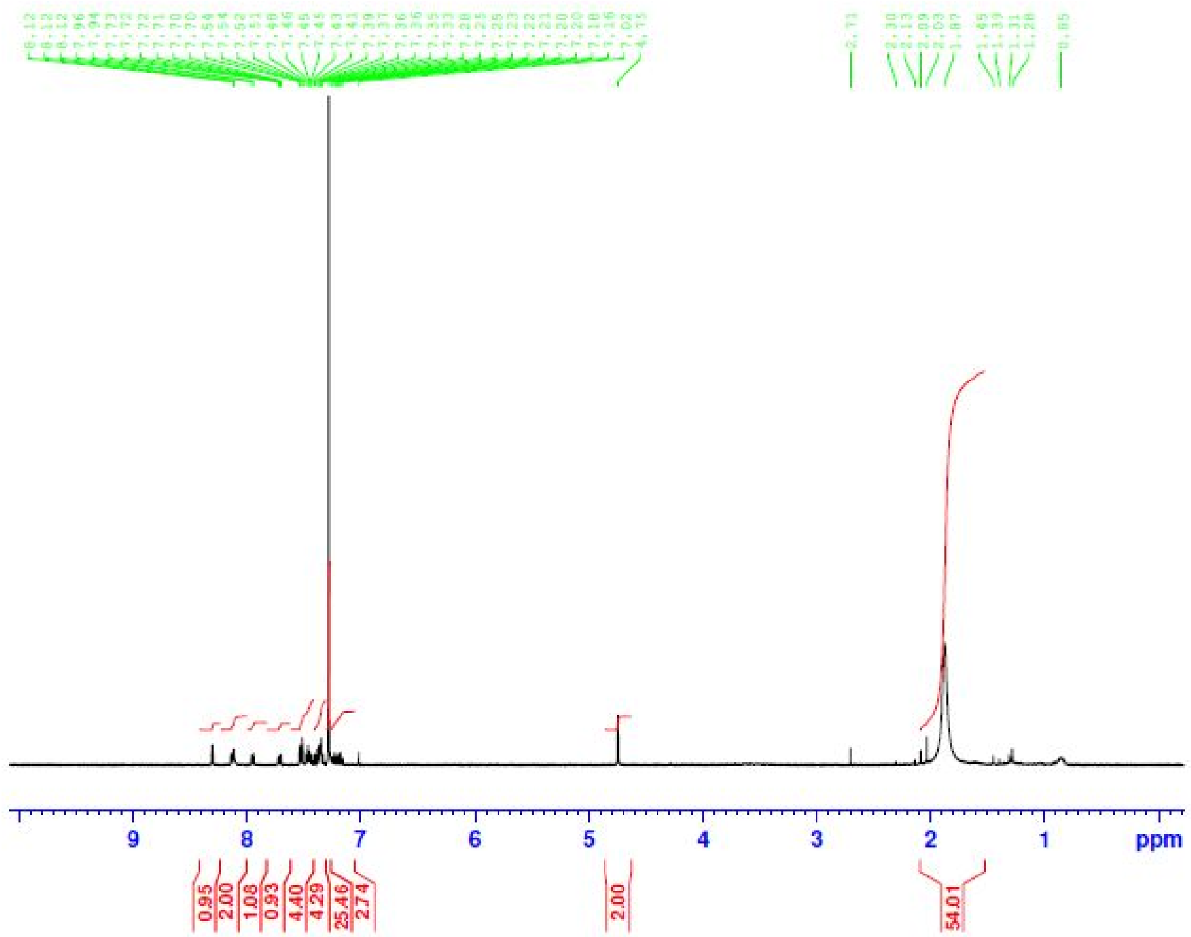

LCMS spectra for compound 55

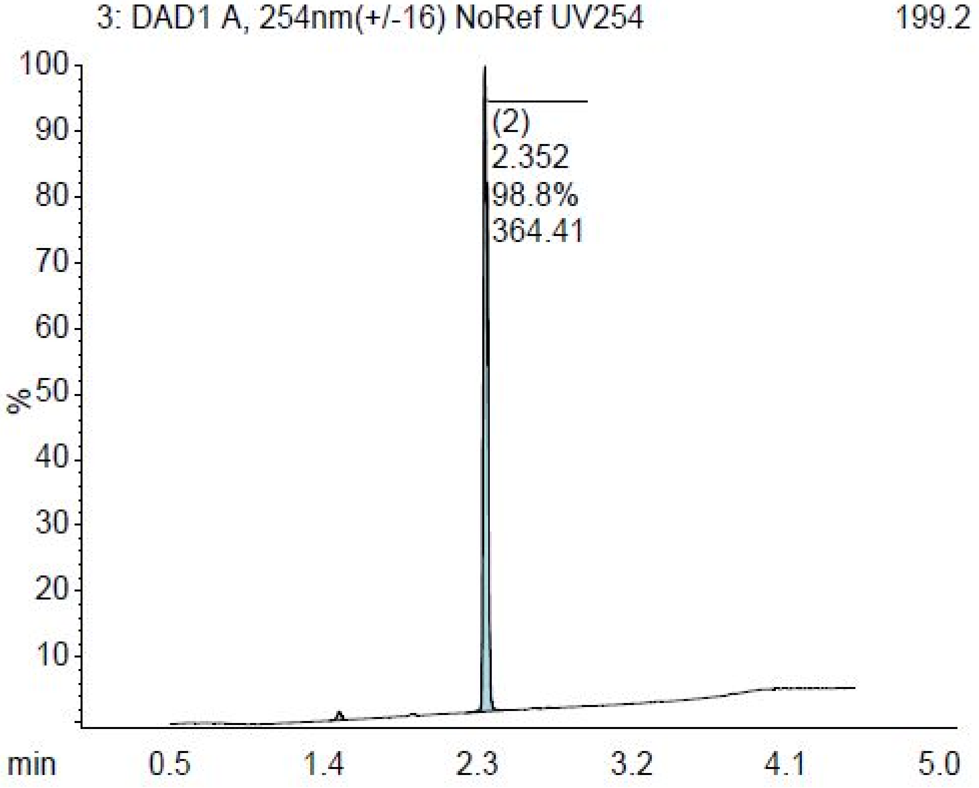

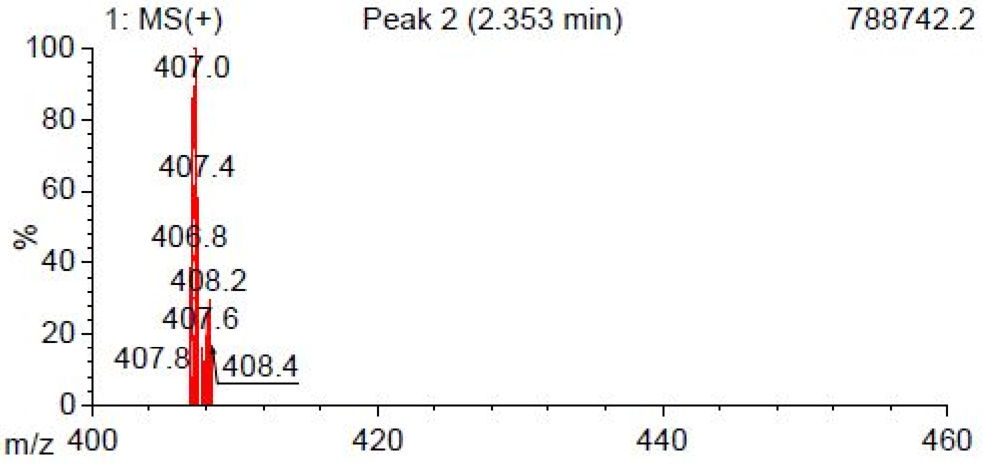

^{1}H spectra for compound 56

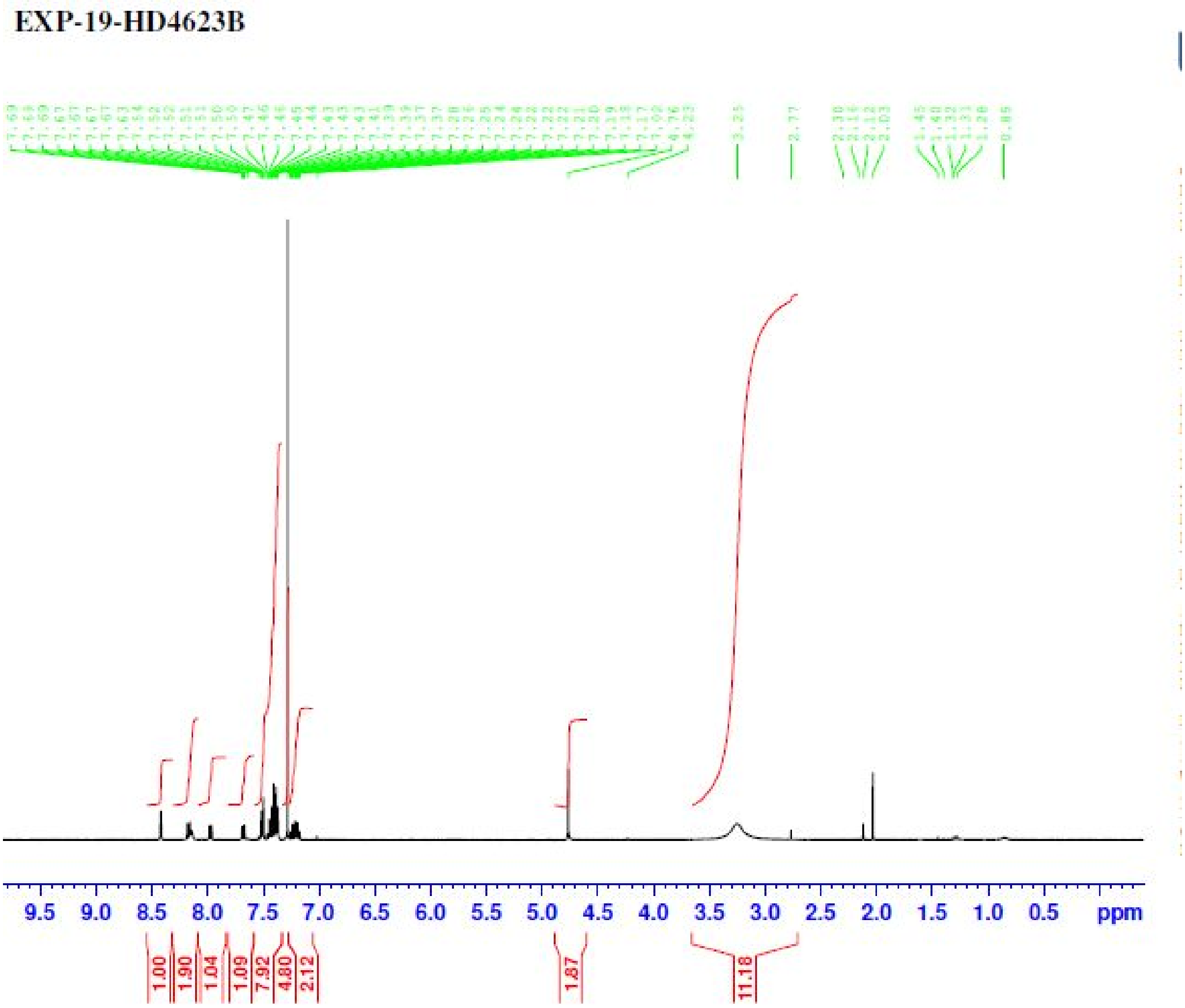

LCMS spectra for compound 56

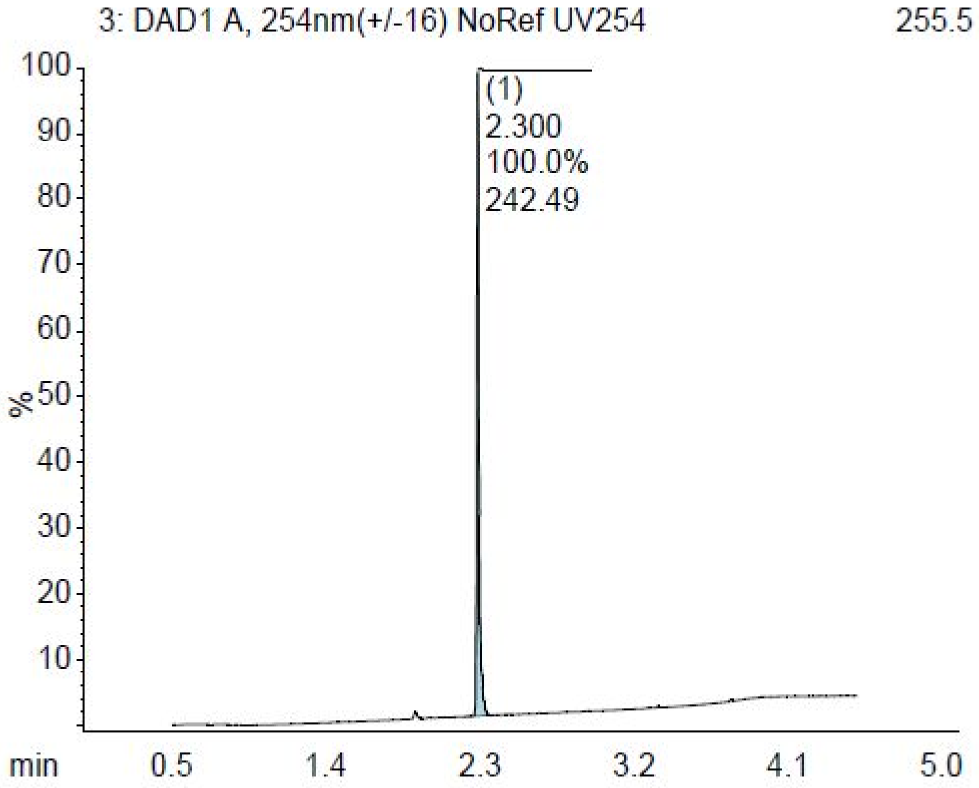

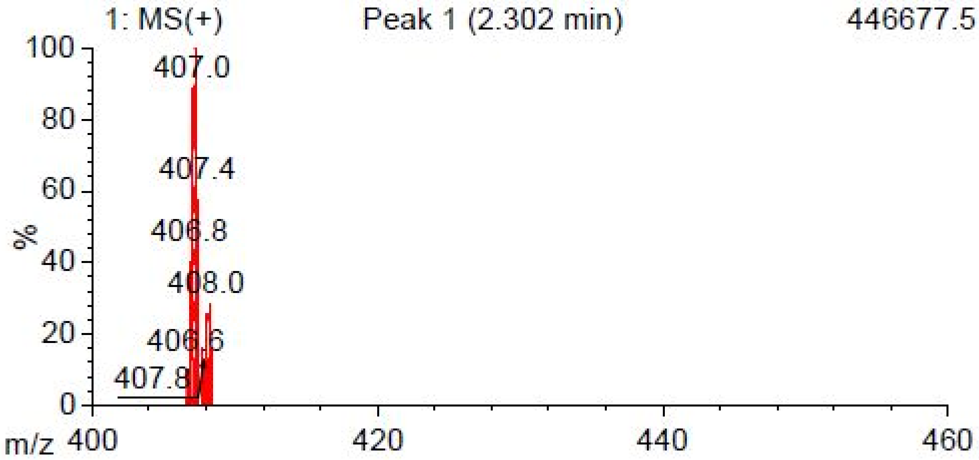

^{1}H spectra for compound 57

EXP- 19-HD4617

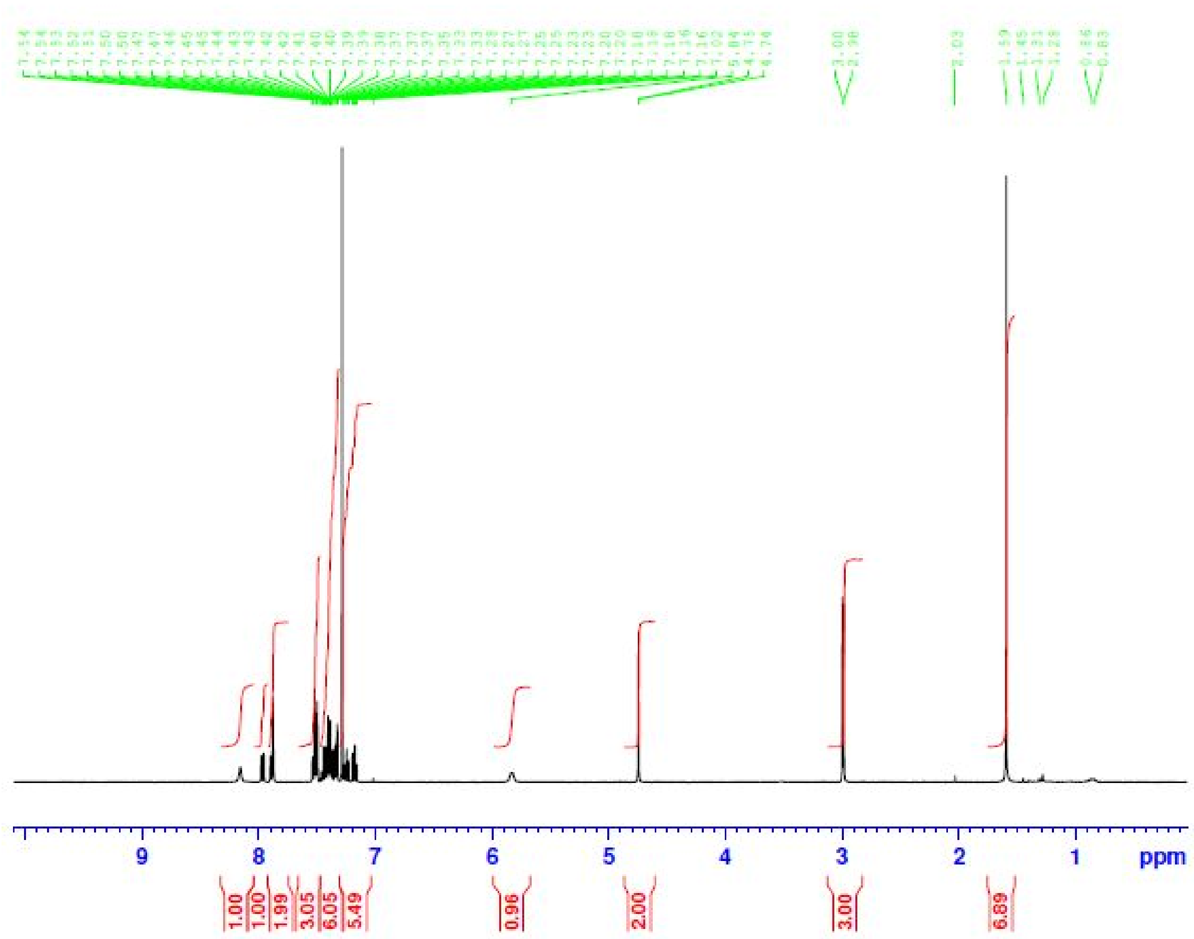

LCMS spectra for compound 57

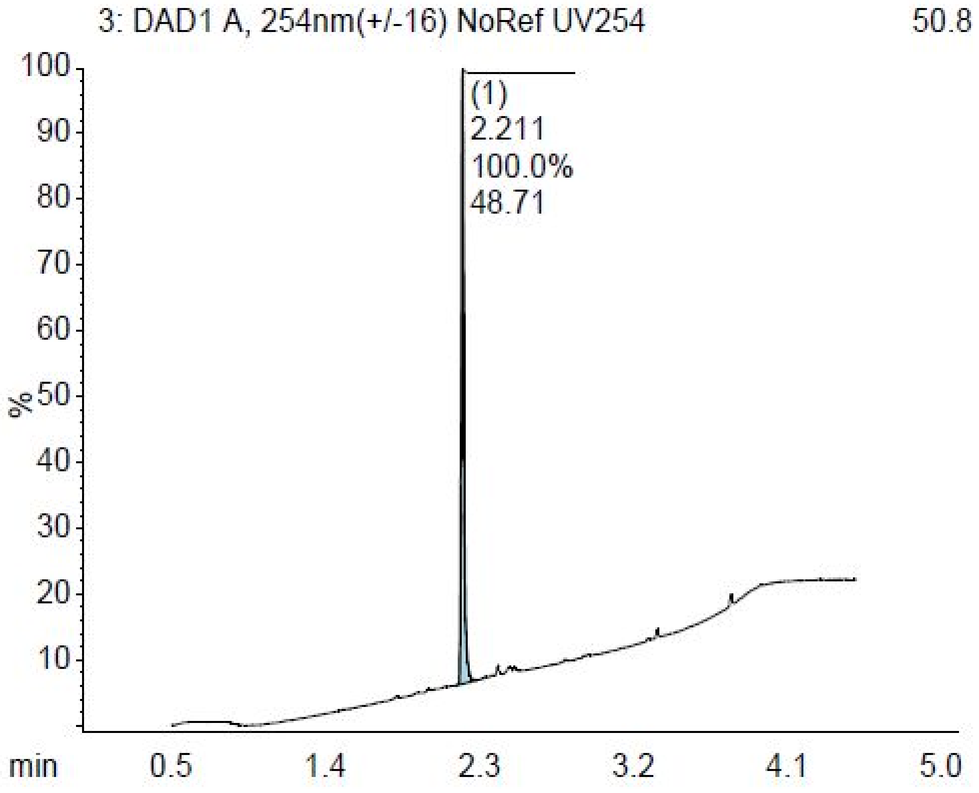

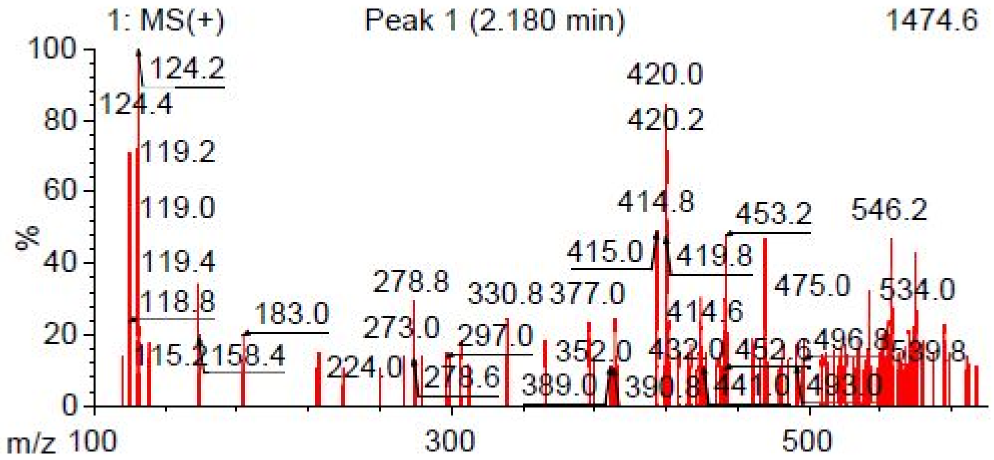

^{1}H spectra for compound 58

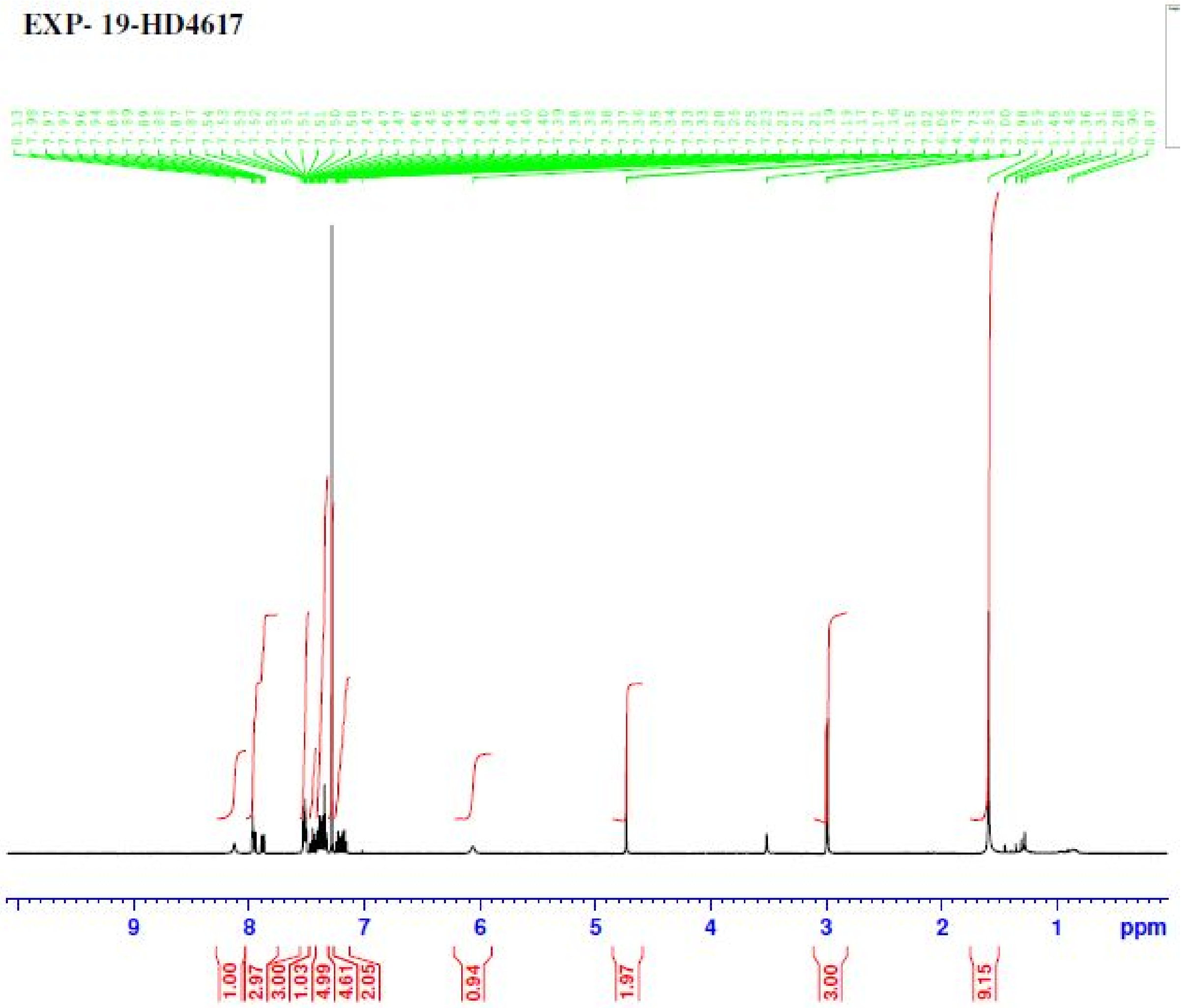

LCMS spectra for compound 58

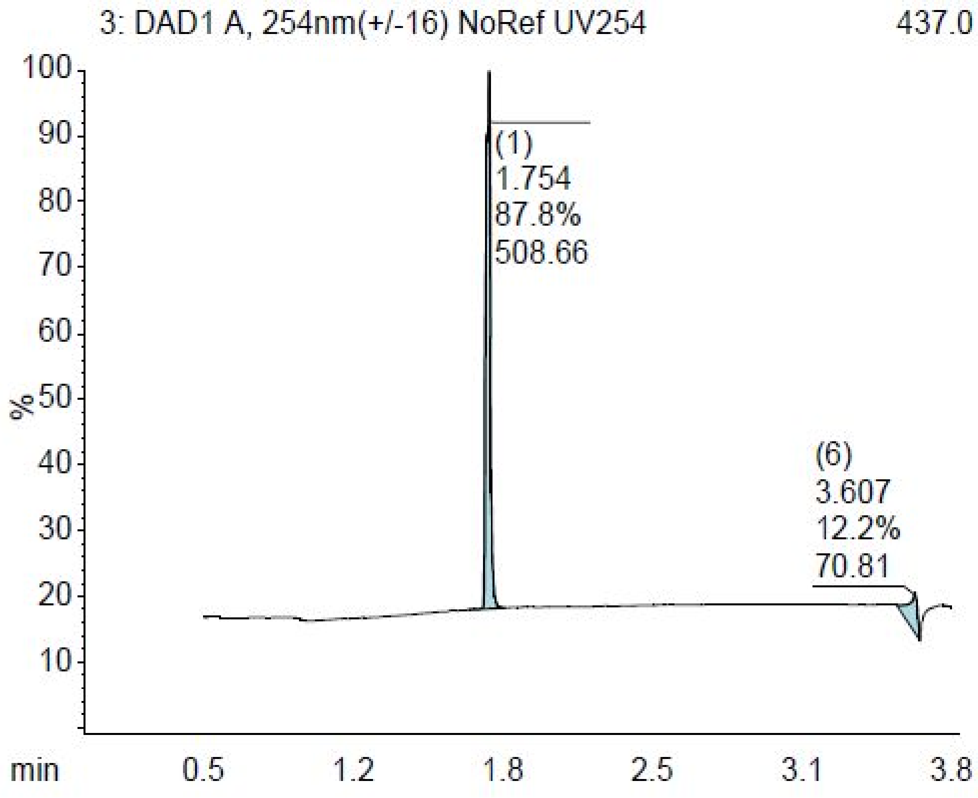

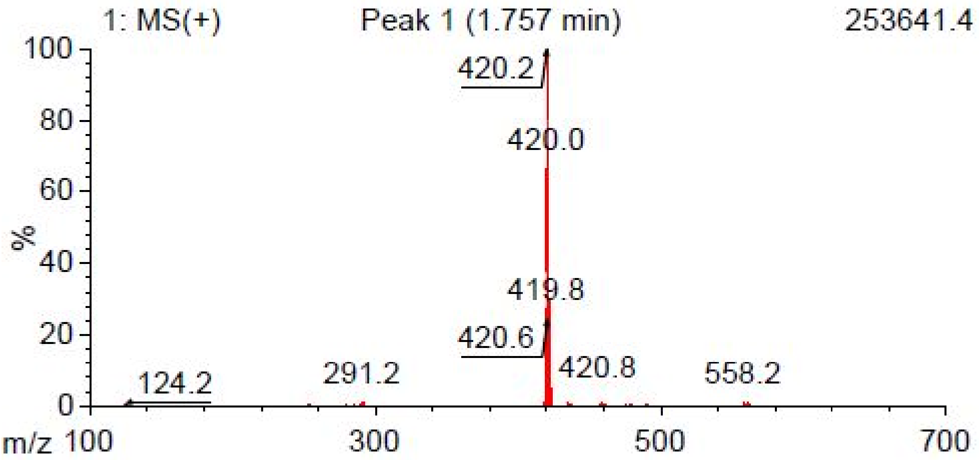

^{1}H spectra for compound 59

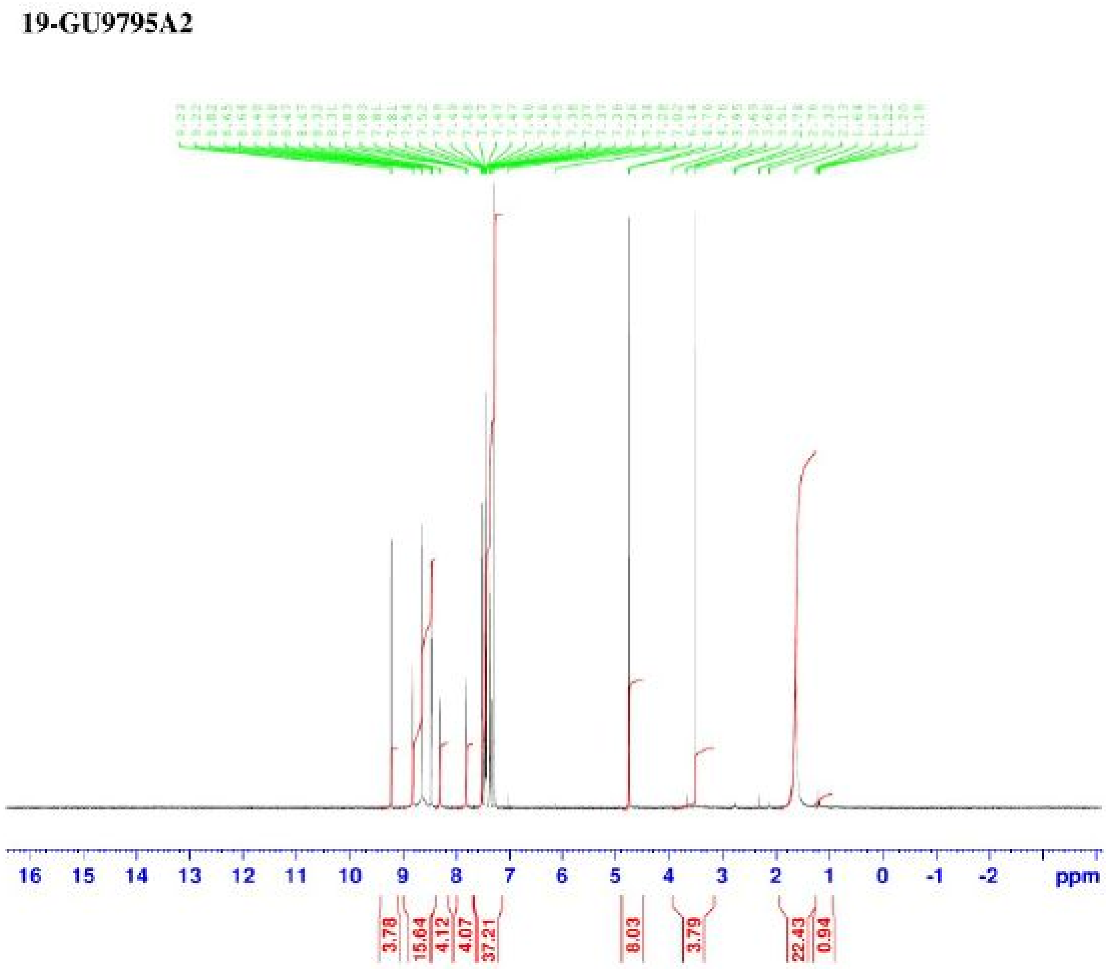

LCMS spectra for compound 59

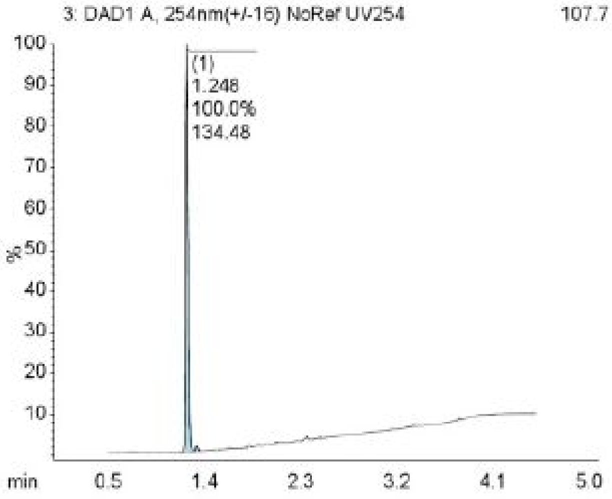

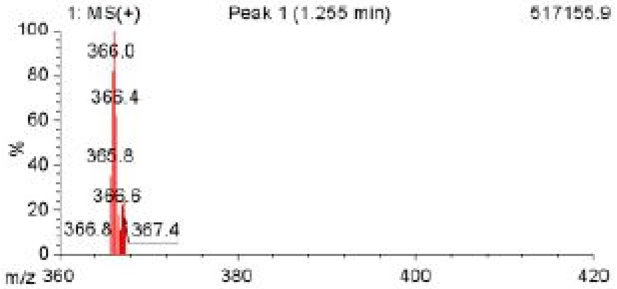

^{1}H spectra for compound 60

19-GU9795B2

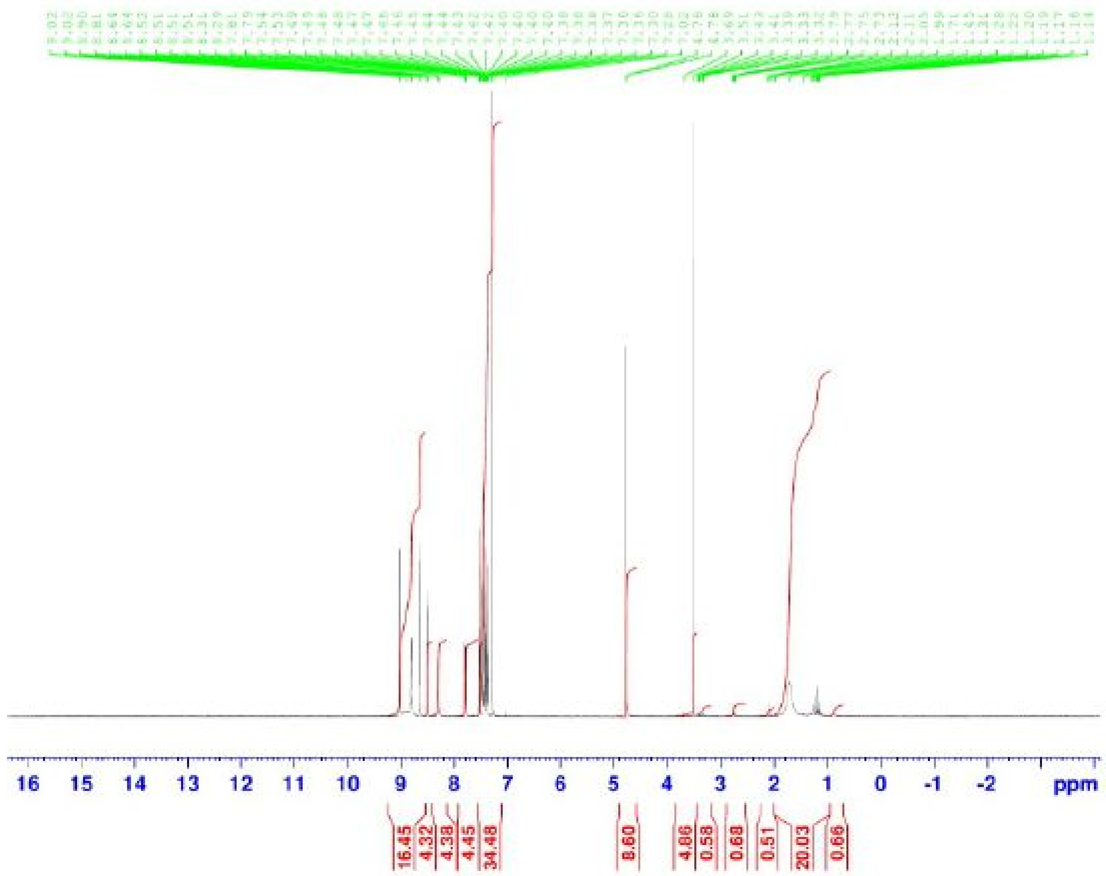

LCMS spectra for compound 60

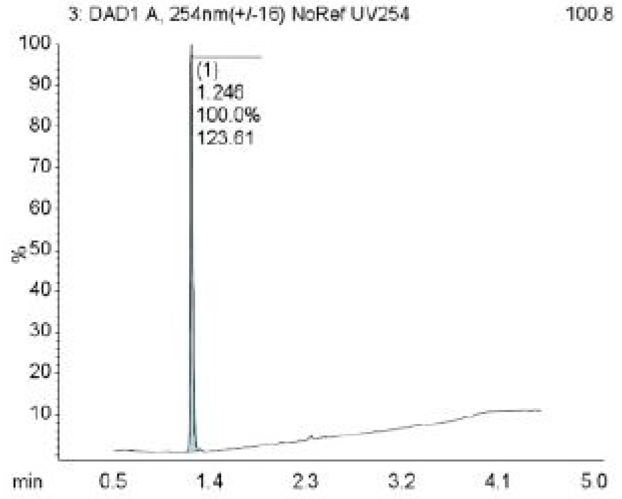

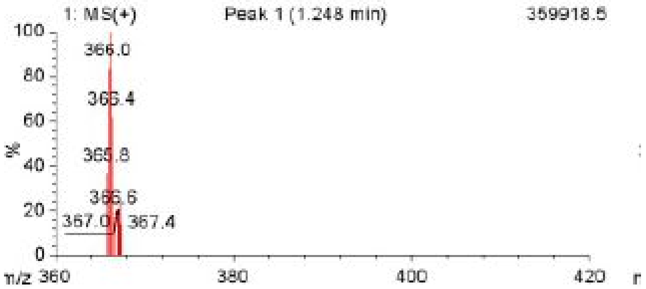

^{1}H spectra for compound 61

19-HE9420B

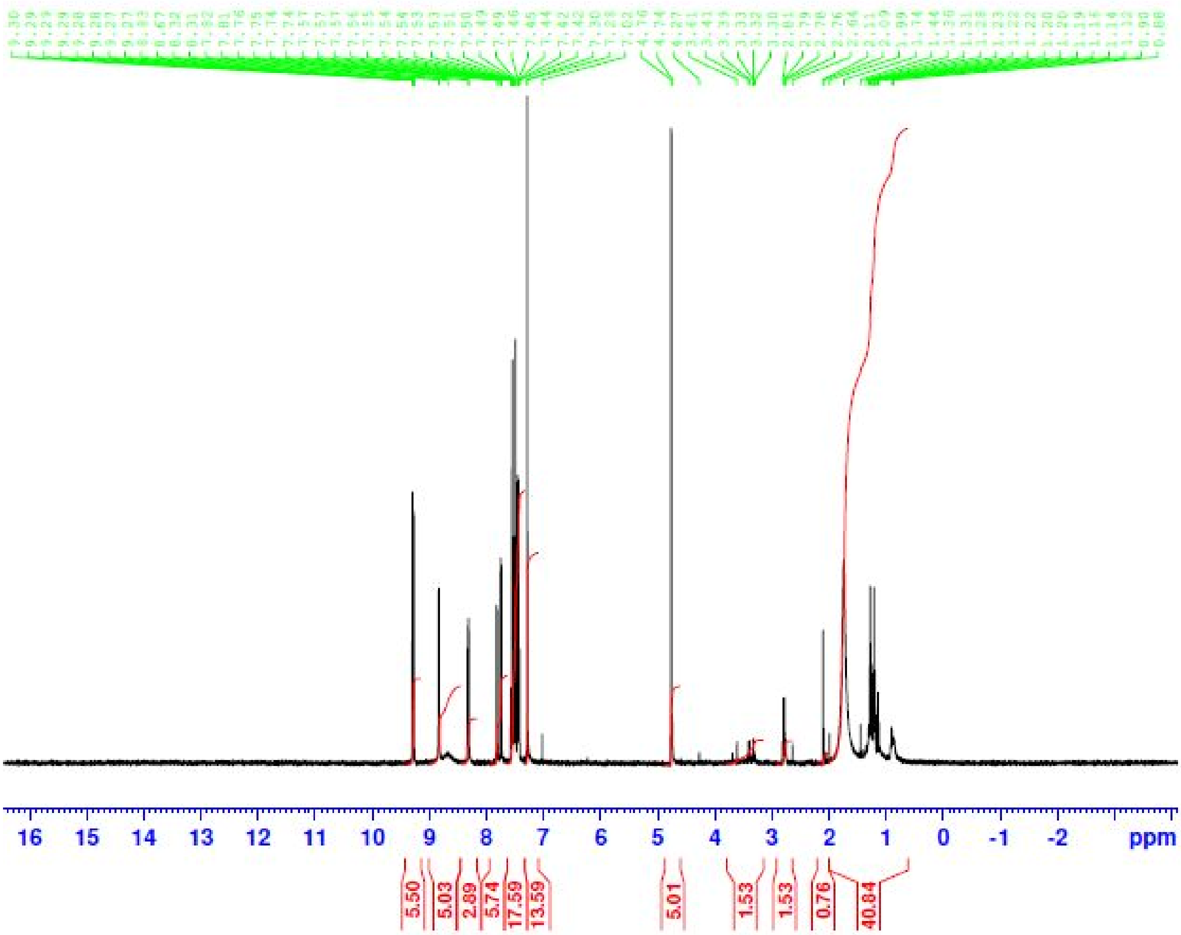

LCMS spectra for compound 61

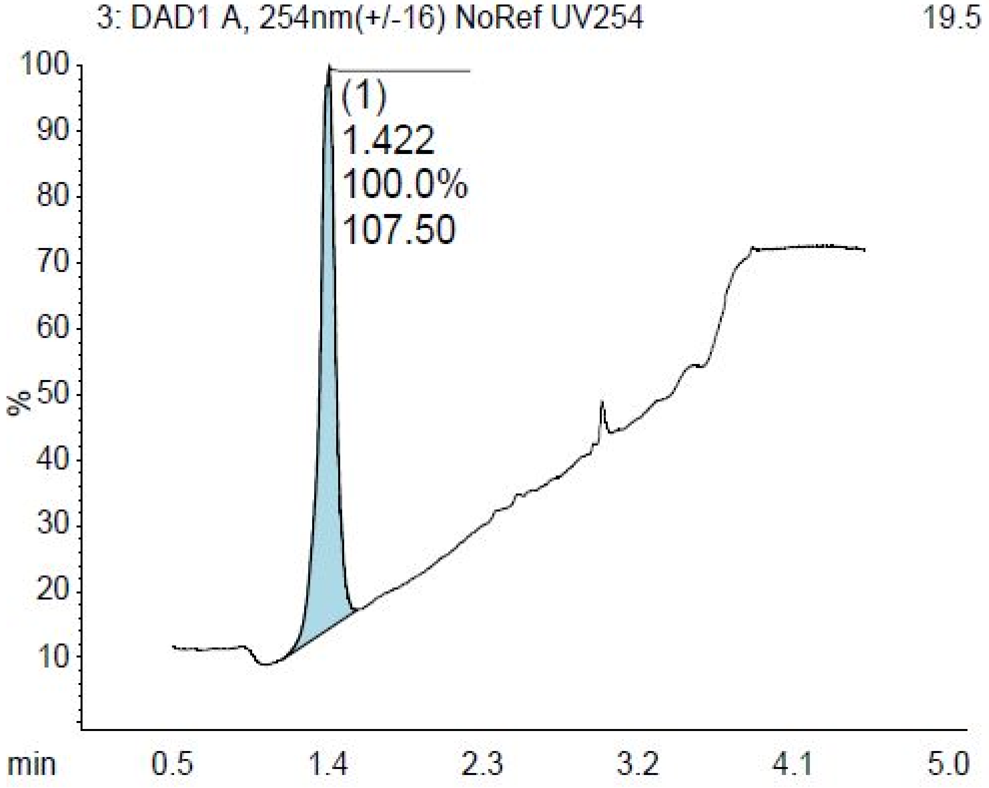

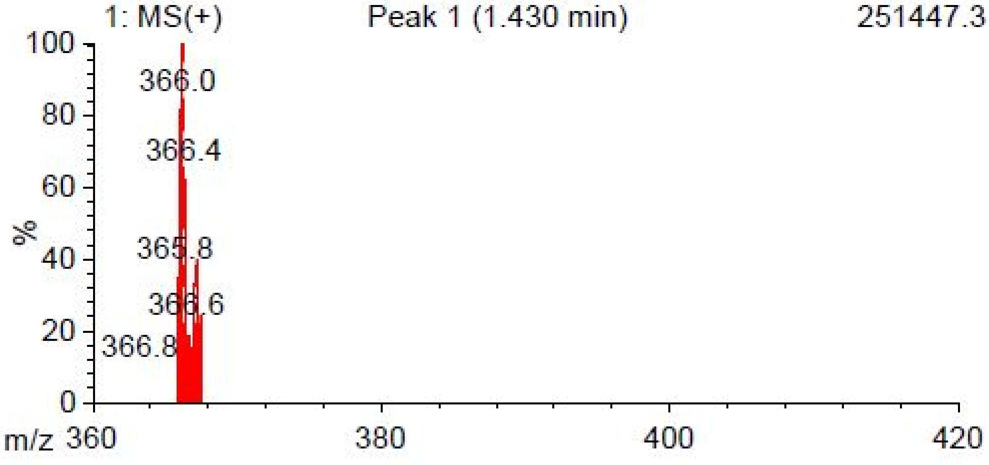

^{1}H spectra for compound 62

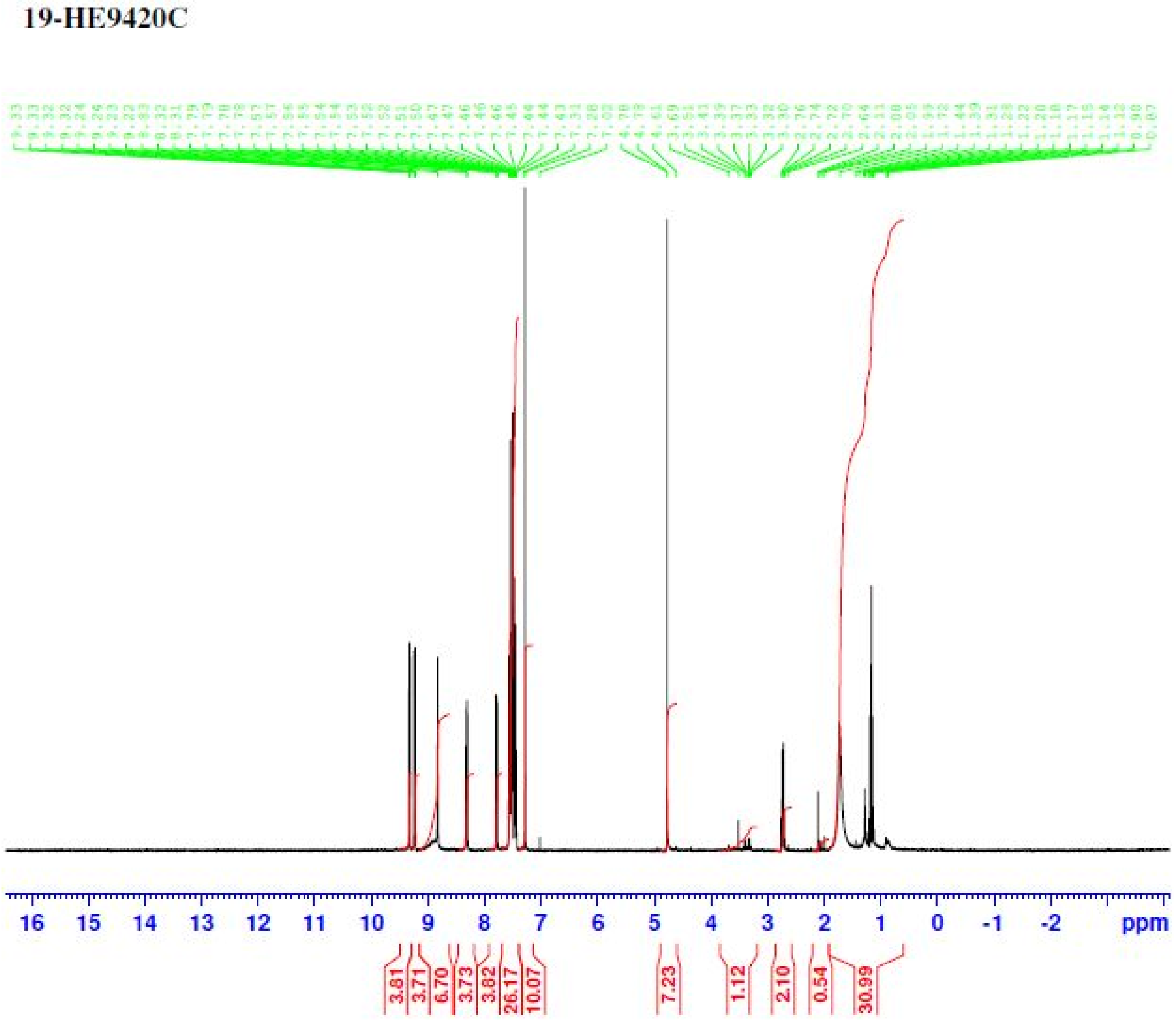

LCMS spectra for compound 62

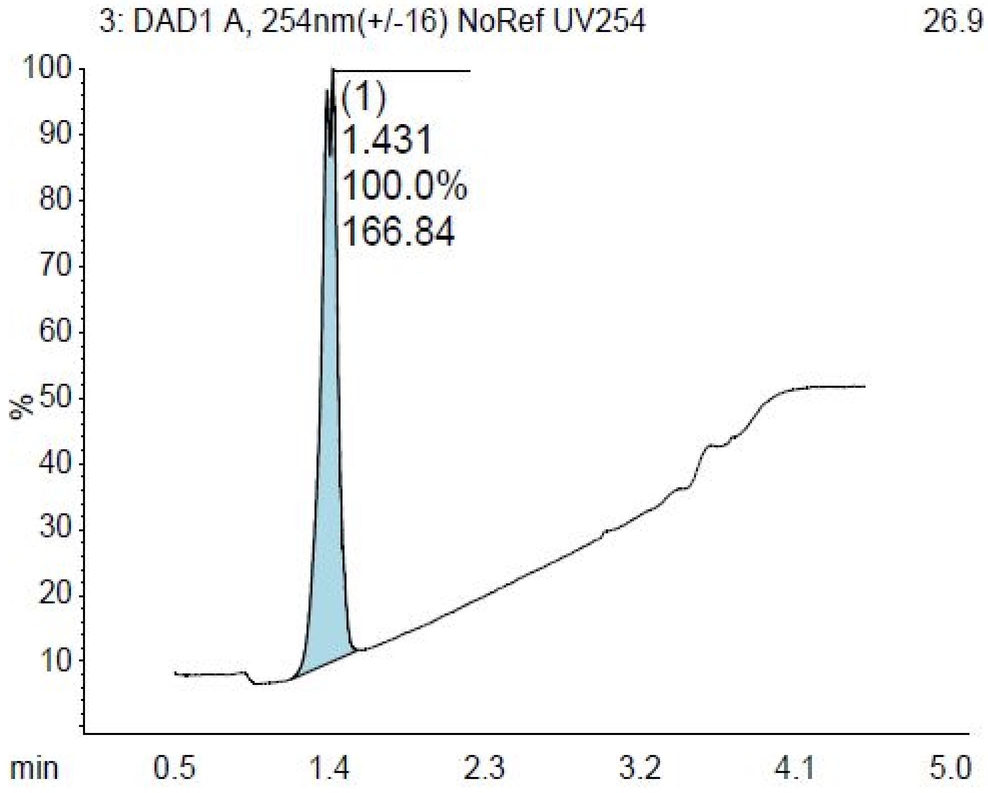

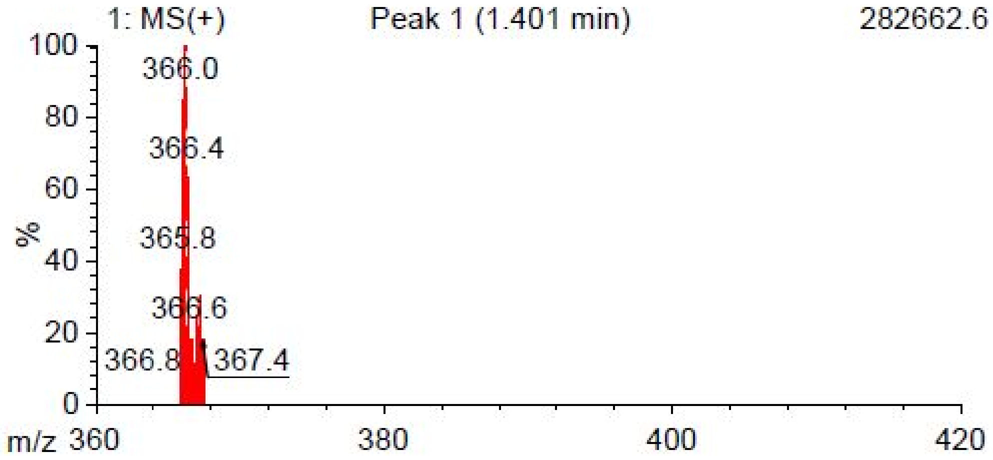

^{1}H spectra for compound 63

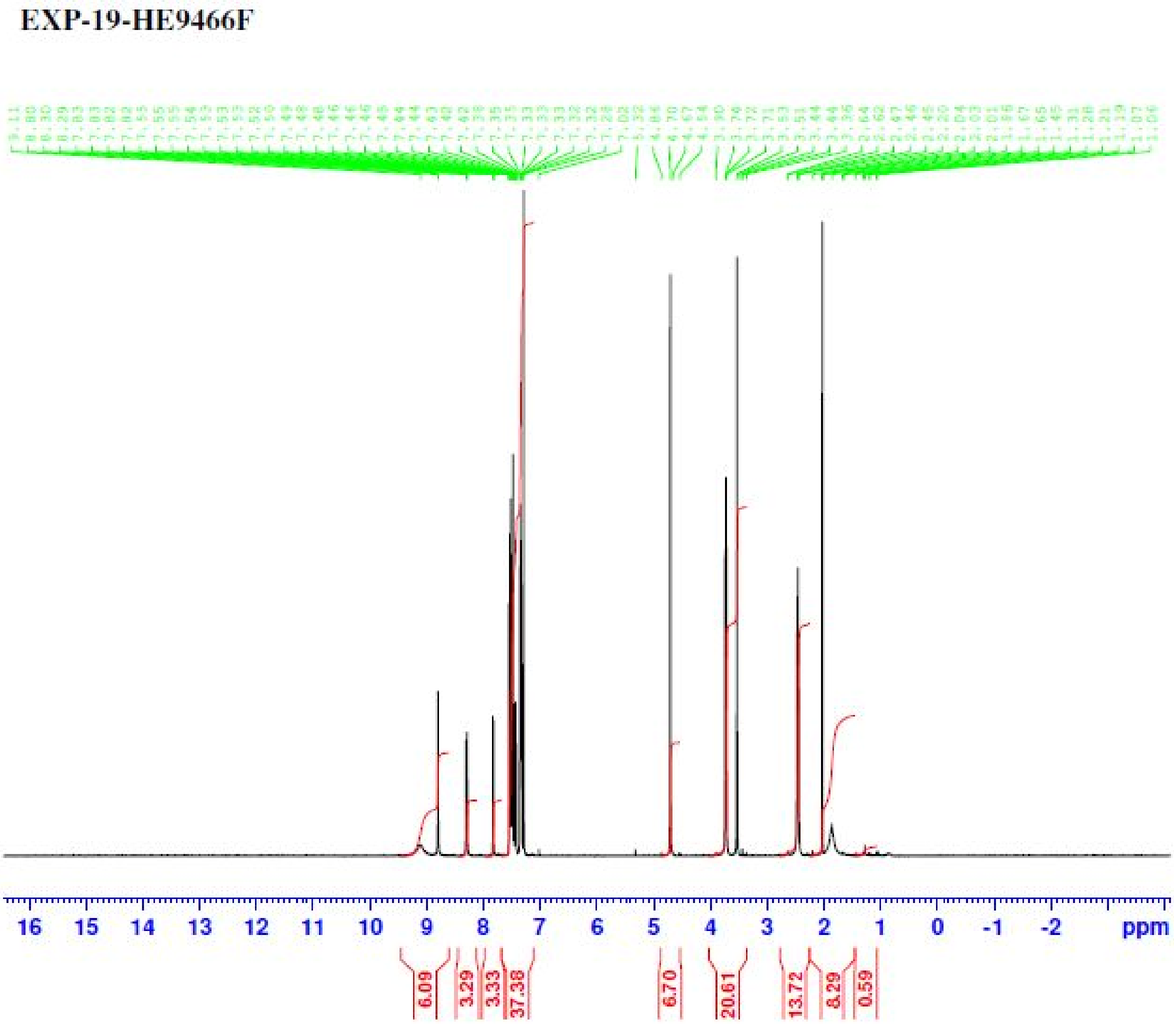

LCMS spectra for compound 63

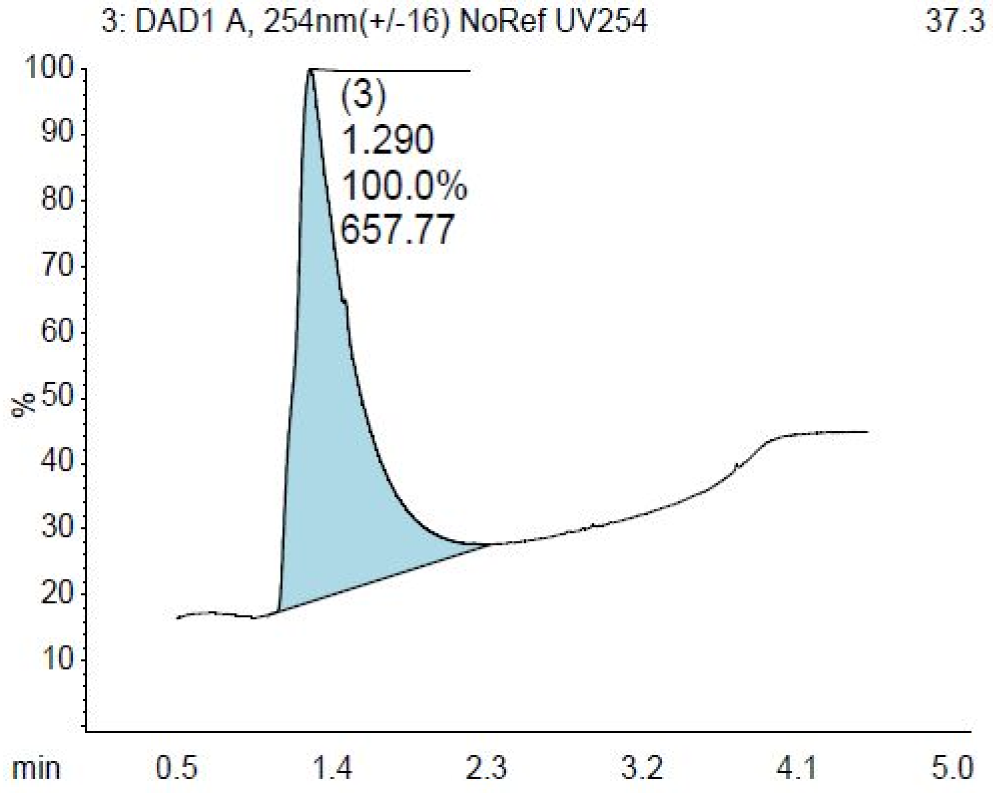

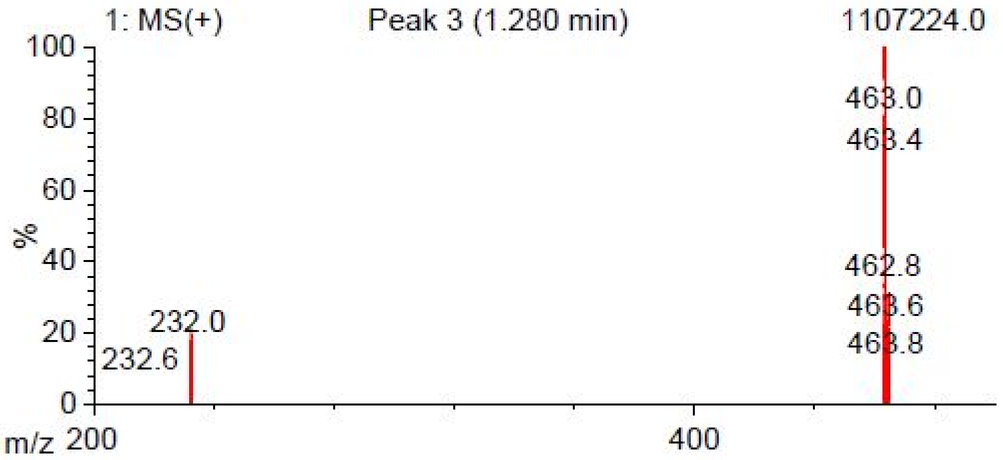

^{1}H spectra for compound 64

EXP-19-HE9466E

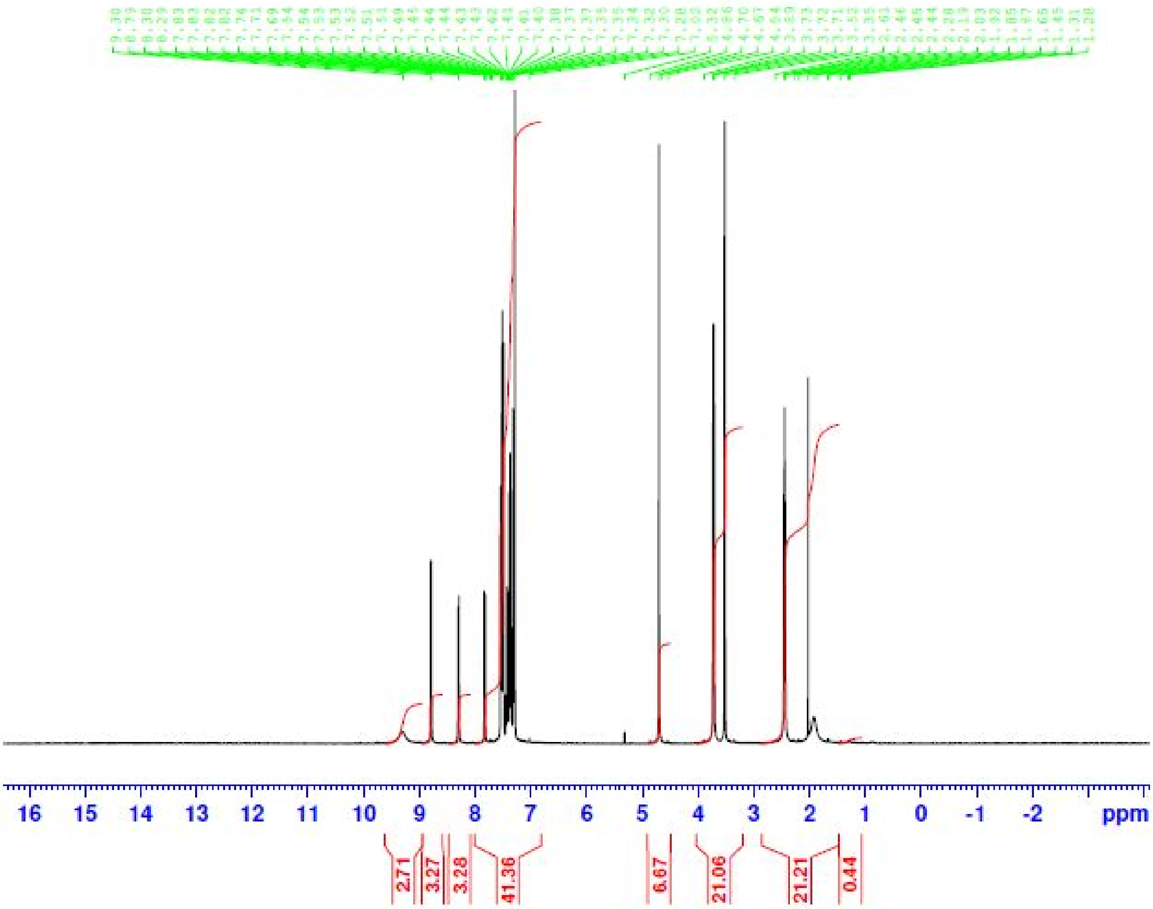

LCMS spectra for compound 64

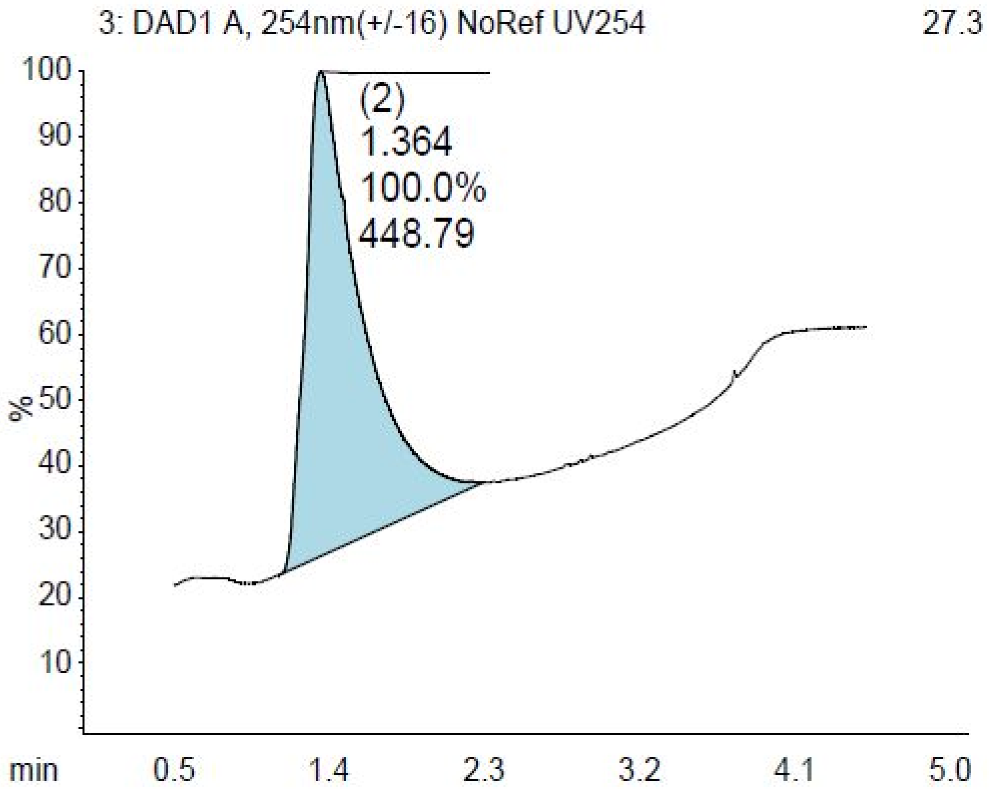

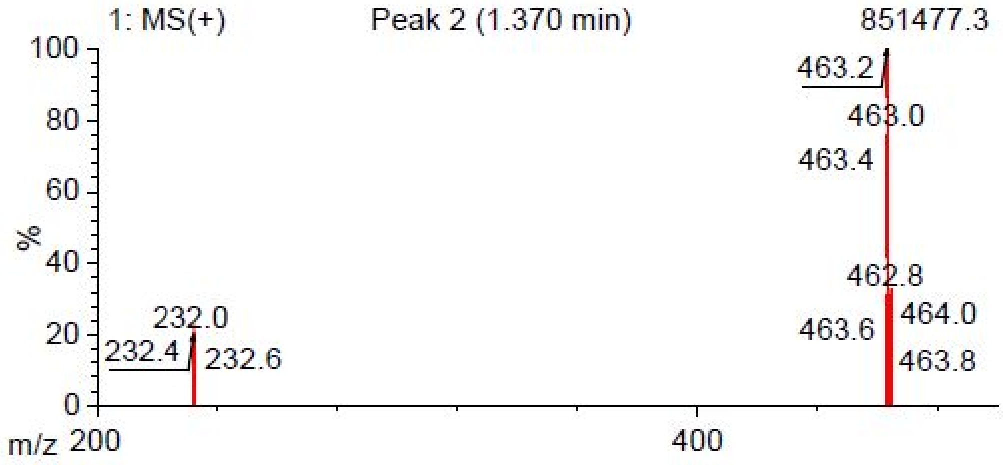

^{1}H spectra for compound 65

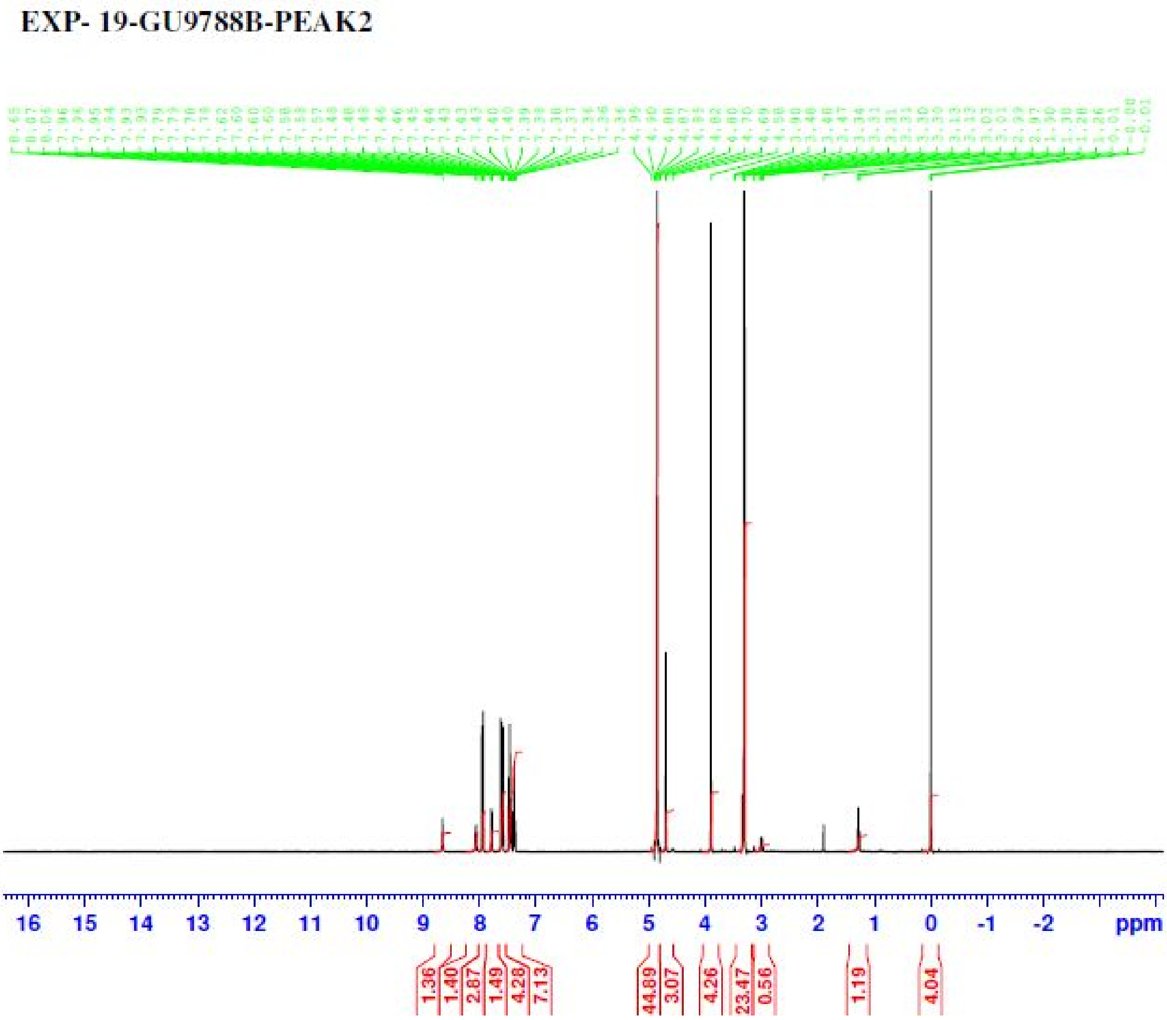

LCMS spectra for compound 65

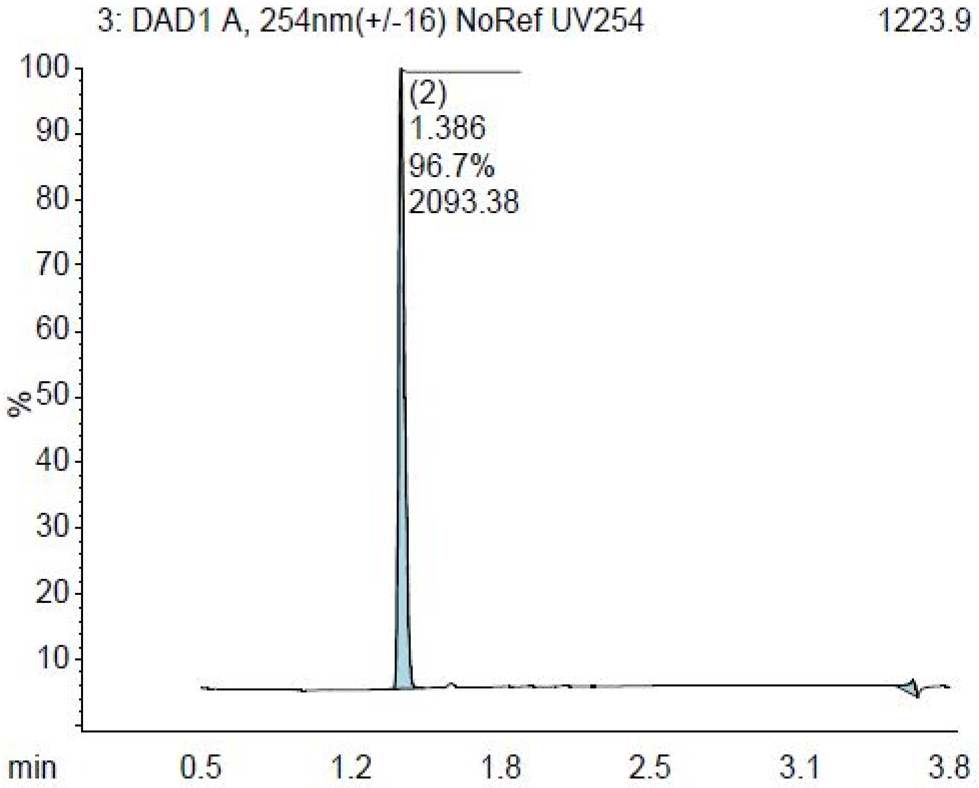

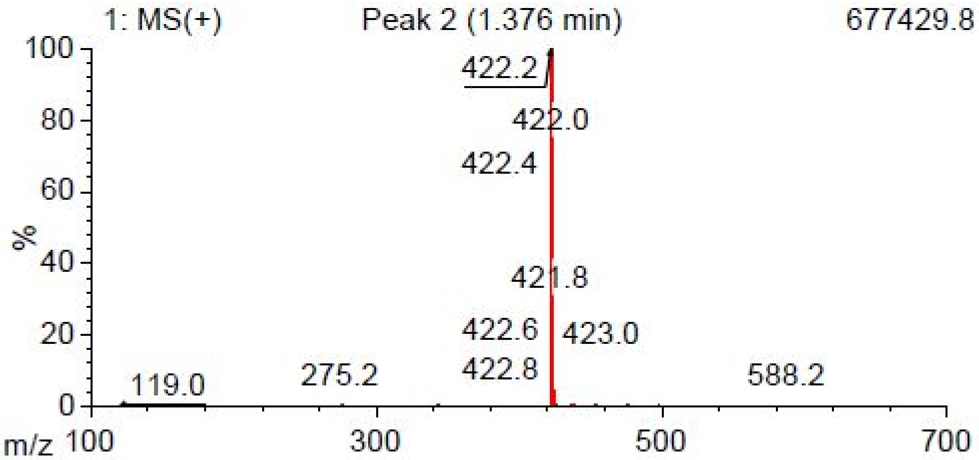

^{1}H spectra for compound 66

EXP- 19-GU9788A-PK1

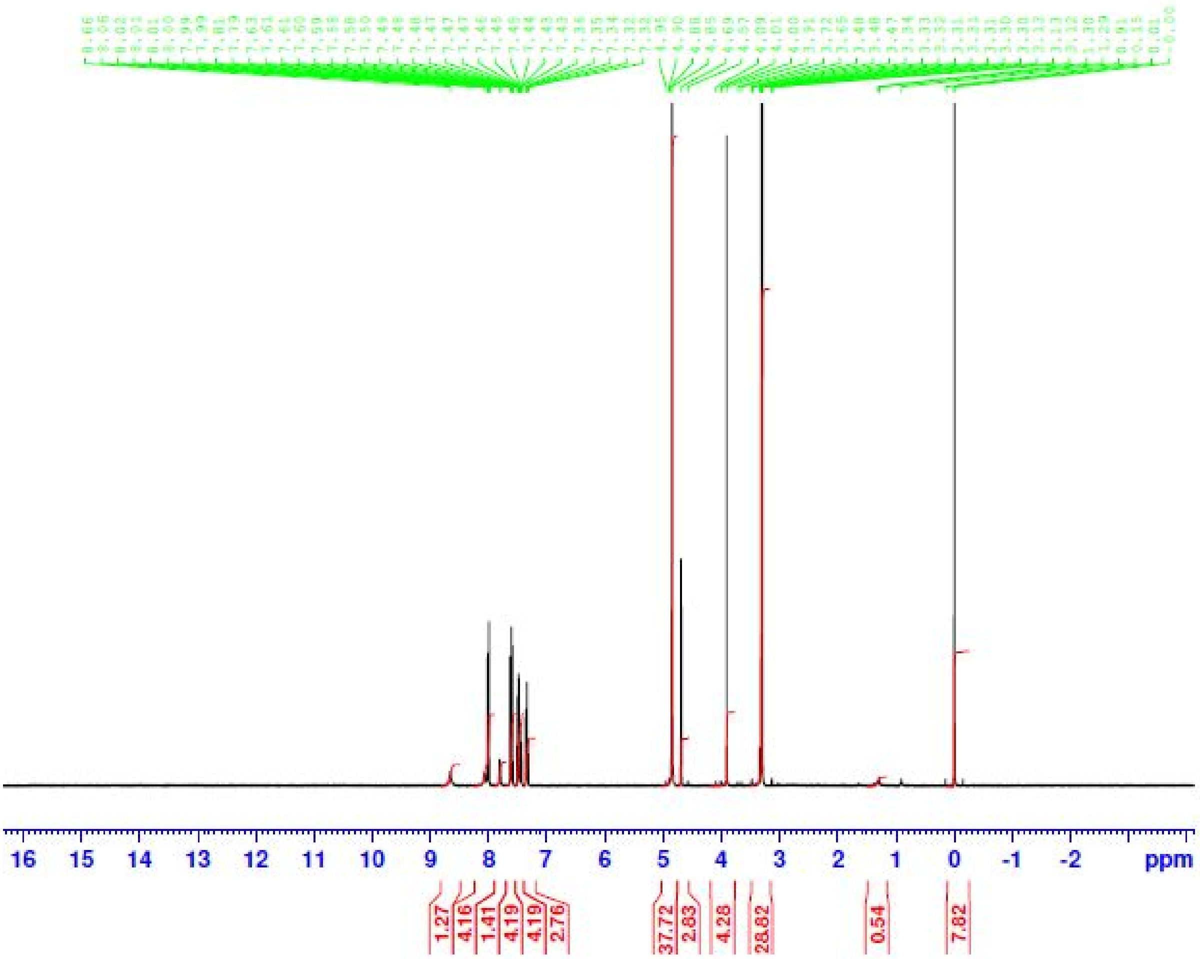

LCMS spectra for compound 66

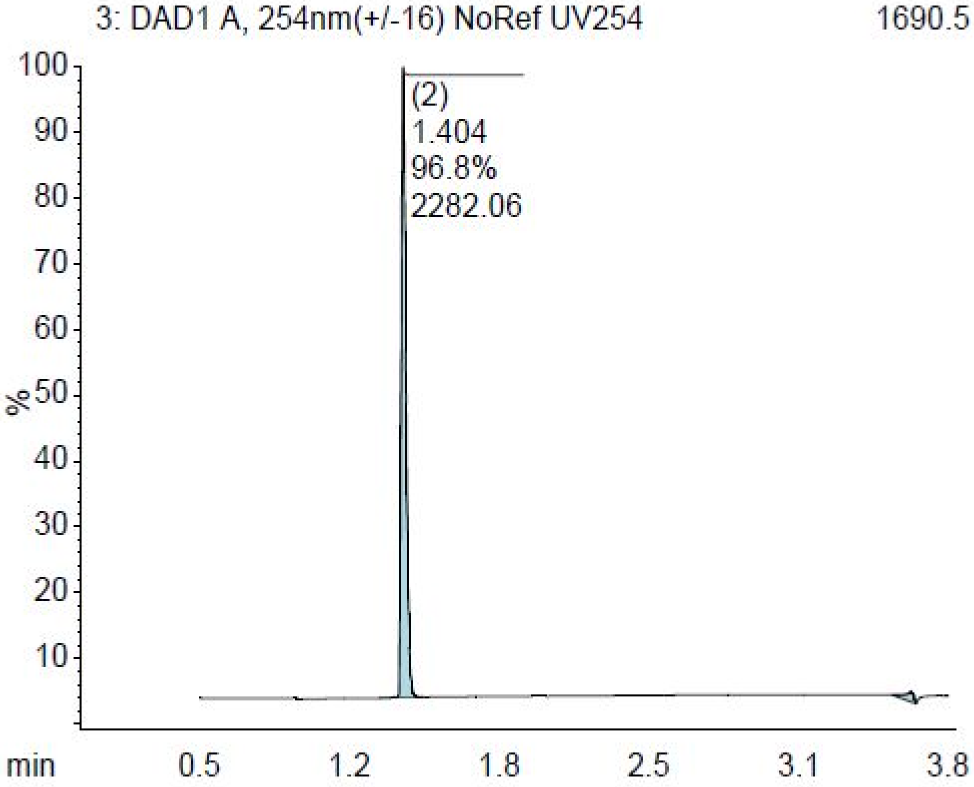

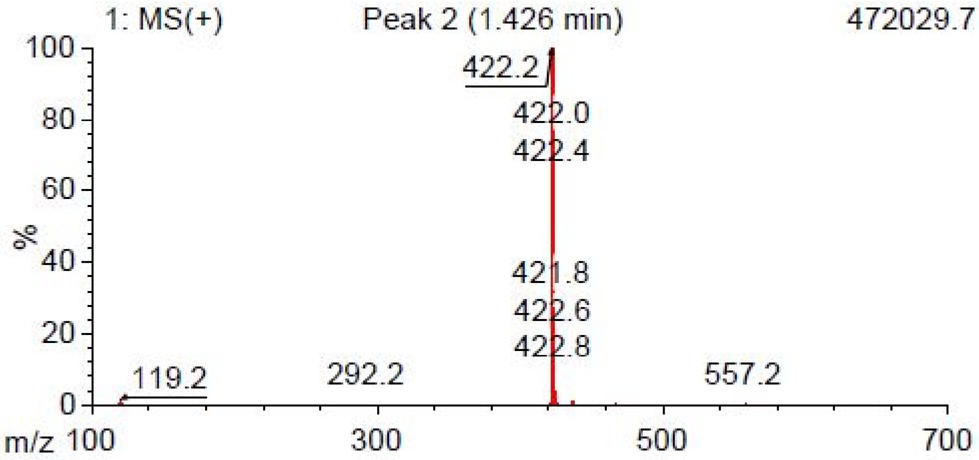

^{1}H spectra for compound 67

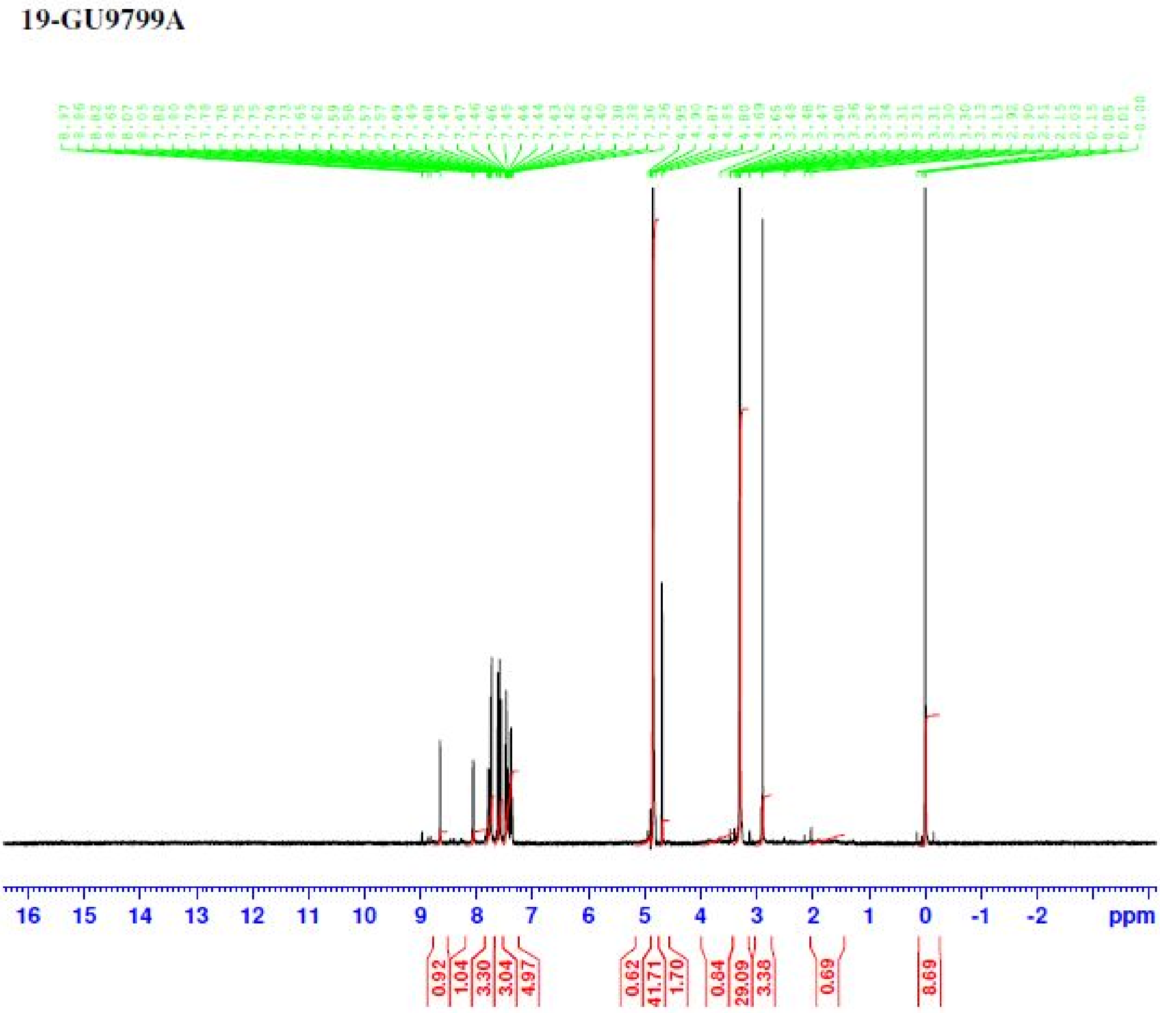

LCMS spectra for compound 67

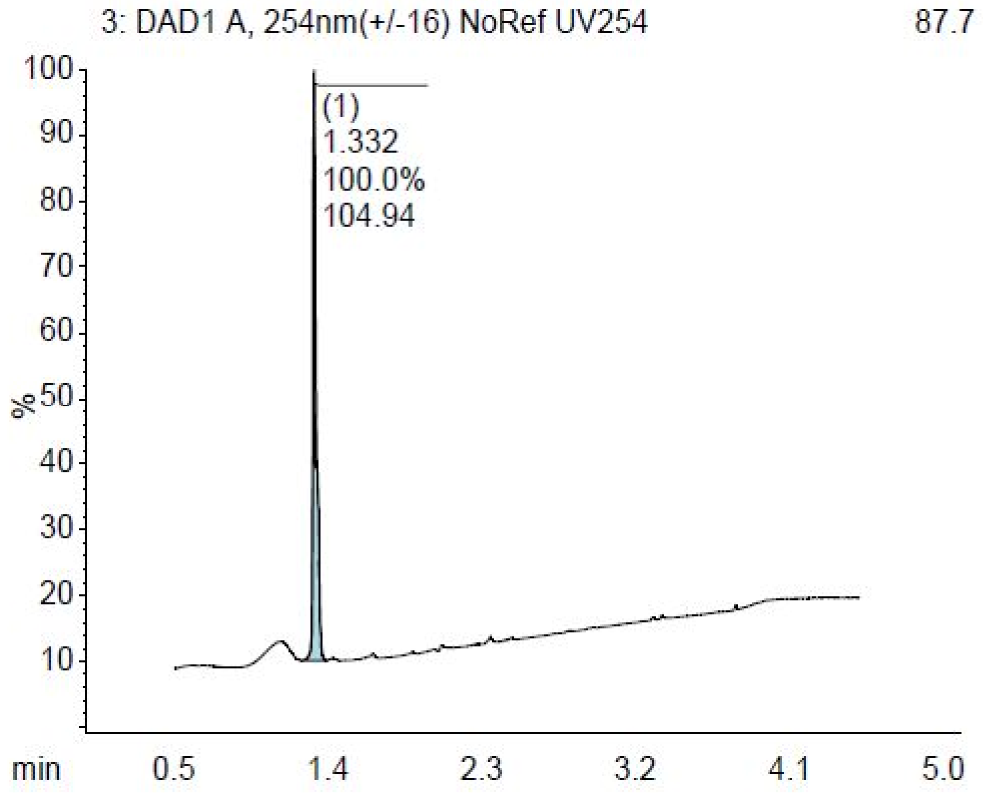

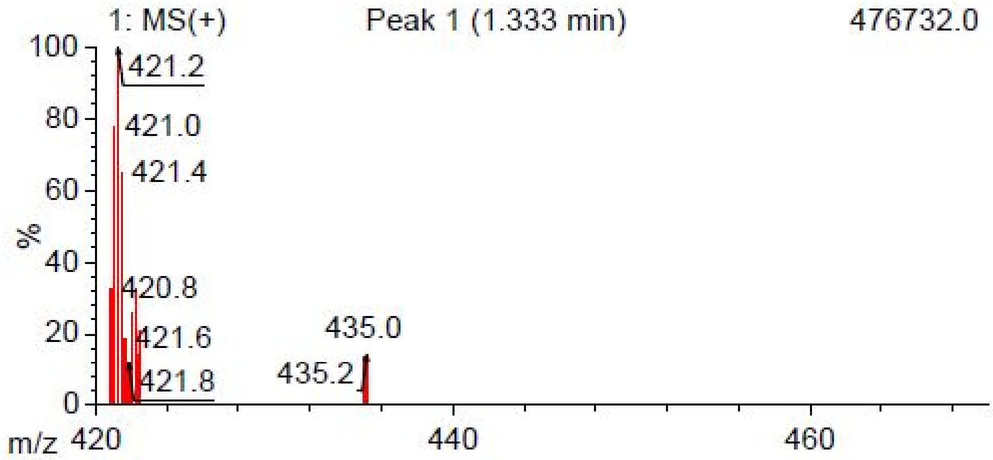

^{1}H spectra for compound 68

19-GU9798A

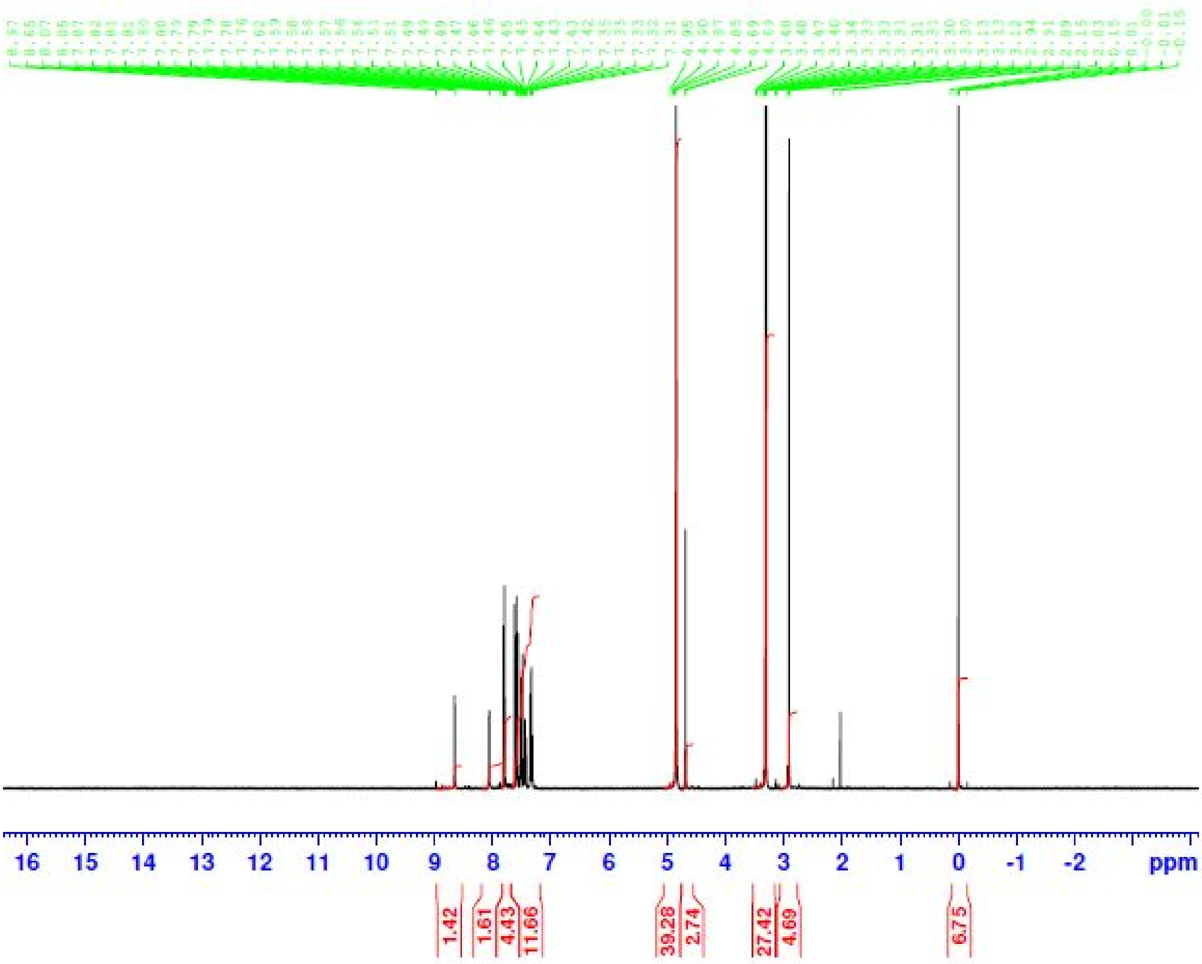

LCMS spectra for compound 68

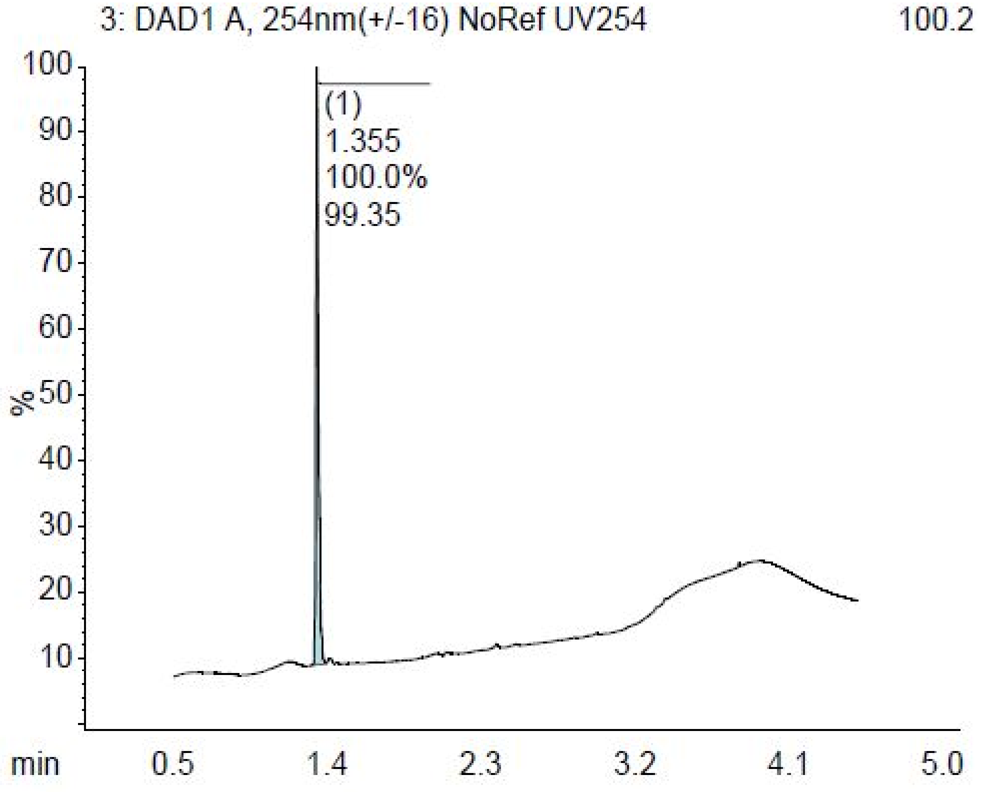

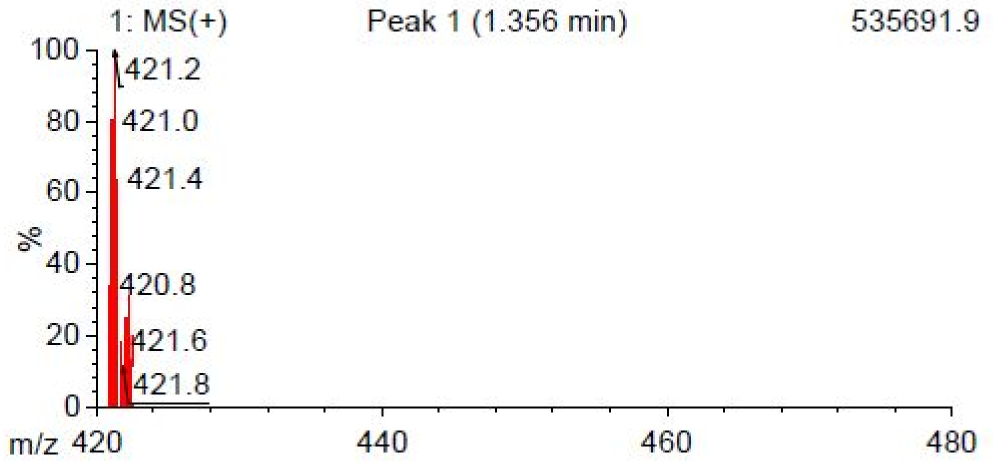

^{1}H spectra for compound 69

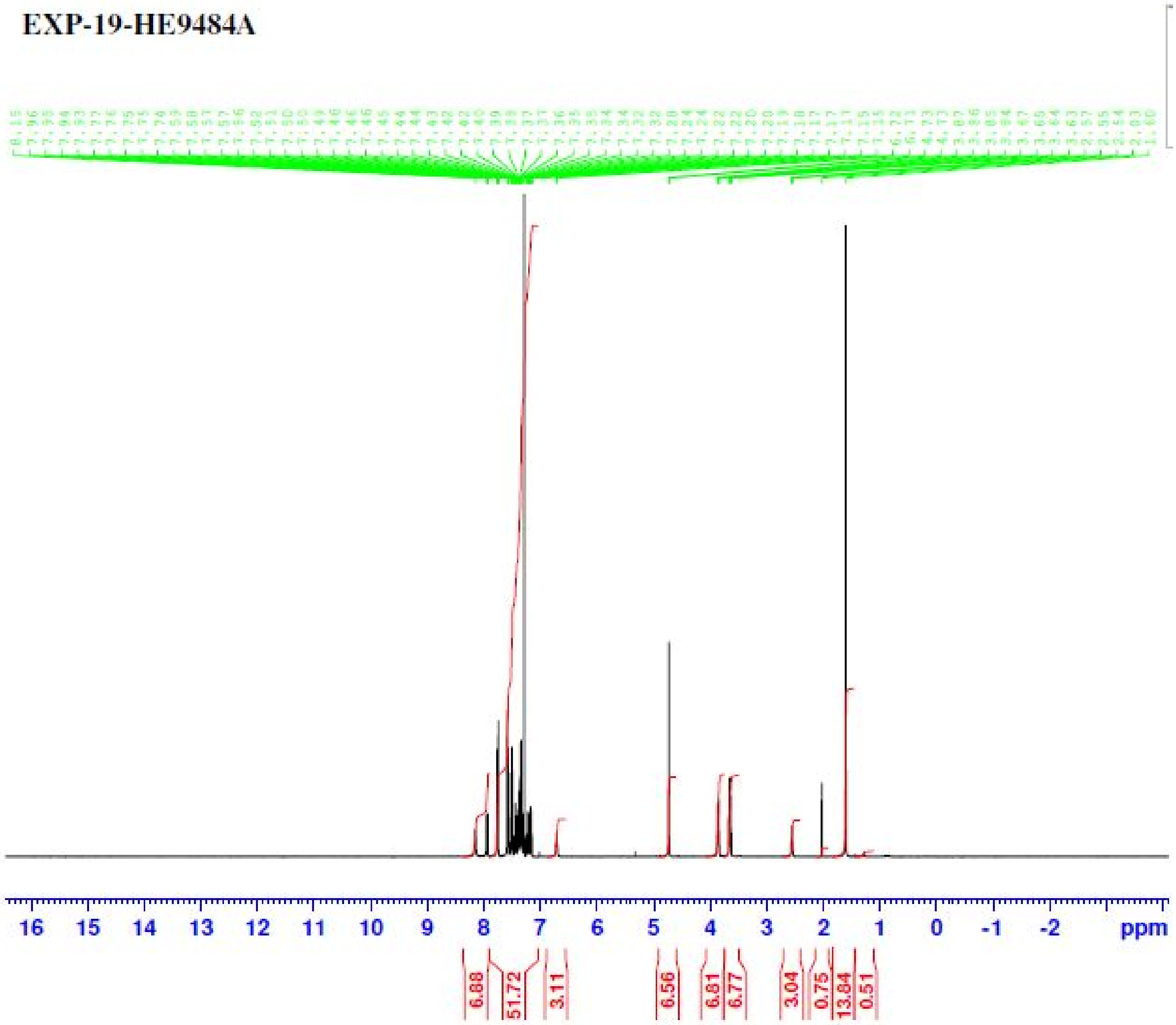

LCMS spectra for compound 69

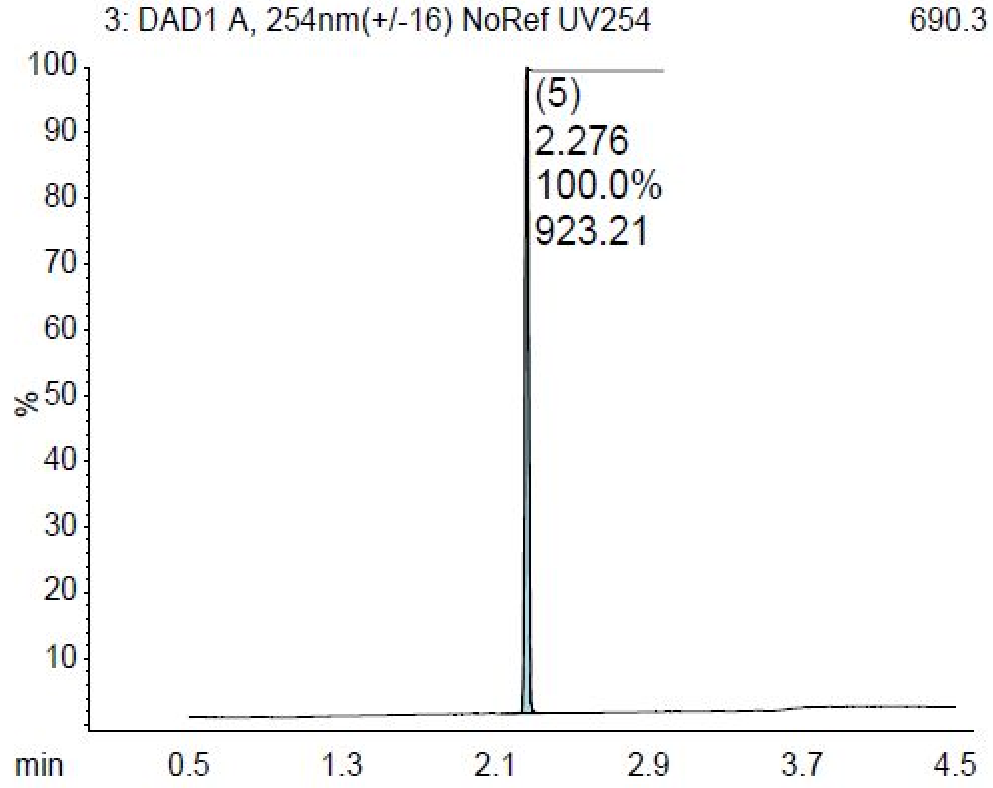

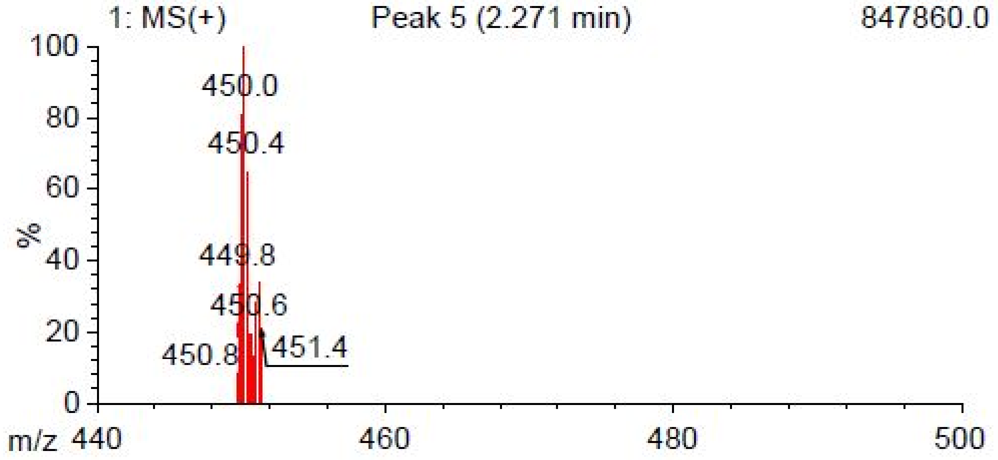

¹H spectra for compound 70

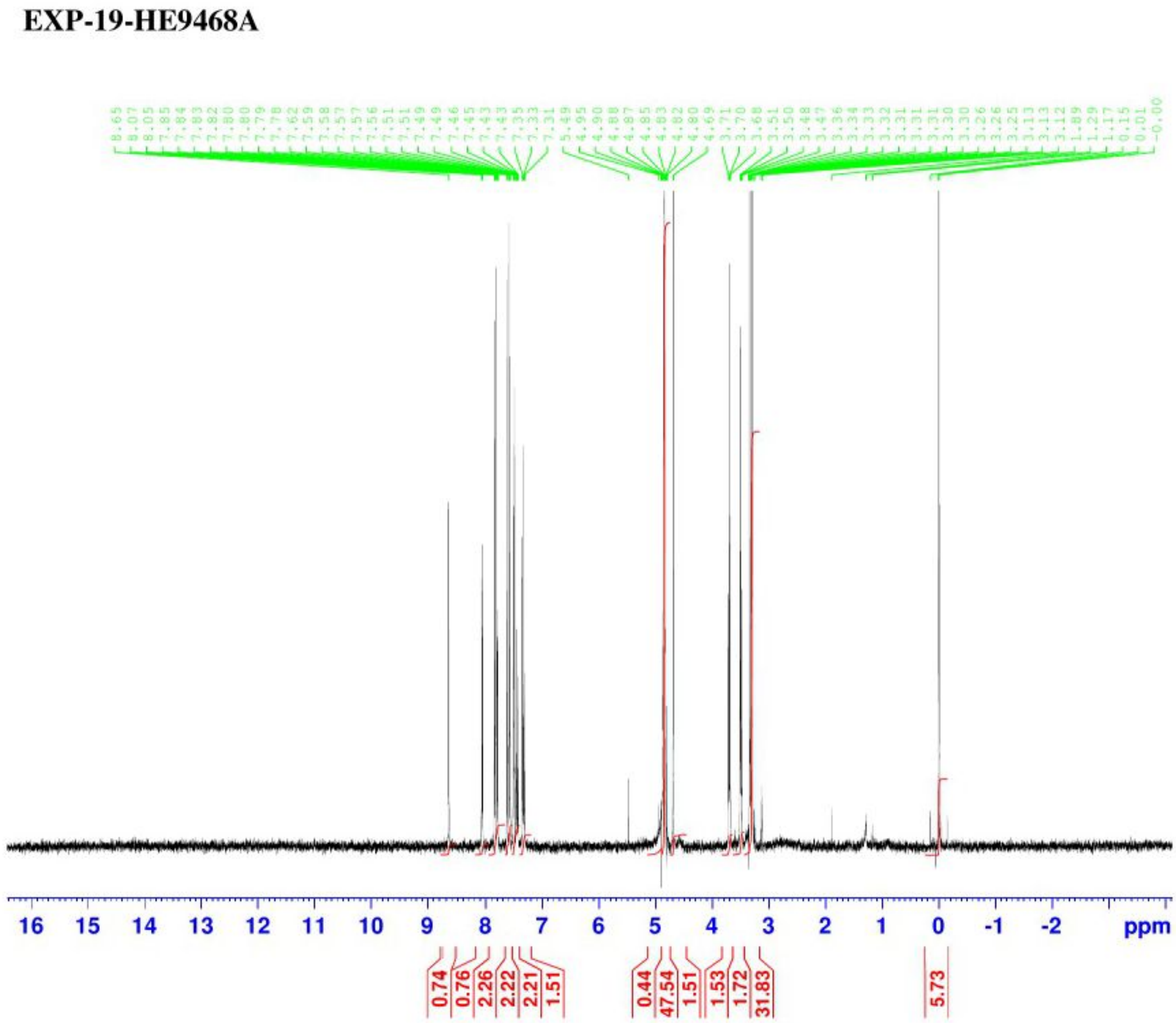

LCMS spectra for compound 70

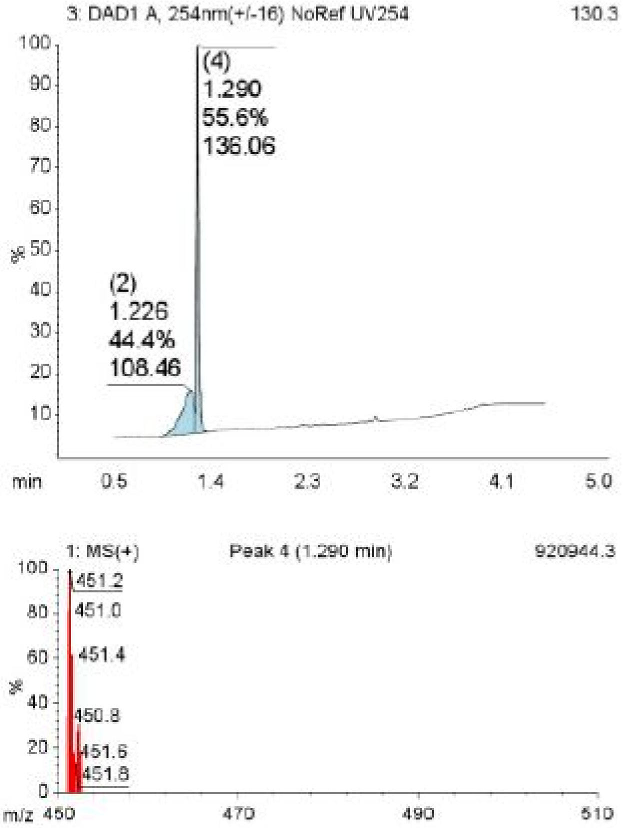

^{1}H spectra for compound 71

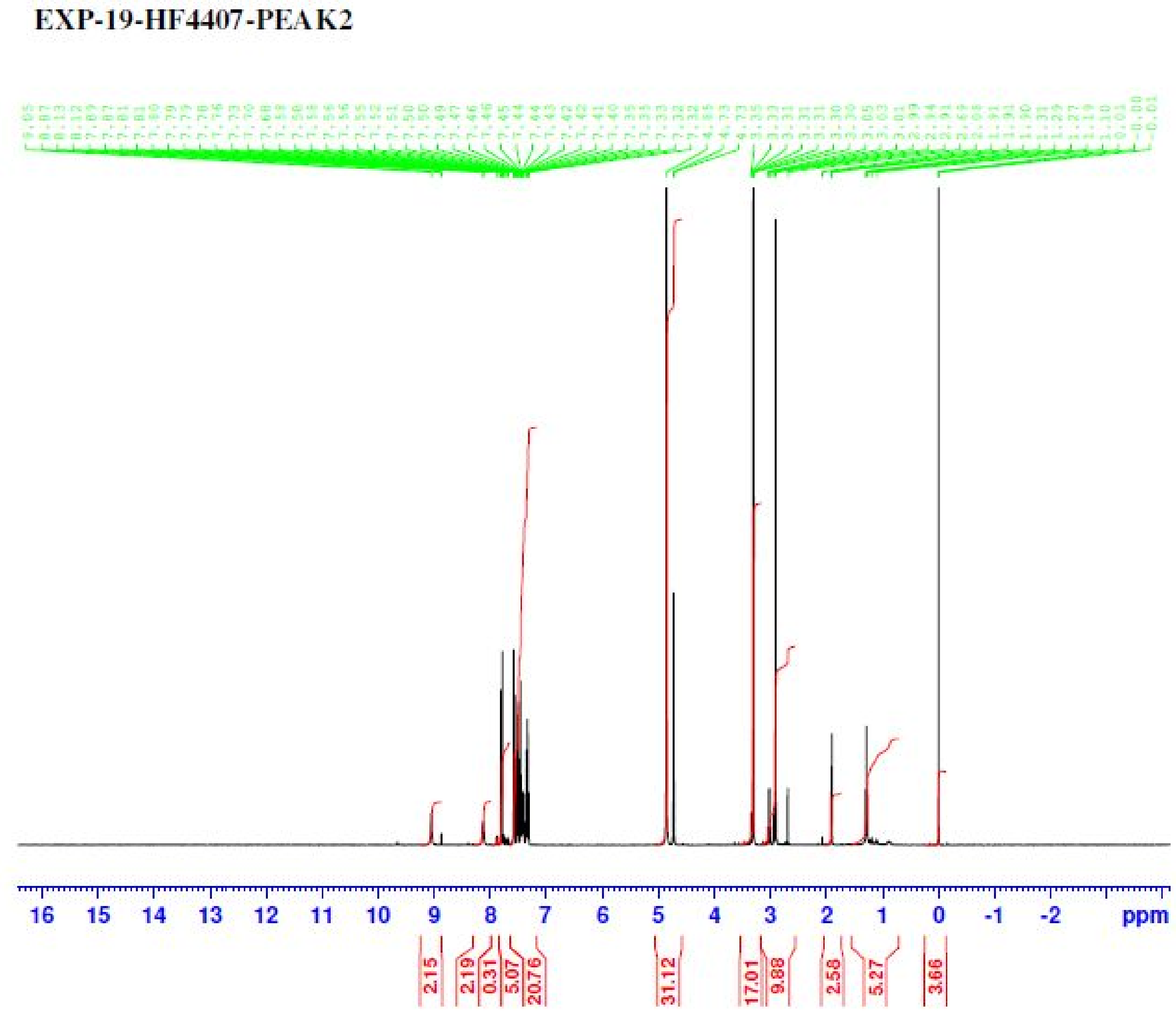

LCMS spectra for compound 71

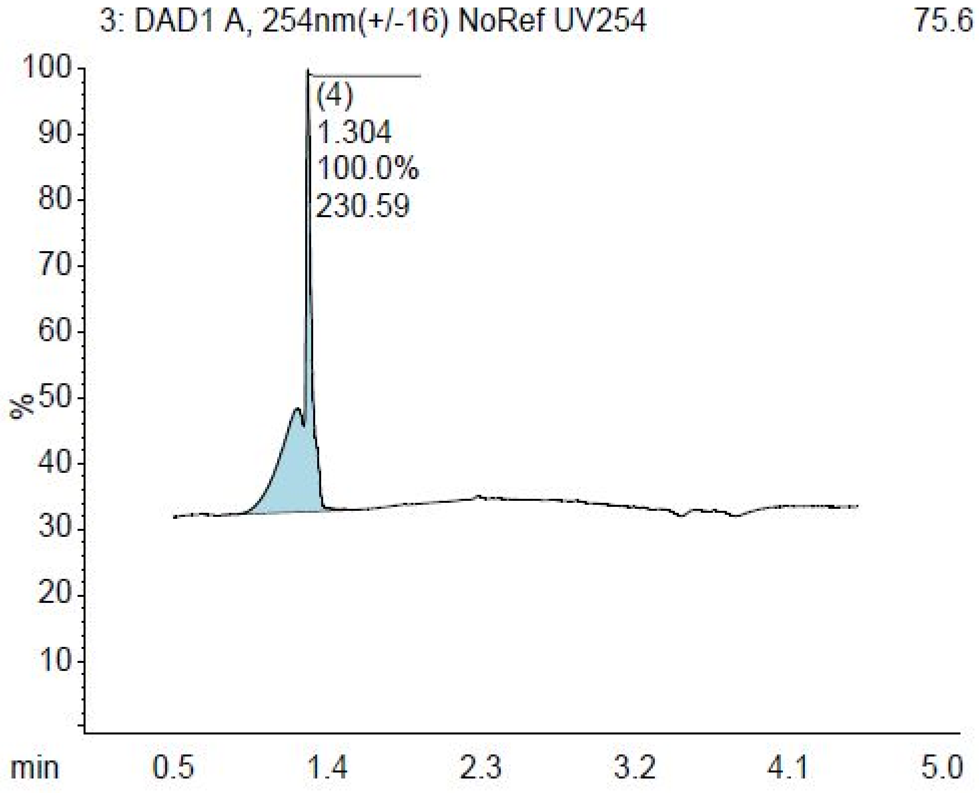

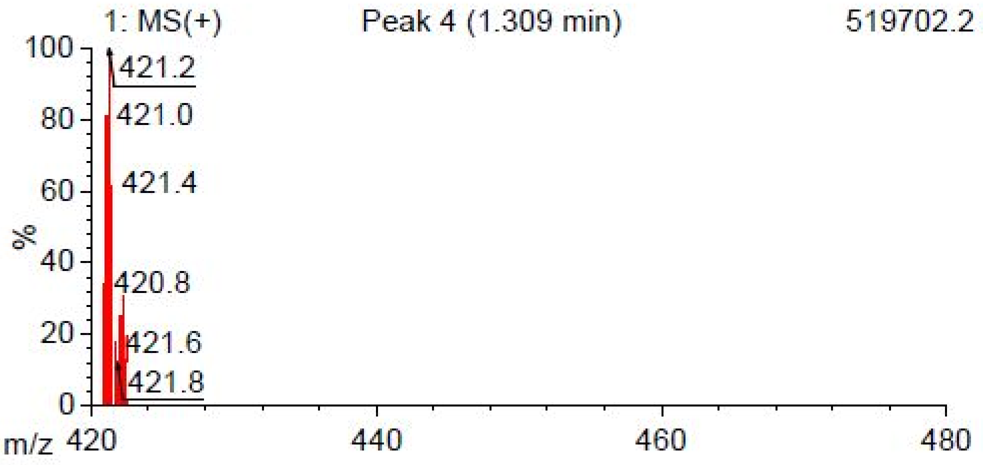

^{1}H spectra for compound 72

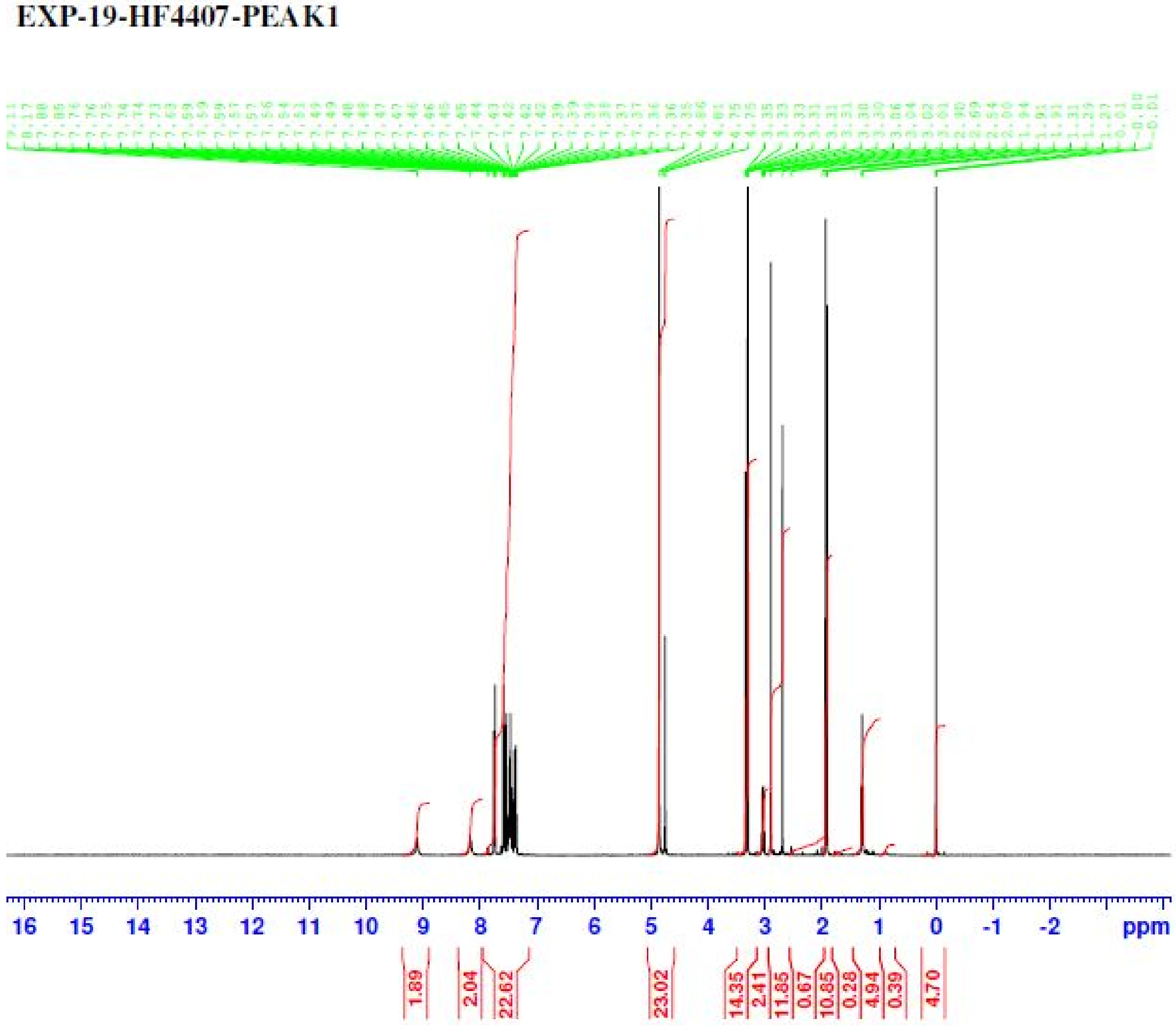

LCMS spectra for compound 72

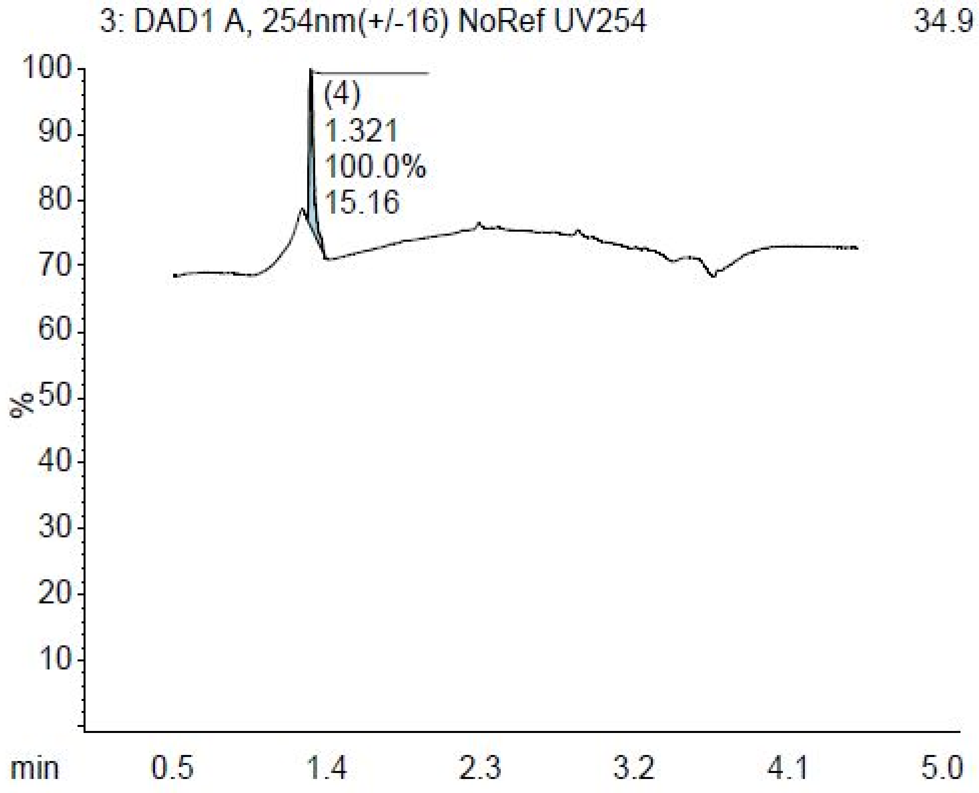

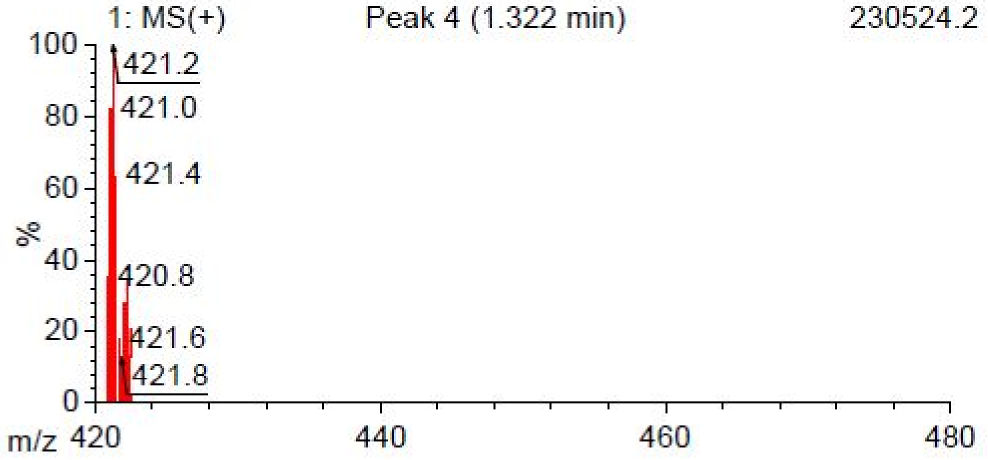

^{1}H spectra for compound 73

EXP- GY9067B

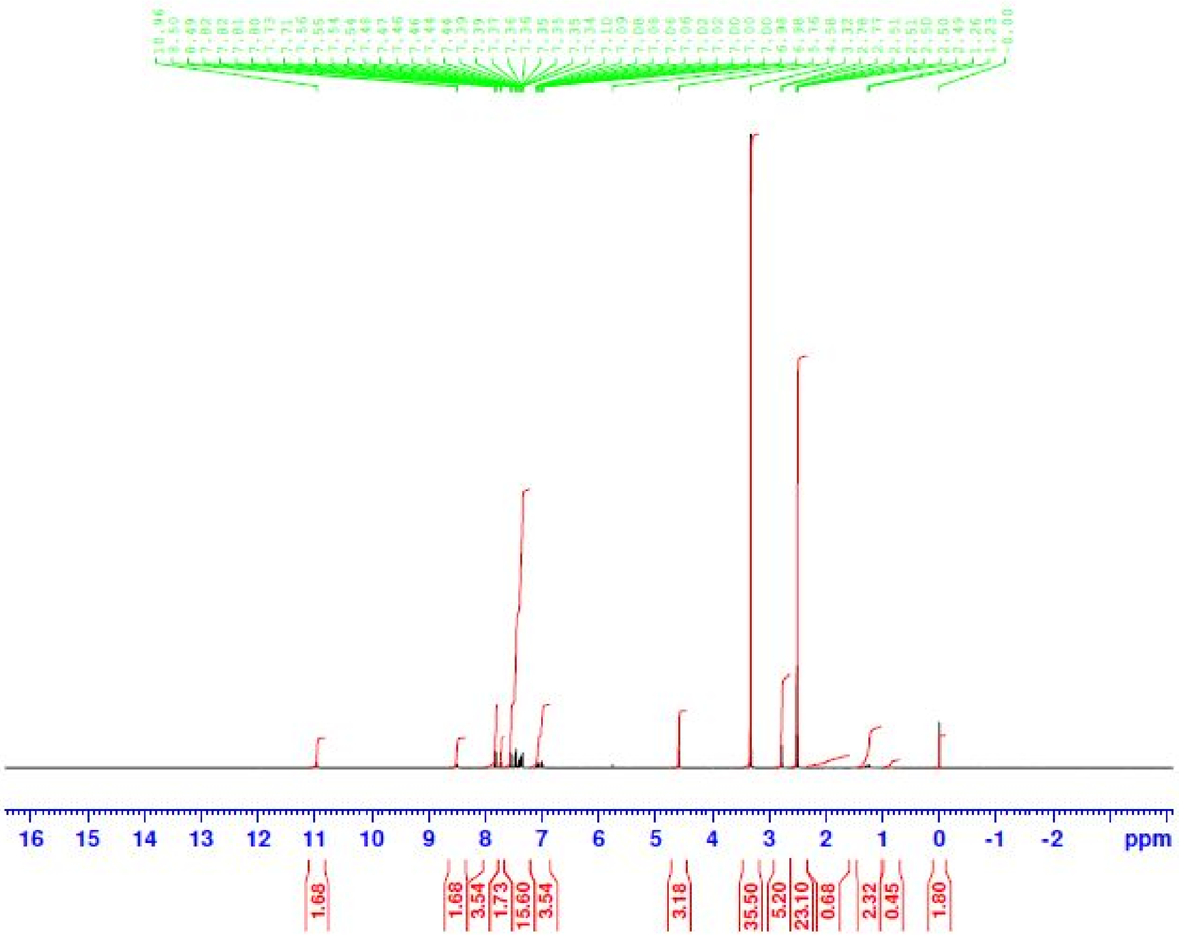

LCMS spectra for compound 73

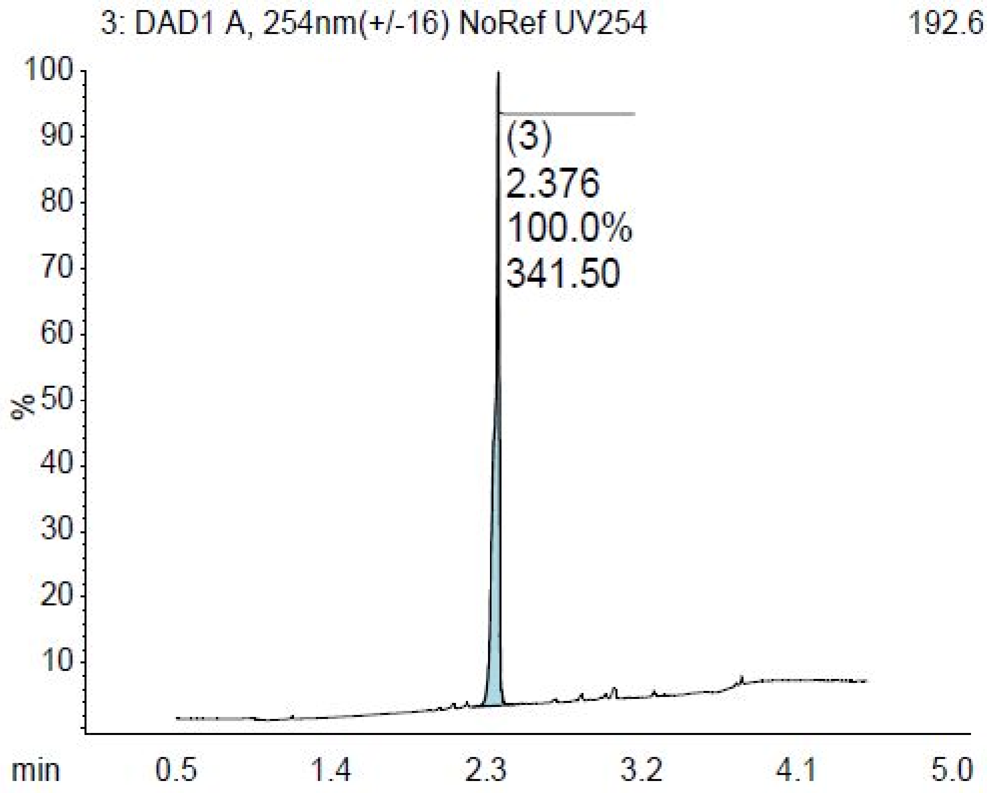

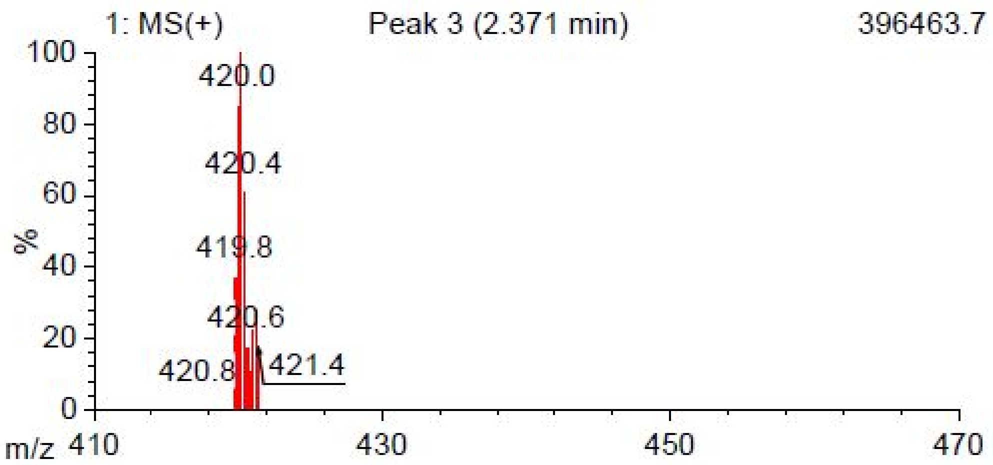

^{1}H spectra for compound 74

EXP- 19-GY9095C

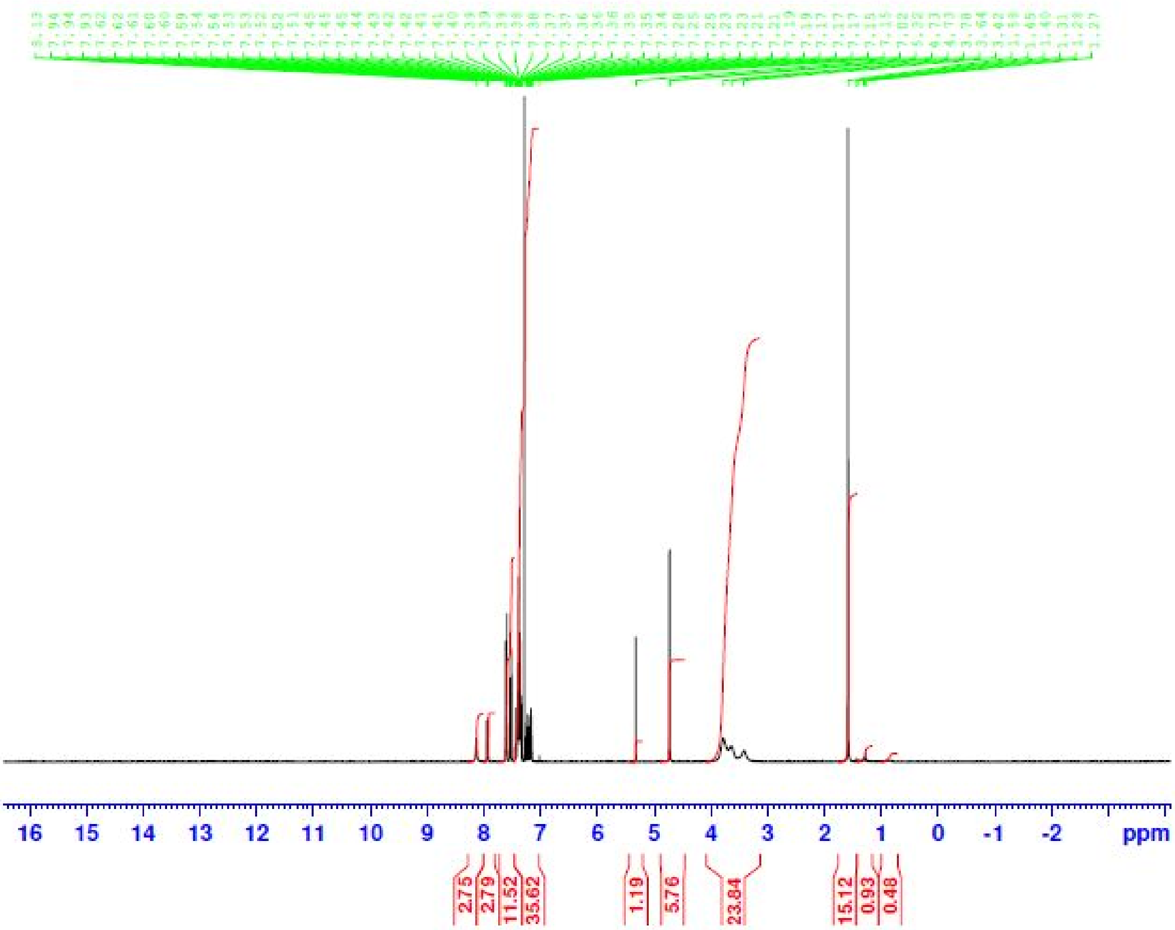

LCMS spectra for compound 74

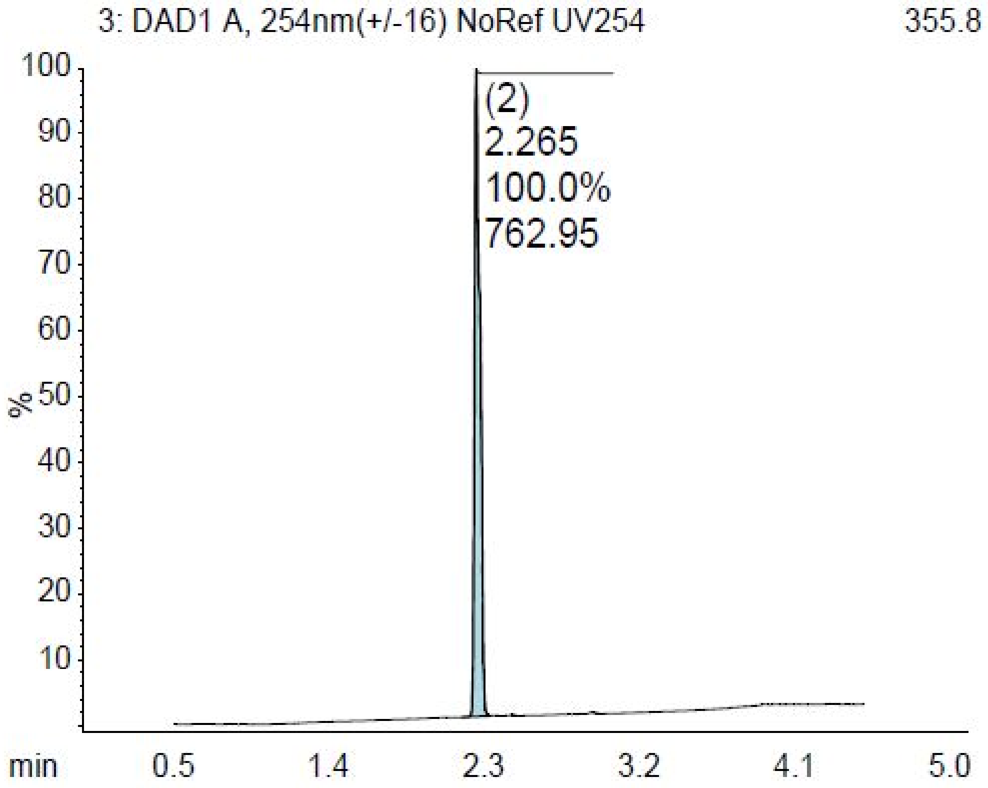

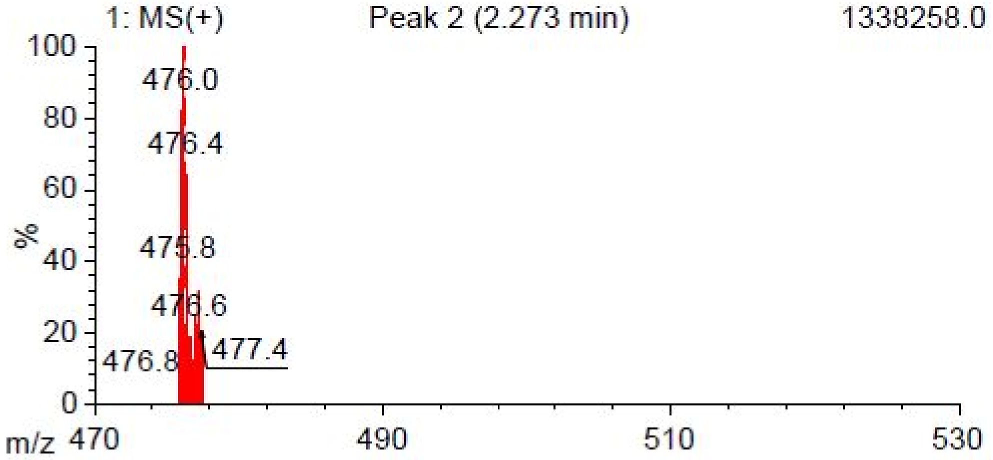

^{1}H NMR spectra for compound 75

EXP- 19-GY9096B

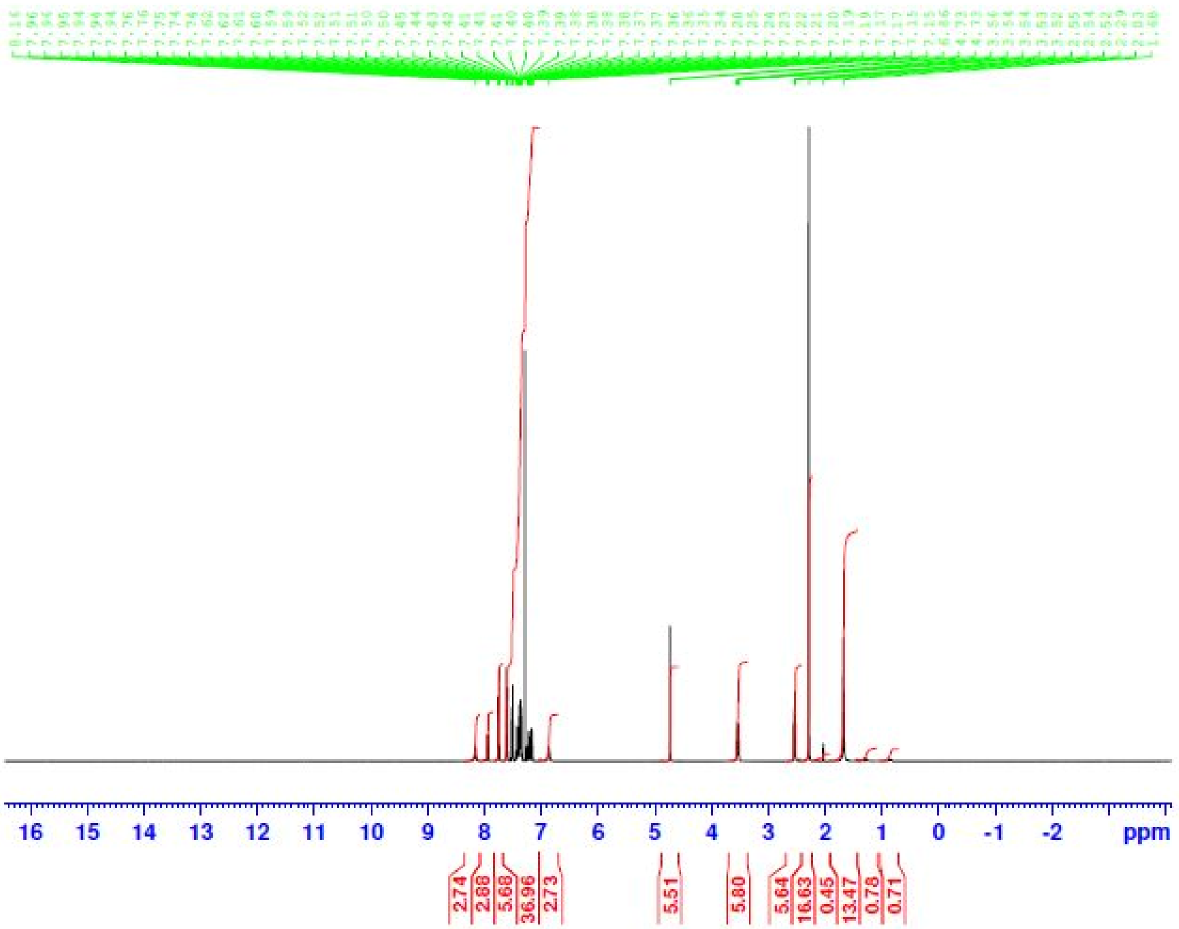

LC-MS spectra for compound 75

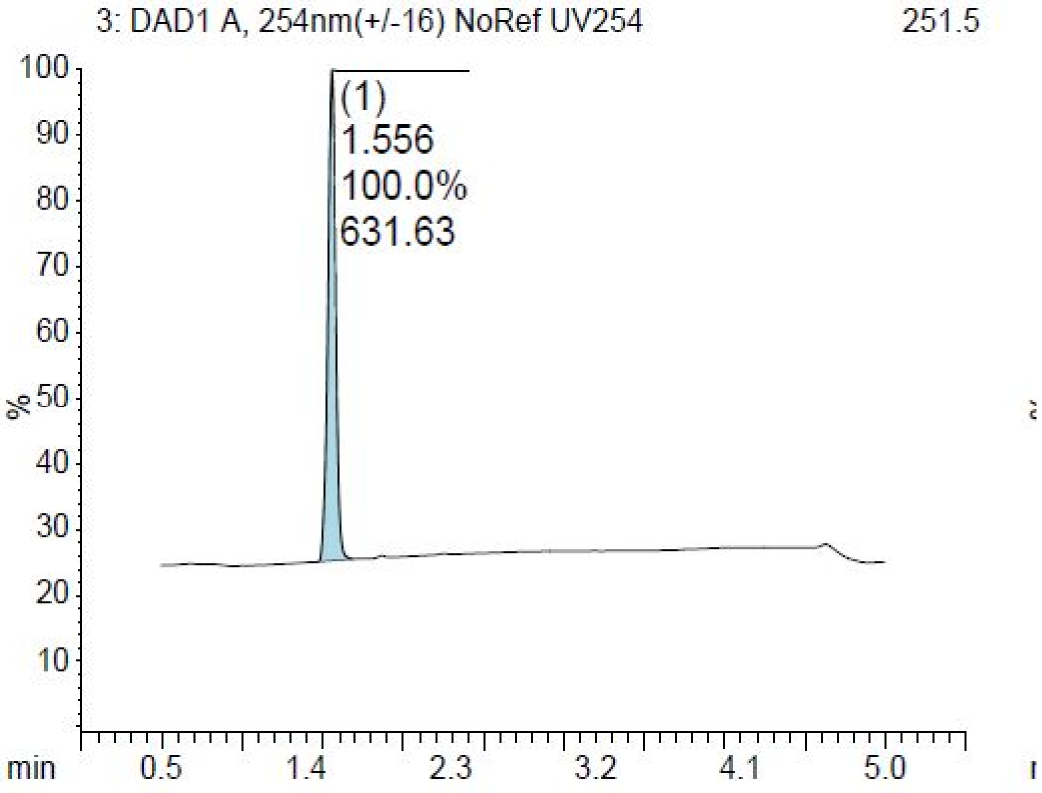

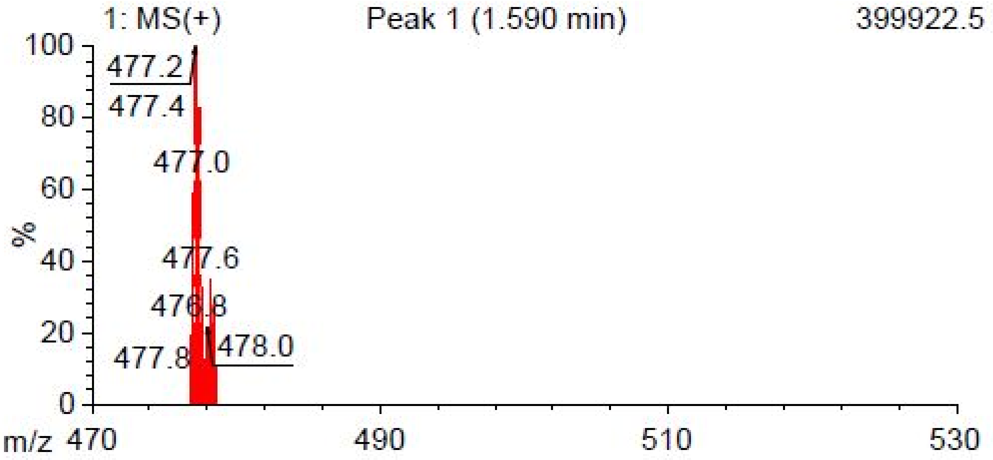

^1H NMR spectra for compound 76

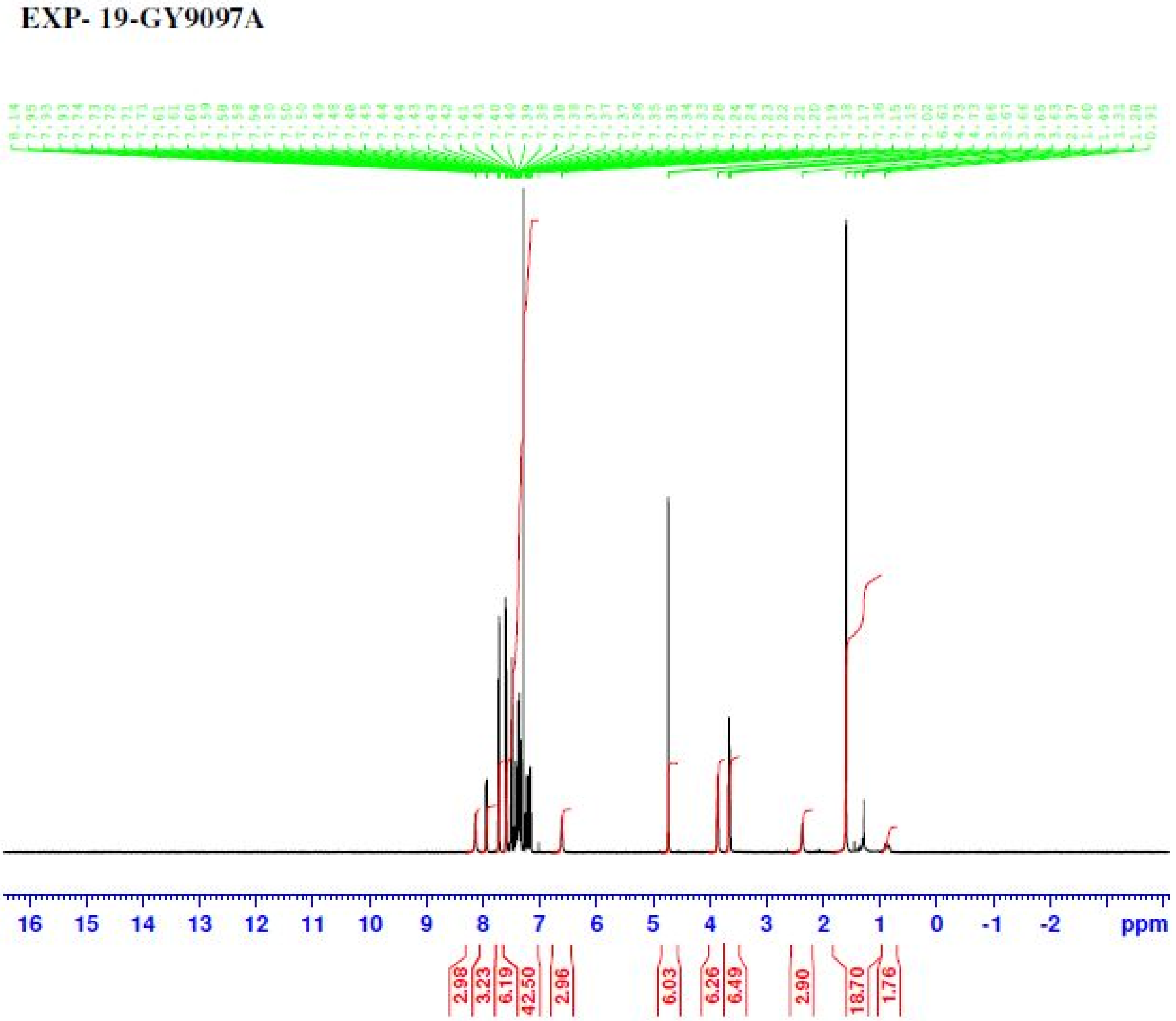

LC-MS spectra for compound 76

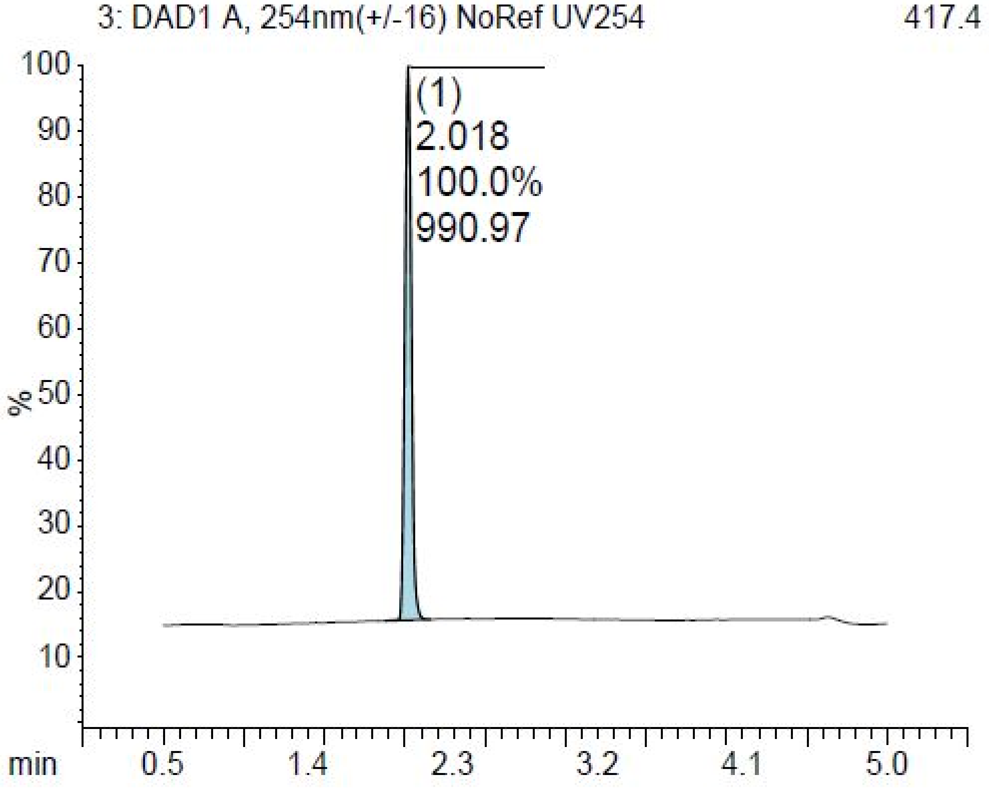

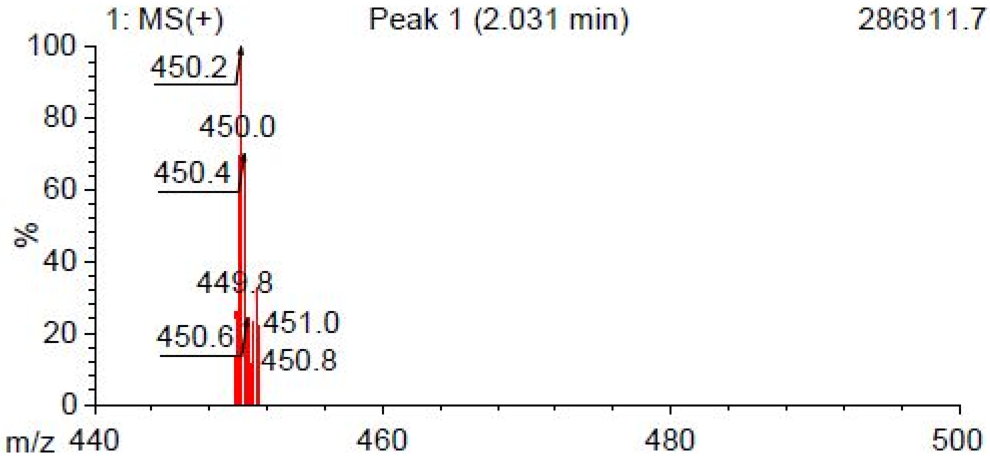

^{1}H NMR spectra for compound 77

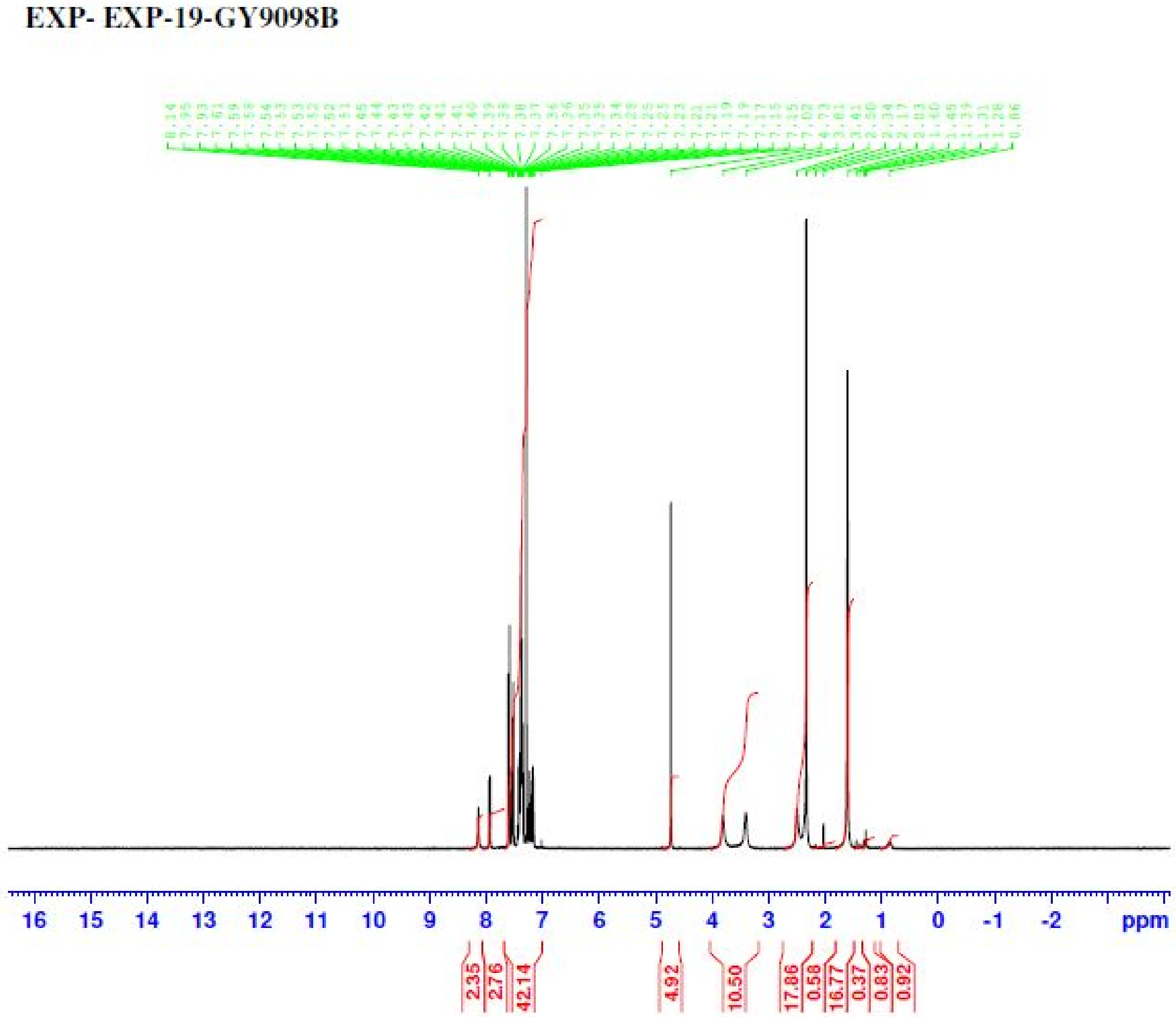

LC-MS spectra for compound 77

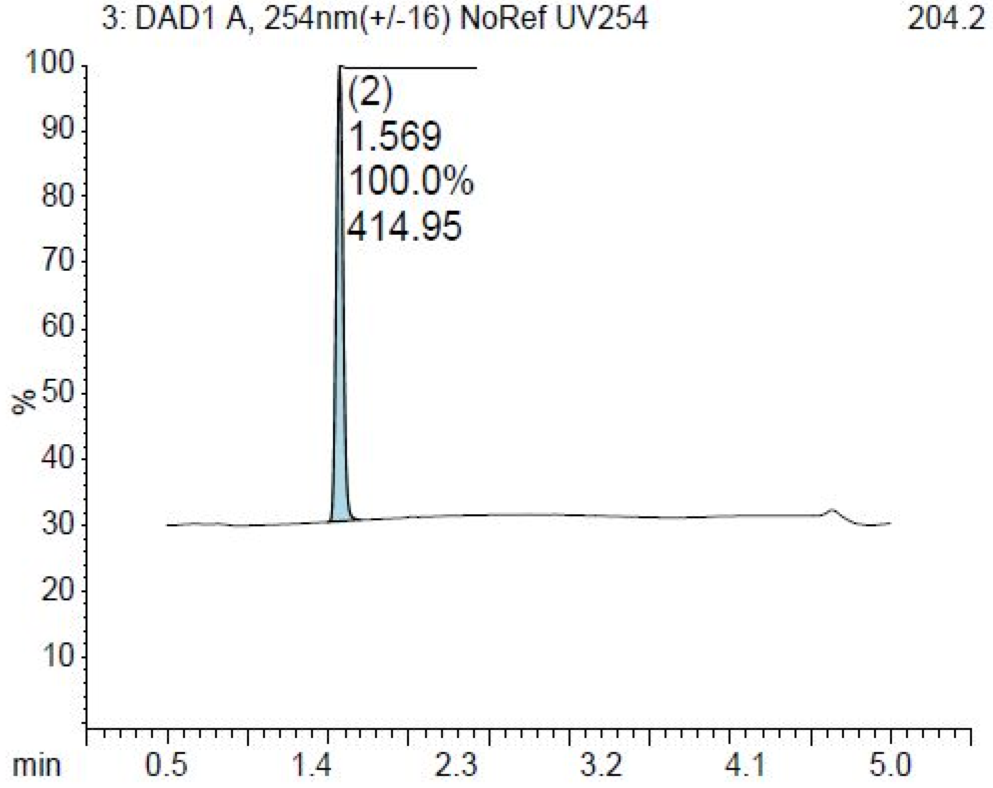

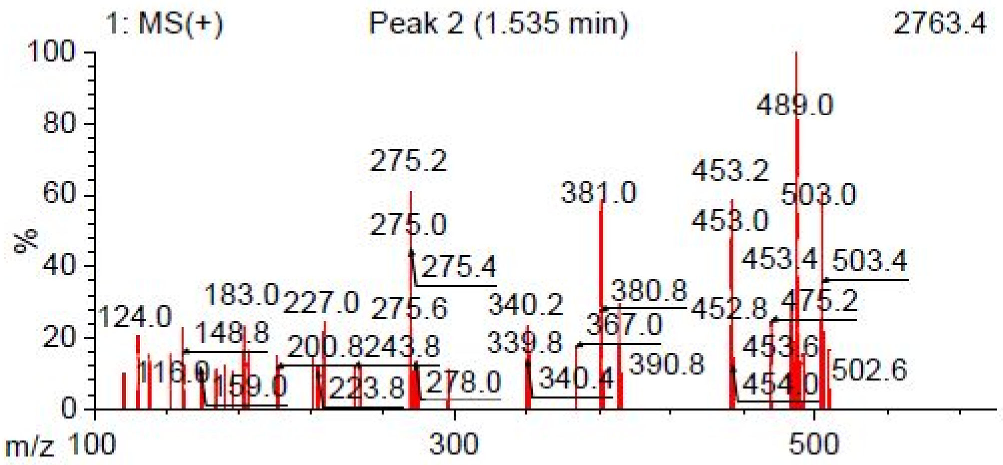

^{1}H NMR spectra for compound 78

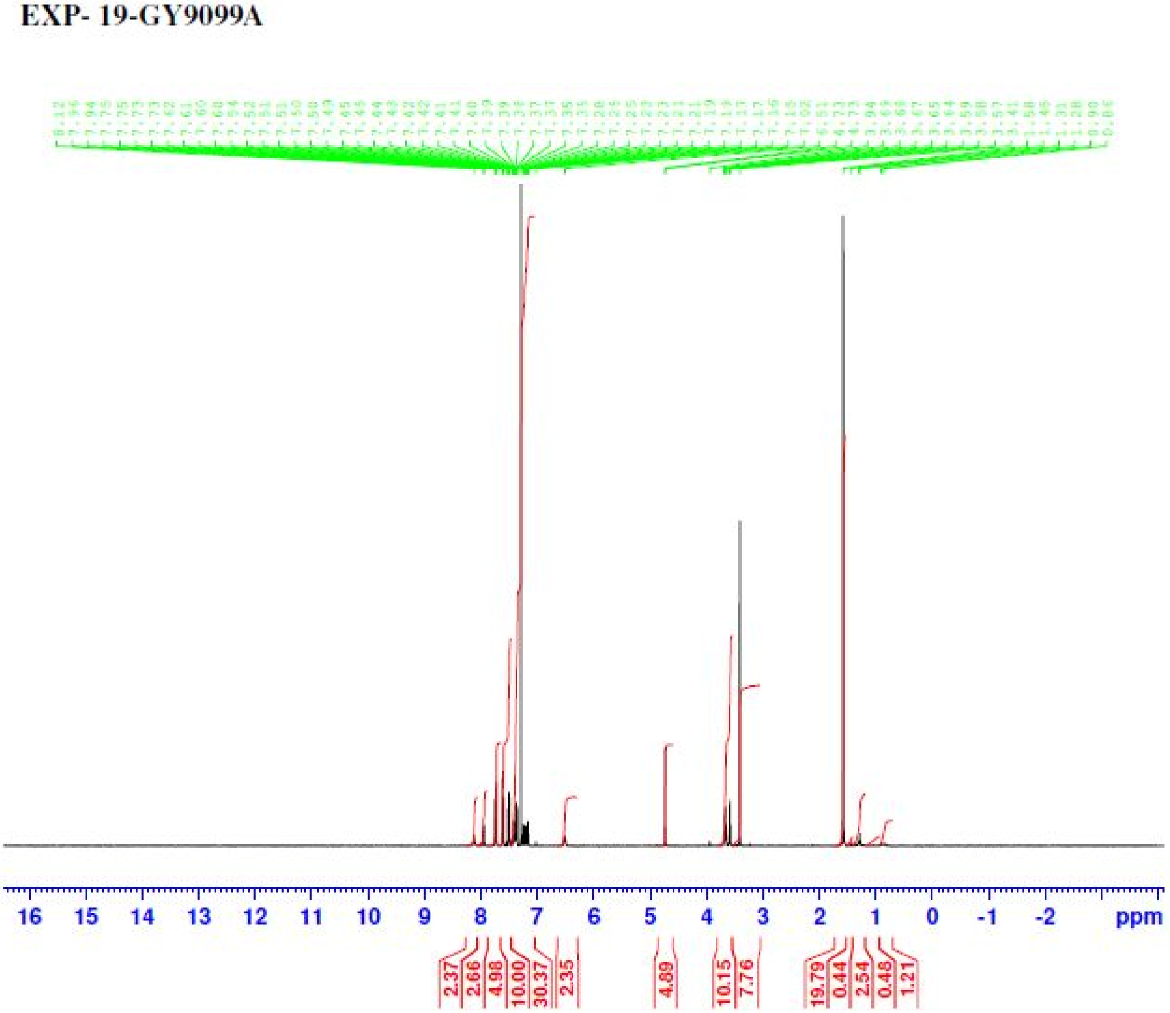

LCMS spectra for compound 78

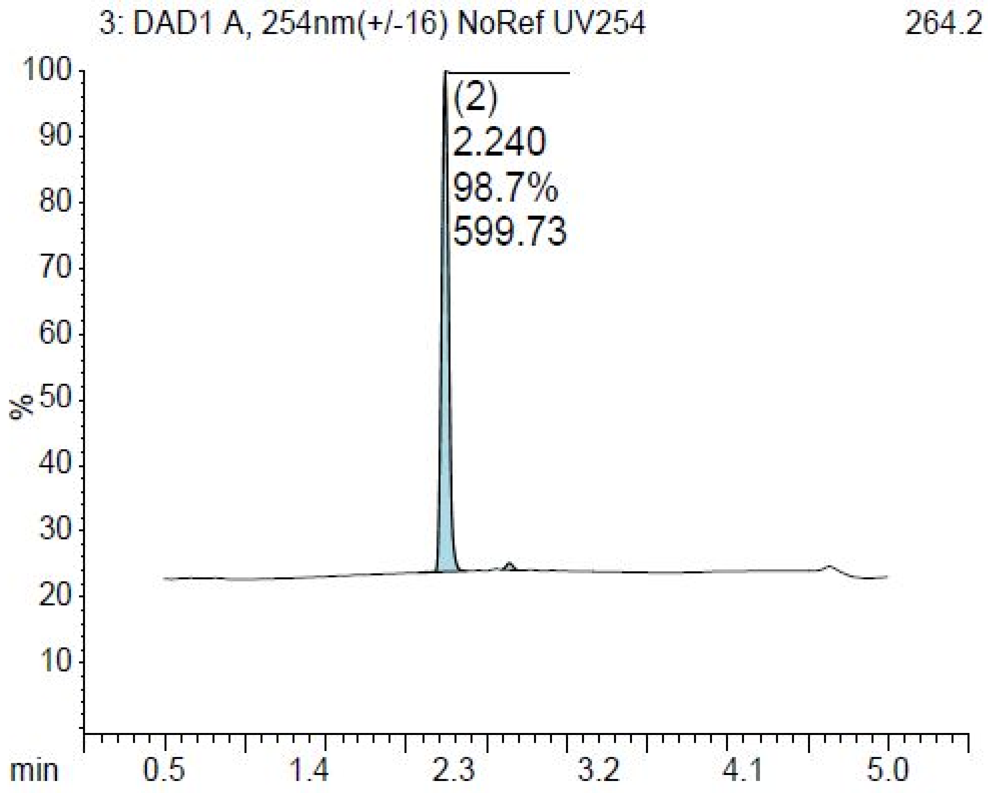

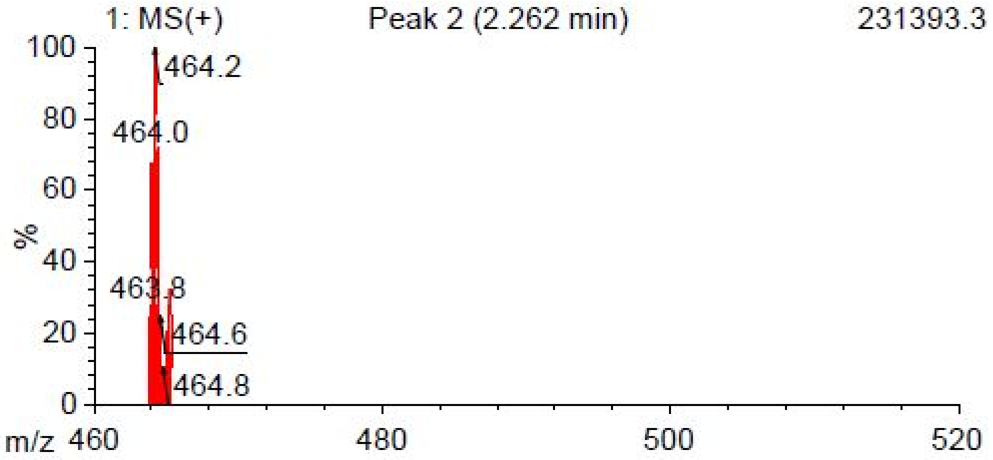

^{1}H NMR spectra for compound 79

EXP- 19-HE9400B

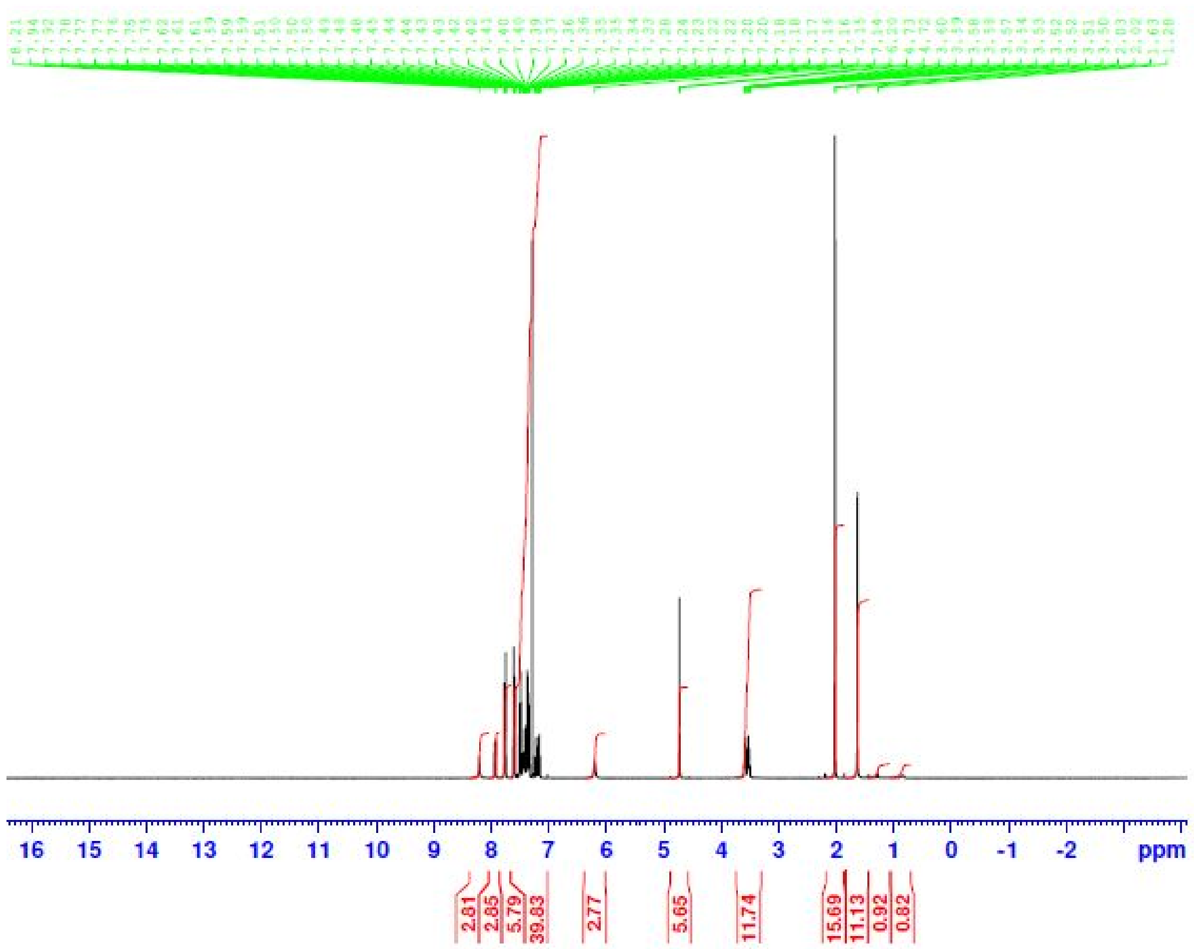

LCMS spectra for compound 79

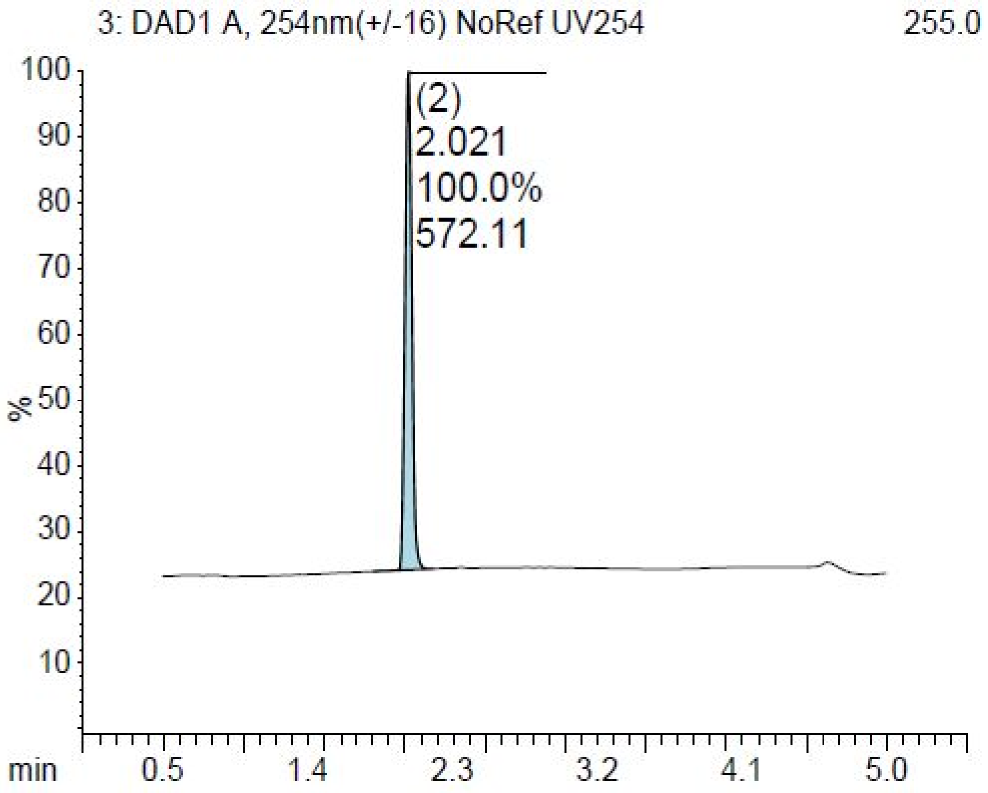

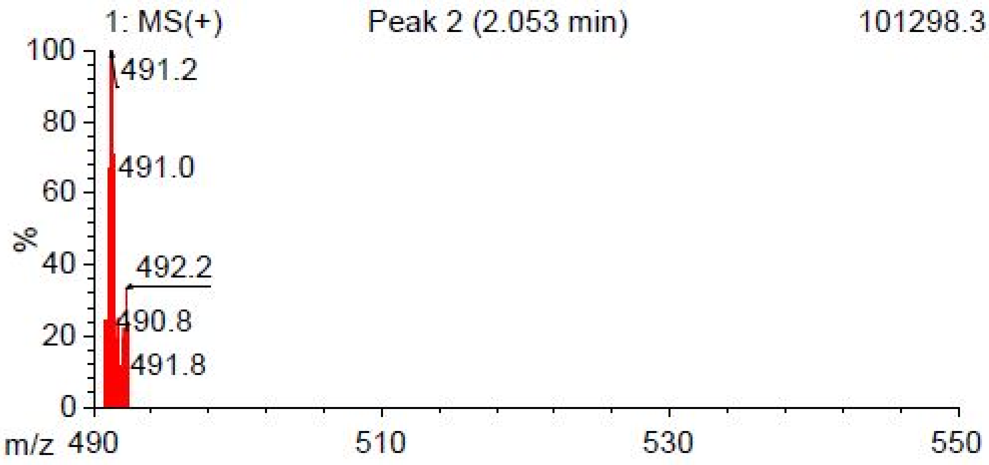

^{1}H NMR spectra for compound 80

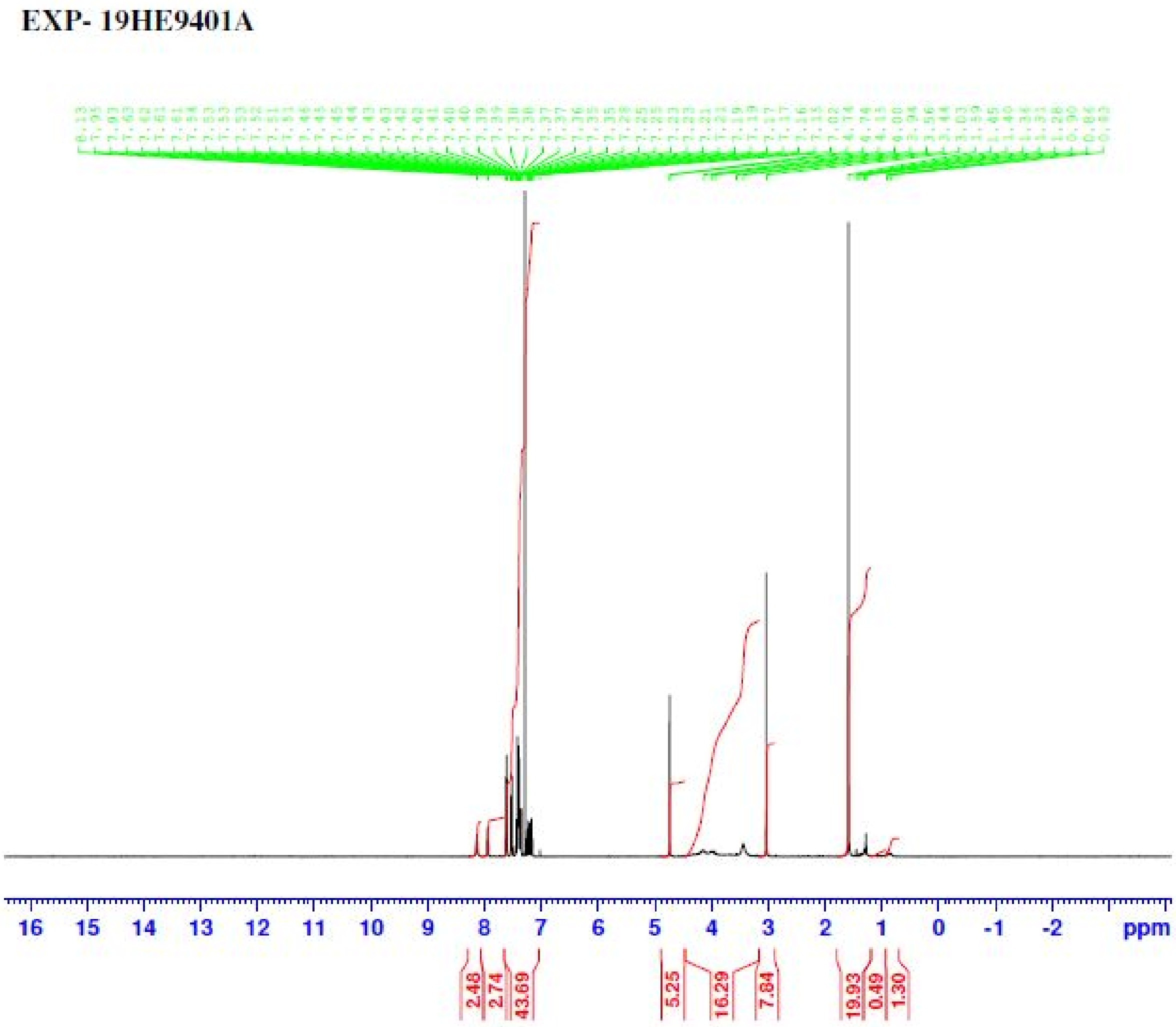

LCMS spectra for compound 80

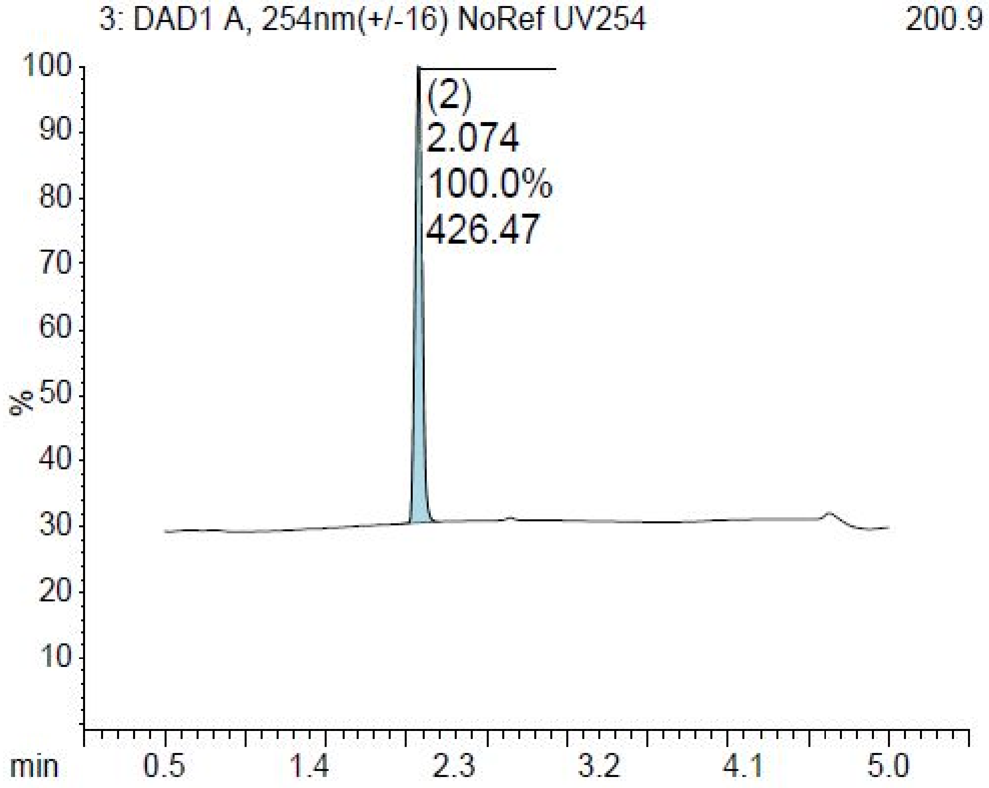

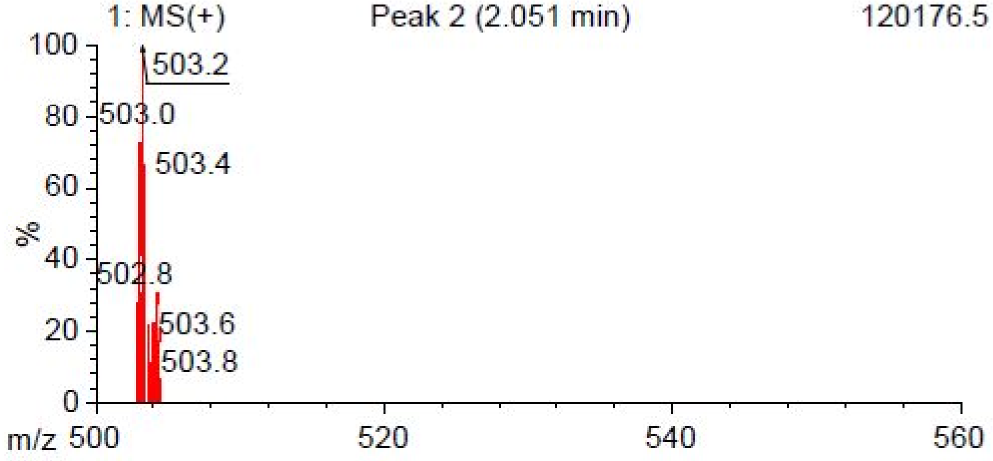

^{1}H NMR spectra for compound 81

EXP- 19-HE9402C

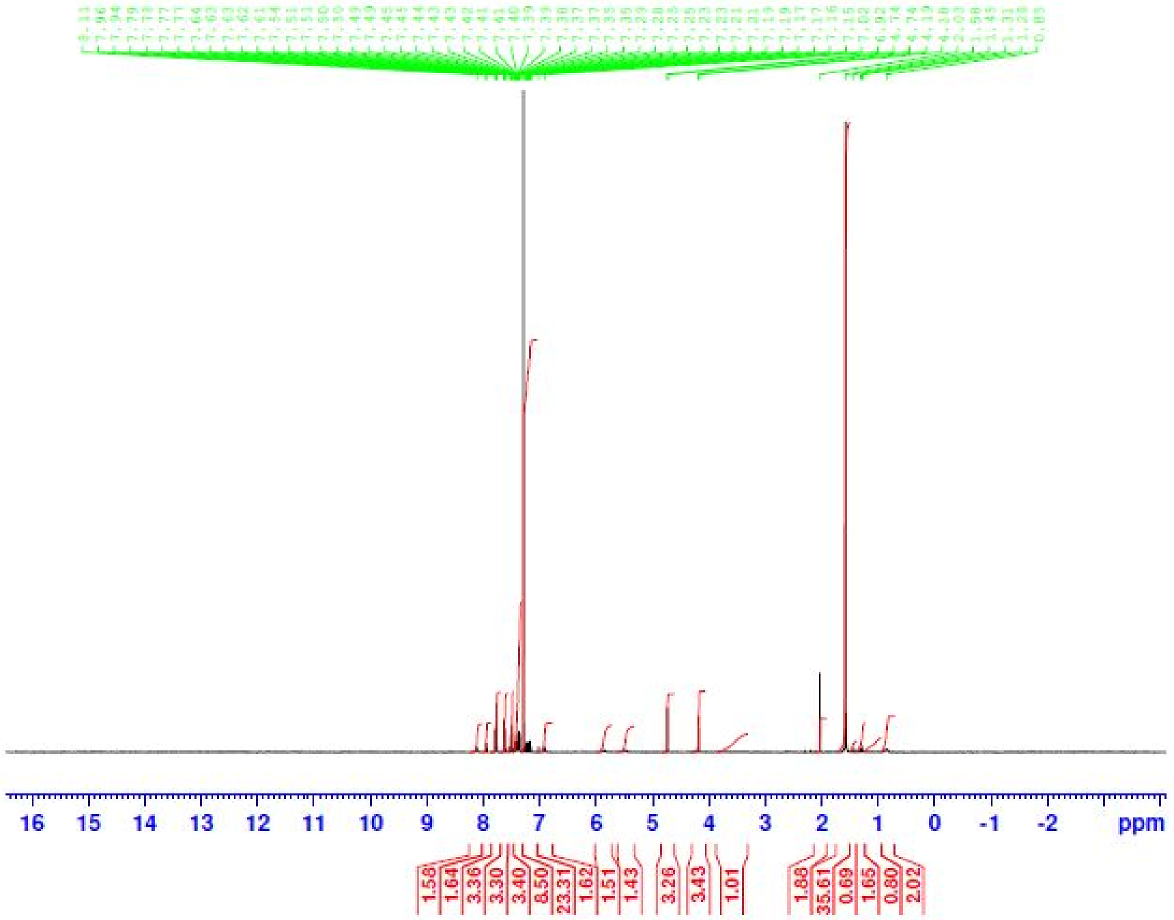

LCMS spectra for compound 81

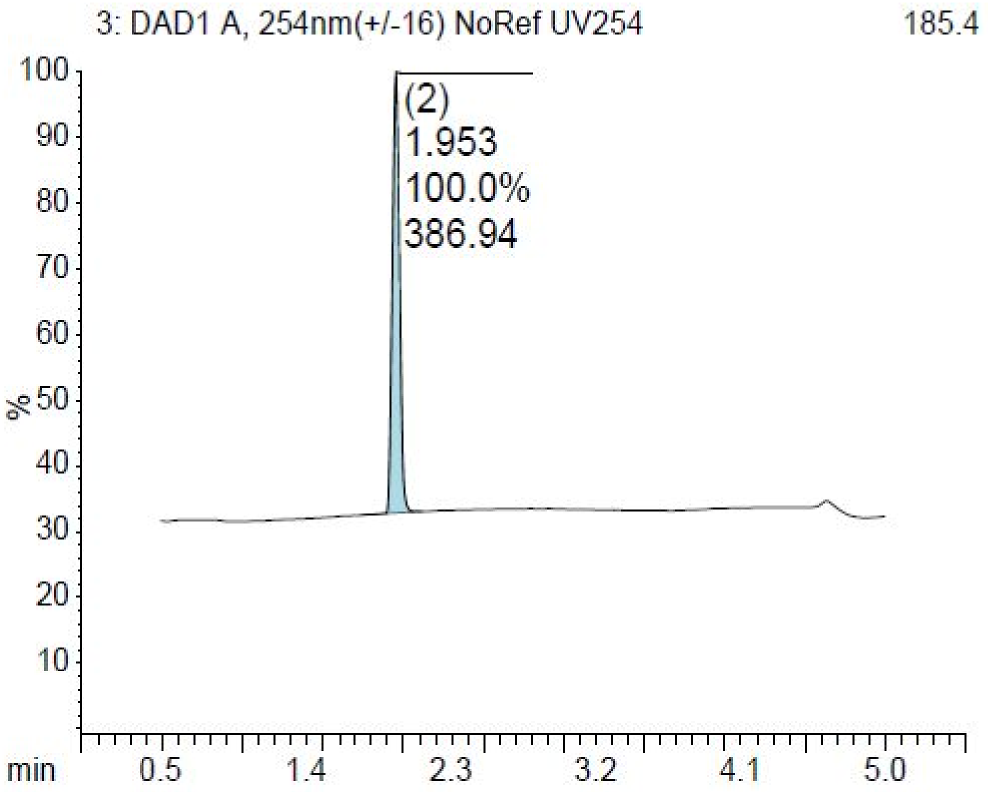

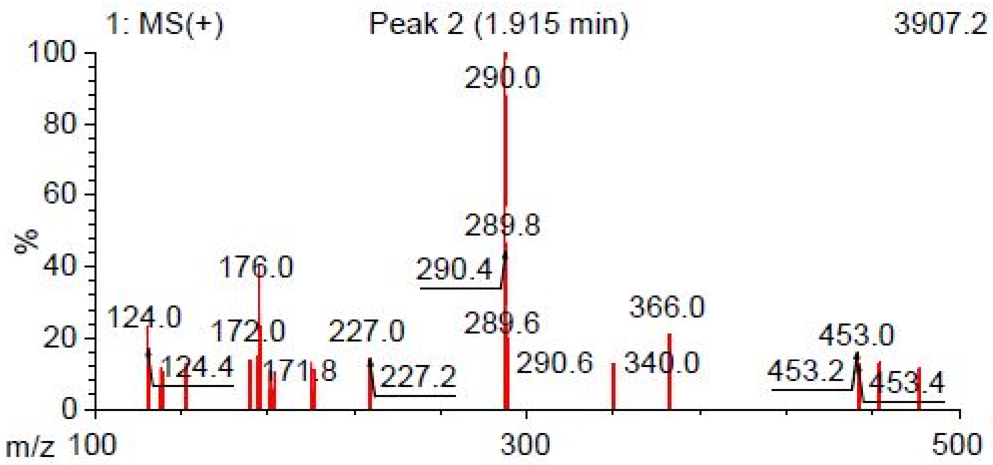

^{1}H NMR spectra for compound 82

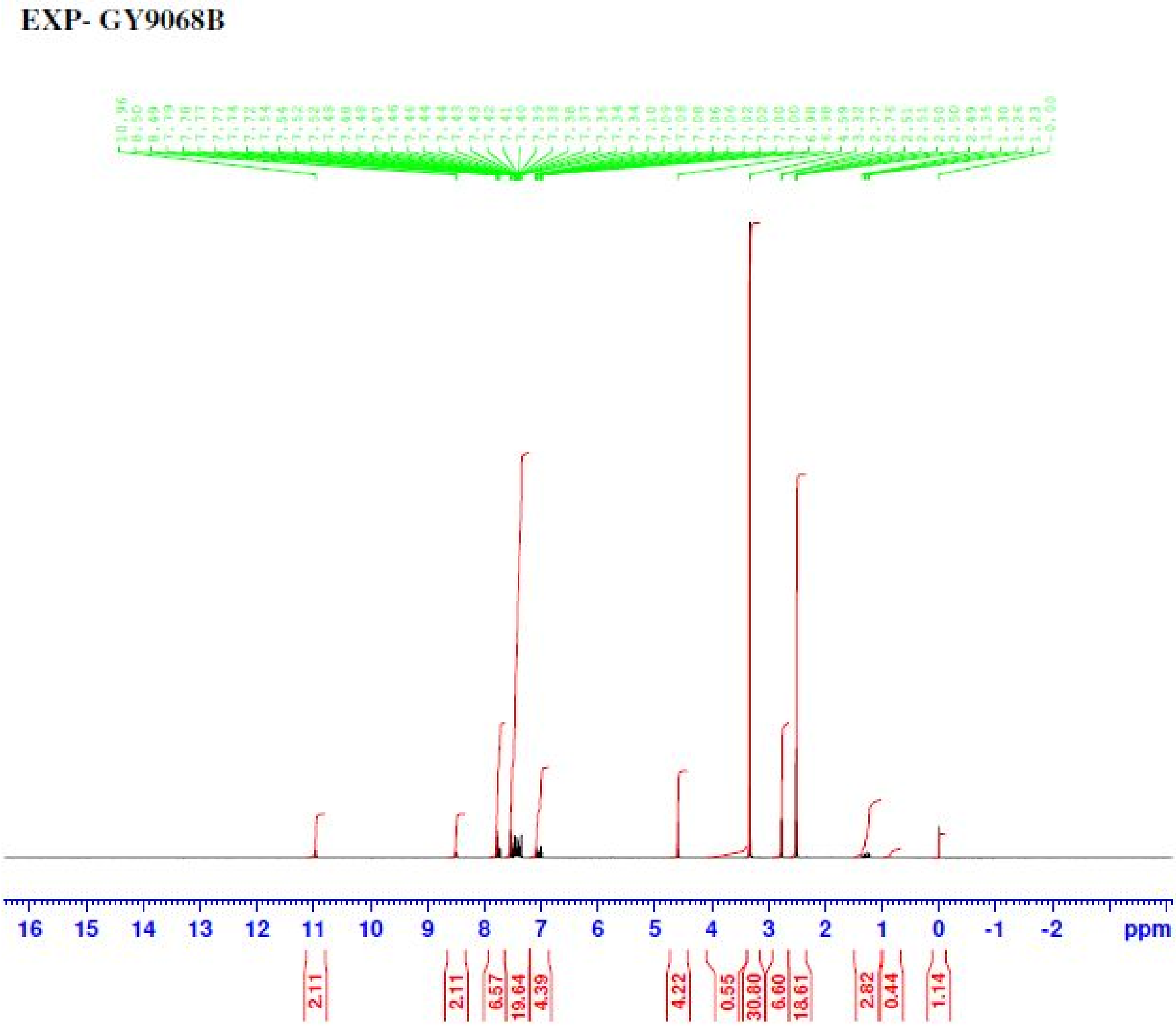

LCMS spectra for compound 82

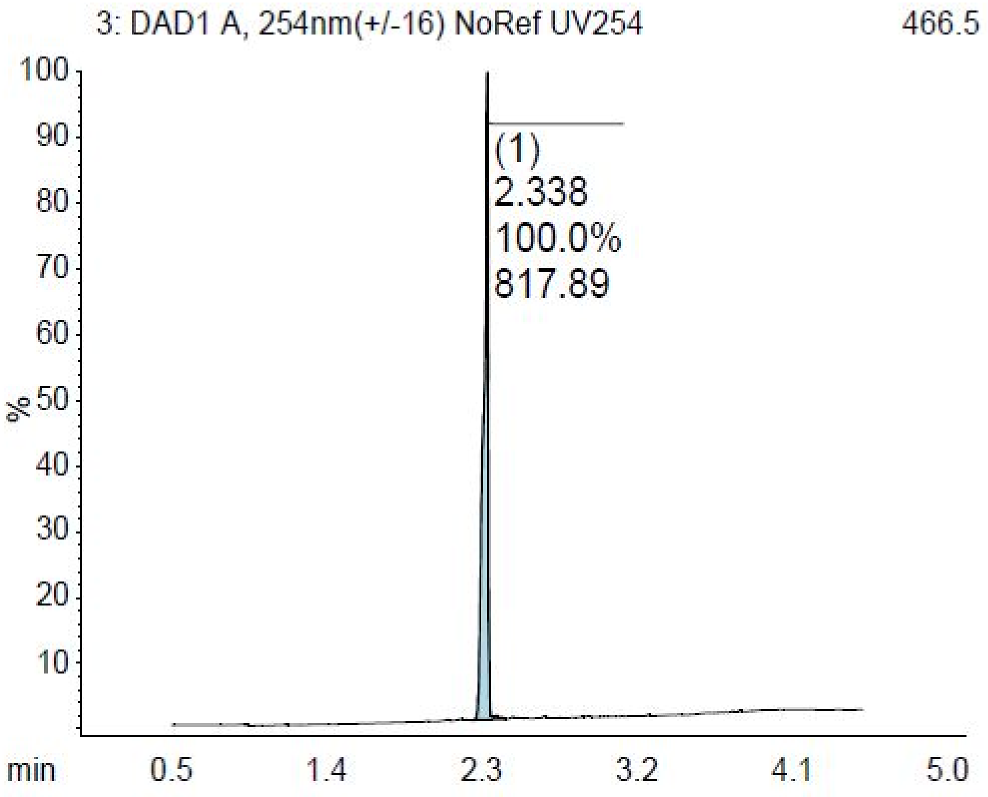

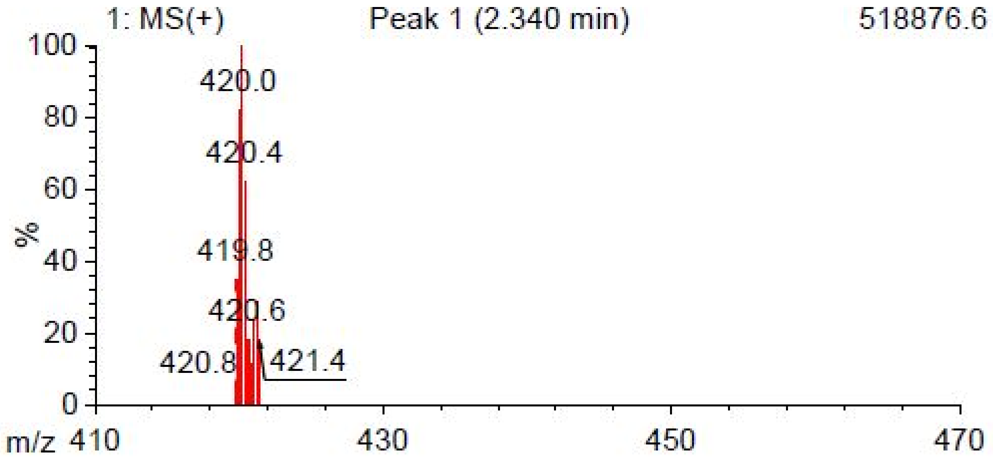

^{1}H NMR spectra for compound 83

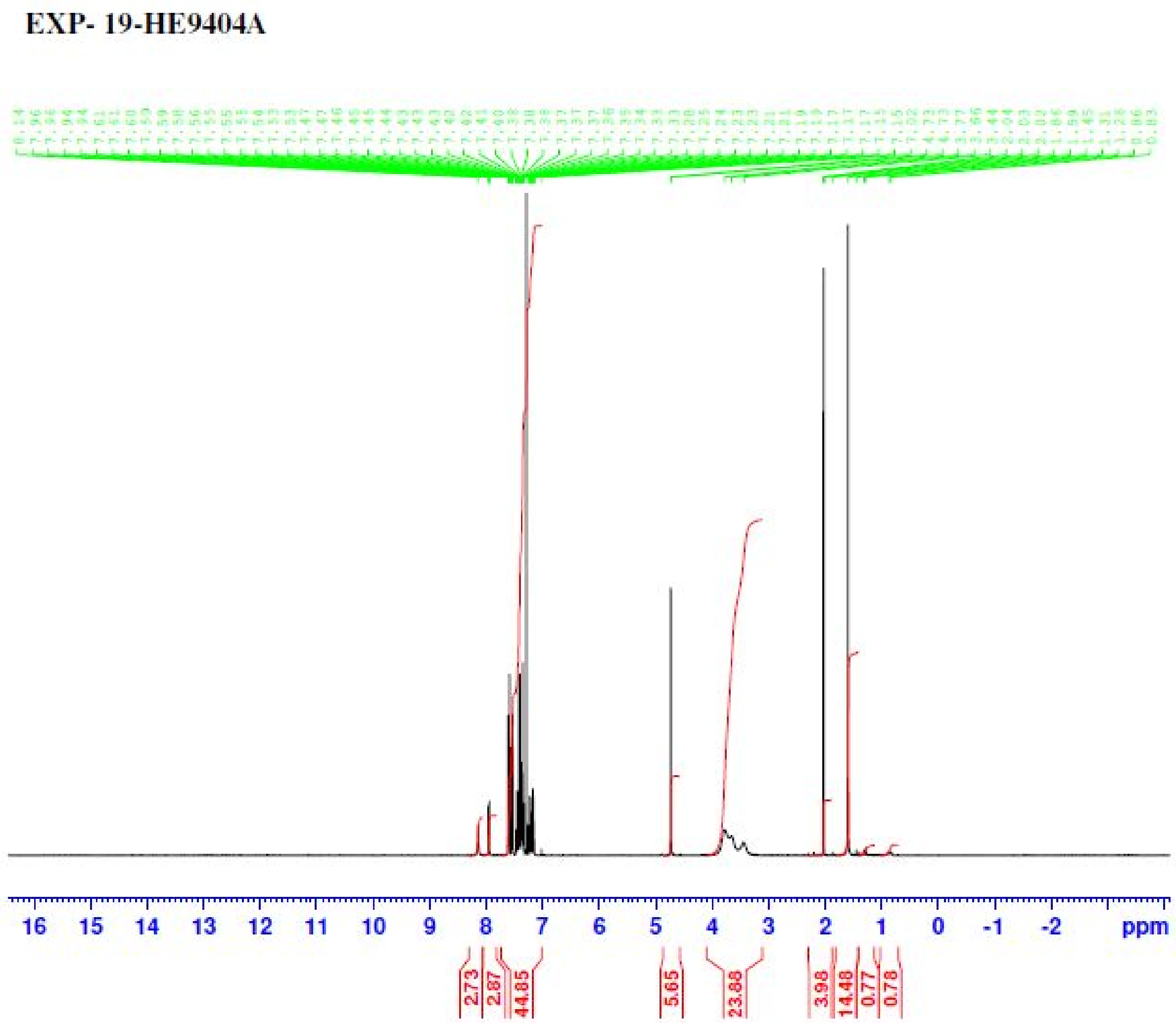

LCMS spectra for compound 83

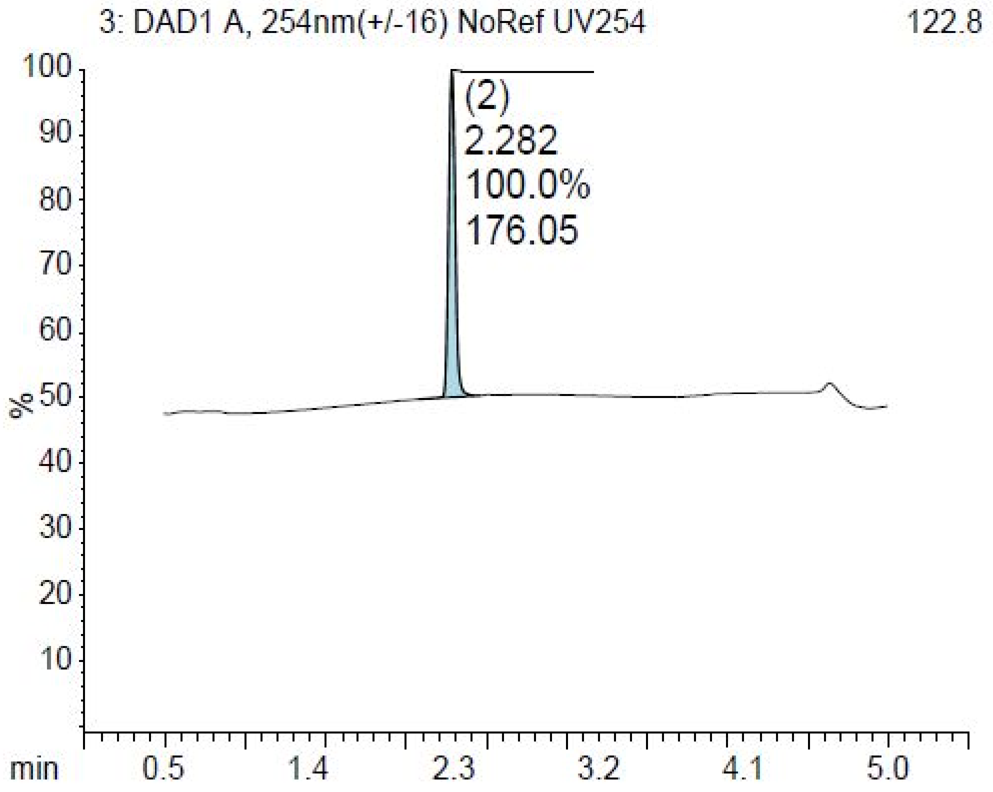

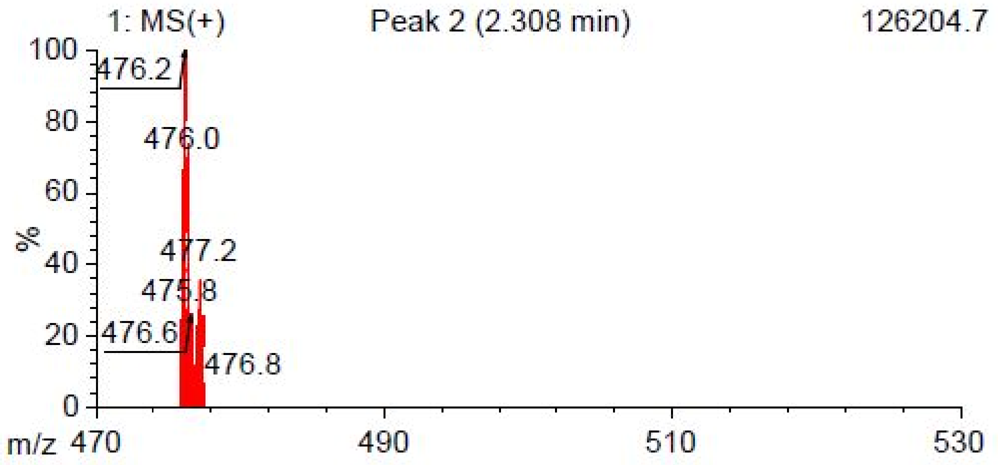

^{1}H NMR spectra for compound 84

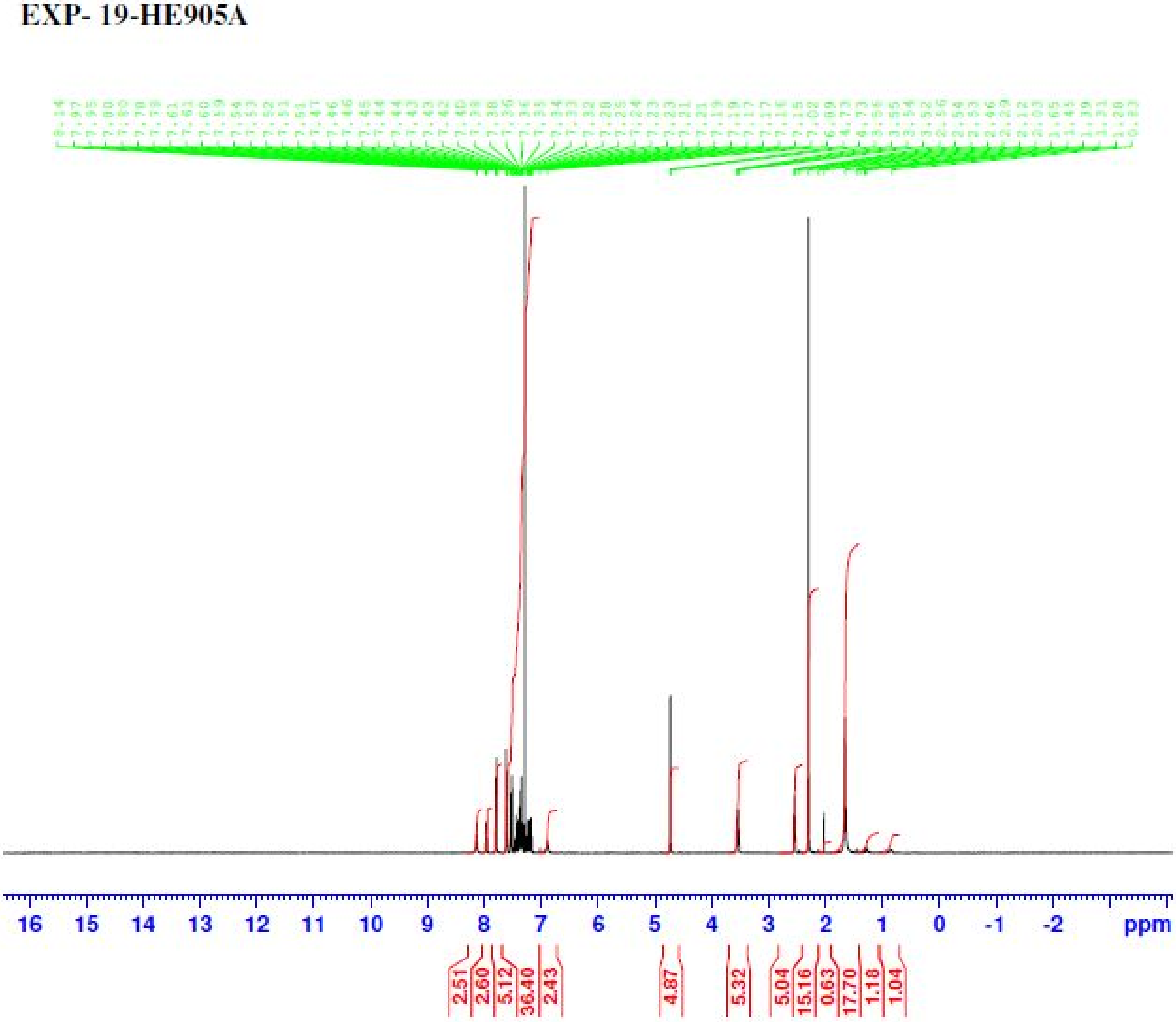

LCMS spectra for compound 84

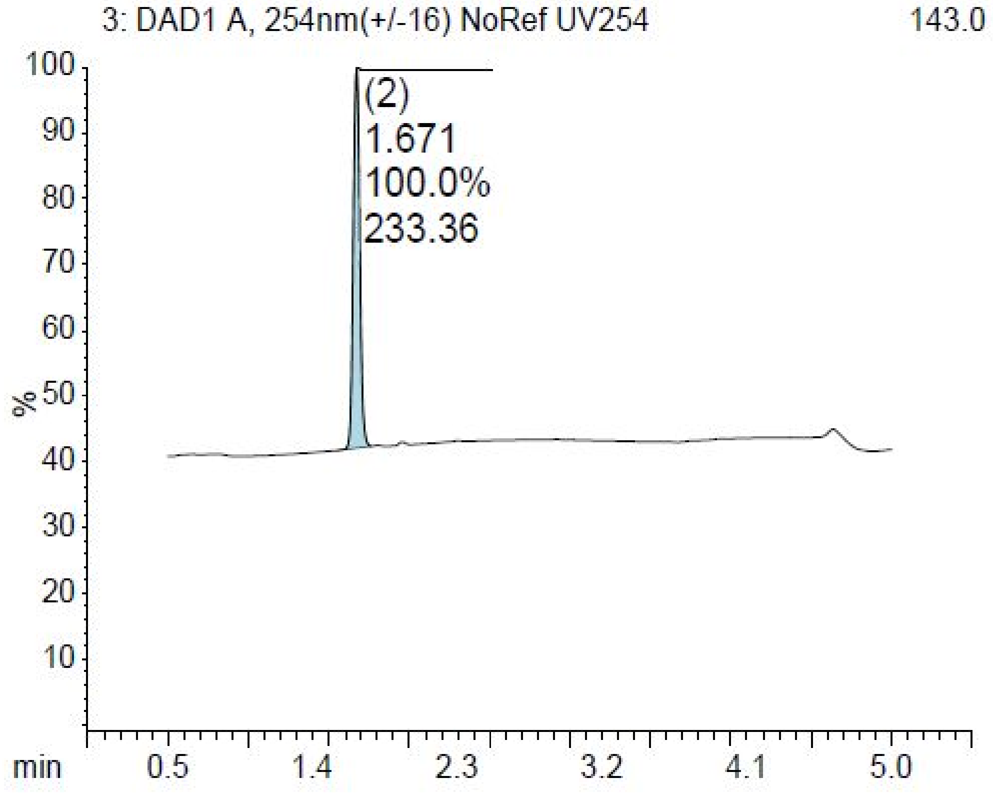

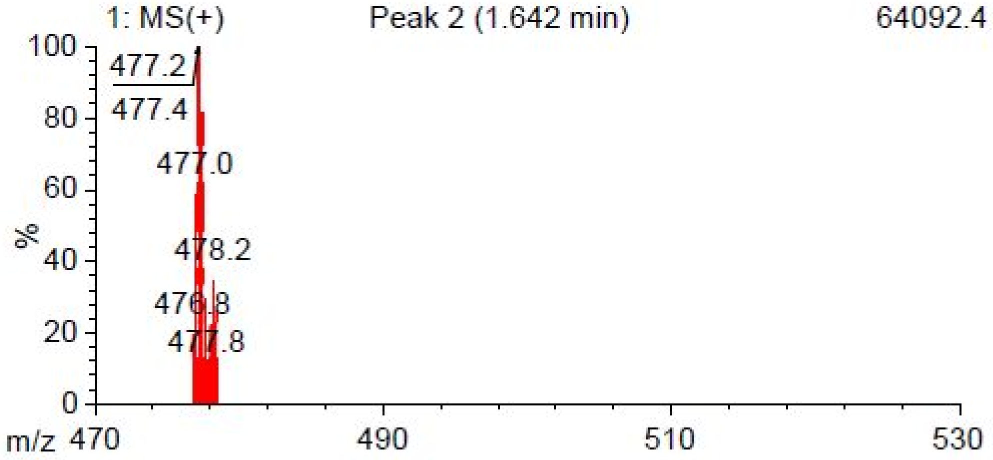

^{1}H NMR spectra for compound 85

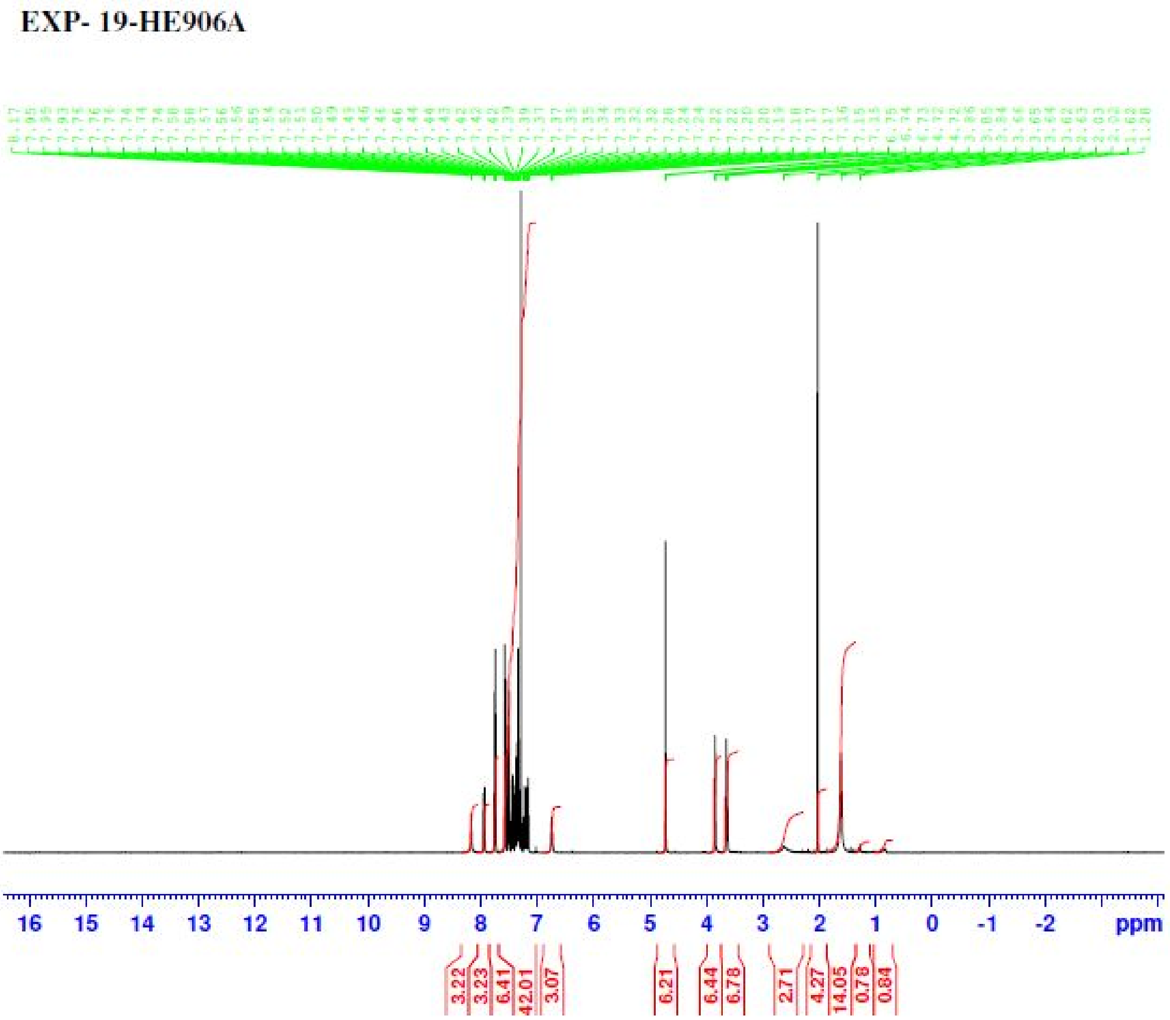

LCMS spectra for compound 85

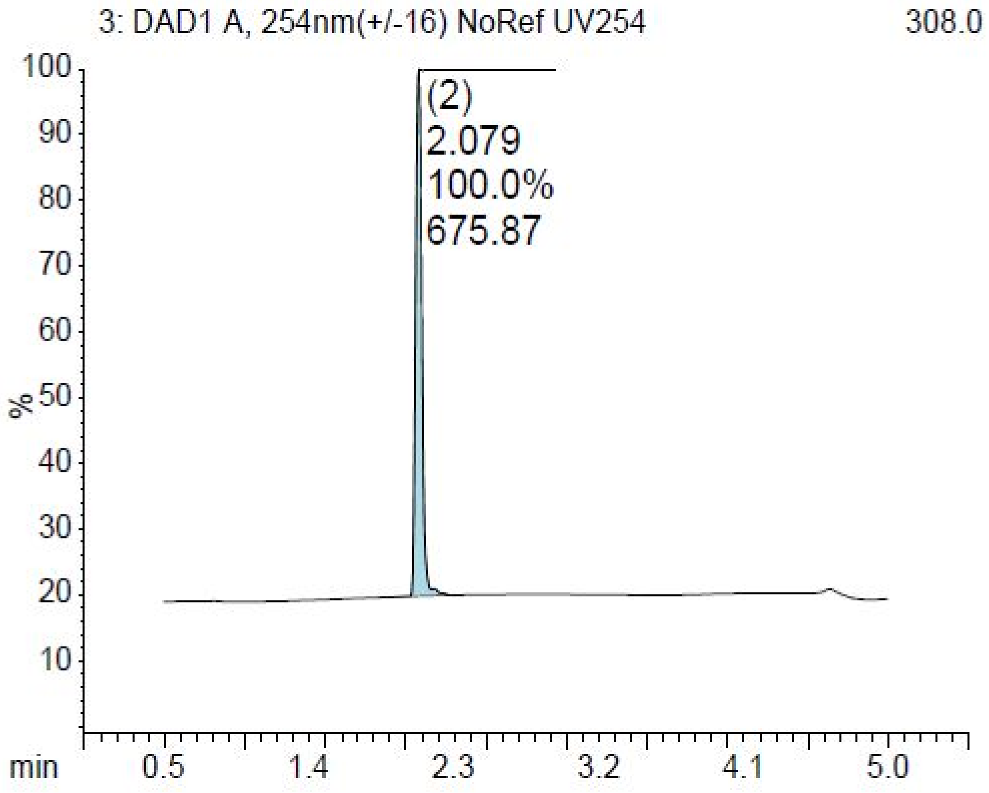

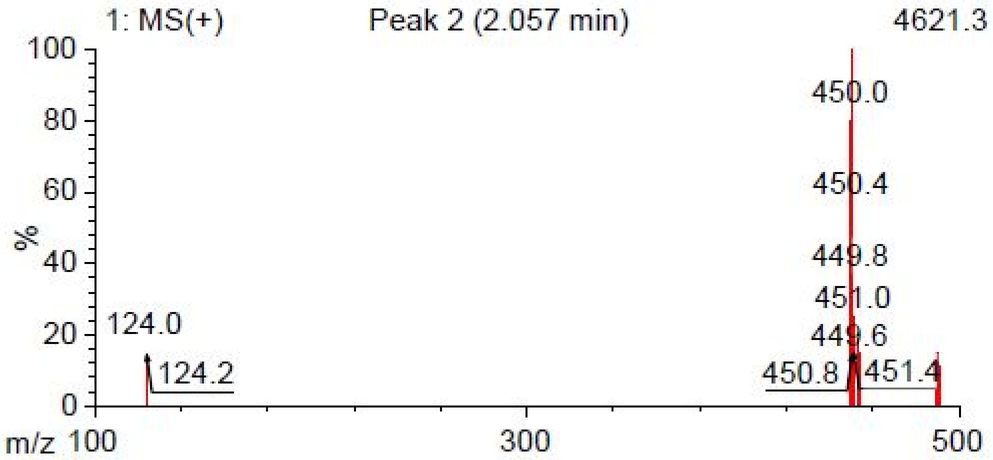

^{1}H NMR spectra for compound 86

EXP- 19-HE9407A

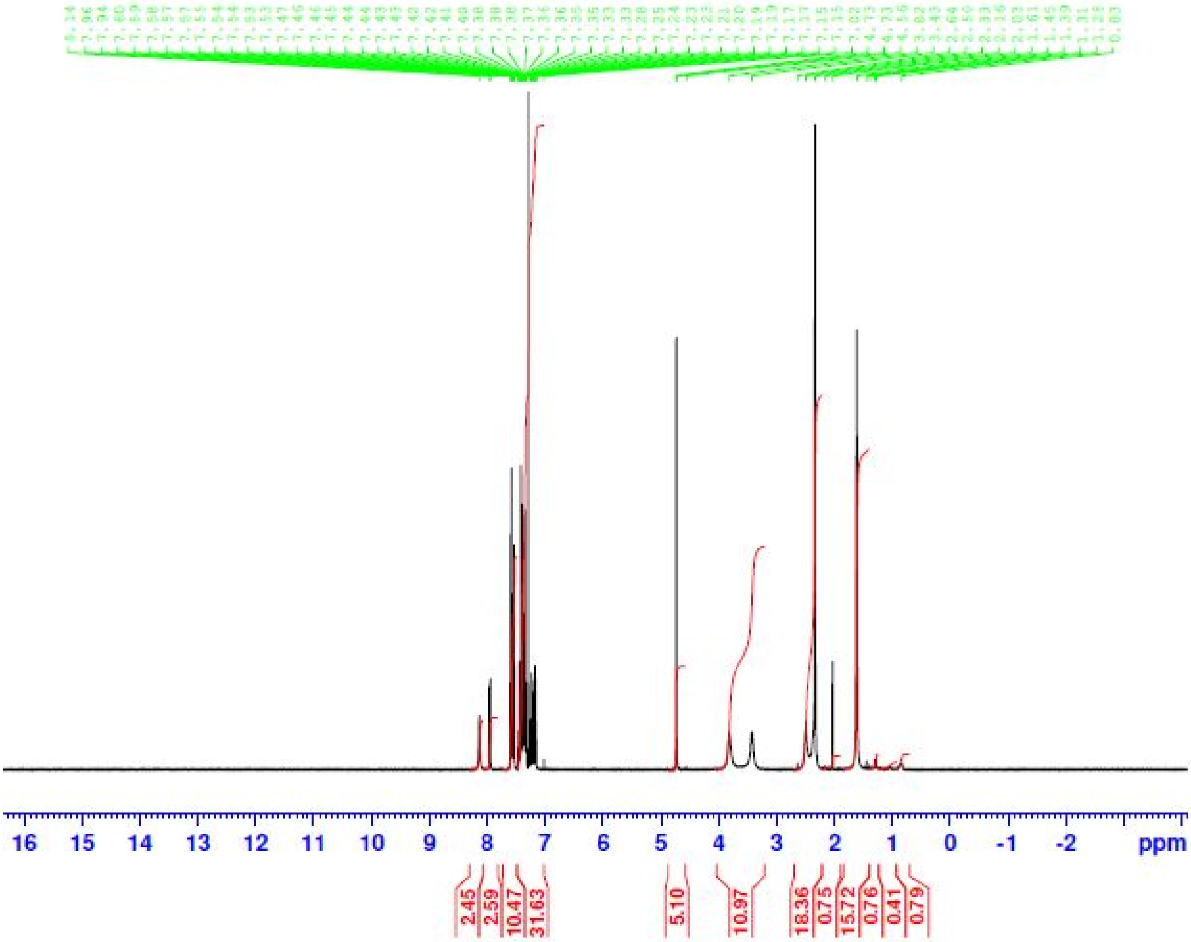

LCMS spectra for compound 86

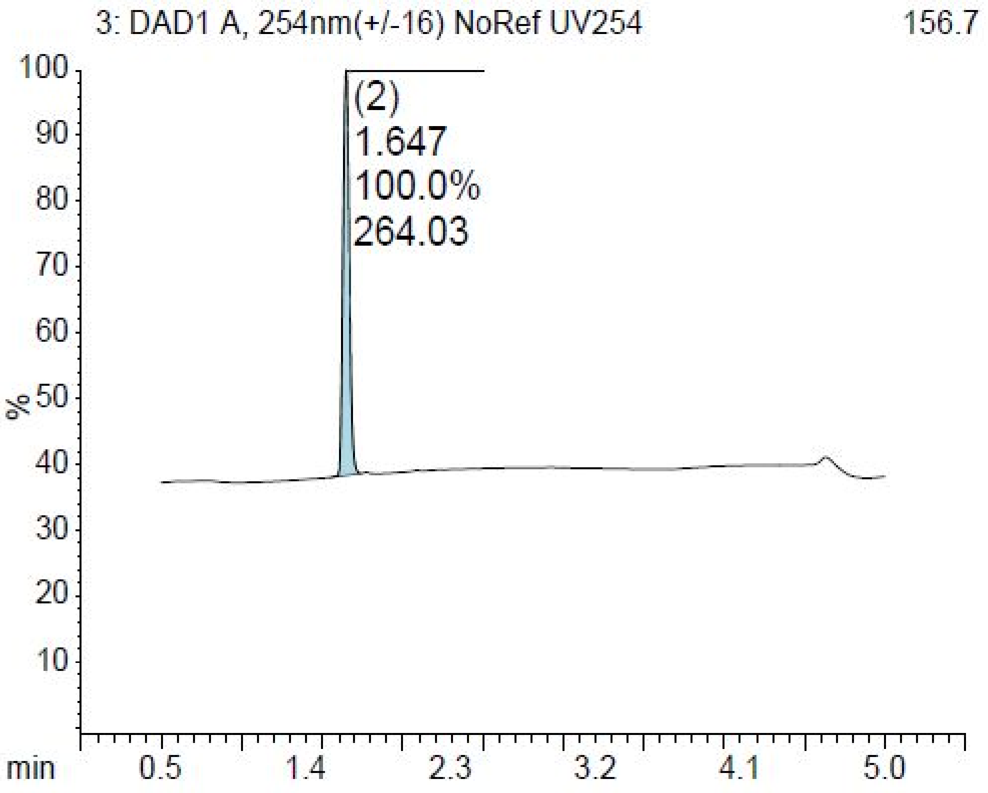

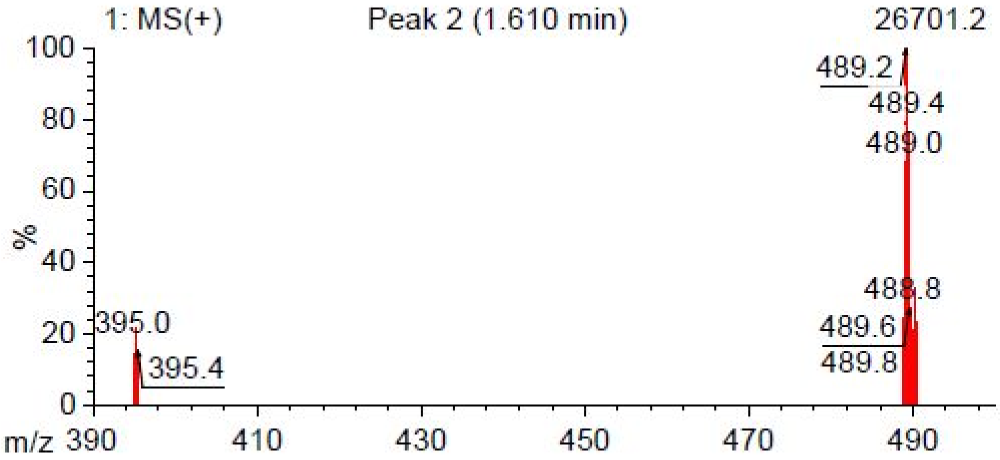

^{1}H NMR spectra for compound 87

EXP- 19-HE94078A

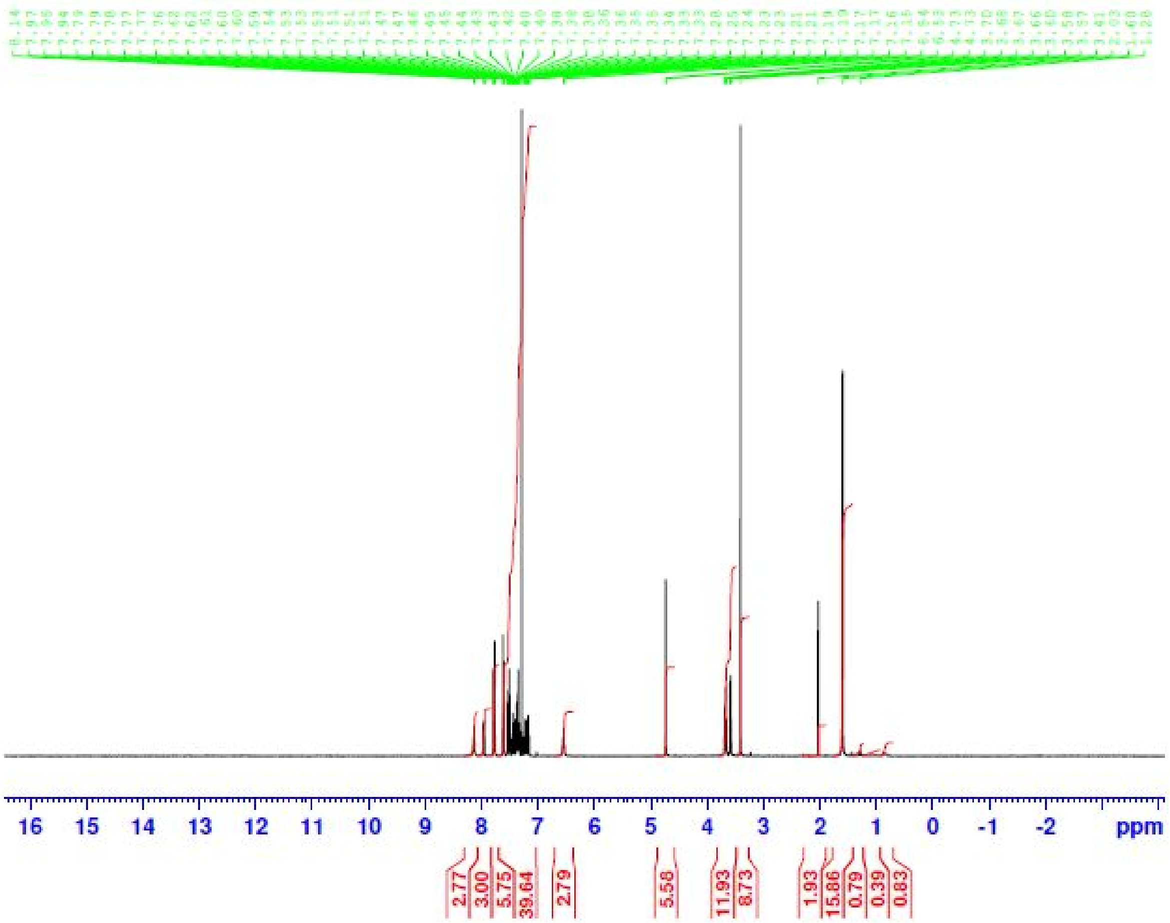

LCMS spectra for compound 87

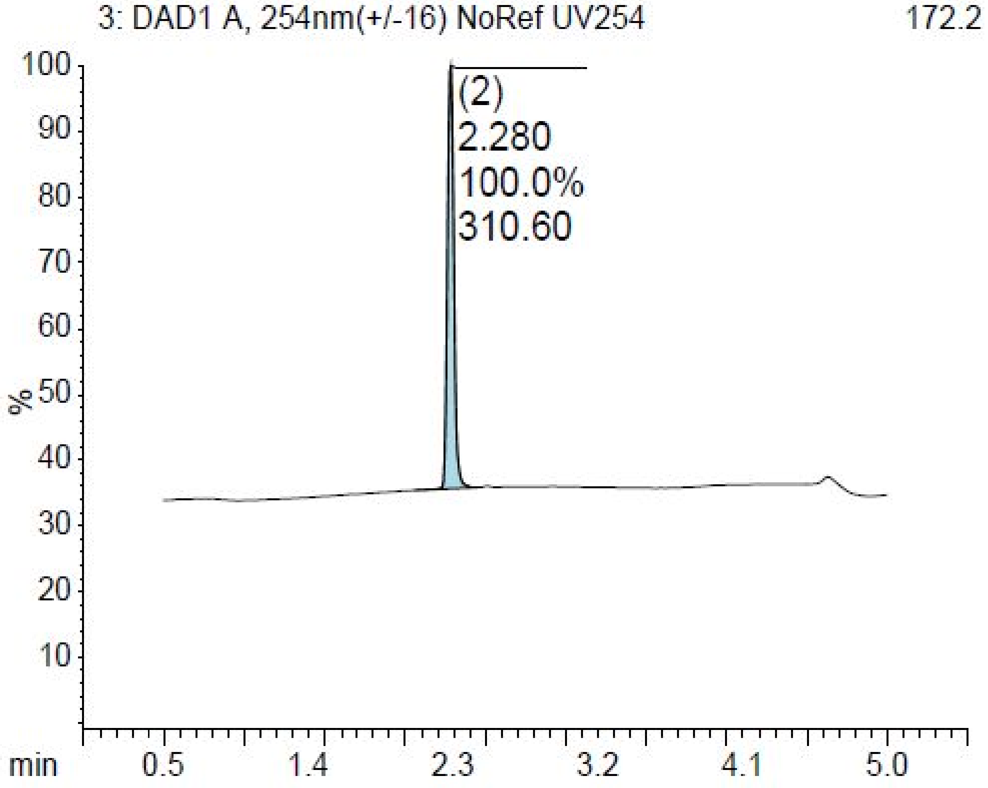

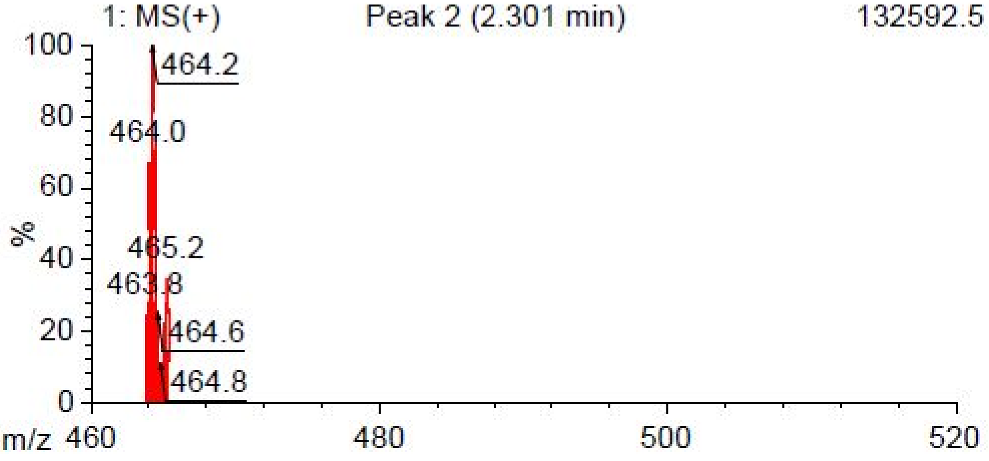

^{1}H NMR spectra for compound 88

EXP- 19-HE9409A

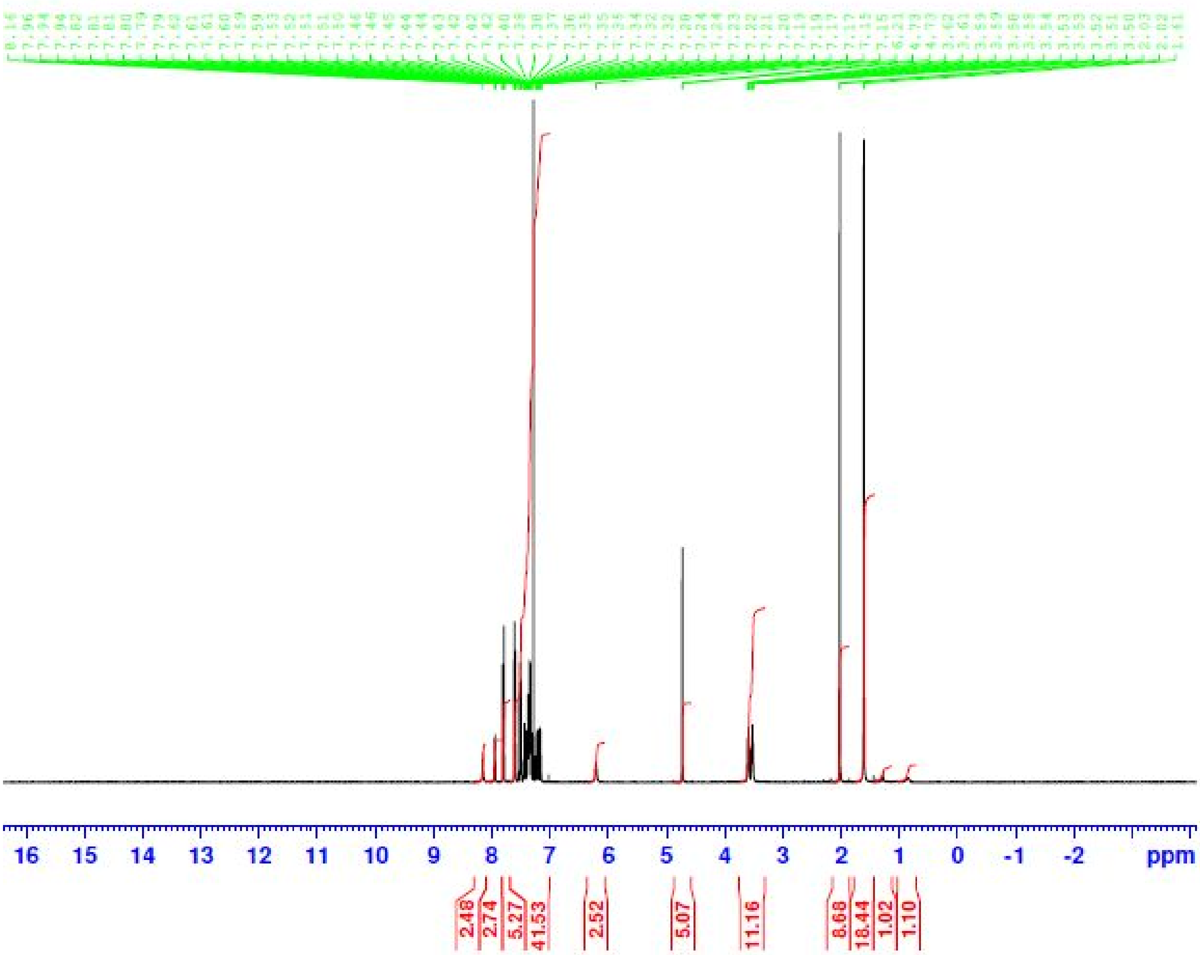

LCMS spectra for compound 88

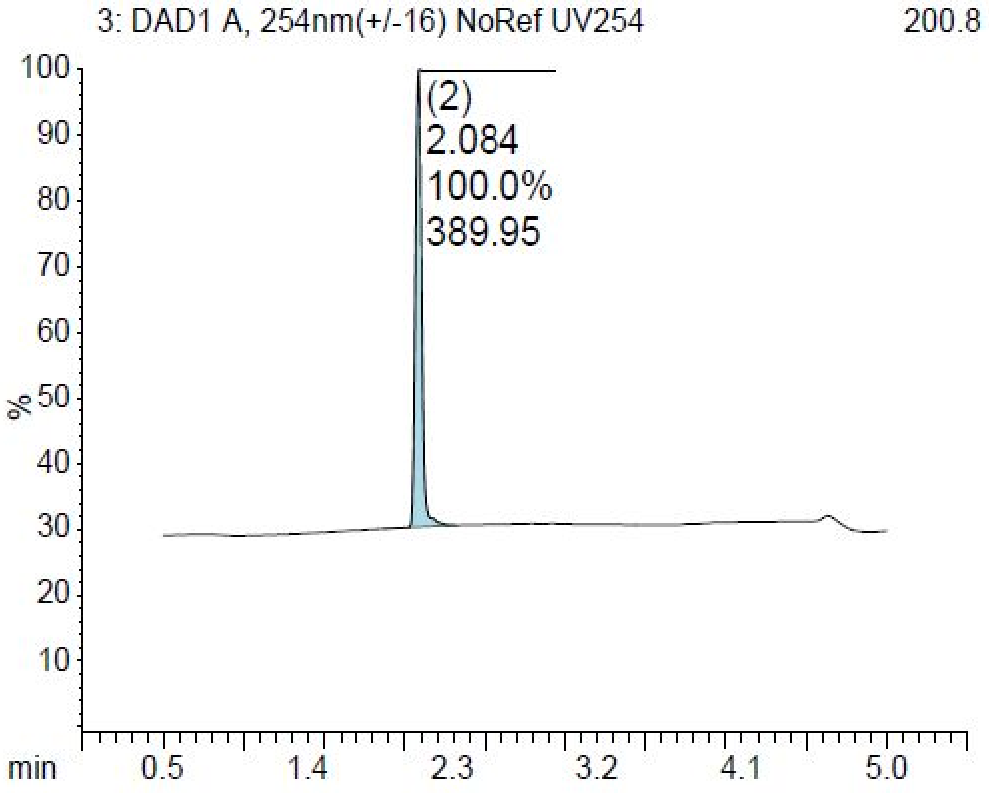

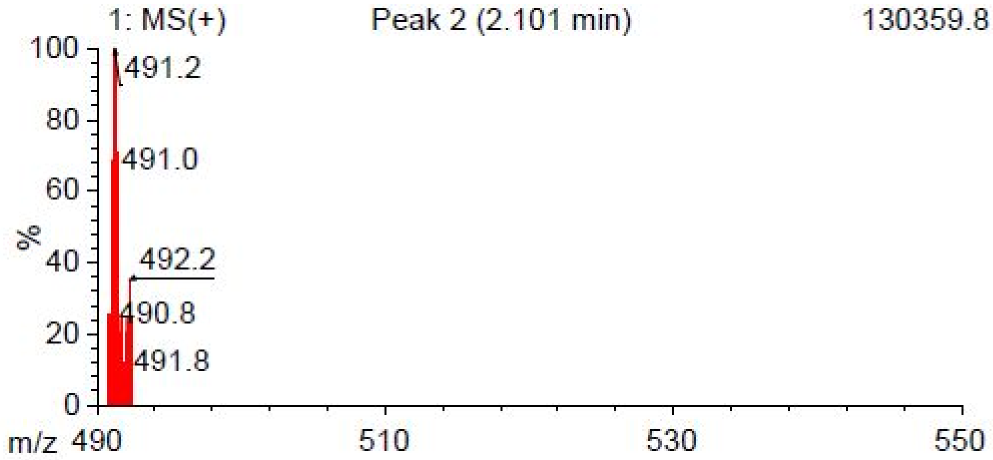

¹H NMR spectra for compound 89

EXP- 19HE9410A

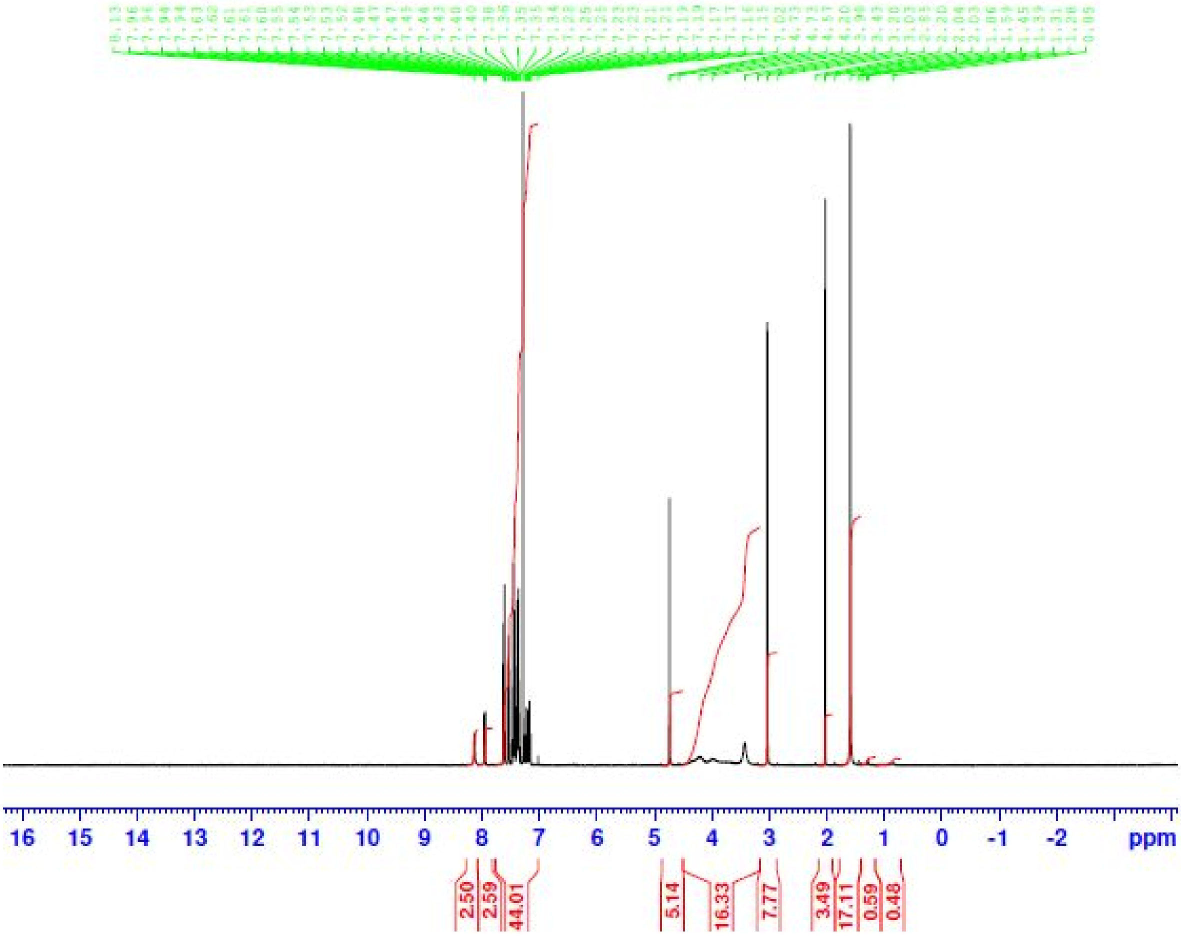

LCMS spectra for compound 89

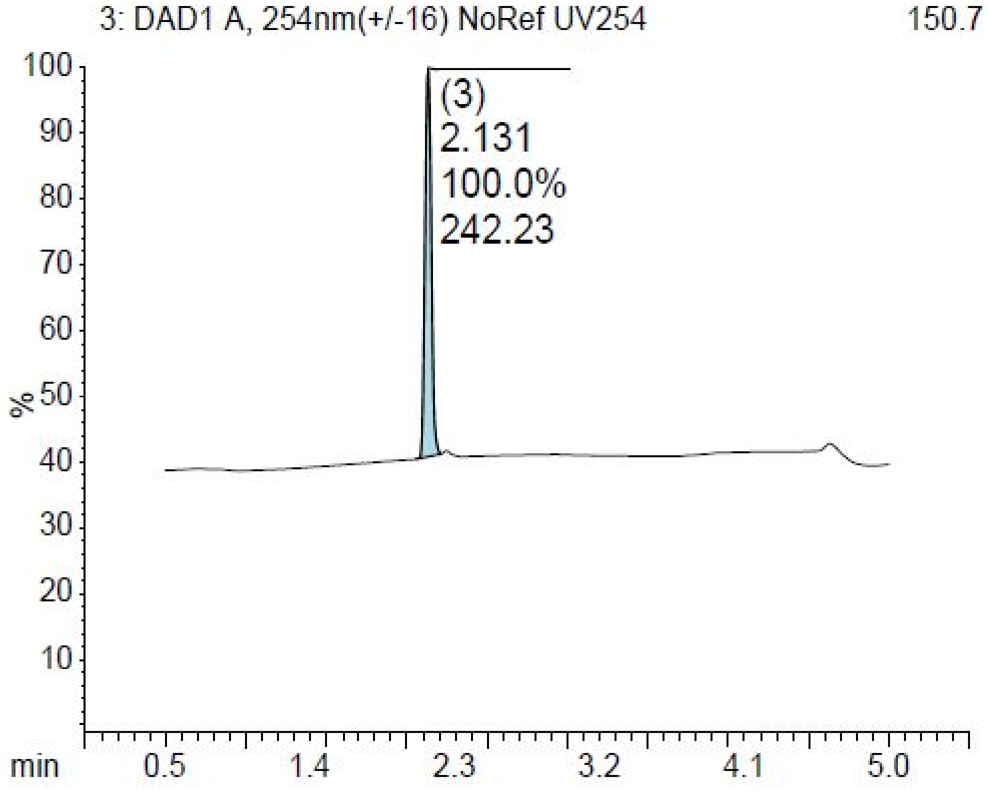

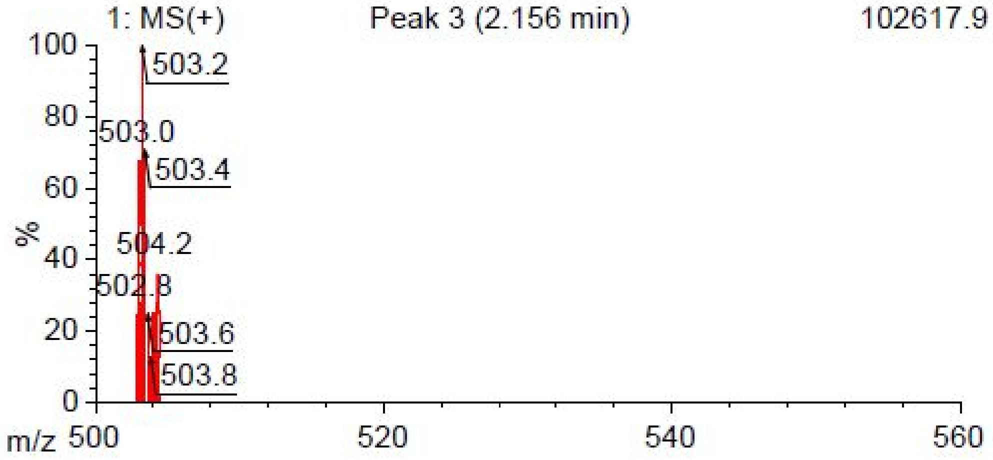

^{1}H NMR spectra for compound 90

EXP- 19HE9411A

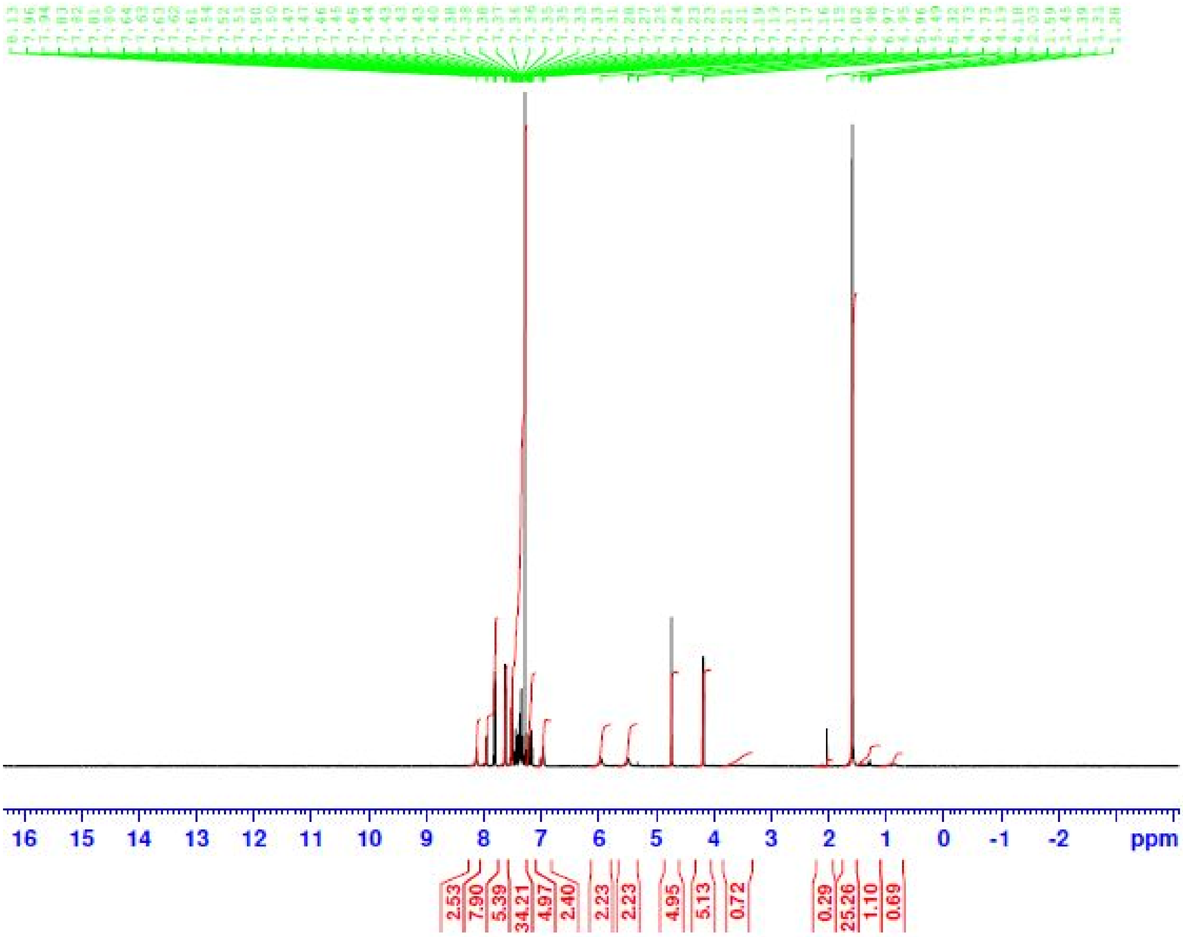

LCMS spectra for compound 90

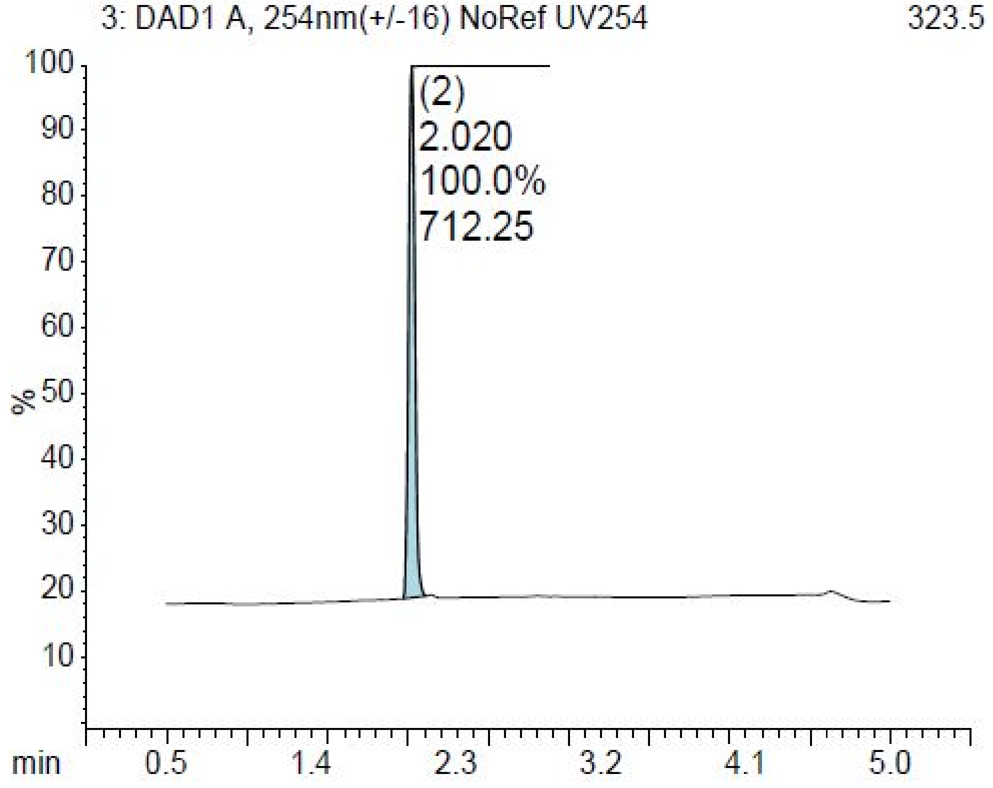

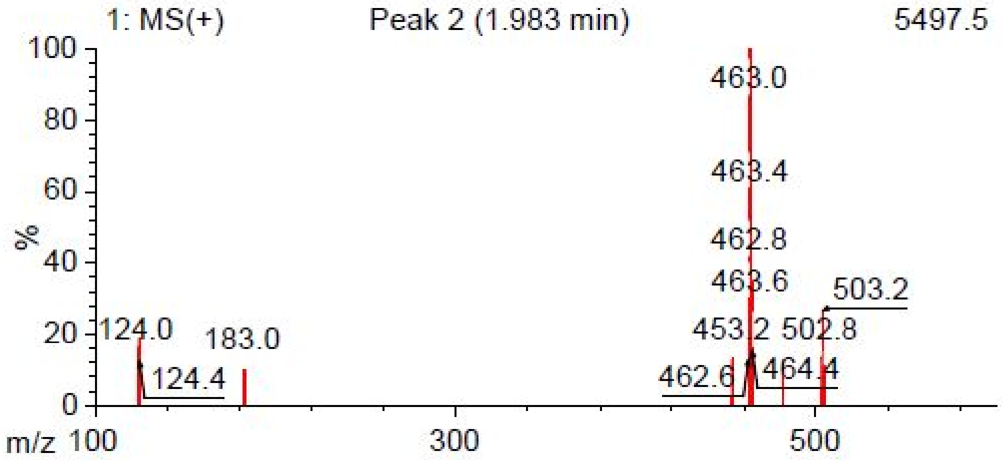

III. Antagonist activity at mouse GPR84

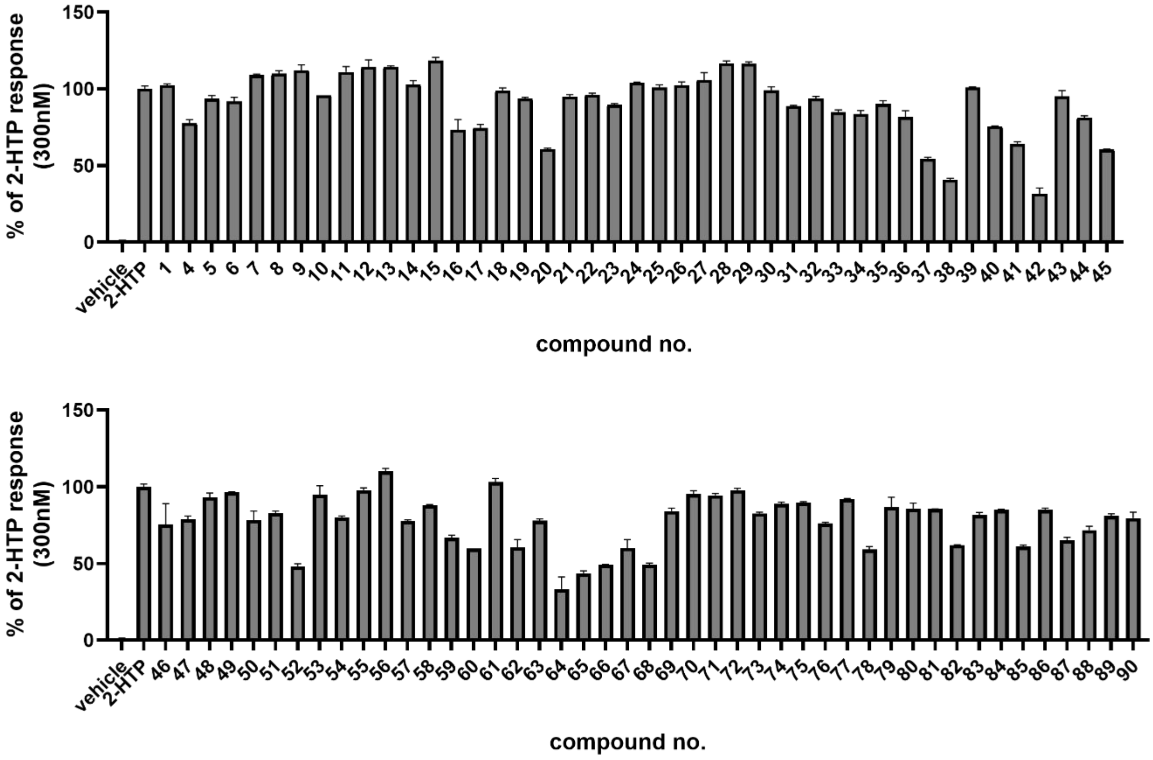

Fig S1: **Antagonist activity at mouse GPR84.**

[^{35}S] GTPγS binding was performed on membrane preparations from Flp-In T-REx 293 cells induced to express mouse GPR84-Gα_{i2}. Very few compounds (tested at 10 μM) were able to block effects of 2-HTP.
